# *Giardia’s* primitive GPL biosynthesis pathways with parasitic adaptation ‘patches’: implications for *Giardia*’s evolutionary history and for finding targets against *Giardia*sis

**DOI:** 10.1038/s41598-017-10054-1

**Published:** 2017-08-25

**Authors:** Qingqing Ye, Haifeng Tian, Bing Chen, Jingru Shao, Yan Qin, Jianfan Wen

**Affiliations:** 1 State Key Laboratory of Genetic Resources and Evolution, Kunming Institute of Zoology, Chinese Academy of Sciences, Kunming, Yunnan 650223 China; 2 Kunming College of Life Science, University of Chinese Academy of Sciences, Kunming, Yunnan 650204 China; 3 Kunming Institute of Botany, Chinese Academy of Sciences, Kunming, Yunnan 650201 China

## Abstract

*Giardia* is a worldwide spread protozoan parasite colonizing in small intestines of vertebrates, causing *Giardia*sis. The controversy about whether it is an extremely primitive eukaryote or just a highly evolved parasite has become a fetter to its uses as a model for both evolutionary and parasitological studies for years. Glycerophospholipid (GPL) synthesis is a conserved essential cellular process, and thus may retain some original features reflecting its evolutionary position, and this process should also have undergone parasitic adaptation to suit *Giardia*’s dietary lipid-rich environment. Thus, GPL synthesis pathways may be a perfect object to examine the controversy over *Giardia*. Here, we first clarified *Giardia*’s previously confusing GPL synthesis by re-identifying a reliable set of GPL synthesis genes/enzymes. Then using phylogenetic and comparative genomic analyses, we revealed that these pathways turn out to be evolutionarily primitive ones, but with many secondary parasitic adaptation ‘patches’ including gene loss, rapid evolution, product relocation, and horizontal gene transfer. Therefore, modern *Giardia* should be a mosaic of ‘primary primitivity’ and ‘secondary parasitic adaptability’, and to make a distinction between the two categories of features would restart the studies of eukaryotic evolution and parasitic adaptation using *Giardia* as a model system.

## Introduction

*Giardia* is a widespread intestinal protozoan parasite in human and many other vertebrates, causing one of the most common parasitic diseases – giardiasis. Besides its medical importance, *Giardia* was once regarded as one of the earliest divergent eukaryotes due to having many so-called ‘simple’ and ‘prokaryote-like’ features: 1) the simplicity in cellular structures, such as lack of some cellular organelles (e.g. mitochondrion) and poorly developed endomembrane system; 2) the prokaryote-like metabolic pathways; 3) the basal position on molecular phylogenetic trees. And thus some authors even considered it as a ‘biological fossil’ and to be a valuable model for providing insight into the evolution of eukaryotic cells^1, 2^. However, the lack of mitochondrion was later refuted by the discovery of the vestigial mitochondrial organelles – mitosomes^3^; the metabolic similarity to prokaryotes was proved to be most probably a result of horizontal gene transfer^4^; and the basal position was also attributed to be an artifactual result of long-branch attraction by some researchers^5^. Many authors, therefore, thought of it as just a highly evolved parasite with many parasitic reductions^6, 7^. But some other researchers still persisted in the ‘primitive’ opinion and emphasized *Giardia*’s special significance for the study of the evolution of eukaryotic cells^2, 8–10^. Obviously, the bone of contention is whether the simple or prokaryote-like features are a reflection of *Giardia*’s primitivity or just the consequences of parasitic reduction or adaptation. This debate has become a fetter to its uses as a model system for both evolutionary and parasitological studies for years.

Glycerophospholipids (GPLs) not only are the major structural components of biomembranes, but also play important roles in many important cellular processes^11–13^. As an essential conserved cellular activity, GPL biosynthesis can be expected to retain some original features that can reflect *Giardia*’s primitivity if this organism is really an ancient eukaryote. On the other hand, because *Giardia* lives parasitically in the dietary lipid-rich intestine, it can acquire GPLs directly from the environment without the necessity to entirely biosynthesize them, and thus its GPL biosynthesis pathways should have been subjected to parasitic reduction. Therefore, GPL biosynthesis pathways in *Giardia* might be a perfect object to examine the above controversy.

Unfortunately, GPL synthesis in *Giardia* is still enigmatic. It was early thought to be unable to synthesize phospholipid *de novo* ^14^, and later showed to be probably able to assimilate exogenous PC, PI and SM, and remodel them through Lands cycle and headgroup exchange reactions^15–18^. However, the identification of the two enzymes (Pgps and Psd) and the lack of PG and PE in conventional culture medium^19^ both suggest that *Giardia* has the ability to synthesize some phospholipids *de novo*. Lykidis (2007) once reconstructed partial GPL synthesis pathways of *G. lamblia*, and Yichoy *et al*.^23^ also described *Giardia* GPL synthesis pathways. But it is not until 2013 that the overall GPL synthesis pathways of prokaryotes and eukaryotes are nearly explicit^20–22^, which had seriously limited the previous identification of the homologous genes/enzymes in *Giardia*, and thus the previously reconstructed pathways could not be complete and were even inconsistent in different literatures.

Here, based on the genome database, by a strict identification process, we re-identified the genes/enzymes involved in GPL biosynthesis of *G. lamblia* and reconstructed much more reliable synthesis pathways; as a result of phylogenetic and comparative genomic analyses, we found that *Giardia*’s GPL biosynthesis pathways are very simple indeed and possess some bacteria-like enzymes and steps, but the simplicity turns out to be due to both *Giarida*’s primitivity and parasitic reduction. The implications of these results for the evolutionary position of *Giardia* and for finding targets against giardiasis are discussed.

## Results

### GPL biosynthesis genes/enzymes re-identified in *Giardia*

Through our strict identification process, as many as eighteen homologous GPL biosynthesis enzymes were re-identified in *Giardia*. They were named mainly after their yeast homologs (Table 1).

**Table 1:** The re-identified GPL biosynthesis homologous enzymes in *Giardia lamblia ATCC 50803*.

Pathway	Name	Domain (Pfam)	Localization*	NCBI Ac. No.	Expression evidence
PA pathway	*Gi*Slc1	Acyltransferase	2(160971-162272)(+)	XP_001707002.1	EST, RNA-seq, Microarray, Mass spec
CDP-DAG Pathway	*Gi*Cds	CTP_transf_1	3(241108–242238)(−)	XP_001706909.1	Microarray, RNA-seq, Mass spec
	*Gi*Pis	CDP-OH_P_transf	2(677822–678451)(−)	XP_001707169.1	Microarray, RNA-seq, SAGE
	*Gi*Pgps	CDP-OH_P_transf	2(158634–159527)(+)	XP_001707005.1	Microarray, RNA-seq, Mass spec
	*Gi*PgpB	PAP2	4(2057377–2058315)(−)	XP_001705389.1	EST, Microarray, RNA-seq, SAGE
	*Gi*Pss	CDP-OH_P_transf	4(1834745–1836481)(−)	XP_001707737.1	Microarray, RNA-seq, Mass spec
	*Gi*Psd	PS_Dcarbxylase	1(405550–406794)(−)	XP_001707910.1	EST, Microarray, RNA-seq, SAGE
Kennedy pathway	*Gi*Ek/Ck	Choline_kinase	4(2683198–2684226)(−)	XP_001704814.1	Microarray, RNA-seq, SAGE
Lands cycle	*Gi*LPCAT1	Acyltransferase	4(50798–52006)(+)	XP_001707326.1	Microarray, RNA-seq, SAGE, Mass spec
	*Gi*LPCAT2	Acyltransferase	4(2561304–2562464)(−)	XP_001704595.1	Microarray, RNA-seq
	*Gi*LPCAT3	Acyltransferase	2(1294424–1295698)(−)	XP_001704656.1	Microarray, RNA-seq, Mass spec
	*Gi*LCAT1	LCAT	4(2276173–2278491)(−)	XP_001705338.1	Microarray, RNA-seq, Mass Spec
	*Gi*LCAT2	LCAT	5(4376786–4379413)(+)	XP_001708468.1	EST, Microarray, RNA-seq, SAGE
	*Gi*LCAT3	LCAT	3(471497–474673)(+)	XP_001706263.1	RNA-seq, SAGE
	*Gi*LCAT4	LCAT	4(2602151–2608528)(−)	XP_001704693.1	EST, SAGE, RNA-seq, Mass Spec
	*Gi*PLB1	Phospholip_B	5(2242831–2244537)(−)	XP_001709220.1	Microarray, RNA-seq, Mass spec
	*Gi*PLB2	Phospholip_B	5(3533852–3535786)(−)	XP_001707417.1	EST, Microarray, RNA-seq, Mass spec
	*Gi*PLB3	Phospholip_B	4(2373778–2375412)(+)	XP_001704922.1	EST, Microarray, RNA-seq, Mass spec

Note: The symbol “*” indicates the location of the gene in the chromosome, which is expressed in the following principle: the digit before the first parenthesis indicates the chromosome number, the digit within the first parenthesis indicates the coordinates (start position and end position) on the chromosome, and the plus sign in the last parenthesis indicates the gene is in the plus strand of corresponding chromosome, while the alternative minus sign in the last parenthesis indicates the gene is in the minus strand of corresponding chromosome.

Eleven of them (*Gi*Slc1, *Gi*Cds, *Gi*Pis, *Gi*Pgps, *Gi*Pss, *Gi*Psd, *Gi*Ek/Ck, *Gi*LCAT1, *Gi*LCAT3, *Gi*PLB1and *Gi*PLB3) were confirmed to be the same as those already annotated in *Giardia*DB or reported in previous literatures^19, 23^, while seven others were newly identified here. The identification details of the newly discovered genes/enzymes are as follows:

#### *Giardia* Phosphatidylglycerophosphatase B (*Gi*PgpB)

One homolog (XP_001705389.1**)** to bacterial PgpB^24^ was found and confirmed to have six transmembrane helixes as *E. coli* PgpB^25^ and C1, C2 and C3 motifs (the activate sites of phosphatase).

#### *Giardia* Lecithin–cholesterol acyltransferase (*Gi*LCAT)

Four homologs (XP_001706263.1, XP_001705338.1, XP_001704693.1, XP_001708468.1) were identified to be group XV PLA2–Lecithin–cholesterol acyltransferase (LCAT), by reciprocal blast against nr database and their possession of LCAT domain (PF02450). Two of them (XP_001706263.1, XP_001705338.1) have been annotated previously. Here, we name them *Gi*LCAT 1, 2, 3, 4, respectively, based on the phylogenetic analysis (see below).

#### *Giardia* Lysophosphatidylcholine acyltransferase (*Gi*LPCAT)

Three homologs (XP_001707326.1, XP_001704595.1, XP_001704656.1) were identified to be lysophosphatidylcholine acyltransferase by reciprocal blast against nr database and their possession of the acyltransferase domain (Pfam: PF01553).

#### *Giardia* Phospholipase B (*Gi*PLB)

Three homologs (XP_001709220.1, XP_001707417.1, XP_001704922.1) were identified to be Phospholipase B by reciprocal blast against nr database and their possession of Phospholip_B domain (Pfam: PF04916). Two of them (XP_001709220.1, XP_001704922.1) have already been annotated as PLB in the genome database. Here, we named them *Gi*PLB 1, 2, 3, respectively.

### The evidence for the transcription or expression of *Giardia* GPL biosynthesis genes

By searching the EST, Mass spec, microarray and/or Serial Analysis of Gene Expression (SAGE) data in *Giardia*DB, the corresponding mRNA or proteins of all the eighteen identified *Giardia* genes were found to be present in at least two of these databases (Table 1), confirming that all these genes are real genes that can be expressed in *Giardia*.

### The cellular location of *Gi*Psd

**Figure 1:**
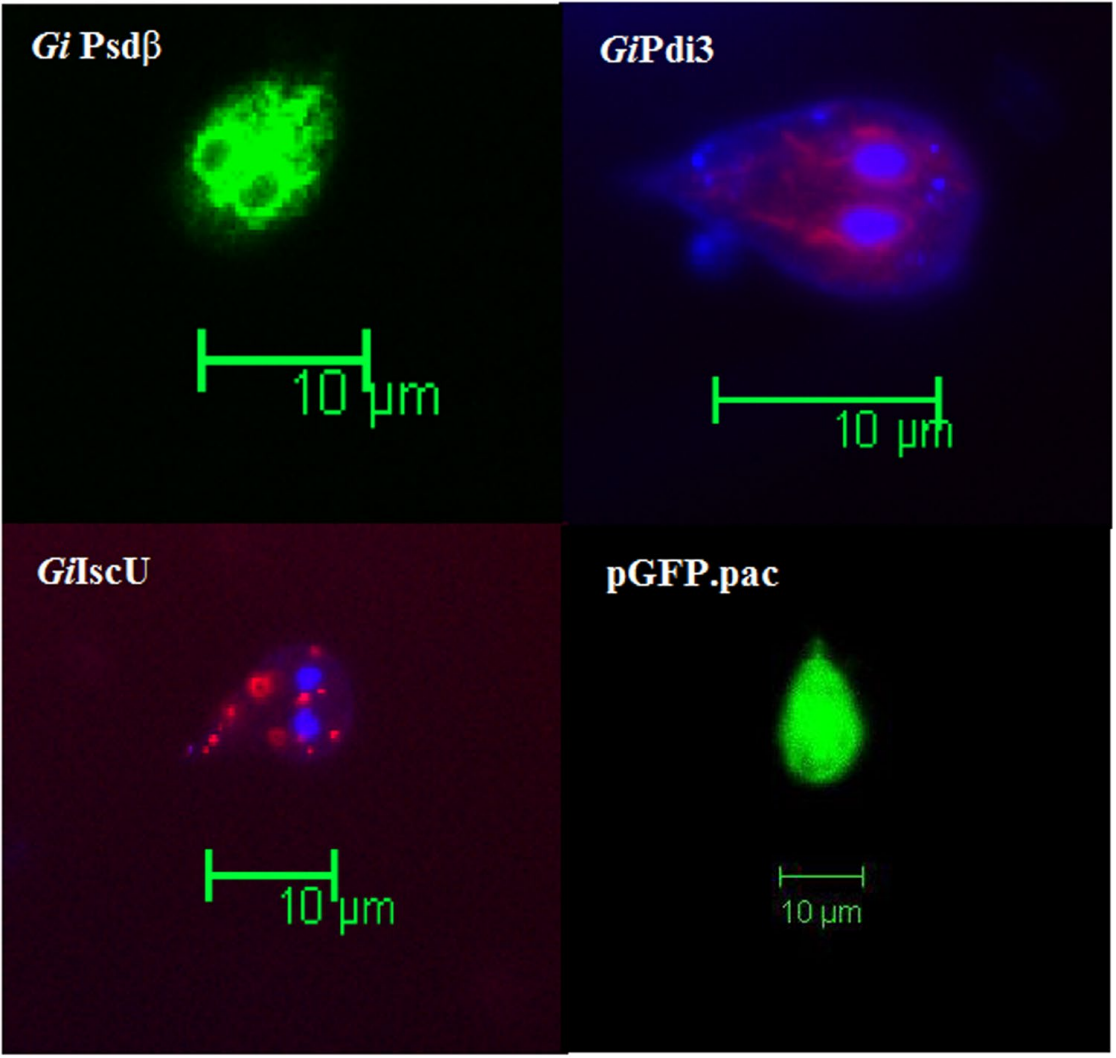
The subcellular localization of *Gi*Psd-GFP in trophozoites of *G. lamblia*. *Gi*Pdi3-DsRed and *Gi*IscU-DsRed are the monitors of ER and mitosome, respectively. The pGFP.pac transformed trophozoite is the control. Scale bar = 10 μm.

In eukaryotes, there are two types of Psd enzymes, which are exclusively located in mitochondrion (Psd1) and cytosolic Golgi/vacuole membrane (Psd2)^26^. *Gi*Psd should be a mitochondrial-type Psd according to its: 1) higher homology to Psd1 than to Psd2; 2) lacking Psd2 characteristic C2 domain; and 3) phylogenetic affinity with other experimentally verified mitochondria-localized Psd1. But *Giardia* does not possess typical mitochondria but remnant ones – mitosomes, so we want to examine the location of *Gi*Psd experimentally. The result showed its location pattern (Fig. 1) is similar to that of ER-localized *Gi*PDI3^27^, obviously different from that of mitosome-localized *Gi*IscU^3^. Therefore, *Gi*Psd is not located exclusively in mitosome but probably in the perinuclear/ER region of *Giardia*.

### Phylogenies of *Giardia* GPL biosynthesis enzymes

**Figure 2:**
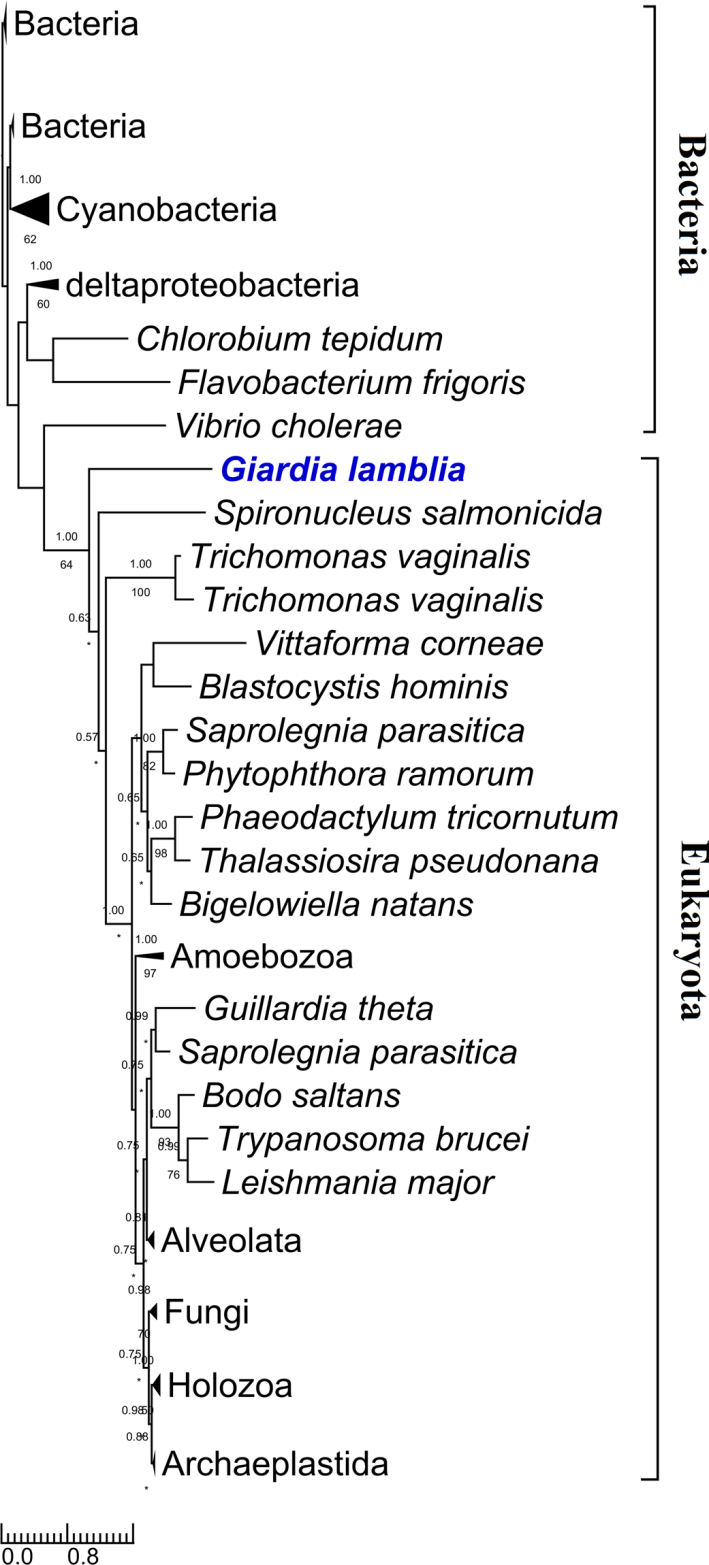
The rooted phylogenetic tree of the 127 obtained homologous sequences to Cds. Numbers above and below branches show posterior probabilities for Bayesian and bootstrap values for maximum likelihood respectively. Asterisks indicate values lower than 50%. Other values below 50% in both methods are not shown. The 211 conserved sites in the alignment were used for the tree construction. The tree was rooted using bacterial sequences as outgroup. Both RaxML and Mrbayes results show that *Giardia* (in bold and blue) is at the basal position of eukaryotes. Scale bar indicates number of change per site.

**Figure 3:**
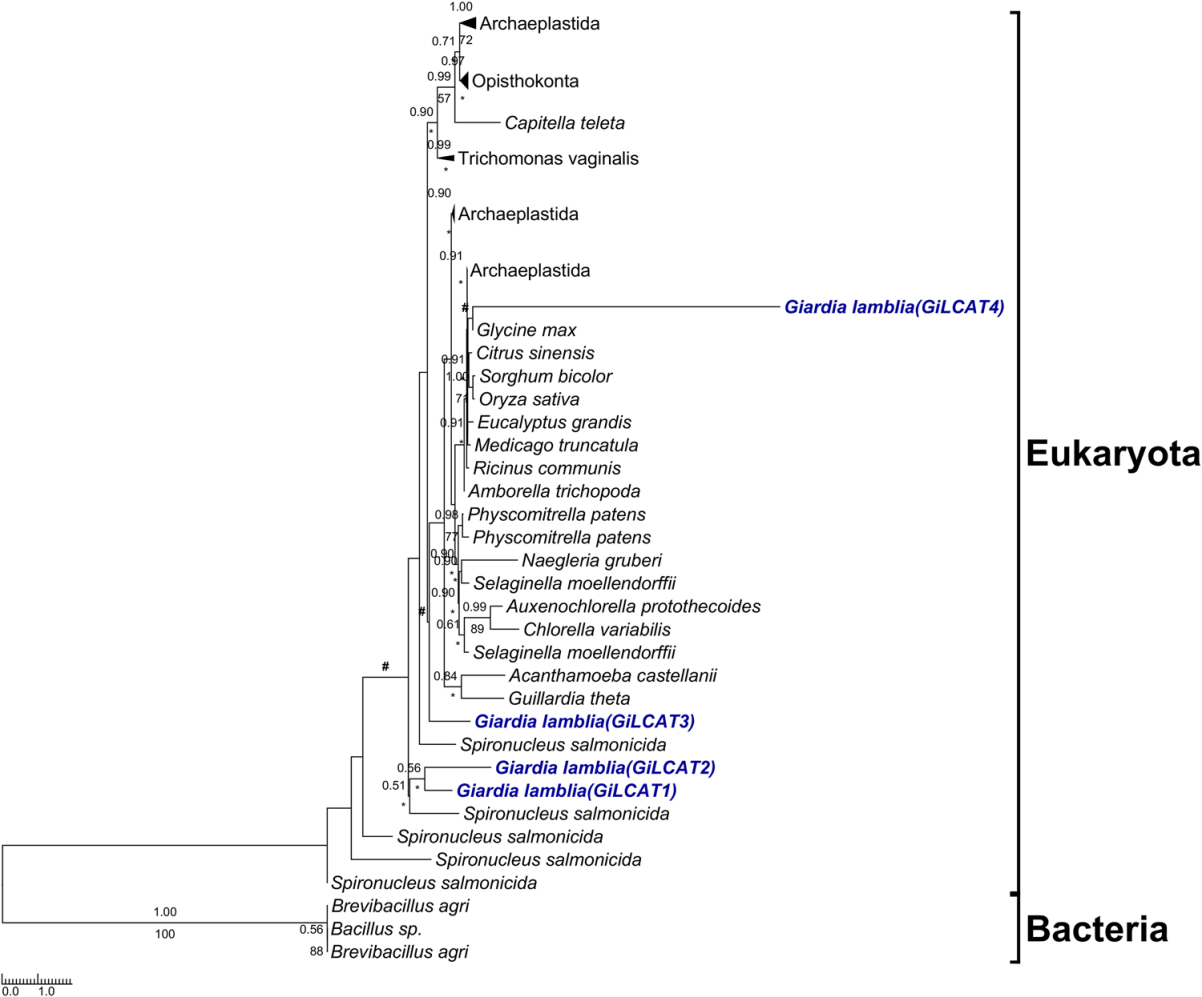
The rooted phylogenetic tree of the 94 obtained homologous sequences to LCATs. The tree is illustrated using the same conventions as in Fig. 2. The 99 conserved sites in the alignment were used for the tree construction. The symbols “#” above the branches indicate conflicts against the current displayed branching of *Gi*LCAT1-4 in the MrBayes tree. RAxML result shows that *Gi*LCAT1-3 reflect *Giarida*’s basal position among eukaryotes, while *Gi*LCAT4 has a very long branch and does not support *Giarida*’s basal position.

**Figure 4:**
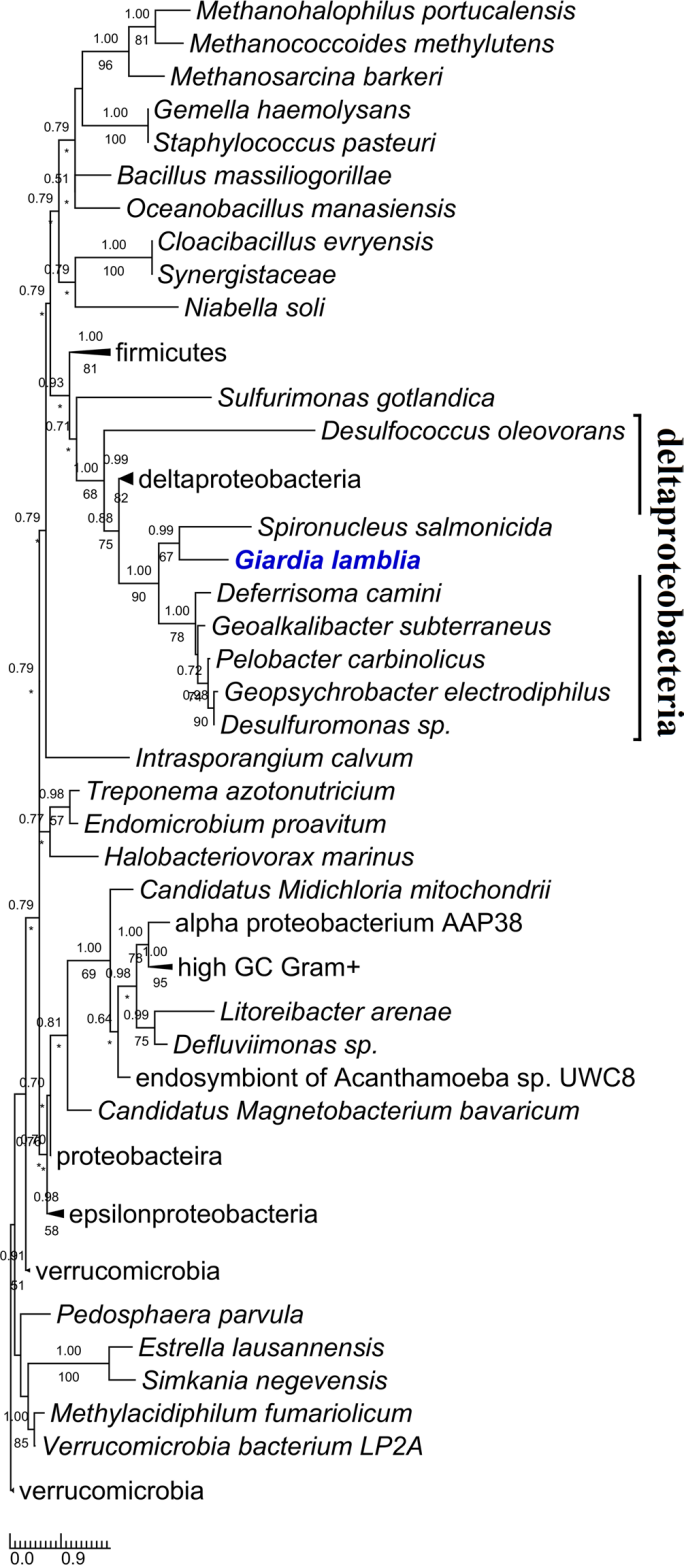
The rooted phylogenetic tree of the 70 obtained homologous sequences to Pss. The tree is illustrated using the same conventions as in Fig. 2. The 131 conserved sites in the alignment were used for the tree construction. Both RaxML and Mrbayes results show *Giardia* falls into the cluster of deltaproteobacteria, suggesting *Giardia*’s Pss (*Gi*Pss) (in bold and blue) was most probably acquired from deltaproteobacteria via horizontal gene transfer.

**Figure 5:**
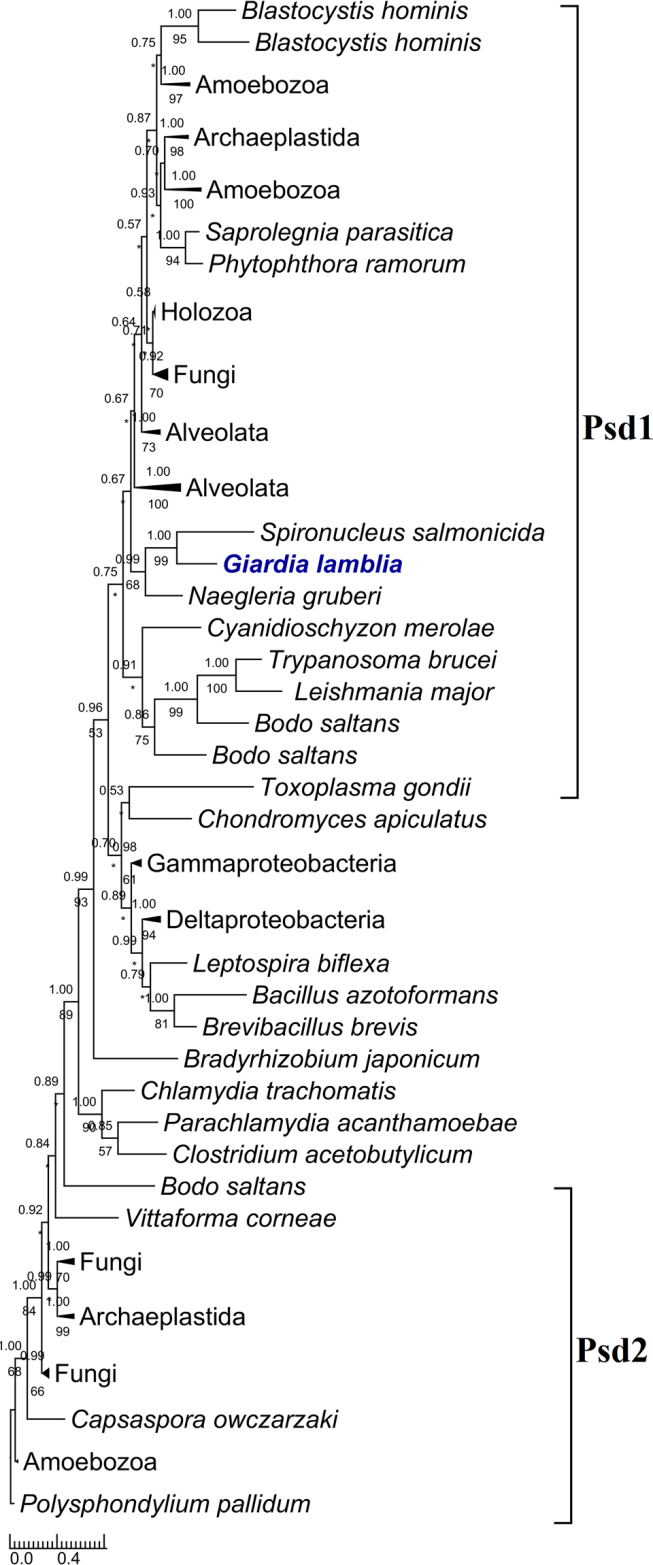
The rooted phylogenetic tree of the 95 obtained homologous sequences to Psd. The tree is illustrated using the same conventions as in Fig. 2. The 148 conserved sites in the alignment were used for the tree construction. This tree was rooted using Psd2 paralogs. Both RaxML and Mrbayes results do not show that *Gi*Psd (in bold and blue) is at the basal position of eukaryotic Psd1.

For all the eighteen *Giardia* enzymes, phylogenetic analyses were performed to explore their origins and evolutionary relationships with those of other organisms. The results are: 1) nine of them (*Gi*Cds, *Gi*LCAT1–3, *Gi*LPCAT1–3, *Gi*Ek/Ck, and *Gi*Pis) each were grouped closely with their corresponding eukaryotic homologs, and moreover each (often together with the homologs of *Giardia*’s close relative *Spironucleus* within Diplomonadida) were recovered by at least one of the two phylogenetic methods to branch off at the base of all eukaryotes (for four of the nine enzyme (*Gi*LCAT1–3, and *Gi*Pis), Mrbayes analysis did not recover that *Giardia* is at the base position. This is probably due to the low resolution of this phylogenetic method for the four enzymes, because multifurcation appeared on the corresponding trees). This suggests these nine enzymes were inherited from the eukaryotic common ancestor and still maintain the very primitive features reflecting *Giardia*’s early divergence from the eukaryotic trunk (Figs 2 and 3 (see *Gi*LCAT1–3); Supplementary Figs S1–3). 2) Three enzymes, *Gi*Pss, *Gi*Pgps, and *Gi*PgpB, were horizontally transferred from bacteria, and the donors of *Gi*Pss and *Gi*Pgps (together with the homologs of *Spironucleus*, a close relative of *Giardia*) are most probably deltaproteobacteria (Fig. 4) and Verrucomicrobia (Supplementary Fig. S4), respectively. As for *Gi*PgpB, no phylogenetic tree was reconstructed because of low similarities among all the obtained homologues. 3) Far away from *Gi*LCAT1–3, a member of the same *Gi*LCAT family–*Gi*LCAT4 formed a distinctively long branch alone on the phylogenetic tree (Fig. 3), implying this gene has once undergone a rapid evolution. 4) *Gi*Psd and *Gi*Slc1 branched later than some eukaryotes such as *Naegleria gruberi and Nematocida parisii* (Fig. 5 and Supplementary Fig. S5). 5) The phylogeny of PLB is very complicated (Supplementary Fig. S6), given many disagreements of its tree with common ones, such as the splits of some common monophyletic groups (e.g. Holozoa), which are probably due to its complicated evolutionary history or other unknown reasons, it is excluded from our analysis.

Therefore, it is obvious that all *Giardia* genes/enzymes can be mainly classified into two groups: 1) the primitive feature-keeping ones, which include nine genes/enzymes that were vertically inherited from the common ancestor of eukaryotes with relatively few changes; 2) the secondarily changed ones, which contain at least six genes/enzymes that either have undergone fast evolution and changed a lot or were secondarily horizontally transferred from bacteria.

### Reconstruction of *Giardia* GPL biosynthesis pathways

**Figure 6:**
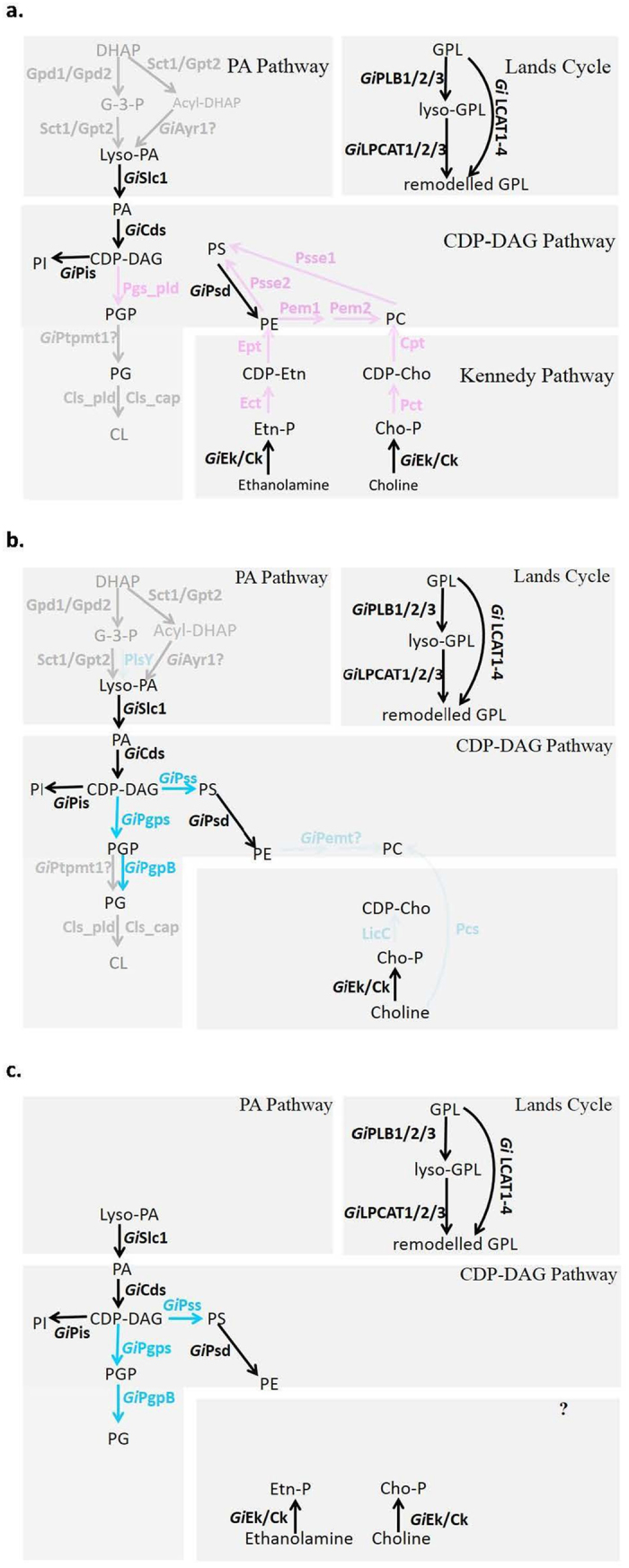
Reconstruction of *Giardia*’s GPL synthesis pathways. The identified *Giardia*’s genes/enzymes homologous to those of eukaryotes are posed on the typical eukaryotic GPL biosynthesis pathways (**a**), those homologous to those of bacteria are posed on the typical bacterial GPL biosynthesis pathways (**b**), and the reconstructed *Giardia*’s GPL synthesis pathways are showed in (**c**). *Giardia*’s genes/enzymes, homologous to those of both bacteria and eukaryotes are in dark, homologous to those specific to eukaryotes are in purple, and homologous to those specific to bacteria are in blue. The absent genes/enzymes and reactions in *Giardia* are in grayish-purple, greyish-blue, and grey, corresponding to their eukaryote-specific, bacterium-specific, and common to both eukaryotes and bacteria, respectively. The identified *Giardia* homologs with uncertain functional specificity are marked with “?”

According to the reported typical eukaryotic and bacterial GPL biosynthesis pathways, the identified *Giardia* genes/enzymes were used to reconstruct pathways (Fig. 6).

Compared with those of eukaryotes, *Giardia’*s pathways have the following features: 1) PA pathway only has the last step, not beginning with the G-3-P or DHAP but the intermediate lyso-PA (LPA) which can be acquired from the host’s digested foods in the duodenum (Fig. 6a). 2) CDP-DAG pathway has the same three steps: from PA to CDP-DAG then to PI, and from PS to PE, but lacks all the reactions catalyzed by eukaryote-specific enzymes (from CDP-DAG to PGP, from PE or PC to PS through base-exchange reactions, and from PE to PC through successive methylation) and the two universal steps: from PGP to PG and then to CL (Fig. 6a). But interestingly, instead, there exist three steps (from CDP-DAG to PGP and then to PG, from CDP-DAG to PS) catalyzed by three bacterial type enzymes, *Gi*Pgs, *Gi*PgpB, and *Gi*Pss, respectively, which all were laterally transferred form bacteria according to our domain and phylogenetic analyses above (Fig. 6b). 3) Kennedy pathway seems to only have the first step, lacking the rate-limiting second step and the third step (Fig. 6a), nevertheless the step can also belong to the bacterial LicC pathway^28^ (Fig. 6b). Therefore, whether *Giardia* really has Kennedy pathway is uncertain. 4) Lands cycle is almost the same as that of typical eukaryotes (Fig. 6a), suggesting *Giardia* can remodel the GPLs absorbed from host’s digested foods to generate its own GPLs.

By combining Fig. 6a and b, *Giardia*’s GPL biosynthesis pathways was finally reconstructed (Fig. 6c). Except Lands cycle, all the other three pathways lack some steps remarkably, especially the reactions catalyzed by eukaryote-specific enzymes, and thus, overall, *Giardia*’s GPL biosynthesis pathways are very simple or incomplete compared with those of typical eukaryotes.

### Phylogenetic distribution of the *Giardia*’s absent genes in prokaryotes and in *Giardia*’s closely related protozoans

To explore whether the absence of GPL biosynthesis genes in *Giardia* is a primitive feature or just due to parasitic reduction, we further investigated the phylogenetic distribution of these genes in Archaea, Bacteria (mainly in the hypothetical bacterial-ancestor-of-eukaryote co-descendants according to several hypotheses about the origin of eukaryotic cells^29–31^), the relatives of *Giardia* within Excavata (including both parasitic and free-living species), and some parasitic protozoans living in the similar environment as *Giardia* and their free-living relatives. The results are summarized in Table 2.

**Table 2:** Summary of the phylogenetic distribution of the *Giardia*’s absent GPL biosynthesis enzymes in other organisms.

Organism		PA pathway		CDP-DAG pathway					Kennedy Pathway	
		Gpd1/Gpd2(NAD^+^)	Sct1/Gpt2	Pgs_pld	Cls_cap	Cls_pld	Pem1/Pem2	Psse1/Psse2	Ect/Pct	Ept/Cpt
Bacteria*		+	+	−	+	+	−/−	−	−	−
Archaea**		+	+	−	−	+	+/−	−	−	−
Excavata	***Giardia lamblia*** **(P)**	−	−	−	−	−	−/−	−	−	−
	***Trichomonas vaginalis*** **(P)**	+	−	−	−	−	−/+	+	+	+
	***Trypanosoma brucei*** **(P)**	+	+	+	−	+	−/−	+	+	+
	***Leishmania major*** **(P)**	+	+	+	−	+	+/+	+	+	+
	***Bodo saltans*** **(F)**	+	+	+	−	+	+/+	+	+	+
	***Naegleria gruberi*** **(F)**	+	+	+	−	+	+/+	+	+	+
Amoebozoa	***Entamoeba dispar*** **(P)**	−	−	−	−	−	−/+	+	+	+
	***Entamoeba histolytica*** **(P)**	−	−	−	−	−	−/+	+	+	+
	***Acanthamoeba castellanii*** **(F)**	+	+	+	+	+	+/+	+	+	+

The symbol “*” indicates the hypothetical bacterial-ancestor-of-eukaryote co-descendants (we call them “the putative bacterial ancestor” in the text for short). For the details of the distribution of the enzymes in these bacteria please see the Supplementary Table S6. The symbol “**” indicates that all the genome-sequenced archaea organisms in NCBI so far. (P), parasitic; (F), free-living.

The genes for Gpd1/Gpd2(NAD^+^), Sct1/Gpt2, Cls_pld, and Pem1/Pem2 are present in the hypothetical prokaryotic ancestors of eukaryotes, and particularly also present in at least part of the investigated protozoans (especially the free-living species), suggesting that they had originated in the prokaryotic ancestors of eukaryotes and their absence in *Giardia* should be due to secondary loss. Moreover, their absence in parasitic Amoebozoa species (*E. dispar* and *E. histolytica*), which live in the similar intestinal environment as *Giardia*, further suggests they were most likely secondarily lost due to adaptation to intestinal parasitism.

The gene for Cls_cap is distributed in the hypothetical bacterial ancestors and the four eukaryotic supergroups Opisthokonta, Archaeplastida, SAR and Amoebozoa (Please see Supplementary Table S1), but none of the Excavata has it. Based on the notion that eukaryotes evolved from prokaryotes, it can be inferred that the last common ancestor of Excavata might have already lost this gene. Therefore, its absence in *Giardia* might be due to an ancient loss in the common ancestor of Excavata.

The gene for Pgs_pld is absent in the hypothetical bacterial ancestors. Within Excavata, parasitic species do not have them, while the free-living ones possess. Interestingly, within Amoebozoa similar distribution pattern was found: the intestinal parasitic species (*E. dispar* and *E. histolytica*) do not have, while the free-living species possess them. These suggest its absence in *G. lamblia* is most likely a secondary loss due to parasitism.

The last three genes for Psse1/Psse2, Ect/Pct, and Ept/Cpt, are also absent in the hypothetical prokaryotic ancestors, but present in all the other Excavata and all the Amoebozoa investigated, either free-living or parasitic. This suggests that their absence in *Giardia* might not have tight connection with parasitism, and that they might have not evolved in the bacterial ancestors of eukaryotes and *Giardia* yet, but later emerged in the eukaryotes after the divergence of *Giardia* from the eukaryotic trunk. This inference is consistent with the proposition of Hampl *et al*. (2009)–“No matter where the root of eukaryotes lies, our results indicate that Excavates are not uniquely related to any one of the other 5 iconic supergroups, and, if monophyletic, stemmed from a very deep branching event within the history of Eukaryotes”. However, if that the position for the root of the eukaryotic tree is between unikonts and bikonts is correct, the presence of the three genes in the investigated Amoebozoa (belonging to unikonts) and other Excavata organisms (belonging to unikonts) seems to imply that the absence of these genes in *Giardia* can also be explained to be due to secondary loss. But that the parasitic Amoebozoa investigated here (*E. dispar* and *E. histolytica*), which live in the same intestinal environment just as *Giardia*, do not lose these genes suggests the absence of these genes in *Giardia* is unlikely due to the parasitic loss. Therefore, their absence in *Giardia* might be a primary trait.

In conclusion, all the *Giardia*’s absent GPL biosynthesis genes can also be divided into two groups: 1) the primarily absent ones, which had not yet evolved in *Giardia* and might be the reflection of *Giardia*’s primitivity; 2) the secondarily lost ones, which were lost in *Giardia* mostly due to parasitic reduction.

## Discussion

### Clarification of the previously confusing GPL biosynthesis of *Giardia*

Here, much more complete sets of genes for GPL biosynthesis enzymes in eukaryotes and bacteria, especially including the recently experimentally verified ones, were used as baits to search for *Giardia*’s homologous genes, and the candidate were further confirmed via a more strict process. Finally, besides all the previously annotated or reported genes/enzymes, as many as seven new genes/enzymes were found out, and meanwhile a previously mistakenly identified *Gi*Pss was re-identified: *Gi*Pss was re-identified to be a bacterial-type enzyme rather than to be an eukaryotic enzyme possessing Psse1 and Psse2 activities as reported by Yichoy *et al*.^23^. Therefore, the common eukaryotic base-exchange steps from PC or PE to PS catalyzed by Psse1 or Psse2 do not exist in *Giardia* yet (Fig. 6a), instead, *Giardia*’s PS is formed through a bacteria-specific reaction–condensation of CDP-DAG with serine catalyzed by the bacterial-type *Gi*Pss (Fig. 6b and c).

Our reconstructed pathways (Fig. 6c) indicate that *Giardia* almost cannot biosynthesize its GPLs *de novo*, but can either apply its Lands cycle to remodel exogenous GPLs or use host’s intermediate products of dietary lipid digestion–lyso-PA to synthesize some GPLs mainly through a simple/incomplete CDP-DAG pathway. Thus, our work has clarified the previously confusing GPL biosynthesis of *Giardia*.

### *Giardia*’s primitive GPL biosynthesis pathways with secondarily parasitic adaptation ‘patches’

Nine of the *Giardia* GPL biosynthesis genes/enzymes were showed to be at the base of their corresponding phylogenetic trees, suggesting that they are very primitive among eukaryotic homologs. Consisting of so many primitive genes/enzymes, these *Giardia*’s pathways, therefore, should be ancient ones among eukaryotes.

In addition, our phylogenetic distribution investigation indicated that the three eukaryotic-specific enzymes–Psse1/Psse2, Ect/Pct, and Ept/Cpt are most likely to have not evolved in *Giardia* yet, though the possibility of secondary loss can not be absolutely excluded. Thus the eukaryotic-specific Kennedy pathway seems to have not yet evolved. These also suggest that *Giardia*’s GPL biosynthesis pathways are ancient indeed.

Therefore, *Giardia*’s GPL biosynthesis pathways are quite ancient and retain many primitive features of the early eukaryotic cells.

On the other hand, interestingly, our work showed that *Giardia*’s GPL biosynthesis genes and pathways have undergone obvious secondary parasitic adaptation, which can be seen from the following perspectives.

First, **Pathway Reduction via Gene Loss:** Our analysis indicated that the absence of Gpd1/Gpd2, Sct1/Gpt2, Pgs_Pld, Cls_pld, Cls_cap, and Pem1/Pem2 in *Giardia* is most likely caused by secondary losses. Some of the losses can be reasonably attributed to parasitic adaptation. For example, the losses of Gpd1/Gpd2, Sct1/Gpt2 may be due to the un-necessity for *Giardia* to synthesize PA *de novo*, since the intermediate product–lyso-PA is rich in the intestinal milieu^32^, and can be absorbed through receptor-mediated endocytosis by *Giardia* ^33^. Besides, the loss of Cls_pld and Pgs_pld may be in company with *Giardia*’s reduction of mitochondria for adaptation to anaerobic respiration^34^.

Second, **Relocation of Enzyme:** The close affinity with common eukaryotic mitochondrion-localized Psd and the predicted mitochondrial location of *Gi*Psd both suggest that this enzyme is mitochondrion-located as common eukaryotic Psd1. However, our experiments indicated that this enzyme is not localized in mitochondrion-derived mitosome exclusively but probably in the perinuclear/ER region. Therefore, *Gi*Psd might have undergone secondarily subcellular re-localization, in company with the parasitic reduction of mitochondrion.

Third, **Rapid Evolution of Genes and Gene Transfer from Bacteria:** The phylogenetic analysis showed that within the *Gi*LCAT family, *Gi*LCAT4 might have undergone a secondary accelerated evolution, though the direct correlation of this with *Giardia*’s parasitic adaptation is unclear yet. Besides, *Gi*Pss, *Gi*Pgps, and *Gi*PgpB were showed to be secondarily acquired through HGT from bacteria. Thus, like some other bacteria-like enzymes, whose presences were once considered as the reflection of *Giardia*’s primitivity^35^ but were later proved to be result of HGT from bacteria^4^, here, all these ‘bacteria-like’ enzymes are also showed to be secondarily acquired from bacteria via HGT. The three genes might have advantage in adaptation to anaerobic/microaerophilic conditions in bacteria originally, and were later transferred to *Giardia* probably for better fitting *Giardia*’s parasitic microaerophilic condition. What is more interesting, not having the eukaryotic Psse1/Psse2 genes (this has been inferred above most likely as a primitive feature), by secondarily acquiring the bacterial-type *Gi*Pss via HGT, *Giardia* got a bacterial-specific reaction to synthesize its PS (Fig. 6b and c) actually, which might have a further advantage in the parasitic adaptation.

Overall, *Giardia’*s GPL biosynthesis genes and pathways show an apparent “duality”: “the primary primitivity” and “the secondary parasitic adaptation”. This means that during *Giardia*’s transformation from free-living lifestyle to parasitism–the common evolutionary route of parasites, on the basis of the primitive GPL biosynthesis genes and pathways a series of secondary parasitic adaptation ‘patches’ have been ‘sewed’.

### Implications for *Giardia*’s evolutionary history and for finding targets against giardiasis

As mentioned in Introduction, the bone of contention regarding *Giardia* is whether the ‘simple’ or ‘prokaryote-like’ features are a reflection of *Giardia*’s primitivity or just the consequences of parasitic adaptation.

It is true that some of the early reported ‘simple’ features have been proved to be the results of parasitic reduction. The lack of mitochondria, for instance, was proved to be highly-reduced mitochondria in response to the oxygen-poor niche^36^. But, not all such features can be attributed to parasitic reduction. For example, the reported lack of anaphase-promoting complex can not be inferred to be the result of parasitic degeneration considering the same absence in *Giardia*’s non-parasitic relative *Spironucleus vortens* and the noticeable presence in the obligate intracellular parasite *Toxoplasma gondii* ^9^; The simplicity of *Giardia*’s 5 S rRNA system can not be inferred to be the result of parasitic reduction yet^37^. Therefore, modern *Giardia*’s simplicity might be a combination of the primitive simplicity and secondary loss of complexity due to parasitism. Here, our work, for the first time, has proved this conjecture with practical evidence, by revealing that its simplicity/incompleteness of the GPL biosynthesis system is due to both primitivity and secondary parasitic reduction.

As for the prokaryote-like features, as mentioned above we do found three bacteria-like enzymes (*Gi*Pss, *Gi*Pgps, and *Gi*PgpB) and even a bacterial-specific PS synthesis reaction, but has proved them to be secondarily acquired from bacteria via HGT, which is another sort of parasitic adaptation, not to be due to *Giardia*’s primitivity at all.

Therefore, our work strongly suggests that *Giardia* is a very early-branching eukaryote and hence retains many primitive features – the ‘primitive simplicity’; on the other hand, during its becoming a very successful parasite from free-living precursor, it has undergone a series of parasitic adaptations including: reduction resulting in another kind of simplicity – the ‘reductive simplicity’, horizontal gene transfer resulting in the similarity to prokaryotes, and so on. In fact, some authors had once proposed that although parasitic reduction is the main contributor to the minimal systems found in *Giardia* spp., the simple organization may also reflect some evolutionarily basic characteristics^8^. But for the first time, it is the present work here that has provided practical evidence for this mosaic simplicity of this organism. We believe studies from more aspects of *Giardia* would provide more evidence for this viewpoint. Therefore, actually *Giardia* is really an early-diverging eukaryote with many remnant primitive features, and on the basis of this primitivity, it has acquired many secondary parasitic adaptive features.

Not making a distinction between the ‘primitive simplicity’ and the ‘reductive simplicity’ has affected *Giardia*’s studies on both eukaryotic evolution and parasitic adaptation. As we all know, it is the primitive features that are useful in the eukaryotic evolutionary study, contrarily, it is the parasitic features, rather than the primitive ones, that can be important objects for discovering drug targets against giardiasis. Since parasitic adaptive features: 1) arise from the adaptation to the parasitic lifestyle, they are very important to the successful parasitism of parasites; 2) have no counterparts in hosts, they can be important objects for developing specific and efficient antiparasitic drugs or vaccines. As a sort of parasitic adaptive features, horizontally transferred genes in parasites are often used for this task. Therefore, the bacteria-like enzymes (*Gi*Pss, *Gi*Pgps, and *Gi*PgpB), which were identified here to be transferred from bacteria for parasitic adaptation rather than to be due to *Giardia*’s primitivity, might be potential targets against giardisis.

Therefore, to make a distinction between the two categories of features in *Giardia* may endow this organism with a unique dual value: its primitive parts can be used as valuable models to study the evolution of many aspects of the eukaryotic cell, while its parasitic adaptive parts can be applied to finding targets against *Giardia*sis.

## Methods

### Identification of *Giardia* GPL biosynthesis genes/enzymes

A comprehensive pipeline using a combination of BLAST and HMMER was created to search for *Giardia* GPL biosynthesis genes/enzyme homologs. 1) A local similarity research with BLAST software^38^ against the downloaded *Giardia* genome database was carried out. Protein sequences of *Saccharomyces cerevisiae* enzymes involved in all the GPL biosynthesis pathways–PA, CDP-DAG, Kennedy pathways, and Lands cycle, were downloaded from SGD (http://www.yeastgenome.org/), and were used as queries. To exhaustively identify homologous genes/enzymes in *Giardia*, the reviewed bacterial genes/enzymes and many other eukaryotic sequences from Uniprot http://www.uniprot.org/ and published literatures were also used as queries, particularly including the isoenzymes in different organisms such as yeast GEP4, bacterial pgpA, pgpB, and pgpC, all of which are responsible for the dephosphorylation of PGP to PG but do not show homology to each other. The detailed accession numbers of the queries are listed in Supplementary Table S2. Then all the significant hits were used to re-blast against the *Giardia* genome database. All the homologous sequences found by the two rounds of blast (E < 0.001) were collected. 2) The HMM profiles for the function domains of each gene (detailed in the Supplementary Table S3) were queried using hmm search command included in the HMMER (v3.0) software. All the candidate homologs identified through these two methods were further confirmed with Pfam search http://pfam.sanger.ac.uk/ to ensure the presence of functional domain. Only the sequences with the functional domain were used to re-blast against the NCBI non-redundant (nr) database to see whether their top hits are GPL biosynthesis genes/enzymes.

### Transcription or expression examination

To verify whether the identified genes are expressed in *Giardia*, the EST, Mass spec, Microarray and/or Serial Analysis of Gene Expression (SAGE) data in *Giardia*DB (http://www.*Giardia* db.org/ *Giardia* db/) were searched to find whether their corresponding mRNA or proteins are present in these data.

### Phylogenetics

Phylogenetics were carried out mainly based on the guideline described in one article^39^. Briefly, two separate rounds of analysis were performed. In the first round, as many homologous protein sequences as possible were collected by blasting against nr databases in NCBI using the sequences of five eukaryotes representative of the currently accepted five major eukaryotic supergroups^40^ (*Giardia lamblia*, *Toxoplasma gondii*, *Homo sapiens*, *Arabidopsis thaliana*, *Dictyostelium fasciculatum)* as queries and e value was set to 0.001. The gene trees generated by RAxML 7.7.9^41^ displayed gene clades that are most closely related to the in-group sequences. These clades are selected as potential outgroups for the second round of analysis. In the second round, homologous sequences were obtained by selecting representative in-group sequences based on the trees obtained in the first round and place-holder representatives from the most closely related clades identified in the first round as potential out-groups. The root for each gene tree was placed between the most distant of these (selected as the out-group) and the rest.

The obtained homologous sequences were aligned with muscle^42, 43^ and were visually inspected to identify unambiguously aligned regions that are suitable for phylogenetic analysis (the edited multiple sequence alignment files are showed in Supplementary Data S1–S10), then the best amino acid substitution model for each gene was selected using ProtTest 3.2^44, 45^. The detailed obtained best amino acid substitution model for each gene was available in the Supplementary Table S4.

Phylogenies were inferred for each gene by maximum-likelihood (ML) and Bayesian methods using the corresponding best amino acid substitution model defined by ProtTest. The detailed arguments are as follows: 1) RAxML 7.7.9^41^ for rapid bootstrap support (Rbs); 2) MrBayes 3.2.6^36^ was used to perform parallel Bayesian analyses, two Metropolis-coupled Markov Chain Monte Carlo (MCMCMC) runs with four chains each were performed. Burnin fraction was set to 0.25, Convergence of the chains was assessed by monitoring that the standard deviation of split frequencies was <0.01. The 50% majority-rule consensus tree was determined to calculate the posterior probabilities for each node. Each final Gene tree was depicted by combining phylogenies from RaxML and MrBayes using TreeGraph 2^47, 48^.

### Comparative genomic analysis of the phylogenetic distribution of *Giardia*’s absent GPL biosynthesis genes

To explore whether the absence of the GPL biosynthesis genes is a primitive feature of *Giardia* or due to parasitic reduction, the phylogenetic distribution of *Giardia’s* absent genes were investigated and compared among all the Archaea with genome sequencing data, Bacteria (mainly includes several kinds of bacteria which were considered as the bacterial-ancestor-of-eukaryote co-descendants according to several hypotheses about the origin of eukaryotic cells^29–31^), some relatively close protozoans of *Giardia* in Excavata, and some parasitic protozoa living in the similar intestinal environment. Their genome data were downloaded from NCBI, Sanger, JGI, and EuPathDB. Custom databases were constructed from these resources. The identification details of these genes/enzymes are the same as those mentioned above.

### Vector Construction, Expression and Location Observation

The plasmids p*Gi*PSD-GFPa.*pac*, p*Gi*PDI3-DsRed-T2tetRNNL*.neo* and p*Gi*IscU-DsRed-T2tetRNNL*.neo* were reconstructed (Supplementary Table S5). They were transformed into *E. coli* DH5α competent cells, and the positive clones were confirmed by automated sequencing before transfection. The transformation of *Giardia*l trophozoites and the establishment of stable transfectants were done as previously described^49, 50^, but the electroporation conditions were modified (GenePulserXL (Bio-Rad) at 400 V, 1000 μF and 700 Ω).

Stable transfectants were collected and fixed with 1% paraformaldehyde to be prepared for fluorescence microscopy (Leica DM6000B).

### Data Availability

The datasets generated during and/or analysed during the current study are available from the corresponding author on reasonable request.

## Electronic supplementary material

Supplementary file
