# Supplementary material for: Giardia’s primitive GPL biosynthesis pathways with parasitic adaptation ‘patches’: implications for Giardia’s evolutionary history and for finding targets against Giardiasis
Source: Sci Rep. 2017 Aug 25;7:9507. doi: 10.1038/s41598-017-10054-1 (PMC5573378; doi:10.1038/s41598-017-10054-1)
Supplement: Supplementary file 1 — Supplementary file [file 41598_2017_10054_MOESM1_ESM.pdf]

Giardia's primitive GPL biosynthesis pathways with parasitic adaptation 'patches': implications for *Giardia*'s evolutionary history and for finding targets against *Giardia*sis

Qingqing Ye[†, 1,2], Haifeng Tian[†,1], Bing Chen[1], Jingru Shao[1], Yan Qin[1,3], and Jianfan Wen[*,1]

[1] State Key Laboratory of Genetic Resources and Evolution, Kunming Institute of Zoology, Chinese Academy of Sciences, Kunming, Yunnan 650223, China;

[2] Kunming College of Life Science, University of Chinese Academy of Sciences, Kunming, Yunnan 650204, China；

[3] Kunming Institute of Botany, Chinese Academy of Sciences, Kunming, Yunnan 650201, China

[†]These authors contributed equally to this work.

***Corresponding author:** Jianfan Wen, E-mail: wenjf@mail.kiz.ac.cn

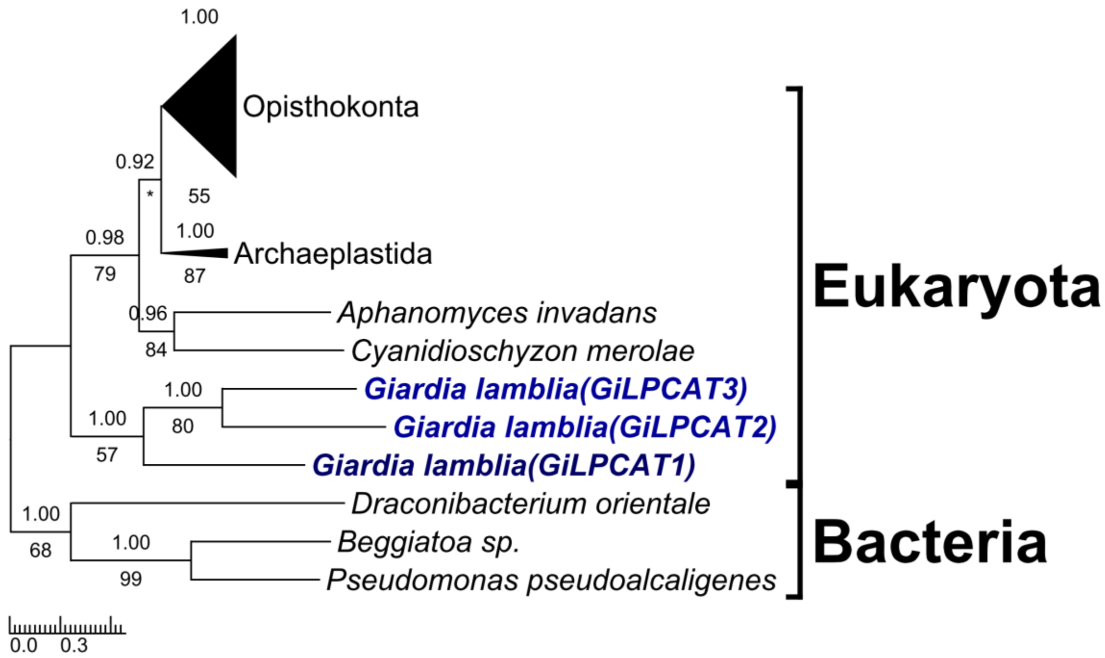

Supplementary Figure S1. The rooted phylogenetic tree of the 98 obtained homologous sequences to LPCATs The tree is illustrated using the same conventions as in Figure 2. The 122 conserved sites in the alignment were used for the tree construction. Both RaxML and Mrbayes results show that *Giardia* (in bold and blue) is at the basal position of eukaryotes.

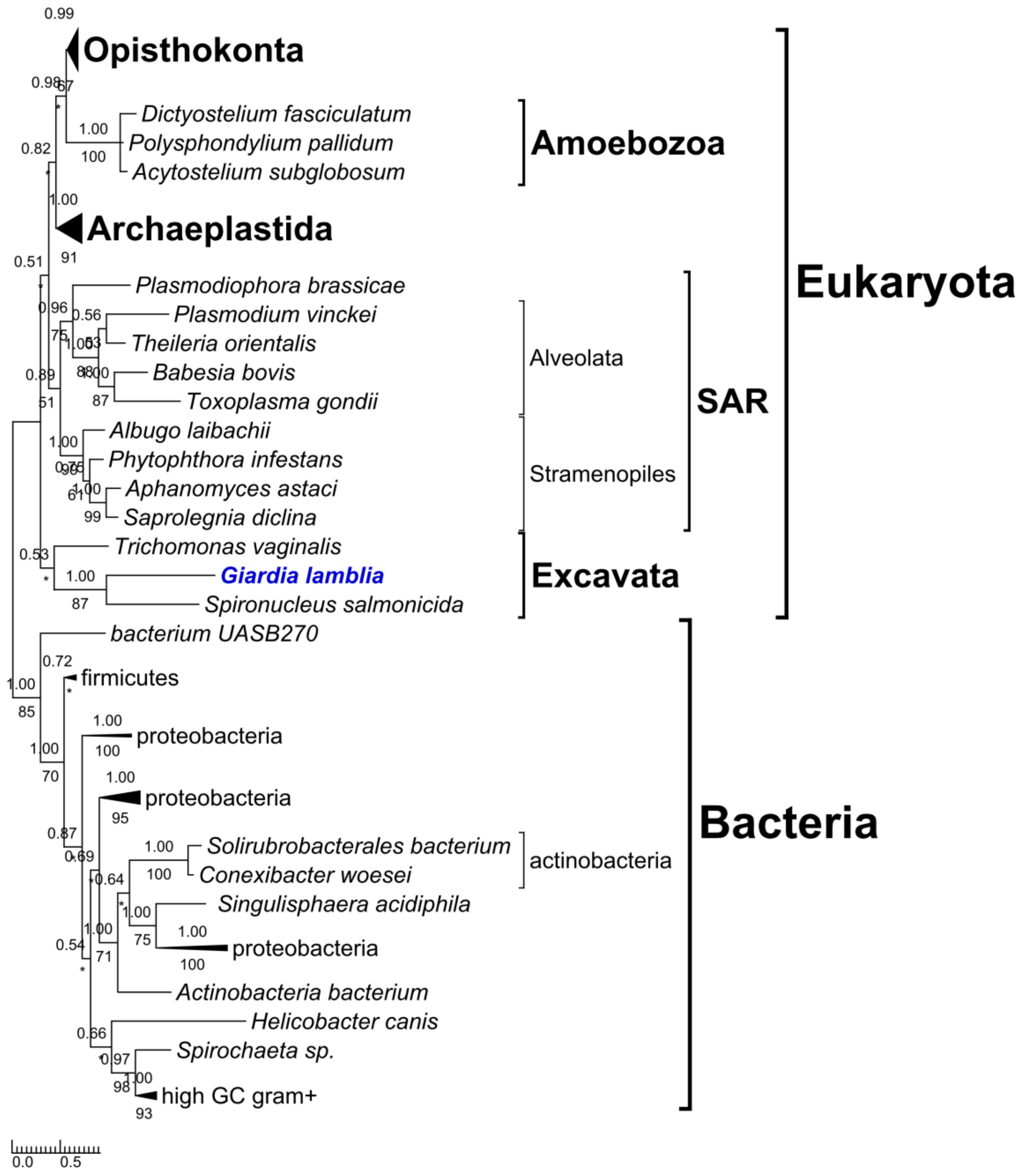

Supplementary Figure S2. The rooted phylogenetic tree of the 112 obtained homologous sequences to Ek/Ck. The tree is illustrated using the same conventions as in Figure 2. The 168 conserved sites in the alignment were used for the tree construction. Both RaxML and Mrbayes results show that *Giardia* (in bold and blue) is at the basal position of eukaryotes.

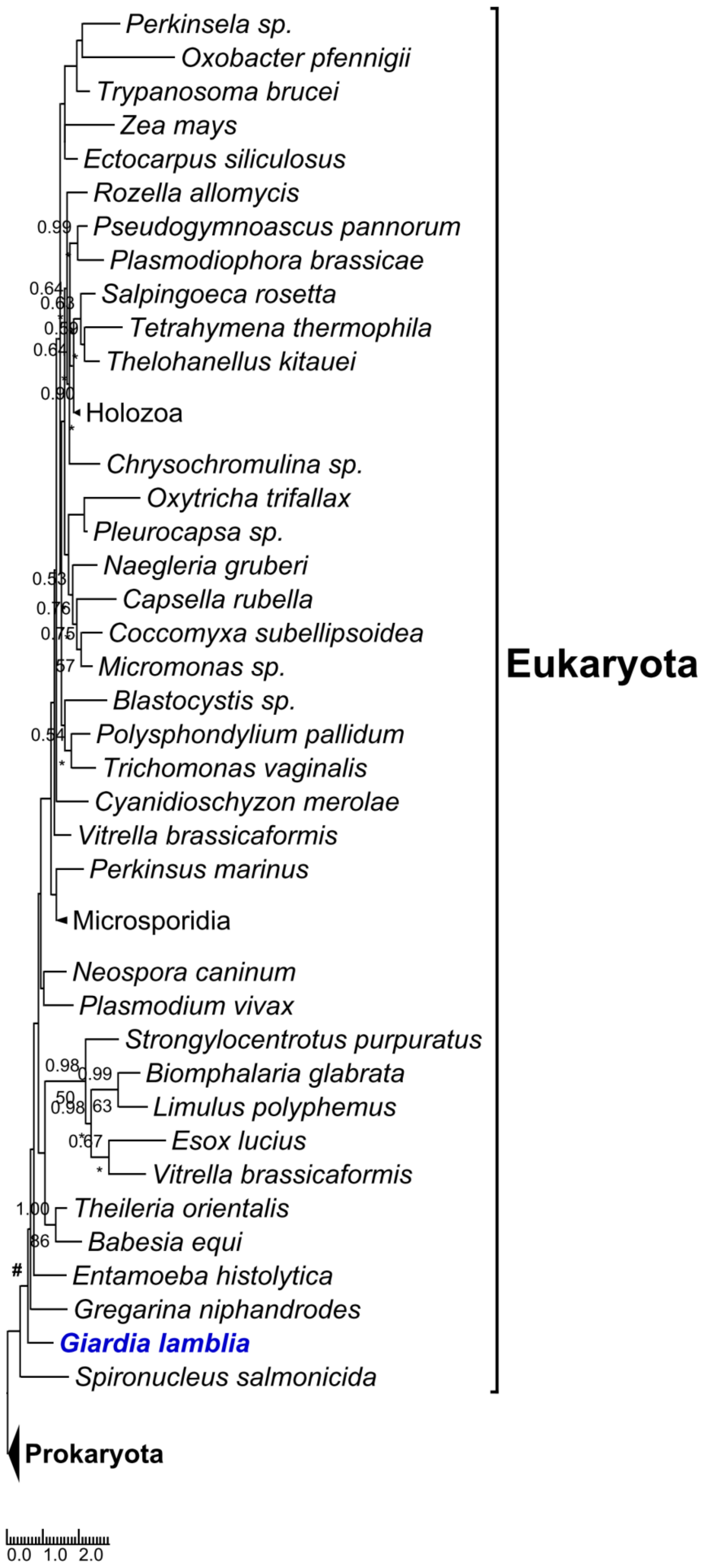
Perkinsela sp.
Oxobacter pfennigii
Trypanosoma brucei
Zea mays
Ectocarpus siliculosus
Rozella allomycis
Pseudogymnoascus pannorum
Plasmodiophora brassicae
Salpingoeca rosetta
Tetrahymena thermophila
Thelohanellus kitauei
Holozoa
Chrysochromulina sp.
Oxytricha trifallax
Pleurocapsa sp.
Naegleria gruberi
Capsella rubella
Coccomyxa subellipsoidea
Micromonas sp.
Blastocystis sp.
Polysphondylium pallidum
Trichomonas vaginalis
Cyanidioschyzon merolae
Vitrella brassicaformis
Perkinsus marinus
Microsporidia
Neospora caninum
Plasmodium vivax
Strongylocentrotus purpuratus
Biomphalaria glabrata
Limulus polyphemus
Esox lucius
Vitrella brassicaformis
Theileria orientalis
Babesia equi
Entamoeba histolytica
Gregarina niphandrodes
Giardia lamblia
Spironucleus salmonicida
Eukaryota
Prokaryota
0.0 1.0 2.0

Supplementary Figure S3. The rooted phylogenetic tree of the 85 obtained homologous sequences to Pis. The tree is illustrated using the same conventions as in Figure 2. The 91 conserved sites in the alignment were used for the tree construction. The symbol "#" above the branch indicates conflict against *Giardia*'s early branching in the Mrbayes tree. The RAxML tree shows that *Giardia* (in bold and blue) is at the basal position of eukaryotes.

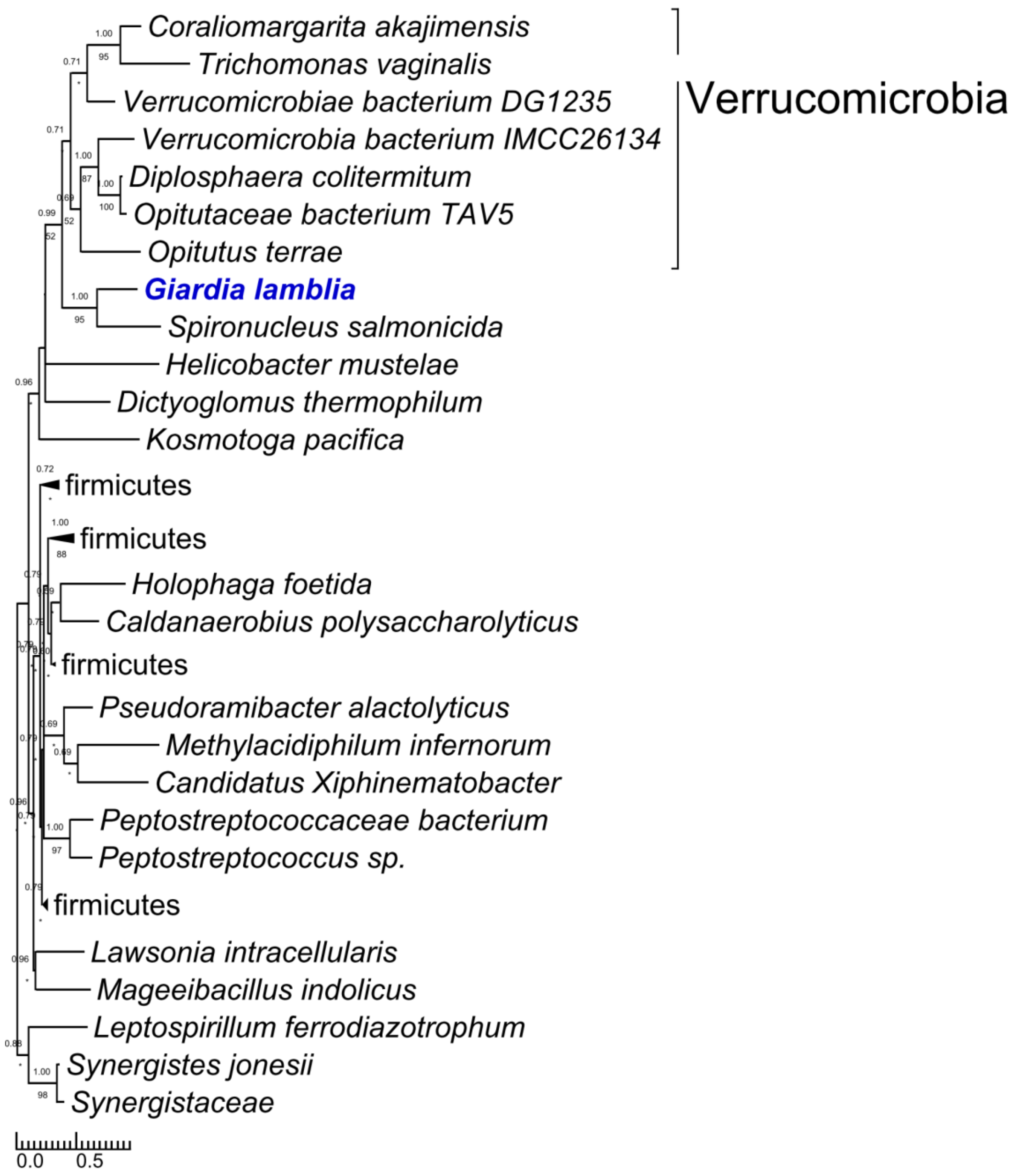

Supplementary Figure S4. The rooted phylogenetic tree of the 47 obtained homologous sequences to Pgps The tree is illustrated using the same conventions as in Figure 2. The 122 conserved sites in the alignment were used for the tree

construction. Both RAxML and Mrbayes results show *Giardia* (in bold and blue) (together with its close relative *Spironucleus* and another parasitic protozoan, *Trichomonas*) form a cluster with the homologs of Verrucomicrobia, suggesting *Giardia* (and the two other parasitic protozoans) most probably once horizontally transferred the gene from Verrucomicrobia.

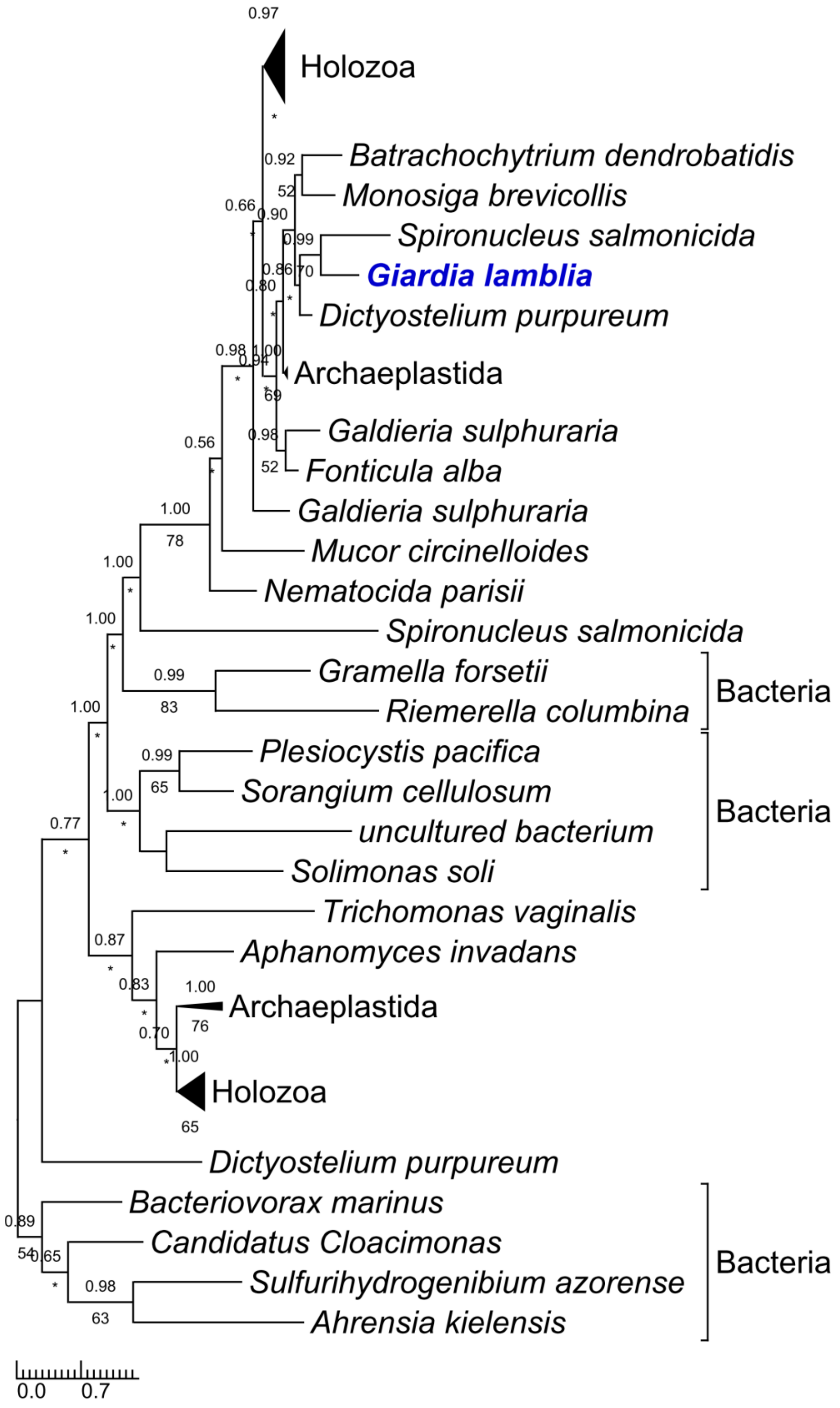

Supplementary Figure S5. The rooted phylogenetic tree of the 101 obtained homologous sequences to Slc1 The tree is illustrated using the same conventions as in Figure 2. The 126 conserved sites in the alignment were used for the tree construction. Both RAxML and Mrbayes results do not show that *Giardia* (in bold

and blue) is at the basal position of eukaryotes.

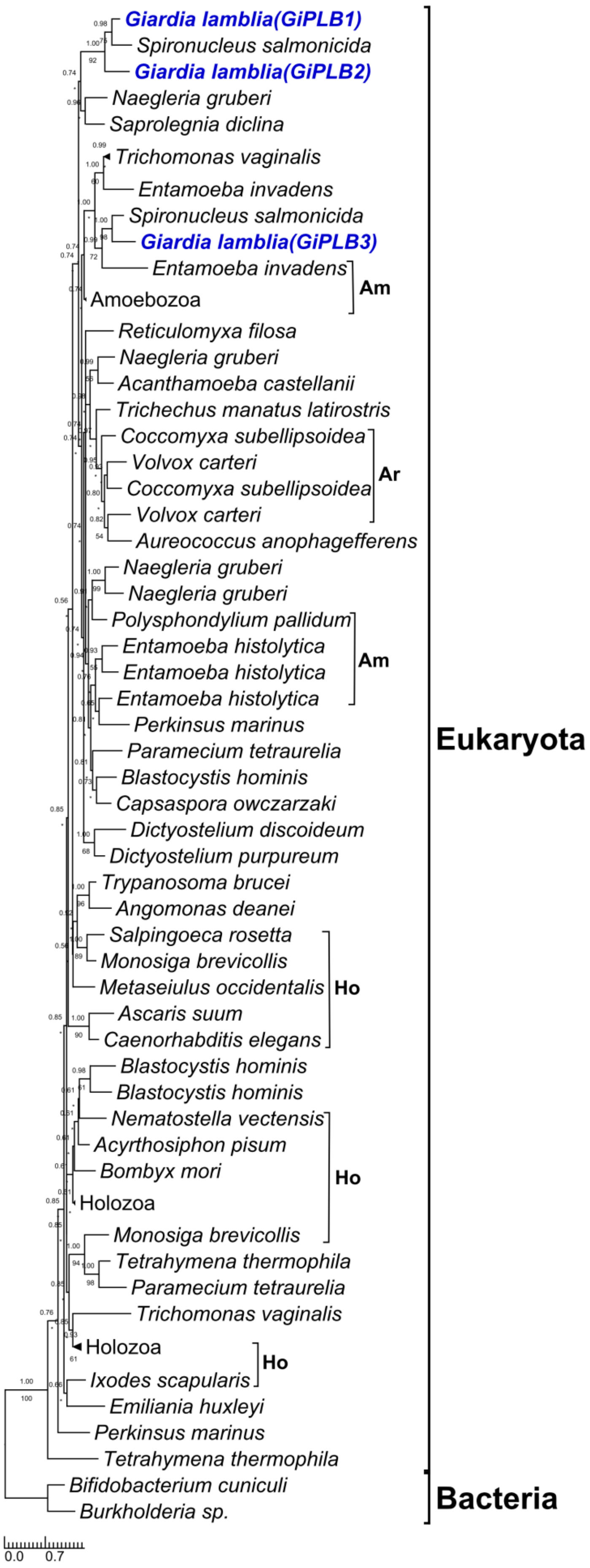

Giardia lamblia(GiPLB1)
Spironucleus salmonicida
Giardia lamblia(GiPLB2)
Naegleria gruberi
Saprolegnia diclina
Trichomonas vaginalis
Entamoeba invadens
Spironucleus salmonicida
Giardia lamblia(GiPLB3)
Entamoeba invadens
Amoebozoa
Am
Reticulomyxa filosa
Naegleria gruberi
Acanthamoeba castellanii
Trichechus manatus latirostris
Coccomyxa subellipsoidea
Volvox carteri
Coccomyxa subellipsoidea
Volvox carteri
Ar
Aureococcus anophagefferens
Naegleria gruberi
Naegleria gruberi
Polysphondylium pallidum
Entamoeba histolytica
Entamoeba histolytica
Entamoeba histolytica
Am
Perkinsus marinus
Paramecium tetraurelia
Blastocystis hominis
Capsaspora owczarzaki
Dictyostelium discoideum
Dictyostelium purpureum
Trypanosoma brucei
Angomonas deanei
Salpingoeca rosetta
Monosiga brevicollis
Metaseiulus occidentalis
Ho
Ascaris suum
Caenorhabditis elegans
Blastocystis hominis
Blastocystis hominis
Nematostella vectensis
Acyrthosiphon pisum
Bombyx mori
Holozoa
Ho
Monosiga brevicollis
Tetrahymena thermophila
Paramecium tetraurelia
Trichomonas vaginalis
Holozoa
Ixodes scapularis
Ho
Emiliania huxleyi
Perkinsus marinus
Tetrahymena thermophila
Bifidobacterium cuniculi
Burkholderia sp.
Eukaryota
Bacteria
0.0
0.7

Supplementary Figure S6. The rooted phylogenetic tree of the 74 obtained homologous sequences to PLB. The tree is illustrated using the same conventions as in Figure 2. The 144 conserved sites in the alignment were used for the tree construction. "Am" is short for "Amoebozoa", "Ar" is short for "Archaeplastida", "Ho" is short for "Holozoa". The phylogeny of PLB is very complicated with many disagreements with the common phylogenetic relationships (e.g. the split of Holozoa on the tree), which is probably due to the complicated evolutionary history of this enzyme or other unknown reasons.

Supplementary Table S1. The phylogenetic distribution of Cls_cap among representative eukaryotic organisms

	Organism	Cls_cap
Opisthokonta	*Homo sapiens*	+
	Xenopus laevis	+
	Drosophila melanogaster	+
	Caenorhabditis elegans	+
	Monosiga brevicollis	+
	Saccharomyces cerevisiae	+
	Aspergillus fumigatus	+
	Encephalitozoon cuniculi	-
Amoebozoa	*Entamoeba histolytica*	-
	Entamoeba dispar	-
	Acanthamoeba castellanii	+
Archaeplastida	*Arabidopsis thaliana*	+
	Oryza sativa	+
	Chlamydomonas reinhardtii	+
	Ostreococcus lucimarinus	+
	Cyanidioschyzon merolae	+
SAR	*Tetrahymena thermophila*	-
	Plasmodium faciparum	-
	Phytophthora sojae	+
	Pythium ultimum	+
	Saprolegnia parasitica	+
	Thalassiosira pseudonana	+
	Ectocarpus siliculosus	+
Excavata	*Leishmania braziliensis*	-
	Leishmania infantum	-
	Trypanosoma cruzi	-
	Bod saltans	-
	Naegleria gruberi	-
	Trichomonas vaginalis	-
	Giardia lamblia	-

Supplementary Table S2. The accession numbers of the queries used in the blast similarity search for each gene

Gpd1/Gpd2(NAD+): Glycerol-3-phosphate dehydrogenase

Saccharomyces Genome Database accession numbers: S000002180, S000005420

UniprotKB accession numbers: A0LLR7, A0Q4Y5, A0QLB8, A1BJJ1, A5CE97, A5CVT6, A5GTA8, A7MX60, A9BHX5, A9NGK6, B0BAG9, B0BXL6, B0SAV3, B1MZX5, B2I5B4, B2ID14, B2V656, B3DTU0, B3WCP6, B5YEC5, B7GZW3, B7J4Y4, B8GR94, B9KD88, C0QYQ8, C1F6U2, C1KWN6, C6BYZ0, C6E7R1, O22216, O26468, O29390, O57656, O67555, O83973, P13706, P21695, P21696, P41911, P58142, P61741, P61745, P61748, P61749, P73033, Q00055, Q1DCL2, Q1G8H5, Q1IPR2, Q1MQ45, Q1QBS6, Q21IX1, Q24VA4, Q2GEH4, Q2IMY8, Q2JPE4, Q2L0B2, Q2RND0, Q2S2H6, Q2W015, Q31E81, Q3AP01, Q3JF19, Q3K8F9, Q3ZYV3, Q4QHG4, Q5F5A8, Q5FPS8, Q5HAM0, Q5LLR6, Q5NL81, Q5PA02, Q5YRY6, Q5ZT56, Q65X70, Q67NS7, Q6AFK3, Q6AQJ3, Q6F1R6, Q6KHG2, Q6UGN0, Q82SU6, Q83G27, Q895X7, Q8D216, Q8EWH5, Q8EZB6, Q8H2J9, Q8N335, Q935Z2, Q949Q0, Q9CBR9, Q9R9L6, Q9RR76, Q9SCX9, Q9Z751, Q9ZKP0

Sct1/Gpt2: Glycerol 3-phosphate/dihydroxyacetone phosphate acyltransferase

Saccharomyces Genome Database accession numbers: S000001775, S000000107

UniprotKB accession numbers: A4IF87, O15228, P98192, Q9ES71, P32784, P36148

Ayr1: NADPH-dependent 1-acyl dihydroxyacetone phosphate reductase

Saccharomyces Genome Database accession number: S000001386

UniprotKB accession numbers: P40471, Q09851

Slc1: 1-acyl-sn-glycerol-3-phosphate acyltransferase

Saccharomyces Genome Database accession number: S000002210

UniprotKB accession numbers: Q9NUQ2, P33333, Q9D1E8

Cds: Phosphatidate cytidylyltransferase

Saccharomyces Genome Database accession number: S000000233

UniprotKB accession numbers: P56079, Q94A03, Q9M001, P38221, Q92903, P98191, O35052, O95674, O04928, P53230, Q99L43, Q9P381, P9WPF7, Q1PE48, P0ABG1, O49639, O04940, Q91XU8, A0JNC1, P44937, Q55D90, Q59640, O67292, Q9X1B7, O31752, P73548, Q9ZML7, P75160, Q1RII7, Q9ZDA8, Q99UL1, Q4L5W3, Q8G0E0, Q6GHH4, Q5HPT0, Q2YRP9, P53439, P9WPF6, Q8YHH2, Q95ZE3, Q9Z7Y6, Q4ULR7, P63759, Q9CBU1, Q5HGH0, Q7A121, Q49X46, P0C102, Q9PJU1, Q68WV5, Q7A5Y4, Q8CST9, O84457, P0ABG2, O25004, Q49433, Q92I31, P0ABG3, Q6G9V2, O74339, Q3TUH1, Q61X59, Q6DJM2, Q550P4, Q3B7H2, Q96BW9, Q32L81, Q8INF2, Q9N4G7

Pgs_pld: CDP-diacylglycerol--glycerol-3-phosphate 3-phosphatidyltransferase (belonging to the phospholipase D family)

Saccharomyces Genome Database accession number: S000000510

UniprotKB accession numbers: Q2KJ28, Q32NB8, Q9Z2Z7, P79001, Q7KWX2, Q5R8K7, Q9HDW1, Q8BHF7, Q5ZHN9, P25578

Pgps_cap: CDP-diacylglycerol-glycerol-3-phosphate 3-phosphatidyltransferase (belonging to the CDP-alcohol phosphatidyltransferase family)

UniprotKB accession numbers: O67908, P46322, A1AC64, P0ABG0, Q0TGS6, P0ABF9, P44528, P0ABF8, P47360, P75520, Q6D364, Q7N5C8, Q1RAM8, Q492P7, P45419, Q92JJ2, Q9ZE96, Q7VR12, Q3IYX7, Q57N57, Q5PI18, Q68XS5, Q8XFD0, Q4UN77, Q322L9, Q7CQB9, Q1RKM3,

Q32HD9, Q83R47, Q0T3L5, Q2NTS8, P63755, P63756, Q6GHF2, Q5HGE8, P63757, Q5HPQ8, Q3Z2T6, Q8CPF7, Q6G9T0, A1JLU9, Q8D2N9, Q1CHE5, O66076, Q8ZF51, A4TK40, Q66BN4, Q1C8L0
**Ptpmt1：Phosphatidylglycerophosphatase and protein-tyrosine phosphatase 1**<br>UniprotKB accession numbers: Q66GT5, Q8WUK0, Q8WUK0-2, Q8WUK0-3, P0C089
**PgpA: Phosphatidylglycerophosphatase A**<br>UniprotKB accession numbers: P18200, P44157
**PgpB: Phosphatidylglycerophosphatase B**<br>UniprotKB accession numbers: 0A924, P0A925, P0A926, P44570
**PgpC: Phosphatidylglycerophosphatase C**<br>UniprotKB accession numbers: P0AD42, P0AD43
**Gep4: Phosphatidylglycerophosphatase GEP4**<br>UniprotKB accession numbers: P38812, Q9Y7U3
**Cls_cap: Cardiolipin synthase (belonging to the CDP-alcohol phosphatidyltransferase family)**<br>Saccharomyces Genome Database accession number: S000002301<br>UniprotKB accession numbers: Q5U2V5, Q80ZM8, Q8H8U0, Q8MZC4, Q93YW7, Q9UJA2
**Cls_pld: Cardiolipin synthase (belonging to the phospholipase D family)**<br>UniprotKB accession numbers: P0A6H8, P45860, P71040, P0AA84, P75919, Q81V75, Q81TR2, Q5HMD3, Q8XCC6, Q87TY7, P63799, Q83RM8, Q8ZEI2, Q1CJ46, P45865, P0AA85, Q8Z883, P59715, Q9K8Z4, O66043, Q0TQG9, Q927Z0, Q8Y4E3, Q81I00, Q5HGA3, Q6GH88, Q5HPM5, Q8CPD8, Q81GK4, Q5HEB2, Q6GEY7, Q8CNK3, B8D966, P57361, Q8K9P9, B8D7H0, Q89AL5, A8AG50, A7MNA4, A7ZL20, B7UQD9, B7ML01, B7LHJ4, B5YYF4, B7NVL8, B7MU36, B7LY04, C4ZTU1, B1XAT9, A7ZZI5, A1AAH3, Q0TIC2, P0A6H9, B1ITK7, B7N460, B6I9W2, B1LH51, Q1RCI7, C5BDA3, A4WB84, B2VKV4, B7LS29, B5XQA9, A6TAJ7, Q6D4S2, C6DGY3, B4EWI7, B7V5T6, Q02DW8, Q9HTH0, Q1I2L0, Q4K3D9, C3K1C4, A5WB80, B0KR91, Q88C19, P31048, B1JFN5, Q4ZL66, B5F4L0, A9MPD5, Q57NS0, B5FU52, B5R3N1, B5R6L2, B4TJM2, B4SUC9, Q5PCS9, A9MWP9, C0Q393, B5BIA8, B4TX53, P63798, A8GFA0, B2TZZ4, Q31ZU1, Q32GV1, Q3Z0W3, Q2NT72, Q8D2I8, A1JQ40, A7FI50, Q1C7R0, B2K3U5, A9R8K8, A4TJ85, Q66AL9, B1JKT9, Q8FJN9, Q8ZQP4, P0C2E2, Q0ST20, Q5L1S5, C5D794, A4IL76, C1KYS0, Q71WS5, B8DBK8, A0ALI7, Q8EM16, P63800, P63801, Q6G7M2, P63802
**Pis: CDP-diacylglycerol-inositol 3-phosphatidyltransferase**<br>Saccharomyces Genome Database accession number: S000006317<br>UniprotKB accession numbers: O14735, P70500, Q8LBA6, P06197, Q8VDP6, P9WPG7, Q8GUK6, Q10153, P9WPG6, Q9F7Y9
**Psse/Psse2: Phosphatidylserine synthases 1/2 (involved in base-exchange reactions)**<br>UniprotKB accession numbers：Q803C9, Q5PQL5, B2GV22, Q00576, Q99LH2, E7EY42, Q08D11, Q5ZM65, P48651, O08888, Q9BVG9, Q2KHY9, B1H3H9, E1BYA3, Q9Z1X2, F4HXY6
**Pss: CDP-diacylglycerol--serine O-phosphatidyltransferase (belonging to the CDP-alcohol phosphatidyltransferase family)**<br>Saccharomyces Genome Database accession number: S000000828<br>UniprotKB accession numbers： Q48269, P39823, P9WPG1, Q58609, P08456, O94584, Q95ZE2
**Psd: Phosphatidylserine decarboxylase**<br>Saccharomyces Genome Database accession numbers: S000005113, S000003402<br>UniprotKB accession numbers: P39006, Q5ABC5, Q84V22, O14333, C4QX80, F6N7K6, Q10949,

Q10T43, Q97N08, P53037, Q5AK66, Q9UTB5, F4KAK5, Q5JN42, C4R360, Q97KW7

Pem1/Pem2: Phosphatidylethanolamine N-methyltransferase/ Phosphatidyl-N-methylethanolamine N-methyltransferase

Saccharomyces Genome Database accession numbers: S000003389, S000003834

UniprotKB accession number: P05375, Q9SAH5, O74827, Q7S5W9, C8VRV0, C0NLX2, A1C7T5, A2R616, A6S950, C5M4D4, A7TNI7, C5GN10, C4YL78, Q2H0U8, C5PEI5, B0D4E6, A5DS78, C7Z7C3, C1G565, A5DL79, B2WFD4, B6QG32, Q2UDE5, Q5BBC6, A4RHN5, Q7SAJ6, B6HJA3, B8MBN3, Q6C6U9, C8Z951, C5DZU3, C5FZ62, B8N6H2, Q0CVD7, C4Y206, Q6CJI9, A8PRN6, A1DIF7, C1GZK1, C0RZV6, B2B2N5, O74787, C4JDF8, C7GQ65, B5VJA0, Q4WZS1, B9WL59, C6H4B5, Q754G0, Q59LV5, A7TLA7, D4AT37, B0XUW3, C5JCV0, Q6FVB6, Q6BY28, C4QXE9, C5DGB6, Q0U2R3, A3LQW6, B6JWP7, D1ZIW5, D4DGR3, B3LI73, A6ZUG8, P05374, Q54H80, Q08388, Q54SD5, Q9UBM1, Q7YRH6, Q61907

Pemt: Phospholipid N-methyltransferase

NCBI accession numbers (obtained from the published literatures): AAA26152.1, BAA34057.1, NP_773634.1, NP_773274.1, AAG10237.1, CAB91878.1, NP_774806.1, NP_771444.1

PLB: Phospholipase B

Saccharomyces Genome Database accession numbers: S000004608, S000004610, S000005371

UniprotKB accession numbers: O54728, Q6P1J6, Q3TTY0, O70320, Q06HQ7, Q05017, Q9UWF6, Q03674, P39105, P78854, Q08108, Q9P8P2, B0Y665, B0XZV8, P0C957, O59863, P0CP75, P0C958, P0CP74, Q8TG07, Q9UVX1, Q8TG06, O93795, Q9P8P4, B0Y1M7, Q55FN1, Q55BJ6, Q9GL30, Q5U2V4, Q6P4A8, Q8VCI0, Q54M94, Q3TCN2, Q4QQW8, F8S101, Q550U9, F8J2D3, Q54PS7, Q54ZI6, Q554H5, Q9XWV2, O62146, Q8NHP8, Q9BL07, Q2KIY5, Q8NHP8-2, Q3TCN2-2, Q9GL30-2

LPCAT: Lysophosphatidylcholine acyltransferase

Saccharomyces Genome Database accession number: S000006344

UniprotKB accession numbers: Q0KHU5, Q1LWG4, Q502J0, Q8NF37, P0C1Q3, Q9D5U0, Q8BYI6

LCAT: Lecithin-cholesterol acyltransferase

UniprotKB accession numbers: Q71N54, Q9FZI8

Ek/Ck: Choline/ethanolamine kinase

Saccharomyces Genome Database accession numbers: S000004123, S000002554

UniprotKB accession numbers: P35790, O54804, Q01134, Q9Y259, O55229, O54783, P54352, Q9HBU6, Q9D4V0, Q03764, Q9NVF9, A7MCT6, D3ZRW8, Q869T9, Q554D8, A7SK27, O54804, Q01134, Q9Y259, O55229, O54783, P46558, P46559, P46560, Q10276, P20485

Ect/Pct: Ethanolamine-phosphate cytidylyltransferase/Choline-phosphate cytidylyltransferase

Saccharomyces Genome Database accession numbers: S000003434, S000003239

UniprotKB accession numbers: P49584, P49585, P49586, P19836, Q9Y5K3, Q811Q9, Q9QZC4, P49583, P49587, O74975, P13259, Q8SQW6, Q9UTI6, P33412, Q5EA75, Q55BZ4, Q99447, Q922E4, O88637

Lic: CTP phosphocholine cytidylyltransferase

NCBI accession numbers (obtained from the published literatures): AAK94072.1

Ept/Cpt: Choline/ethanolaminephosphotransferase

Saccharomyces Genome Database accession numbers: S000001165, S000005074

UniprotKB accession numbers: P17898, P22140, Q9Y6K0, Q9C0D9, Q80TA1, Q8BGS7, Q6AXM5,

Q5ZKD1, Q17QM4, Q28H54, Q7ZYQ3, Q5NV96
**Pcs: Phosphatidylcholine synthase**<br>UniprotKB accession numbers: A9CIM3, Q9KJY8, Q89LF9, O51265, D0B707, Q5ZV56, Q9HXE9, Q1MGQ9, Q98MN3, D5KX81<br>NCBI accession numbers (obtained from the published literatures): AAF27310.1, Q9HXE9.1, AAK87563.2, AAL53937.1, BAB48080.1, WP_028178769.1, WP_015921054.1

Supplementary Table S3. The HMM profiles for the function domains of each gene used in the hmmsearch

Gene	Pfam domain
Gpd1/Gpd2(NAD+)	PF01210.22(NAD_Gly3P_dh_N) PF07479.13(NAD_Gly3P_dh_C)
Sct1/Gpt2	PF01553.20 (Acyltransferase)
Ayr1	PF00106.24(adh_short)
Slc1	PF01553.20(Acyltransferase)
Cds	PF01148.19(CTP_transf_1)
Pgs_pld	PF00614.21(PLDc) PF13091.5(PLDc_2)
Pgps_cap	PF01066.20(CDP-OH_P_transf)
Ptpmt1	PF00782.19(DSPc)
PgpA	PF04608.12(PgpA)
PgpB	PF01569.20(PAP2)
PgpC	PF12710.6(HAD)
Gep4	PF09419.5 (PGP_phosphatase)
Cls_cap	PF01066.20(CDP-OH_P_transf)
Cls_pld	PF13396.5(PLDc_N) PF13091.5(PLDc_2)
Pis	PF01066.20(CDP-OH_P_transf)
Psse1/Psse2	PF03034.10 (PSS)
Pss	PF01066.20(CDP-OH_P_transf)
Psd	PF02666.14(PS_Dcarbxylase)
Pem1/Pem2	PF04191.12(PEMT)
Pemt	PF08241.7(Methyltransf_11) PF00398.15(RrnaAD)
PLB	PF00657.21(Lipase_GDSL)<br>PF01735.17(PLA2_B)<br>PF04916.12(Phospholip_B)
LPCAT	PF01553.20(Acyltransferase)
LCAT	PF02450.14(LCAT)
Ek/Ck	PF01633.19 (Choline_kinase)
Ect/Pct	PF01467.25 (CTP_transf_like)
LicC	PF12804.6(NTP_transf_3)
Ept/Cpt	PF01066.20(CDP-OH_P_transf)

Pcs	PF01066.20(CDP-OH_P_transf)

Supplementary Table S4. The detailed information for the phylogenetic reconstruction of each gene tree.

Gene	Sequence number	Sites number	Evolutionary Model
Cds	127	211	VT+I+G+F
LPCATs	98	122	LG+I+G
Ek/Ck	112	168	LG+I+G
LCATs	94	99	LG+G
Pis	85	91	LG+I+G+F
Pss	70	131	LG+I+G+F
Pgps	47	122	LG+G
Psd	95	148	LG+I+G
Slc1	101	126	LG+G
PLB	74	144	LG+G

Supplementary Table S5. Primer sequences used for amplifying β subunit of *Gi*Psd, and open reading frames of *Gi*Pdi3 and *Gi*IscU, respectively, from their corresponding cDNA. Restriction enzyme recognition sites are shown as italics and underlined.

Gene	Primer	Antisense primer
*Gi*Psd-β	AGAAGA*CCATGG*AGTAATTTTTAATGCGCATCGTTGATCTCA	*CCATGG*TCCGAAGTGGAATTGACC
*Gi*IscU	AGAAGA*CCATGG*ATAAATTCTAATTTAATGACAAGCCTTCAGC	TCTTCT*TCATGA*TAGAAGACTTTGATACCTGTATCTTGCTTCCCC
*Gi*Pdi3	AGAAGA*CCATGG*AGTAATTTTTAATGATTGCCGGCCTCCTCC	TCTTCT*TCATGA*TCAGCCTCTGCTTGATCCACTCCTTCATTGCA

Supplementary Table S6. The distribution of *Giardia*'s absent enzymes in putative bacterial-ancestor-of-eukaryote co-descendants

Bacteria*	Gpd1/Gpd2(NAD^+)	Sct1/Gpt2	Pgs_pld	Cls_cap	Cls_pld	Pem1/Pem2	Psse1/Psse2	Ect/Pct	Ept/Cpt
Spirochaetes [1]	+	+	-	-	+	-/-	-	-	-
Delta-proteobacteria [2, 3]	+	+	-	+	+	-/-	-	-	-
Gamma–proteobacteria [4]	+	+	-	+	+	-/-	-	-	-
Alpha-proteobacteria [5,6]	+	+	-	+	+	-/-	-	-	-

Bacterium [7]	+	+	-	+	+	-/-	-	-	+

The symbol “*” indicates the putative bacterial-ancestor-of-eukaryote co-descendants, which are according to various references in square brackets [ ]. These references are as follows:

[1] Margulis et al. (2006). *PNAS* 103: 13080.

[2] Moreira & Lopez-Garcia (1998). *J Mol Evol* 47: 517.

[3] Lopez-Garcia & Moreira (1999). *Trends Biochem Sci* 24:88.

[4] Horiike et al. (2004). *J Mol Evol* 59: 60.

[5] Martin & Muller (1998) *Nature* 392:37.

[6] Rochette et al., (2014). *MBE* 31:832.

[7] Lake JA (1988). *Nature* 331: 184.

Supplementary Data S1. The edited multiple sequence alignment for the phylogenetic tree reconstruction of Cds

>159113365_Giardia_lamblia_ATCC_50803-Phosphatidate_cytidylyltransferase_
LRVVCMFGML-FLYTLLLFCEILTCPSYLNMDLPFVVGLYLNKYSFVISTFFLLVLLLHVFLVLCGLFLTCLEIIMTVQKGM-YFYLPSLVVNDTFAYILGKLLGRHSLVRVSPSKTWEGYIGVTLVFHCAL
IEFRKGV---SLSMICMLLLWISIVGPVGGMLASLAKRCAKIKNFIPGHGGVDRIDCHILA-IAFYDFF----------
>340370254_Amphimedon_queenslandica-PREDICTED__phosphatidate_cytidylyltransferase__photoreceptor_specific_like_
WRVGIMFIFIMLLLLSFT--EIIILPYYF--FLYIGLLAYHLTFFLYVFGFVMFVLTLQKYGQFFILFFMGSDMVMQNIYGLFWFLIPVLVINDIMAYVFGFFFGRTKLINLSPKKTWEGFLGFTILFLLFI
LAYACLFF-MILYVIHLMLIFASLIAPFGGFFASGFKRAFKIKDFIPGHGGLDRMDCQILMAFVYFTFCR-LISLVFLL
>829100842_Tetrahymena_thermophila_SB210-phosphatidate_cytidylyltransferase_
ARILIMFIIVAFCLLITA--EIILIPFFFFYFYGYTIILYHFAFSLWIIGFLVFVLSLTQYRQFFLILIVVAQVIVSNLYGLVWFILPALVINDIFAYIFGKLFGKTKLIQLSPNKTWEGFIGFSLFFVLFI
FLCQCFYNTFILSFVHLYLLFASLIAPFGGFFASGFKRALKVKDFIPGHGGLDRMDCQLLMTFCYNYIVVINFSLKKEN
>813150221_Saprolegnia_parasitica_CBS_223_65-phosphatidate_cytidylyltransferase_
FRIFSLYSIIGLVLLVLLTMELVVLPFFGVYNYGLRSYSYHWVFSLYALLFVFSVLSLKKYKQMLVLCLIVFQYVLDNIFGLFWFFFPSLVVNDCFAYFCGKLFGKKQFLKLSPNKTWEGFIGFTLIFFFSW
LMCPCVFIWTVLFVLHIIFSFTSVISPFGGFYASAIKRAYKLKDFLPGHGGVDRMDCQFITLFVYSTFIVISVRLILLV
>145546113_Paramecium_tetraurelia_strain_d4_2-hypothetical_protein__macronuclear__
FRIIVMFMVLAIIGLVNI--EILLIKSFGFFCYGLQLILYHIVFMLWVAGFLMFTLSLKKYRQFFILILVVAQVMIMNIFGIVWFILPCLVINDIFAYIFGVSFGKTPLIELSPKKTWEGFIGFTLVAFCFF
LLCPCVFF-FVFSFIHMVFLFTSLVSPFGGFFASGFKRGIKIKDFIPGHGGIDRMDCQILTMFLYHQIVLVYVQLLFLL
>294877856_Perkinsus_marinus_ATCC_50983-Phosphatidate_cytidylyltransferase__putative_
LRFVVMFFIVMFCLLVAA--EICLLPFFFFYGYKMSFIVYHIIYTLLVMGLVAFVLTLRKLRQFLVLLTVVVQAMTANVYGLIWFFLSTVVINDIFAYLFGSFFGYHPLIKLSPKKTWEGFIGITMIFMIWV
LFCKCMYI--LLTMVHLVLSFASLVAPFGGFLASGFKRAFKIKDFIPGHGGLDRFDCEVVMMFVYSNFVALRVQLLFIL
>145551765_Paramecium_tetraurelia_strain_d4_2-hypothetical_protein__macronuclear__
FRIIVMFMVLAIIGLVNI--EILLVKSFGFFCYGFQLILYHIIFMLWVAGFLMFTLSLKKYRQFFILILVVAQVMIMNIFGIVWFILPCLVINDIFAYIFGVSFGRTPLIELSPKKTWEGFIGFTLVAFCFF
LLCPCVFF-FIYSFLHMVFLFTSLVSPFGGFFASGFKRGIKIKDFIPGHGGIDRMDCQILTMFLYHQIVLVYVQLLFLL
>294936211_Perkinsus_marinus_ATCC_50983-phosphatidate_cytidylyltransferase__putative_
SRLVLG---IA---------EICLLPFFFFYGYKMTFIVYHIIYTLFVIGLVAFVLSLRKLRQFLVLIIVVVQAMTANIYGLIWFFLPTVVINDIFAYLFGFFFGHHPLIKLSPKKTWEGFIGITMIFMIWV
LFCKCIYA--ASYPTLPRSSFASLVAPFGGFLASGFKRAFKIKDFIPGHGGLDRFDCQVVMMFVYSNFVALKVQLLYY-
>1214707490_Phytophthora_megakarya-Phosphatidate_cytidylyltransferase_

FRVFAMFIILGMAGLVLLTLELVVIPFFVFYNYGFGIYLYHWVFTMYAMLFVMSVLSLKKYKQMYVLGLIVFQYVLTNIFGLFWFLFPVLVINDCFAFFCGKLFGRKPFLRLSPNKTWEGFIGVTVIYFFSF
ILCPCVFVLIILLIFHIWFIFASVVSPFGGFYASAIKRTYNLKDFIPGHGGVDRMDCQLITCFVYNTFIVILVQLVLIL
>321472774_Daphnia_pulex-hypothetical_protein_DAPPUDRAFT_315618_
WRIWVMFAMIALLLVVQV--EIIILPFLIYFFYGLVFLVYHFIFSLYIGGFVWFVLSLVKYMQFFVLLIVVTQLIIRNIFGLIWFIVPVMIVNDVMAYMFGFFFGRTPLIKLSPKKTWEGFIGFTVLFLMIV
LFCPCLYVITVIIFFHLSMVFSSVIGPFGGFFASGFKRAFKIKDFIPGHGGIDRFDCQYLMTFVYSSFIK-QISLLFLL
>22035624_Homo_sapiens-phosphatidate_cytidylyltransferase_1_
WRILTMFFIIMFLLLVQV--EIIILPFLLYFFYGVAFLIYHFIFALYLAGFCMFVLSLVKYRQFFVLLITVTQLVIQNLFGMIWFLVPISVINDITAYLFGFFFGRTPLIKLSPKKTWEGFIGFTVVFFIAV
LFCPCLFLVLVLYFIHIALTFASLIGPFGGFFASGFKRAFKIKDFIPGHGGIDRFDCQYLMTFVYTSFIK-QLVLIYLL
>85091044_Neurospora_crassa_OR74A-phosphatidate_cytidylyltransferase_
FRLFVMFFALMIIVIIQI--EVIIIQTLAYFLHGVIFLAHHFIFTLYVVGFVFFVSTLKGLKQFFMLFLIVGQFIMNNVFGLIWFFLPALVINDIFAYVCGITFGRTQLIKLSPKKTVEGFLGVTVLFILLI
LFCPCVFMFPIMAMIHFFWTFASLIAPFGGFFASGLKRTFKIKDFIPGHGGIDRMDCQFIMFFIYHTFIFLVARLAKVA
>513183097_Gallus_gallus-PREDICTED__phosphatidate_cytidylyltransferase_1_
WRILAMFFIIMFLLLVQV--EIIILPFLLYFFYGVAFLIYHFIFALYLAGFCMFVLSLVKYRQFFVLLITVTQLVIQNLFGMIWFLVPISVINDIAAYIFGFFFGRTPLIKLSPKKTWEGFIGFTVLFFVFF
LFCPCLFVVLVMYFMHIALTFASLIGPFGGFFASGFKRAFKIKDFIPGHGGIDRFDCQYLMTFVYTSFIK-QLVLKSVI
>123493035_Trichomonas_vaginalis_G3-phosphatidate_cytidylyltransferase_family_protein_
LRVCLLQIIFAYLAELLVFLELLLLIRVLYALFGMLFISYHITFGFVMLALIFFVTGLTPDQAYFCAMIVCVPLYARFAESLFWFAVPLCVMNDTSAYFCGRFFGRHQLIKLSPNKTVEGFVGITPITIGII
ALCACEFV--FLLAIHVVILFASLISPFGGFFASGFKRSLGIKDFIPGHGGLDRIDCQLVNTFLYITFV----------
>1160570913_Polysphondylium_pallidum_PN500-CDP_diacylglycerol_synthase_
LRVAMMFTILTFVLFVALLLEMVLIPFLLFFFYAILLFVYHWHFSLYCIGFVSFIFTLRKYRQFFMLMMVVVQFIISNIYGFIWFILPVIIVNDIFAYFNGFFLGKKPLMKISPNKTWEGFIGITLVFYYFF
LCCPCVFLYIFYVIYHLVLLFGSLIAPFGGFFASGIKRAYKIKDFFPGHGGVDRTDCQFIMLFVYLTFIAIGLMLIYLI
>219114335_Phaeodactylum_tricornutum_CCAP_1055_1-predicted_protein__partial_
---------VMVITLVIIMLELVVIPFFVFYTYGFLIYMYMSVFLLYSGTFVLTITTLQRIKQILLLCLTFGQYIMHNIFGLFWYVFPCLVFNDIMAYVSGMTWGRKPFIRFSPGKTWEGFIGITMVIWYLF
LMCPCVFIYF---VVHLWLMFASLVAPFGGFLASAIKRAYGVKDFIPGHGGVDRMDCQFLMLCVHNTFVVLMYLLSVRK
>30696710_Arabidopsis_thaliana-CDP_diacylglycerol_synthase_1_
FRYLWMFVVVMLIAMVVIIFELFLLPIFFLFVYGLSLLIYHAIYLLYIIGFMWFILTLKKYKQFYMLIVVFTQFTVANIFGIFWFLLPALIINDIFAYIFGFFFGRTPLIKLSPKKTWEGFIGVTIISFVLI
LLCPCLFLWFMILVWHLCLLFASIIAPFGGFFASGFKRAFKIKDFIPGHGGIDRMDCQMVMVFIYQSFIVLILNLLFLL

>167538010_Monosiga_brevicollis_MX1-hypothetical_protein_
WRMIVMFAVVLMLIMIQL--EIIILPFLMYFFYGLLFIIYHFIFWLYVIGFVSFVLSLRKYKQFFLLLLVVATLIVRSIYGLVWYLLPSLVINDFMAYMFGFFFGKTQLISISPKKTWEGFLGFTMVFVLFT
LFCP---IGVFLKIFHLVLIFASLIAPFGGFFASGLKRAFKIKDFIPGHGGVDRFDCQFVMVFVYHSFID-PHSR----
>156085088_Babesia_bovis_T2Bo-cytidine_diphosphate_diacylglycerol_synthase_
FRTIFIFIMALVLALIFLGVTYDLGDIFIMLFSTLLLILYHLLFVLFFSGILKFIITLEKIRQFFMLLYVVMQMVIANIYGMIWIILPHMISNDVMAYFFGKMFGKTPLISLSPNKTLEGFIGITTMIAIMI
IMCSCIYLIVIYRCVHMILLFASLFAPFGGFLASGFKRALKIKDFIPGHGGMDRFDCHILMTFLYKTFVAVVIRMLLLF
>79325225_Arabidopsis_thaliana-cytidinediphosphate_diacylglycerol_synthase_2_
------FAIVLLIAMVVIIFELFLLPFFFLFVYGLSLLIYHAIYSLYISGFVWFILTLKKYKQFYMLIVVFTQFTVANIFGIFWFLLPALIVNDIFAYICGFFFGRTPLIKLSPKKTWEGFIGITVISFLLI
MLCPCLFLWLIILVWHLCLLFASIIAPFGGFFASGFKRAFKIKDFIPGHGGIDRMDCQMVMVFIYQSFVVLIISLLLLL
>15235611_Arabidopsis_thaliana-cytidinediphosphate_diacylglycerol_synthase_2_
FRYFWMFAIVLLIAMVVIIFELFLLPFFFLFVYGLSLLIYHAIYSLYISGFVWFILTLKKYKQFYMLIVVFTQFTVANIFGIFWFLLPALIVNDIFAYICGFFFGRTPLIKLSPKKTWEGFIGITVISFLLI
MLCPCLFLWLIILVWHLCLLFASIIAPFGGFFASGFKRAFKIKDFIPGHGGIDRMDCQMVMVFIYQSFVVLIISLLLLL
>1032286774_Arabidopsis_thaliana-CDS2_
FRYFWMFAIVLLIAMVVIIFELFLLPFFFLFVYGLSLLIYHAIYSLYISGFVWFILTLKKYKQFYMLIVVFTQFTVANIFGIFWFLLPALIVNDIFAYICGFFFGRTPLIKLSPKKTWEGFIGITVISFLLI
MLCPCLFLWLIILVWHLCLLFASIIAPFGGFFASGFKRAFKIKDFIPGHGGIDRMDCQMVMVFIYQSFVVLIISLLLLL
>145348827_Ostreococcus_lucimarinus_CCE9901-predicted_protein_
LRQLLMSSI-VHYMMLAVFAELFFTALFWFAVYGAKYLAHHFLYCAYLVGLVYFVLTLRKYAQFFMVAATTMTFNVSNLLGMIWFVFPLLVINDIMAYVFGKIFGKTPLIKISPNKTWEGFLGVTLVALAMY
LFCPCPFLLTVCSFVHLALTFASAVAPFGGFFASGFKRAFKIKDFIPGHGGIDRMDCQVINCFLYSNFILLKILALYLI
>558596964_Spironucleus_salmonicida-Phosphatidate_cytidylyltransferase_
FRIIAMLISIYILTLMCSLMEISI---FK-----LIVIEYLALAVIIFSFISLTIIFSLKCLTFFFSGILITIFLILNQP---LFLTQLLVINDSFAYFCGKLFGKHKLITLSPSKTWEGFVGVT-------
----CVYI-----LEVAVQIFVATVAPIGGFFGSLVKRSCGIQDFIPGHGGFDRLDCQILT-FSYAWCA----------
>123473371_Trichomonas_vaginalis_G3-phosphatidate_cytidylyltransferase_family_protein_
LRIVVIEVLMIYLAELIIFLELLLPIFILYLISGILFIAFHIAFLIVMLALFLFVTGLTPDQAYFCAFIIGIPLYTKFADSLFWFSIPICVVNDTAAYFCGRLFGRHQLIKLSPNKTVQGFVGITPLVIGAI
AICRCEFI--FILAIHTVILFASLVSPFGGFFASGFKRSLGIKDFIPGHGGIDRVDCQLVNIFLYITFV----------
>20143480_Homo_sapiens-phosphatidate_cytidylyltransferase_2_
WRILAMFFIILMLIIVQI--EIIILPFLLYFFYGVTFLSYHFIFTLYLIGFCMFVLSLVKYRQFFVLLIVVTQLVIHNLFGMIWFIVPICVINDIMAYMFGFFFGRTPLIKLSPKKTWEGFIGFTVVFLLLV

MFCPCLFIVIVMYFIHIALTFASLIGPFGGFFASGFKRAFKIKDFIPGHGGIDRFDCQYLMTFVYASFIKLQFTLIFLL
>71028080_Theileria_parva_strain_Muguga-phosphatidate_cytidylyltransferase_
FRIIFLFLIFCFISVVLAIKEILLVYFLEIALVLLVLVAYHLIFMLTMAGMIKFVISMERYKHFLVVLYVVCEMVITNIYGMVWFLCPHMVANDVLAYLFGRFVGKRPLIVISPKKTVEGFLYLTTFLVLIF
LLCPCVYLLLLYSFLHLVLLFASLFAPFGGFLASGFKRALKVKDFIPGHGGIDRFDCHILMGFFYKTFVEVMFKLRQ--
>50759504_Gallus_gallus-PREDICTED__phosphatidate_cytidylyltransferase_2_isoform_X2_
WRILAMFFIILMLTIVCVIKEIIILPFLLYFFYGVTFLSYHFIFALYLTGFCMFVLSLVKYRQFFVLLIVVTQLIIHNLFGMIWFIVPICVINDIMAYMFGFFFGRTPLIKLSPKKTWEGFIGFTVLFLLLV
MFCPCLFIVVVMYFIHIALTFASLIGPFGGFFASGFKRAFKIKDFIPGHGGIDRFDCQYLMTFVYASFIK-QFTLKALV
>788006687_Trypanosoma_brucei_gambiense_DAL972-phosphatidate_cytidylyltransferase_
LRVLIMFLLVIEIFLLLC--EVSINKLCT-----SMYYYNAMIFGFPLVGMVGFVLSLRKYRQFLMLLYVTAQAQISNVMGMLWFVLPICVINDTWAYIFGKLFGRTKLLALSPKKTVEGFVGVTIIWFWFF
LMCACLFMLTICAVQHLVFAFASLIAPFGGFFASGLKRAFKMKDFIPGHGGIDRMDCQGIMFFVYQSYVPICAQLLLLL
>813200016_Saprolegnia_parasitica_CBS_223_65-hypothetical_protein_SPRG_14247_
FRILLMFLIVLPCALVVLAIELVLMPFFLFFVYGLMFIVHHAIFALYVVGFVSFVFSLRKYKQFFVLLMIVAQFMISNMFGLIWFLLPCLIINDVFAYIFGFFWGRTPLIKLSPKKTWEGFIGGTVLWMGFF
MMCPCIFLMLVIAIYHVFFIFASIVAPFGGFFASGFKRAFKIKDFIPGHGGVDRMDCQIIMMFVYTNFISIVLRLVFFL
>50553064_Yarrowia_lipolytica_CLIB122-YALI0E14443p_
FRILVMFFVLAIVALVLVILEVILLPVLAYFLEGIIILFHHFLYCLYILGFVFFVASLEKYRQFFMLLFGVVQFIVNNIFGLFWFFVPALVINDIFAYLCGITFGKHQLIAISPKKTVEGFLGITIIWVIFF
LFCPCVFILTFAAIFHGILSFASLVAPFGGFFASGLKRAFQVKDFIPGHGGIDRMDCQFLMFFLYETFVVLLMTFFEKL
>301106959_Phytophthora_infestans_T30_4-phosphatidate_cytidylyltransferase__putative_
LRLFMMFLIIALVLLVQI--ELAVVPFFYFYVYSMQFIALHFLFSLYASMFCITVLSFKKYKQMLIVFMIVFQFAIDNIKGMFWFLLPCLVINDCMAYFSGKAFGKKPFMKLSPNKTWEGFIGITCIFFVYI
LICTCVFIILVMLVMHVLLMFASLVAPFGGFFASAIKRAYDIKDFIPGHGGIDRMDCQFIMMAVHTTFIQ---------
>302830372_Volvox_carteri_f__nagariensis-hypothetical_protein_VOLCADRAFT_56233__partial_
FRVVALFIIISVLFFIQASLELFILPLFFFWLYLIKLALHHLIYSLYMAGFVGFVLSLKKYMQFYMILITTVPFFVSNIFGLIWYVLPALIVNDICAYLAGFFFGRTPLIKLSPKKTWEGFIGLTLVIFFLV
MLCPCVFLWLFVAILHLSLSFASIIAPFGGFFASGFKRAFHMKDFIPGHGGVDRFDCQILMVFCYYSYILVLAKLLVLL
>544214405_Cyanidioschyzon_merolae_strain_10D-phosphatidate_cytidylyltransferase_
FRVLVMFTIVLQVLMVVLALEVLIIPFLLYFIYGLSYLLHHFVFWLFLIGFMMFVLSLRPYRQLFMLLVVVAQFIIFNIKGLIWFLLPAMIINDIMSYCVGFFFGRTRLTALSPKKTWEGYIGITFVAILLH
LMCPCTFVFLLIKIIHMVLAFASTLGPFGGFFASGVKRAFRVKDFIPGHGGVDRMDCQLLMAFVYLNAVVLYIDLLHLL
>224012218_Thalassiosira_pseudonana_CCMP1335-phosphatidate_cytidylytransferase_like_protein__partial_

LRILVMFIILSVVAFIGIVLELVVIPFFFFYAYSVVIYALAALFTLYTGTFVLTIATMQTIKQLLVLCLTVGQYIMHNIYGLIWFTLPILVINDVMAYFSGVTCGRKTFMELSPNKTWEGFIGVTMIVWYLW
LMCPCIFVAIIIVILHISLLFASLVAPFGGFLASAIKRAYGIKDFIPGHGGLDRFDCQMIMLCVHNTFVVMMYLLFMI-
>799327131_Cryptococcus_neoformans_var__grubii_H99-phosphatidate_cytidylyltransferase_
MRIFIMFILLMPMLLVLCTLEVTLDSNFVYFLYGIIFFANHFIFMLYVVGFVGFVANLQRLRQFFILLLVVVSFIVNNILGLVWFFVPALVINDVMAYVCGKLFGKTPLIKLSPKKTVEGFVGITLLFIAWF
WMCPCVFFILIYAFIHLVMTFASLVAPFGGFFASGFKRAFNIKDFIPGHGGMDRMDCQFMMLFVYSSLIVMVVSLLLLL
>330790000_Dictyostelium_purpureum-hypothetical_protein_DICPUDRAFT_44595_
---ACMFIVLTFVLFVALLLEMILIPFLFFFFYAILLFVYHWHFSLYCIGFVSFIFTLRKYRQFLMLMMVVVQFLISNIYGLIWFILPVIIVNDIFAYFNGFFLGKKPLMKISPNKTWEGFIGFTLLFYYFF
LVCPCVFFYFFYIIYHLVLLFGSLIAPFGGFFASGIKRAYKVKDFFPGHGGVDRTDCQFIMLFVYLTFIPIGIMLIYLI
>66825773_Dictyostelium_discoideum_AX4-CDP_diacylglycerol_synthase_
LRVAFMFTVLTFVLFVALLLEMILIPFLFFFFYAILLFVYHWHFSLYCIGFVLFILTLRKYRQFLMLMMVVVQFLISNIYGLIWFILPVIIVNDIFAYFNGFFLGKKPLMKISPNKTWEGFIGGTLLFYYFF
LICPCVFFYLFYIIFHLVLLFGSLIAPFGGFFASGIKRAYKVKDFFPGHGGVDRTDCQFIMLFVYNTFIPINIMLIYLI
>731439315_Vitis_vinifera-PREDICTED__phosphatidate_cytidylyltransferase_1_
MRRVWMFAIIMLIAMVVIIFELFLLPFFFLFVYGLSLLIYHVTYFLYIAGFMWFILTLKKYKQFYMLIVVFTQFTVANIFGIFWFLLPALIVNDIAAYIFGFFFGRTPLIKLSPKKTWEGFIGITIISFVLI
MLCPCLFLWFVILVWHLCVLFASIIAPFGGFFASGFKRAFKIKDFIPGHGGIDRMDCQ---------------------
>855310982_Blastocystis_hominis-uncharacterized_protein_
IRIFAIFIIVMYVMLIAIFGELLVIPFFMFIVFGSAMVVYHFIVILYSSLFVILVLSLKKLKQIILIIIVIYQAAVPMIMGIIWVVLPCTVANDTMAYFFGMAFGRKPLT-LSPNKSWEGFIGISIVIEIMY
FYCPCYYFILFLEIIHFFLLFAALVAPFGGFFASAIKRAYGKKDFIPGHGGLDRFDCQFIMYFMYNTFI----------
>240256083_Arabidopsis_thaliana-Phosphatidate_cytidylyltransferase_family_protein_
MRCLWMVVIIMLIAMVVIIFELFLLPFFFLFVYGIQLLIYQVIYFLYIAGLIWFILTLKNYKQFYMLIVVFTQFTVANIFGIFWFLLPALIANDVAAYFFGFYFGKTPLIKLSPKKTWEGFIGVTIISFIFV
LLCPCLFFFSITLVWHFSLLFASIMAPFGGFFASGFKRAFKIKDFIPGHGGFDRMDCQMVMVFIYQSFIVLISSLLYLL
>754344494_Capsaspora_owczarzaki_ATCC_30864-hypothetical_protein_CAOG_08539_
WRIWIMFCIVLFLVLVQI--EIVILPFLFYYLYGLFYMAHHLIFSLYMLGFVSFVATLKKYKQFFILVLIVTQLVMLNIFGLVWLILPALVVNDVMAYMFGFFFGRTRLTQLSPKKTWEGFIGFTYIFFVLY
LFCPCYFIGLVVYVLHLSMTFASIVAPFGGFFASGFKRAFKIKDFIPGHGGIDRFDCQFLMTFVYSSFVPISIALLFLL
>169847770_Coprinopsis_cinerea_okayama7_130-phosphatidate_cytidylyltransferase_
VRIFVMFILLMAMVLVLCTLEVTLLKKFAYFLYGIIFFANHMIFTLYIIGFMGFVMSLKKLKQFFMLLIIVFSFIINNILGLIWFWVPALVVNDCFAYIWGKTMGRTPLIKLSPKKTVEGFVGFTIIFVLWY
FMCPCVFLISIYAYLHLVLCFASLVAPFGGFFASGFKRAFDIKDFIPGHGGMDRMDCQFLMVFVYSSLIVLIVNLLILL

>929761067_Sphaeroforma_arctica_JP610-hypothetical_protein_SARC_06068_
WRFWVLFVIIGLVLMVGIVAEINIVPVLF--YFLILPLAHHFFFLLYIVGIVVFVLTLRNYRQFFVVVLLGSSAIFSNITGLVWFLLPVIVINDCTAYIFGFFFGHTPLIELSPKKTWEGFLGITMFIYGFL
FFCPCIFIVIYTKILHLVFLFGSLVAPFGGFMASGFKRAFKVKDFIPGHGGFDRFDCQFLMSFVYQSFIDIHLKLQAAA
>159467317_Chlamydomonas_reinhardtii-CDP_DAG_synthetase__partial_
FRIVLLFIIIAVLFFILLFLELFILPLFFFWLYLIKLAVHHLIYSLYMAGFVGFVLSLKKYLQFYMILITTVPFFVSNVFGILWYVLPALIVNDICAYLAGFFFGRTPLIKLSPKKTWEGFVGLTLVIFYLY
MMCPCVFLAAFVAILHLSLAFASIAAPFGGFFASGFKRAFHMKDFIPGHGGVDRFDCQIVMVFTYWSYILVSAKLLVML
>66362538_Cryptosporidium_parvum-cytidine_diphosphate-diacylglycerol_synthase_
WRIIILFLILATSMLVIS--EVNLLPFLG-----WVIIVYHLIYVGALFGFIAFILSLRNLRQFFLLLLVVTQFMIANTYGLIWFILPSLVINDIFAYIVGKIFGKTRLFRLSPKKTVEGFIGITIFAIILV
LFCPCVFIIFFIPSFHIAIIFAAFVAPFGGFFASGLKRALRIKDFIPGHGGIDRFDCQILILFLYRAFIVVLAMLLILI
>17539014_Caenorhabditis_elegans-Phosphatidate_cytidylyltransferase_
WRLIIMFTIVRTLFLVLIFKEIIIFPFLLYFFFGLIWLVYHLVFALYCIGFVSFVLSLRKYMQFFLLLLIVSQFIIQNIFGLIWFLAPVMIICDIMSYMFGFFWGKTPLIKLSPKKTWEGFIGFTVVFILLA
LFCPCAFVLRILCFFHIALLFASILGPFGGFFASGFKRAFKIKDFIPGHGGLDRFDCQLLMTFVYHSFIKLQITLIFLL
>124808255_Plasmodium_falciparum_3D7-cytidine_diphosphate_diacylglycerol_synthase_
LRQFILYFILAFCILVVT--EIILLPIFFMTLGIVILLLYHINFILAFVGFVWFILSLRKLKQFILSLFIVTQMHIANIYGMIWFIVPVSVVNDIFAYVFGILFGKTRLIQLSPKKTVEGYVGITIVWILIF
LFCPCIFVILIYSMFHLMLLFAAFLAPFGGFFASGFKRALKIKDFIPGHGGVDRFDCQIFIMFIYKTFVYLQLNINTIK
>168032592_Physcomitrella_patens-predicted_protein_
WRFVWMFIIVMLIAMIVILYELFLLPFFLLYVYGLNLLIYHLIYSLYIAGFVWFILTLKKYKQFFMLLVVFAQFTVANIFGIIWFLLPALIVNDIMAYFFGIFFGKTPLIKLSPKKTWEGFIGITVMSFMLV
MLCPCLFLWFFVMVRHMAFLFASLVAPFGGFFASGFKRAFKIKDFLPGHGGMDRMDCQMVMVFIYQSFITLILNLLFLL
>17539016_Caenorhabditis_elegans-Phosphatidate_cytidylyltransferase_
WRLIIMFTIVRTLFLVLIFKEIIIFPFLLYFFFGLIWLVYHLVFALYCIGFVSFVLSLRKYMQFFLLLLIVSQFIIQNIFGLIWFLAPVMIICDIMSYMFGFFWGKTPLIKLSPKKTWEGFIGFTVVFILLA
LFCPCAFVLRILCFFHIALLFASILGPFGGFFASGFKRAFKIKDFIPGHGGLDRFDCQLLMTFVYHSFIK-QITLQSSL
>50290049_Candida_glabrata_CBS_138-hypothetical_protein_
FRIFIMFFVLSICMLIQI--ECIVLPTLIYYLEGLFLVVHHFIYCFYVFGVVLFVCSLRKLKQFLMMLFVVAQLVTKNVLGLFWFLLPCLVINDIFAYLCGITLGRTKLIAISPKKTLEGFLGFTALAIILL
LLCPCVFTKYIVKIFHLNLTFASLVAPFGGFFASGLKRAFKVKDFIPGHGGIDRVDCQILMSFLYTTFIIIILNFNKLL
>955163202_Bodo_saltans-CDP_diacylglycerol_synthetase__putative_
FRVLLMFLLIAAVIPLFIVMEVLILPFLSALMTLLRVLYHIMTFGLYVIGLVAFVLSLKKYRQFFMLFFIVLQLQVSNMLGMVWFLLPLCVVNDIWAYAFGKSFGRTRLLALSPKKTLEGFLGLTMIFFWFF

LLCPCLFLVTVVCVYHLAFVFASLIAPFGGFFASGLKRAFKLKDFIPGHGGMDRMDCQIIMLFVYRNVVSICVALSV--
>758977095_Ustilago_maydis_521-hypothetical_protein_UMAG_02266_
IRLLIMFILLLPMLLVICTLEIVLQLSFAYFLYGIIFFAHHFLFMLYVFGFMAFVSNLKRLKQFFMLLLIVFSFIVNNILGLIWLWVPALVVNDVFAYICGMTFGRTPLIDLSPKKTVEGFVGITEIFYGWF
FMCPCVFLIAFWSFLHLVMAFASLVAPFGGFFASGFKRAFNIKDFIPGHGGLDRFDCQFLMLFVYSSLIVLVVQLLYIL
>157871233_Leishmania_major_strain_Friedlin-cdp_diacylglycerol_synthetase_like_protein_
VRIILMFLWIVLLFVI--STEVTILPVFV-----VFLFDVYLLFAFFMLGLVIFVLSLRKYRQFFMLMLCQVQGEMRNMVGMIWFLLPVCVVNDIWAYVFGKCFGRRKLLSLSPKKTVEGFLGLTVIWFWFF
LMCPCIFFVSFVSAKHLVLTFASLLAPFGGFFASGLKRAFKLKDFIPGHGGMDRMDCQGLMLFCYRTYVPICARMLVLL
>359494156_Vitis_vinifera-PREDICTED__phosphatidate_cytidylyltransferase_1_
MRYMWMFIIILLIAMVVIIFELFLLPFFFLFVYGLSLLLYQVIYFLYIAGFMWFILTLKKYKQFYMLIVVFTQFIVGNIFGIFWFLLPALIVNDIAAYFFGFFFGKTPLIKLSPKKTWEGFIGVTVISFVLI
LLCPCLFLWFIVLVWHLCLLFASIIAPFGGFFASGFKRAFKIKDFIPGHGGIDRMDCQMVMVFIYQSFVVLILNLLYLF
>6319503_Saccharomyces_cerevisiae_S288C-phosphatidate_cytidylyltransferase_
FRVFVMFFTLSACVLIQI--ECIVLPTLFYYLDGLFFIVNHFIYCLYLMGFVLFVCSLRKLKQFLMLLLVVFQLIIKNVLGLFWFLLPCLVINDIFAYLCGITFGKTKLIEISPKKTLEGFLGFTALAIILI
LLCPCVFLKVIVKIFHLNLTFASLFAPFGGFFASGLKRTFKVKDFIPGHGGIDRVDCQFIMSFLYETFIVLILNLIRSI
>170093043_Laccaria_bicolor_S238N_H82-predicted_protein_
LRLFIMFVLLLAMLLVLCTLEVTLDPSFAYFLYGIIFFANHMIFTLYTIGFMGFVMSLKKLKQFFMLLLIVMSFIVNNILGLIWFWVPALVINDCFAYIWGKTLGRTPLIKLSPKKTVEGFVGWTVIFVIWY
FMCPCVFLWTIYAYLHLLLFFASLVAPFGGFFASGFKRAFNIKDFIPGHGGMDRMDCQFLMVFVYSSLIVLIVGLTRGI
>667639453_Vittaforma_corneae_ATCC_50505-hypothetical_protein_VICG_00593_
FRIIVMFLLCMLLLLSILFGELIISEYLA--FFFLPVRLSFPLFALYSIGLMLSVISFTRLVQLLIVSVCSLALSIKNIIGKFFYVYPALVINDIFAYLIGKSFGRTPLISLSPNKTVEGLVGITFITMVLL
KLDM------LFPVFHLLFMAASFFAPFCGFLASAIKRAFGKKDFIPGHGGIDRFDCQVLMFFYYKGVMIAYLDNLRV-
>303285967_Micromonas_pusilla_CCMP1545-predicted_protein_
----------M---------ELFLLPFYGFFSYGLKLLLHHMMYLGWMCGFVLFVLSLRKYVQFFMVATVLMQCFVANVLGLIWFVFPVIVVNDIAAYLCGFFIGRTPLIKISPKKTWEGFIGVTILAPFIA
MLCPCLFVWLVASFVHVVFWLASIIAPFGGLFASGFKRAFKIKDFIPGHGGIDRMDCQMVMVLLYANFVTVKVLVGNVM
>503141003_Stigmatella_aurantiaca-phosphatidate_cytidylyltransferase_
LRVVVLVLLLKVSILLAA--EYYILKLIV-----VVLAGFAWVAGFF---FFAWTYHLIRLPAPSTFLYAAVGALSALRMGMAWVICALITWNDTVAYFAGRFLGRHKLYEVSPNKTWEGFFGVSVGGFARF
FACV-------------VTIAGGILGPLGDLCESMLKRAYGVKDSIPGHGGIDRIDALLFNPLIYQFIR----------
>499871892_Myxococcus_xanthus-phosphatidate_cytidylyltransferase_

LRVVTLVLLLLYSA-LAAAAEYYI--LMA-----VLLTGTAWLI---VFAFFAWIYHLFKLAAPTNFLYGAVGAVSALRLGLAWVICALITWNDTAAYFFGRFLGKHKLYEVSPNKTWEGFFGLSVGGFARF
FWCV-------------LLIAGGILGPVGDLCESMLKRAYGVKDSIPGHGGVDRIDALLFNPLVYQFVR----------
>903049114_Vibrio_cholerae-phosphatidate_cytidylyltransferase_
MRILILILILLAFLALVVLLEWTF---MI-----LVIAINMTAFIVLGIGSLWWLVSSGLITPRPSFGLFTLLFWSVLFLGAKLVLFVCLVWADSGAYFVGKSLGKHKMAAVSPNKTIEGLVGVAMLVYWVC
F-----F------SPVLLILLTVVISVLGDLVESMFKRVSGIKDSIPGHGGIDRIDSLTAAPVLLFLF-----------
>494229625_Flavobacterium_frigoris-phosphatidate_cytidylyltransferase_
TRIAVYIFILTFIFGVLITVNLII---FI-----YLYFSTFLILSATLIVFIKCIHFLFDKIRIFYLGYIILPIMTKIPFGYNILISIFLIWNDTFAYIVGKSIGKTKLFRISPKKTIEGFFGLSVIAYLVY
YG-----------FWI-IIIIVGVFGTIGDLIESKFKRIAGVKDSMPGHGGIDRLDSVIFVPILFQILN----------
>488140929_Yersinia_pseudotuberculosis_complex-MULTISPECIES__phosphatidate_cytidylyltransferase_
LRILILVIALLVFIVTVVMLEWGL-AFIL-----LLMLVTPWLMGWWVAALMLVLTYPRSVSRNLF--ILTIIFWGMFALGAWWLLYVMLVWADSGAYMFGKLFGKHKLAKVSPGKTWEGLIGLSALILLFY
APK-------------LICVVAALASVLGDLTESMFKREAGIKDSIPGHGGIDRIDSLTAAPVCLLLVF----------
>501357985_Leptospira_biflexa-phosphatidate_cytidylyltransferase_
TRLIVLYVMISYLFYTVMYLELYFSKFFT--YYILGFLRGFPIFLLVALSITVWILILKRLDALVLPIYIAIPFLLLLALGAYYIFLVSITFSDAGAYFGGRWFGKHPAGKISPKKTWEGYVVLAVIGQVLT
WVLP---------GLEIILFVISVISVMGDLAESAMKRDAKIKDSIPGHGGIDLADALLFTPAYYVFKG----------
>488700590_Cystobacter_fuscus-phosphatidate_cytidylyltransferase_
LRVFSLVVLLMLTL-LVAAIEYYI--LIL-----ALLTGGAWVAFFLIFAFTFHLI---RLQAPVMFLYGSVGAMCALRQGLMWVIAALITWNDTAAYFAGRFLGRHKLYAVSPNKTWEGFAGVSVVGFARF
FTCV-------------FLICGGILGPIGDLCESMLKRAYGVKDSMPGHGGIDRIDALVFNPLVYTFLR----------
>446845190_Proteobacteria-MULTISPECIES__phosphatidate_cytidylyltransferase_
LRIFVLVIALLVFIVTCM--EWGL-SFVL-----LLMLVISWALGWWIVALLLVLFYPGSAIRNTF--VLTIVFWGMLALGAIWLLYVMLVWADSGAYMFGKLFGKHKLAKVSPGKTWQGFIGAAAVIW--G
Y-----W------NDVLICIVAALASVLGDLTESMFKREAGIKDSIPGHGGIDRIDSLTAAPVCLLLVF----------
>446854946_Vibrio_cholerae-phosphatidate_cytidylyltransferase_
LRRWWMFVLQ-LYLFFFLFMEFLIMTR-------IPYYGFIFIVYVFYLPMVMVLIGDTKFISAIMTVFCISHYLLVLPSGMLLFLLVMQF-NDVAQYVWGKSFGKHKIVKVSPNKTWQGFIGLVVTVYFAY
LAGL-------------VGVIIALSGFIGDLVISSVKRDLRIKDTIPGHGGIDRVDSLMFTFFLYLYY-----------
>813111699_Saprolegnia_parasitica_CBS_223_65-phosphatidate_cytidylyltransferase_
ARLLVLVLFVLPAYLLVVSIEFALLLLPEYLYL-VACGTYYNHYIPSKHVFALIAIAVHCAIVLIVYGYVALLPIAAFMVGRRLALGFLVVWADTGAYITGHVIPGYRLAHISPKKDVEGTLGFGILLL-LF
VPSVMYL----YTLWQLVFVVGAVISRYGDLLASLIKRLAQVKDTIPGHGGMDRVDALLFT-AVLHRLSS-QPYLFPV-

>383505582_Drosophila_melanogaster-FI18858p1__partial_
WRIWIMFAIIGLLITTLVVKEIIILPFLLYFFYGLVFLVYHFLFALYIIGFVWFVLSLVKYIQFFVLLIVVTQLIIQNIFGLIWFIVPVMIVNDVMAYVFGFFFGRTPLIKLSPKKTWEGFIGFTVLFILFV
LFCPCLF-FGLLYFWHISLLFSSIIGPFGGFFASGFKRAFKIKDFIPGHGGIDRFDCQFLMTFVYSSFIK-QINLIYLL
>237844219_Toxoplasma_gondii_ME49-phosphatidate_cytidylyltransferase_
VRLLILFVILAVSGLVVA--EIIVLPFFLMGFGLSLALLFHLVYTAGFIGLVWFILSLRKMRQFLVLVFIVGQMQIANIYGLIWFILPTLVINDVSAYICGMLFGRTRLIRLSPKKTVEGFVGFTLLWVIVQ
LFCPCAFEWLFFSFFHIVLLFAGFLAPFGGFFASGFKRAARIKDFIPGHGGVDRFDCQILTMFLYTSF-AVAVSMVVLK
>303282267_Micromonas_pusilla_CCMP1545-predicted_protein_
LRALVLGAVIAVFLFMLAFLEYFL--VTM-----MCFGGLNIVTMMGAYGVTATLLMTKQ-DHFFFFFYCGFLFWVKLRAGLIATIIAVCIISDVGAYVVGKTFGKHQLI-LSPNKTIEGAVGVTVGAFALL
LVQ-------------TMLCVIFWSAVAGDLMESVMKRNANMKDSIPGHGGFDRFDSYLFTIAFFVTVMV---------
>491960837_Haemophilus_influenzae-phosphatidate_cytidylyltransferase_
LRLIVLVLALFFFLALAI--EWTFQPIFV-----LGIAGVFQHQLLLINAVSWWGLALLLISPKKNFAFSTLIVAGVLRLGLFLLLYVFLVWADSGAYFSGRAFGKRKLAKVSPGKSWEGVIGIALVLFIFF
SGRN---------TFI-ILVATVAISVLGDLTESMFKRESGVKDSIPGHGGVDRIDSLTAAPFYFFFVL----------
>499227735_Mycoplasma_pulmonis-phosphatidate_cytidylyltransferase_
IRLFIVFLSVFRIFFRIVFLTFMI--FVF--ILFIIILKPYYLIIAMILFFVSYKYISQADNNLILLTFIMVLVFTKFILSL-FFYWPILFICDSFGFFGGLALGKRPFSMISPKKTWEGFIGIFIVFFEFI
FSKI---------NFLISLAFAPLISIFGDLFFSWVKRVNNIKDFLQGHGGVDRFDSMICL-SMLLILI----------
>499689969_Thiomicrospira_crunogena-phosphatidate_cytidylyltransferase_
LRISLLVALLKQWFFTAVFIEWFF--TSA-----LIVADFMLTLVMVIMATTVILYQRRAHFIRLLLLILVNFVLLFFVSGVV--LLSLVIWIDTGAYFSGRRFGRHKLAHVSPGKTWEGVYGLAFVIIGLL
QSLS------------AFITVIGLFSIFGDLFESLLKRQANLKDSLPGHGGVDRADSLLVAPMFVQWVS----------
>551660882_Guillardia_theta_CCMP2712-hypothetical_protein_GUITHDRAFT_70608__partial_
FRRFAMFLVISIVALICIAGEILLIPFFA--YFILQILSHHFKFALWCIGFCTFILSLEKYKQFFILLMVVATCMIKNMYGMIWFFVPVLVINDVYAYVFGRFWGKTPLIKLSPKKTWEGFIDATLVR---L
MMCSCVFLLAMIMFWHINMMFASLIAPFGGFFASGFKRAFRIKDFIPGHGGIDRMDCQVGL-NVERAGIEISIAKLFRT
>598018150_Auricularia_subglabra_TFB_10046_SS5-hypothetical_protein_AURDEDRAFT_111348_
ARLFIMFILLMVMLLVVCSLEVTLTASFAYFLYGIIFFANHLVFMLWTMGFVGFVASLKKLKQFFMLLVVVVSFIVNNILGMIWFWVPAFVINDIFAYVWGVTLGRTPLFKLSPKKTVEGFVGFTIILVIYF
FMCPCVFMLLIYAYFHLALCFASLVAPFGGFFASGFKRAFDIKDFIPGHGGMDRMDCQFLMMFVWSSLVVLIVGLLFLL
>488956239_Campylobacter-MULTISPECIES__phosphatidate_cytidylyltransferase_
NRILVMIIIAIFIFIVVLYLEAKL---FA-----ISPGSSHALFGILLLLVVGYLV----YKASP-YIYPSLPLWQVYLDGMFALFWLIIVACDSGAYFIGKLMGKTPFS-TSPNKTLEGVIGIASVITILF

V-------------WLLLCFFVAIFAVIGDLLESYFKREAGVKDSIPGHGGMDRIDAVIIA-FVAL-------------
>159467319_Chlamydomonas_reinhardtii-phosphatidate_cytidylyltransferase_
FRIVLLFIIIAVLFFILLFLELFILPLFFFWLYLIKLAVHHLIYSLYMAGFVGFVLSLKKYLQFYMILITTVPFFVSNVFGILWYVLPALIVNDICAYLAGFFFGRTPLIKLSPKKTWEGFVGLTLVIFYLY
MMCPCVFLAAFVAILHLSLAFASIAAPFGGFFASGFKRAFHMKDFIPGHGGVDRFDCQIVMVFTYWSYILVSAKLANAL
>499174631_Synechocystis_sp__PCC_6803-phosphatidate_cytidylyltransferase_
TRIVIGAFLLLWFAAILVYLEYFM----------APKTVPHTDFFPLTGALICFYLLFQP-MTIILLFYGGYLYWVRLRLGLLVTILAFCIWADIGAYIMGKWLGRTRLS-ISPKKTVEGSLWGSLLVVLGY
LET-------------ALLLLIGIVSLLGDLTESMMKRDAGVKDSIPGHGGIDRTDSYVFTPLYFVLLL----------
>488157674_Neisseria_meningitidis-phosphatidate_cytidylyltransferase_
LRIMWLMLMLYQLAAFLILIEYAM-GLYL-----LVVLPLVYVLAFWLAVMPLWLR----FKRL-YVGWLLVMFWFALVSDALPLLAVMLVWADICAYFSGKAFGKHKIAAISPGKSWEGAIGVVYMTVSAL
AFTV-------------LILVLTVVSVCGDLLESWLKRAAGIKDSLSGHGGVDRTDSLIAVISAAMSVL----------
>501920821_Brachyspira_hyodysenteriae-phosphatidate_cytidylyltransferase_
MRICLTFLIMYVYFHLAIILEIFMQKLMV-----IICSMLSVMFALIAILFIINMFKVHVFEKGIFFIYVGLGHISLMRFGKYYIVFILCAWSDTGGYIVGRKLGKHKLSSASPNKSYEGLVGITIPF-ILF
LLYLGNF------FQIMLLVIFTFTGFLGDMGESLIKRMYDTKDSFPGHGGVDIFDSVILTPIYVLLLQ----------
>503679597_Clostridium_acetobutylicum-phosphatidate_cytidylyltransferase_
LRLVILLI-LLILIFVALMIEYYV--LYLIAYYV---LIYRNYFVLFMIVIGIFIMLCVPTKNYILFIYVAVFFIALTNDGNYLLWIIFASWCDTLAYYSGRIFGKTKLCKVSPKKTIEGSIGLSVIGTVYI
INAI----------YHIIIILCGVFSQFGDLAASSIKRHAQVKDYIPGHGGIDRFDSILFTVVYYSFIV----------
>551642526_Guillardia_theta_CCMP2712-hypothetical_protein_GUITHDRAFT_78510_
LRFVVLVSWISWFL-VVASREYYMCKAYM-----AY-LPAYDCLPVAYILIVGYLLTLKREKTIVMIFYVGYLFWVRLRAGAVVTWWTMSIASDVGAYFAGKNFGKTKLS-VSPNKTMEGFFGACSIFTTGL
MIGP-------------FYFMISALGLLGDLTVSLFKRDAGVKDTLPGHGGIDRIDSYMLTAPYFKVGN----------
>763384257_Chondromyces_apiculatus-phosphatidate_cytidylyltransferase_
LRLLVALLLLLWWAFLTT--ELFMHPDSQ-----LALWGDAALVLLLILPLAGMLLTLARMQAAVFPLYLGGGALAMLRRGPGFVVLALLSWSDTGAYFAGRFLGRHKLYAVSPKKTVEGALGASVLGV-IF
VPVP------------LVLVVAGALGQAGDLGESLIKRSVGAKDSIPGHGGIDRVDALLVTTMVYMFWW----------
>504342563_Sinorhizobium_meliloti-phosphatidate_cytidylyltransferase_
LRALVLVLATIFFL-LAILLEWSIERFQV--GLV---LLYMISPGLALCVLAAALWVLIK----SGIVYAGLTSLAAIRGGLMAMLFVFVVWTDIFAYFTGRAIGGPKLAAISPGKTWSGAIGIGVLAV--F
MELR------------PVILVLSVASQIGDLFESFVKRRFGVKDSIPGHGGVDRVDGLIFAALVLQFLLAL--------
>501046734_Anaeromyxobacter_sp__Fw109_5-phosphatidate_cytidylyltransferase_

LRTAVLAVITVLFVLAAA--ELVM---FGEVF--AVAGNLPWSLALAGATIVLFVFSLFRLEVPMLWLYCGVLSLVGLRLDVGWVILAFVTWNDTFAYFTGHALGRHKLLRISPKKTWEGFAGVSVLGLVTL
LSGL------------VAILGGALLGPLGDLAESMVKRAAGVKDSIPGHGGLDRIDALLFVPWVCAYLR----------
>570975888_Phytophthora_parasitica_P10297-hypothetical_protein_F442_14396_
VRLLVLITFLLAAATFFVSIEYALSRALTSVFLIVACLVKAFTLTMHLSGVVVIVFRLQTGPTFILYLYIIGSVIVAFVDSYRLLIVLLVVWSDTGAYLTGKLLEHYPLAHLSKNKDYEGTLGFGVTAFISL
LAA-------------IGFVLAVIVGRLGDLFESLLKRAAGVKDSIPGHGGVDRIDALMFALVRYASIV----------
>489535707_Pseudomonas-MULTISPECIES__phosphatidate_cytidylyltransferase_
LRILILALGFLAFLFILVTLEWAL----------ARFLFLHFPLAPWVLGAAVLWWAVATLTPQVTLVIGLLIAWQGLVLGNWLIMAVMLVWADIGAYFSGRKFGKRKLAKVSPGKSWEGVFGLSLLIAAVA
REFA------------LVGGVIVLISVVGDLTESMFKRQSGIKDSLPGHGGVDRIDSLTAAPVVLWMAA----------
>568047750_Phytophthora_parasitica-hypothetical_protein_L914_09981_
FRIVVLVTFLLAATTVMIT-EYALSGGPVVGFLVVVCLVYGFTLTTHLGGMVIILVASTPKVAFFVLLYVTGTILVAFVDTLYLLIALLIVWSDTGAYLIGKMLARYPLAHLSKNKDYEGTVGLGIVTIVAV
LFM-------------VGFVLAVIVGRMGDLFESLLKRAAGVKDSIPGHGGVDRIDALMFAIVRYALQS----------
>503618747_Erysipelothrix_rhusiopathiae-phosphatidate_cytidylyltransferase_
MRTIVLFSALGAFL-IGLLLEIYL--LII-----IITLPYISFAVLIMTLFALTVF----DWTFVIIMMLVLAVTVVLEYKLV-FIYVLATYTDTFAYFGGMMFGKHKLIRISPKKTIEGAVITSALLFIFM
F---------IYVLVIIVGLIIPFIGQIGDLAFSSIKRHFDVKDFFPGHGGVDRVDSVIFALTIITIFI----------
>813154572_Saprolegnia_parasitica_CBS_223_65-hypothetical_protein_SPRG_07150_
FRVVTLVLFFANASGILVAMEFALFQLFG--AVLLVTRCFIDSFMLIFGSCAMYLVHVLTTSAVILLIYVVGTILVNFVVTRKLIVVLLTVWGDSGSYLVGSVLRKYFLAHLSPNKDIEGTVGLALSMF-LY
LPVG------------VLMIVGFAFGRVGDLFESMIKRAAGVKDSIPGHGGIDRVDALLFA-VCITMDN----------
>470510548_Acanthamoeba_castellanii_str__Neff-phosphatidate_cytidylyltransferase_
LRTAFYVIS--VVN-VFMIMEFIC--SKF-----LVLLSLGSLLALAFVIILCFFLTVQERNAFFIWTISHCPYHTSP--GGSILVMLLTGWGDGAAYYVGKNFGRHKAMNVSPNKSWEGIIA-EIVFVLLG
FSVTKATL-----DIHIGLLLTSCLGIFGDAIESLIKRMGNIKDSFPGHGGIDRFDAFFVSPFHYIHVV----------
>567959396_Phytophthora_parasitica-hypothetical_protein_L915_10104_
VRLVVLVTFLLAASIVFMSTEYALTDALLSTFLLVACFLNAFTLTLHLSGMAIFLVATSRNAAFFLLVYIIGTILVAFVDTLYLLIALLVVWSDTGAYITGKALANYPLAHLSKNKDYEGTLGVGIVAVVSI
LFT-------------VAFVVAVVIGRLGDLFESLLKRAAGVKDSIPGHGGVDRIDALMFALVRYAMVY----------
>489963495_Thermoanaerobacter-MULTISPECIES__phosphatidate_cytidylyltransferase_
LRIIVGLFILKNLIALLVILEFYA--VYL--LLY---FLSKAKDVLVIIAMMLFLLFLTN-KNLYMIIYIPLFYIQKLREGMYIVWFVFVSWTDTFAYFTGRFFGKHKLATISPKKTIEGGIGISTISAVFL
FSYL------------VIILLGSIIAQCGDLIASFIKRNCYIKDFIPGHGGIDRFDSILFVPFFIQYLI----------

>585112997_Nannochloropsis_gaditana-phosphatidate_cytidylyltransferase_
YRIFCLQSPLNRFTVMGV--RISV---YI--------KFFQQLLPLGFAAVVMWFLVMRP-PSIIMCVYTGLLYWARLRCGLEVAMLYTIYWSDVGAYFGGKKFGKRKISGASPNKTVEGFLSLAMITLVGV
MWS-------------TMYLMVSVISLLGDLMASMIKRDAGVKDFFPGHGGVDRLDSYIFVPLAFTLFLG---------
>499234385_Chlorobaculum_tepidum-phosphatidate_cytidylyltransferase_
LRAIVGLLLNQLFG-LAL---------WR--HRAVVLLDYYFIYWEAILAVVMLLYVLEIQGQFLVLLYVNLSALLRLRLGEALVLLMLCVWADIFAYFGGRGFGGKRLFRISPKKTWEGYLAASALAWACY
I-CP-----------RIPALLIGVVAPAGDLLESMFKRDAGVKDSIPGHGGVDRFDTVMFVPLFLHHW-----------
>568047749_Phytophthora_parasitica-hypothetical_protein_L914_09980_
ARIAVLVTFLLAATTVFLSTEYALTGALITGLLLVVSLVYVFILTLHLMGFLVVLVFRFANAAFFVLVYITGSILVSFVDDIRLLIALLIVWSDTGAYLVGKTLANYPLAHLSKNKDYEGTVGVGIATVAAI
LFTK------------VAYIVAVVAGRLGDLFESLLKRAAGVKDSIPGHGGADRVDALMFAVVRYALHT----------
>1085009136_Toxoplasma_gondii_ME49-phosphatidate_cytidylyltransferase_
ARSSLPSFAVPLPSQLSLSAELYSASMLASRASALSFFLGVLQLLSILTSAAVFAAFLLASTTIIFLIWCVVLFWVKLRFGVRLLVASFALISDTFAYLVGSLLGSSPISSVSPRKTVQGLLGAAGLALVSL
IPERLLV------AEMVLSVLLSLVGVLGDLTASLVKRDAGVKDSLPGHGGWDRTDSYLLAPLVILFTQLLLQAD----
>224013132_Thalassiosira_pseudonana_CCMP1335-phosphatidate_cytidylyltransferase_
L-VWVY----SYFLLFLMALEYYM--IVV-----FVLAPLHIVPVVSTWAMVWFL-TMRR-ISIITIFYLGYIFWVRETNGANFIFWSWCIASDVGGYFAGRKFGKTKLSKTSPNKTVEGVIGASMILTLGI
QAV-------------PIHVMLALLGLVGDLTASMLKRDAGLKDFIPEHGGIDRVDSYIFTPYFMAYMIAALA------
>168033651_Physcomitrella_patens-predicted_protein__partial_
LRLFIIVLVVAWFIALIW--EYFL-ATRI-----ICMGGVGAVTASF---FLATVLLLQR--RFLFLFYCGYLFWVKLRCGLIATIIAITIIADTGAFLGGRALGRTPLS-VSPKKTLEGAACSSVAVVVLL
FSS-------------MVLLLVFLGSLFGDLTESMIKRDAGVKDSIPGHGGIDRVDSYIFTALSFKVGL----------
>497766501_Bifidobacterium_longum-CDP_diglyceride_synthetase_
MAAAALIVCLLDFL-LMFILDFAV--LWL--F--IAYVGRLLAAGKLASARLESSFNHEGHHRLVLVLYIPLLCVIISLTAIMLVFLPA---SDTGGLFAGAWLGKHKLSRISPKKSVEGLVGLAMAGFVFT
YA-----------RWVIVAILIGAVGTFGDLCASMLKRDIGIKDMLKGHGGVDRVDSILMSPFALWITG----------
>301108403_Phytophthora_infestans_T30_4-cytidylyltransferase_
FKEVVVGIR-SIILKQVLFVEYALRHAGITTFLLVACLVYGFTLAMYLVGMMGLLVCRFSGVAFFLLFYVSGTVLVAFVDRLLLLIALLIVWSDTGAYVTGKALEKYPLAHLSKNKDYEGTVGVGIGAAILV
LFS-------------AMFVVAVITGRIGDLFESLLKRAAGVKESIPGHGGMDRIDALMFALVRYAAGA----------
>955178649_Bodo_saltans-phosphatidate_cytidylyltransferase_like__putative_
IRVFVVVLLLLLWLTLMIHLAASVIVGFS--YFLVLLSDQQEAHQQQVDVFHILELHVIATTLFFFLVWISGLSILLAQVGVMLMVVVLSNWNDIAALIVGKSLKTRPLYKISPNKSLEGAVFLNGVTAVLI

FEKA-VW---GENGWFFLILLMGVVGVMGDLLQSLFKRTARLKDTFPGHGGVDRIDGLLIVPAWVWVAMV---------
>145543627_Paramecium_tetraurelia_strain_d4_2-hypothetical_protein__macronuclear__
WRVLIGVLAIQLLVLIVVYLEFQLSDW-------PTFVHHMISFFLLLVRLMNYLKMVEKQMTFMVFMLFVYPVLIVFQFAQALNLIWLSAWTDNGALFMGSMFGKTLFCKISPNKTWEGVSGFSVFSLILL
NTDS---------HKHLLILIVSIASIFGDLIESFIKRVADVKDSFPGHGGIDRLDSLCFSPFLYQMYMV---------
>504953658_Nostoc_sp__PCC_7524-CDP_diglyceride_synthetase_
WRIIVAALAVLWFIMLVVFLEYFL----------LPKSTNDLAAVMPIAGTVICFYLLFQKMTIVMLFYVGYLYWVRLRAGLTITVLTFCIWADIGAYIFGKFFGKTRLS-ISPKKTVEGAVFSSLAVLIGY
LLT-------------TTLLLIGLASLLGDLTESMLKRDAGVKDSIPGHGGIDRTDSYIFTPLYFTLVLQ---------
>506372003_Brevibacillus_brevis-phosphatidate_cytidylyltransferase_
MRILIGFLLMAVYLLVLLVIEFMM---FY-----LLVSLFSWLISMPPVILLLLIYSVLRNQHIVVALYIGYGYMAATRNGFMLTVMVIGIWTDSGAYFVGKAIGKRKLWEISPNKTVEGALGASVLILSIS
FDAL-------------TILVAGIIGQLGDLVESAFKRHFHVKDSIPGHGGVDRFDSFLLV-FLHLGIV----------
>499400157_Bradyrhizobium-MULTISPECIES__phosphatidate_cytidylyltransferase_
LRLLVLAILAAWWLLVLVIGEWLV---SV-----IVIFGLKAITGLVGGAIVTLIA---R----FAFAYASAAASILVRQGFAALMFVLVVWTDIGGYFAGRGIGGPKLWRVSPKKTWAGALGASLAVAFAG
L---------------LLVAVLSVVSQLGDLFESAVKRRFGVKDSIPGHGGLDRLDGFVAAAWGFRHGVL---------
>157874245_Leishmania_major_strain_Friedlin-phosphatidate_cytidylyltransferase_like_protein_
IRVCIIVLW-SSRATVFAFMEWSVPILFLELYFVVVLSRAPAAACKTRDYFLLMELHMIAPTQFFFIVWIAGIFFVYHITGVPWLSSTLGNFNDIAALVVGRSLGNQPLSAISPNKSVEGAVAINAFSAGLW
FAPANAF------NALLLLTIMGVLGVCGDLLQSLLKRAARVKDAIPGHGGIDRVDGMLLVPFCARGIMGF--------
>145540279_Paramecium_tetraurelia_strain_d4_2-hypothetical_protein__macronuclear__
WRVLIGVLTIQLLVLIVVYLEFQLSDW-------PTFVHHMISFFLLLVRLMNYLKMVEKQMTFMVFLLFVYPVLIVFQYAQALNLIWLSAWTDNGALFMGSMFGKTLFCKISPNKTWEGVSGFSVFSLILM
NTDD---------HKHLLILIVSLASIFGDLIESFIKRVADVKDSFPGHGGIDRLDSLCFSPFLYQLFMA---------
>488792978_Treponema_saccharophilum-phosphatidate_cytidylyltransferase_
IRCAIGVILVLLLI-LVCLLELHI--FVI-----LLLFGNELIYAFMGAIMVCMAWEVIVFESNMFVLYCGFFFIARMTTSRVIIAVYLMVFCDSMAWFFGNLFGNKGFIKASPNKSIAGFLGFSVCVL--H
FFGR---------VVV-LLLLTAFAAIIGDLVESVLKRSSGIKDSIPGRGGMDSLDSIVFAPILSRMIF----------
>504211222_Corallococcus_coralloides-phosphatidate_cytidylyltransferase_
LRVITLVLLLLYSILLAA--EYYI--LMA-----VMLTGAAWLSVFF---FFAWIYNLFRLAAPATFLYGSIGALSALRLGHAWVICALITWNDTLAYFAGRFLGKHKLYAVSPNKTWEGFFGVSVLGFAKF
FWCV-------------VLILGGLLGPVGDLCESMLKRAYGVKDSIPGHGGVDRIDALIFNPLVYQFVR----------
>499656941_Rhodobacter_sphaeroides-phosphatidate_cytidylyltransferase_

LRIAIMGAEVLVFLLVGLGLELAM---IL-----LAIVFEEMLLAALVLAPAAGLLGPRR-RIFY-LMVAGAG--VMLREGSVAILWLIVVVSDVMGYFAGRSLGGPKFWAVSPNKTWSGTIALAAIVL-GI
AG--------------IILPVIALAGQLGDIVESWIKRRSGVKDSIPGHGGVDRFDALTGAVLLVGMLGM---------
>145361012_Arabidopsis_thaliana-cytidinediphosphate_diacylglycerol_synthase_4_
LRIIGIVGVVAWFVALVF--EYFL-GARV-----ICMLTYFNIILVTSAAFVVAIALLVQGSRFLFLFYCGYLFWVKLRCGLVATLISFGVITDTFAFLGGKTFGRTPLT-ISPKKTWEGTIVVCIAIILLY
LLS-------------VAFFLNFFGSVFGDLTESMIKRDAGVKDSIPGHGGIDRVDSYIFTALSFKTSL----------
>42571237_Arabidopsis_thaliana-cytidinediphosphate_diacylglycerol_synthase_4_
LRIIGIVGVVAWFVALVF--EYFL-GARV-----ICMLTYFNIILVTSAAFVVAIALLVQGSRFLFLFYCGYLFWVKLRCGLVATLISFGVITDTFAFLGGKTFGRTPLT-ISPKKTWEGTIVVCIAIILLY
LLS-------------VAFFLNFFGSVFGDLTESMIKRDAGVKDSIPGHGGIDRVDSYIFTALSFKTSL----------
>813216160_Saprolegnia_parasitica_CBS_223_65-hypothetical_protein_SPRG_15879_
LRIILLVTYLLRAIYLVV-IEYALGMLLVYVALTLTSFCHYNKYGMLRWLALIFIFGNRLAISFIIFAYIVLLPIADLLIGADIALGFLVVWTDTGAYVMGHFLKKYPLAHISPNKDVEGSLGAGVGALLVL
MLPE-------VEQIHILFVVGGLLSRYGDLFASLLKRLAGVKDTIPGHGGLDRVDALLFVAVLYRGAFRAVTLVGII-
>145345606_Ostreococcus_lucimarinus_CCE9901-predicted_protein__partial_
LRVLFMGGVIAWWLAMFNASEYFMNKLNL-----LPLATGKTSIVATVISFTAICAALIVEEHFFFLIYCGFLFWVKLRGGLAATITIVSVVADVGAYAFGKNFGKNKLT-VSPNKTVEGAIGLCAGTAALV
MAQ-------------AFLVTVFFTSVFGDLLESVMKRNATMKDSIPGHGGLDRFDSYIFTSVFFIAVLF---------
>813191539_Saprolegnia_parasitica_CBS_223_65-phosphatidate_cytidylyltransferase_
LRVLVLVVYLYHAFYLFVAIEFALTNAFL-----LTCLCTGHHFVPLIEVILALVLHLPLMLGVTVYGYIVQLPMADLLLGPRIALGFLVVWADTGAYCTGHLLHRYPLAHISANKDIEGSLGLGVTGLLVL
LTDE-------QYMFRLGFVVGAAISRYGDLFASLLKRLAGVKDTIPGHGGLDRVDALLFV-AALHRIAL-MVLLPIS-
>493321774_Cylindrospermopsis_raciborskii-phosphatidate_cytidylyltransferase_
WRIIIAAVCTLWFIAIIIFFEYFL--MAA-----ISLICFQSLDAIMIAGTFICFYLLFQQLTIIMLFYVGYLYWVRLRNGFTFTILTFCIWADIGAYIFGKYFGRTPLS-ISPKKTVEGAIFVSLVLLWGF
LFT-------------LTLLLIGIASLLGDLTESLLKRDAGVKDSIPGHGGIDRTDSYIFTPLYFTLIL----------
>159465793_Chlamydomonas_reinhardtii-phosphatidate_cytidylyltransferase_
-----------LTL--FVYQEYFF-QSVA-------MSHYRKITLLAVASFALLVLQVVAKRKFLFLFYCGWLFWLKLRNGLVAVLTAVVIIADTGAYFVGKSLGRTKLT-ISPKKTVEGAAGAAVATLGSL
FPA-------------VVLVMVFFSSLFGDLIESIMKRDAGMKDSIPGHGGLDRFDSYIFSAVFFVFVLYSITGL----
>15232383_Arabidopsis_thaliana-cytidinediphosphate_diacylglycerol_synthase_5_
FRTLGIVGIVAWFVAVVL--EYFL-GARV-----ICMLTYFHIISITSAAFVVAMALLLQGNRFLFLFYCGYLFWVKLRCGLVAILISFGIISDTFAFLGGKAFGRTPLI-ISPKKTWEGAFAVCISIILLS
LLS-------------IAFVLNFFGSVFGDLTESMIKRDAGVKDSIPGHGGIDRVDSYIFTALSFRLHG----------

>219122283_Phaeodactylum_tricornutum_CCAP_1055_1-predicted_protein_
LRVLIRLAVVWLLCDHAIQANISPLFALMLEYYRVNRFTSLEILPIFGLYAMIWFLTMKR-VTIITMFYLGYVFWVRIRIGSIFIFWTWCLASDVGAYFVGRRYGNTKLGATSPNKTVEGVLGASGLLVFGA
QGT-------------AVHILLGLIGLIGDLTASMIKRDAGVKDFIPDHGGIDRVDSFIWSPYLVNSVIA---------
>499188751_Rickettsia_prowazekii-phosphatidate_cytidylyltransferase_
IRIIALFVAILLYL-MLVLGEWYM--------------------------------------TYIILIIIPIPLLIFLSMNRLVIMLYFILWVDTFAMIGGKTFKGIKLAKISPKKTWTGLITASVLVLIPH
ISKI------------FIICILALIAQSSDLFISYFKRKFNIKDSIPGHGGVDRFDSIILTPVCIIYL-----------
>148370463_Brucella_ovis_ATCC_25840-phosphatidate_cytidylyltransferase_
LRIIVLTLLTVVFLFSAILAEWTLTASLI--ILL---ILRGLLIGFLVAGCAILLVTQWK----RALFYAGFSSLSLLRGGFTTIVFLFVVWTDIAAYFNGRALGGPKLARFSPNKTWSGAIGAAVAGLLVL
VWVP-------------VLLLLSIVSQIGDLAESWVKRQFGAKDSLPGHGGVDRVDGLVAAALLFAIFAA---------
>123962980_Prochlorococcus_marinus_str__MIT_9303-Phosphatidate_cytidylyltransferase_
SRSLAAGLVVLWFVGLVIHLEFFM--IVA-----LLTLPDLAALPLSGAAICGWLL--LQITSIIFLFYLGFLHWLRLRNGMAITLVACMIVSDIGSYELGRRFGRLPLS-ISPGKTVEGAVGFAMTIAVAL
LFL-------------VLLVLVALFALVGDLIESMMKRDAGLKDSLPGHGGIDRIDSYLFTAVYATLIL----------
>731421276_Vitis_vinifera-PREDICTED__phosphatidate_cytidylyltransferase_4__chloroplastic_
FRVLGIVGVVAWFAALAAFIEYFL-GTRV-----ICMLTYFNIVSVTSAAFFVAMALLLQGNRFLFLFYCGYLFWVKLRCGLVVTLISISIIADTYAFLGGKAFGRTPLT-ISPKKTWEGVIAGCIATVILI
FLS-------------IAFFLNFFGSLFGDLTESMIKRDAGVKDSIPGHGGIDRADSYIFTALSFKTFL----------
>50951391_Leifsonia_xyli_subsp__xyli_str__CTCB07-phosphatidate_cytidylyltransferase_
LALLVVVVLIILLFDVMAFAELTA--RVI-----VPFLVLAGVLVI-VCRLAEEAFAGAAGGLVLFQMYVSLLYAILLLAGEWWVLGFVLVVVDTGAYVSGLTWGRHPMATISPKKTWEGFAGVAVAAVLVF
MW--------------IVFLVMLLTATSGDLAESLVKRDLGIKDMLPGHGGFDRLDSILPSAAGLLIFS----------

Supplementary Data S2. The edited multiple sequence alignment for the phylogenetic tree reconstruction of LCAT

>28273394_monocots_Oryza_sativa_putative_lecithin_cholesterol_acyl_transferase_
PVILIPGAGGNQLEAYDSVADDYYMNVLASTLYEEGFDLFGCAANGGRPA
ILVAHSLGGLAWRAANVKRLVAPWGGSVQEML-TFASLIRDE-----VE
>33318329_eudicots_Nicotiana_tabacum_phospholipase_A1_
PVLLVSGLAGSILHSYNPKTGYTHFHDMIDMLYKKGTTLFGYKASGGRKV
DIISHSMGGLDVFSKYVNKWITPFQGAPGCINDSLSRWTMHQPFNLSIL
>47208625_bony_fishes_Tetraodon_nigroviridis_unnamed_protein_product__partial_
---VVPGDLGNQLEAYNKTTHTTYFFTIVQALYTRGDDVRGASKAGGP-V
VLIAHSMGNMAWKDKYIKAFIPPWAGVAKTLR-VLSLKIRAQ-----WM
>61889061_even-toed_ungulates_Bos_taurus_group_XV_phospholipase_A2_precursor_
----VPGDMGNQLEAYNQTSHTTYLHTMVESLYERGKDVRGYQLYGGP-V
VLVAHSMGNMDWKDKYIRAFVPPWGGVPKTLR-VLSLKIRAQ-----WS
>115460534_monocots_Oryza_sativa_Os04g0615100_
PVLLVPGIGGSILEAFDASTGKTYYHDMIVQMYQEGKTLFGYIASGEKKI
NLITHSMGGLDVFEKYIKSWIAPFQGAPGYITTSLSKWSMQQPLDMDIL
>123424158_trichomonads_Trichomonas_vaginalis_Lecithin_cholesterol_acyltransferase_family_protein_
PILLLPGIYGSNLFAYYPETDTISFAPMLEYLYTVKKDLFGYEKNDRKAV
VVLGYSCGGLKWKDKYIHKVIPAFGGSSNTID-VAKNDILRQ-----AR
>123437297_trichomonads_Trichomonas_vaginalis_Lecithin_cholesterol_acyltransferase_family_protein_
PIILVPGTMGSNLVAYDPTINDAYMHEYIKYLYTVGQDLFGYVKNDNQKV
VLVGHSLGGYDWRAKYIESALPSFGGAGTVVE-QLNKDIMAK-----PE
>123445579_trichomonads_Trichomonas_vaginalis_Lecithin_cholesterol_acyltransferase_family_protein_
PVILVPGIGASNIYVYDESTQCSYYKKIIQYFYVEGIDLYGYRKTGQK-V
VFLCHSFGTFDWVNKYVDHCVPSFAGAGKAVR---IEWTDED-----VK
>154414526_trichomonads_Trichomonas_vaginalis_Lecithin_cholesterol_acyltransferase_family_protein_

```
PVILLPGIYGSNLYSYDAATDKVSFGPLIDYFYTIKKNLFGYSSNGNQNV
VVLGYSCGGLAWKDKYIHKVIPAFAGSSETLD-VVKNDVLRE-----GE
>154418604_trichomonads_Trichomonas_vaginalis_Lecithin_cholesterol_acyltransferase_family_protein_
PIILVPPMFGSELMGWNYTSGLPELIYVIEKLYVEGVDLFGYAQTGNNKV
ALYGISGGGNDWKDKYIRQVLPSYGGSGEALS-VLNTQNFRD-----LQ
>154418606_trichomonads_Trichomonas_vaginalis_Lecithin_cholesterol_acyltransferase_family_protein_
-------MFGSELMGWNYTSGLPDLIYVINKLYIEGLDLFGYSQTGNQKV
ALYGISGGGNEWKDKYLRQVLPSYGGAGEALN-VLNTQTFRN-----HQ
>159108850_diplomonads_Giardialamblia
YASALTKIIEDVVPAYDNTSLTMSSFQLVNDHQHTGNPIFSTIKEDSDFL
TFYKETSGGFMLMDELAAEYEINYNGRVKSKK-CIKHLSKGVDRASEIM
>159110153_diplomonads_Giardia_lamblia_Lecithin_cholesterol_acyl_transferase__putative_
PIILIPGLCSTKLDIPTSDGRYQYFTTLKNRLYQVDVDLFCLNRTSSLHV
ILIGHSHGALDWH-QHIFRFIPPYDNSSAYMAMSLQASSSVPLGLWEWE
>159112067_diplomonads_Giardia_lamblia_Lecithin_cholesterol_acyl_transferase__putative_
PIILIPGVGGSKLDAPDPETQLYYFVTIIGRLYQPNKNLFGRELNNGMPV
NIIAHSLGGLDWM-THIRRFITPFDGSSMTLNSFINLPPQYEKRYSEIV
>159116494_diplomonads_Giardia_lamblia_Hypothetical_protein_GL50803_12116_
PIILVPGVCGSLLVAYGSRDSVTYYETFAVYLYKEGLNLFACQTTGQR-C
IVVGHSMGGLDWN-DYIAKFVVPYAGSSGLIAAPYASFKSYMPGLRSII
>168028153_mosses_Physcomitrella_patens_predicted_protein_
PVLLVPGIGGSILTAYDPVTGKTYFHDLIEQLYKEGKTLFGCEASRGRKV
DIITHSMGGLQVFQKYANSWIAPFEGAPGFIMDCLAKWSMHQPLNKVIV
>168060499_mosses_Physcomitrella_patens_predicted_protein_
PVLLVPGIGGSILNAYDPVTGKTYFHDLIEQLYEEGKTLFGYEVSGGKKV
DIITHSMGGIEVFERYVNSWIAPFQGAPGFIMDCLAKWSMHQPLNEDIL
```

>209150029_bony_fishes_Salmo_salar_1_O_acylceramide_synthase_precursor_
PVVLIPGDLGNQLEAYNRTTRQTYFVTIVQSLYTRDDDVRGAEKAGGP-V
VLIAHSMGNMTWKDRYIKAFVAPWAGVAKTMK-VVSLKIRSQ-----WE
>222618895_monocots_Oryza_sativa_hypothetical_protein_OsJ_02688_
AVVLVSGMGGSVLHAYNPDTGYVHFHDMIDMLYKKGTTLFGYKASGGKKV
NIISHSMGGLDIFAKYVNKWICPFQGAPGCINDSLSRWVMHQPFNMSVF
>225423706_eudicots_Vitis_vinifera_PREDICTED__lecithine_cholesterol_acyltransferase_like_4_
PVLLVPGIAGSILKAFDPSTGQTYFHDMIVEMFQEGKTLFGYTASGGKKI
NIISHSMGGLDIFEKYVQNWIAPFQGAPGYISSTFSKWSMHQPFNLEIL
>242045288_monocots_Sorghum_bicolor_hypothetical_protein_SORBIDRAFT_02g029710_
PVLLVSGMGGSVLNAYNADTGYVHFHDMIDMLYEKGTTLFGYKTSGGKKV
NLISHSMGGLDVFTKYVNKWICPFQGAPGCINDSLSRWAMHQPFNMSVF
>242074318_monocots_Sorghum_bicolor_hypothetical_protein_SORBIDRAFT_06g028470_
PVLLVPGIAGSILEAFDASTGKTYYHDMIVEMYQEGKTLFGYTSSGGKKI
NLITHSMGGLDVFEKYVKSWIAPFQGAPGYITTSLSKWCMQKPLNLDIL
>255538016_eudicots_Ricinus_communis_1_O_acylceramide_synthase_precursor__putative_
PLIIVPGSGGNQLEAYDQDLDDYYMAPLVESLYIDGESLFGSNLNGRKPV
IILSHSLGGLSWRQKFVRHFIAPWGGSVEVML-TLPLLVREE-----VY
>255547492_eudicots_Ricinus_communis_phosphatidylcholine_sterol_O_acyltransferase__putative_
PVLLVSGIGGCILNSYNPKTGYTHFHDMIDMLYKKGTTLFGYEASGQRKV
NIISHSMGGLDVVSKFVNKWICPFQGAPGCINDSLSRWTMHQPFNFHIL
>290978346_eukaryotes_Naegleria_gruberi_predicted_protein_
PIILIPGYAASMLSVSSSIVGDVYFHELIEYLYEEGVTLFAKTLTKANKV
NLISHSMGGYELY-KSVNVHITPWQGTRDWIASSLGSIAHYE----QRL
>297800066_eudicots_Arabidopsis_lyrata_lecithin_cholesterol_acyltransferase_family_protein_
PVLLVPGIAGSILNAFDPSTGKTYFHEMIVEMFEEGKTLFGYKASGEKKI

NVISHSMGGLDIFEKYVQNWIAPFRGAPGYITSTLSKWSMHQPFNWKIM
>302754442_vascular_plants_Selaginella_moellendorffii_hypothetical_protein_SELMODRAFT_63043__partial_
PLLLVPGIGGSILNAYDPATGKTNFHDLIEQLYKAGTTLFGHKTSGGKKV
NIISHSMGGLASFERLVNSWTTPFQGAPAFVTDCLAKWSTHQPFNRCIL
>302766207_vascular_plants_Selaginella_moellendorffii_hypothetical_protein_SELMODRAFT_85956_
PVILVPGAGGNQLEVYNGGSKKFYMNSLVAALYESQKTLFGSRSNKNTPV
TLISHSLGGLTWKKRFIHRFIAPWGGSVQEMR-VFPLVLRDE-----YE
>302798869_vascular_plants_Selaginella_moellendorffii_hypothetical_protein_SELMODRAFT_113667_
PVLLVPGIGGSILNAFDPKTGETYFHDLIEKMYEQ---VFGYKHAGGKKV
KIVSHSMGGLEFFEKHVDTWIAPWQGAPGFVTDCLAKWSTHQPFNYDIL
>313238782_tunicates_Oikopleura_dioica_unnamed_protein_product_
PTVLVPGILGSRVEAFDNVTKTTYFHDIVQAFYEVNNTLKAFAKFNKK-V
VIVAHSMGNPAWKKKYVKVYAPVYLGAPKSLK-SLGLIQMRS-----YF
>326516384_monocots_Hordeum_vulgare_predicted_protein_
PVILIPGSGGNQLEAYDRDADDYYMDVLATTLYEEGRDLFGCAANDGRPA
ILMAHSLGGLAWRAAHVKRLVAPWGGSVQEML-TFPSLIRDE-----VE
>356521572_eudicots_Glycine_max_PREDICTED__lecithin_cholesterol_acyltransferase_like_4_like_
PVLLVPGVGGSMLHAYDPSTGKTYFHDMIVEMFEEGKTLFGYNAAGGKKI
NIITHSMGGLDIFEKYVKNWVAPFQGAPGTIYSTFSKWSMHQPFNLEIL
>356568525_eudicots_Glycine_max_PREDICTED__phospholipase_A_1__LCAT3_like_
PVLLVSGMGGSIVNSYNPQTGYTHFHDMIDMLYNKGTTLFGHKASGGRKV
NLISHSMGGIDVFTKYVNKWICPFQGAPGCINDSLKRWTMHQPFNFDIL
>357475183_eudicots_Medicago_truncatula_Group_XV_phospholipase_A2_
PVLLVPGVGGSILNAYDPSTGKTYFHDMIVQMYQEGKTLFGYNAAGGKKI
DLISHSMGGLDIFEKYVKNWIAPFQGAPGCTNSTFSKWSMHQPFNSHIF
>357507845_eudicots_Medicago_truncatula_Phospholipase_A1_

PVLLVSGMGGSILHSYNPETGYTHFHDMIDMLYVKGTTLFGYKASGGRKV
NIISHSMGGVDVFSKYVNKWICPFQGAPGCINDALSRWTFHQPFNLAIL
>359476598_eudicots_Vitis_vinifera_PREDICTED__phospholipase_A_1__LCAT3_like_
PVLLISGIGGSILHSYNPKTGYTYFHDMINMLYKKGITLFGYKASGGRKV
NIISHSMGGLDVFSKYVNKWICPFQGAPGCINGSLLRWTMHQPFNFSIL
>390366325_sea_urchins_Strongylocentrotus_purpuratus_PREDICTED__group_XV_phospholipase_A2_like_
PVVLIPGDGGCQLQAYDPATRTSYYTHLVDALYERNVNIRGYQKNGHEPV
VLVSHSLGCLSWKNRFIRAWVGPYAGTTKVMR-VVALTARNA-----LD
>443692514_segmented_worms_Capitella_teleta_hypothetical_protein_CAPTEDRAFT_214081_
PIIIIPGKGGSQLEVYNTSSGESYAAYLVQELYERGVNLLGYLRNGRRRV
LLVSHSMGGLDWKRSHIKGLVTPWDGAMVVAQ-LHRNIIRDQ-----KS
>449441554_eudicots_Cucumis_sativus_PREDICTED__lecithin_cholesterol_acyltransferase_like_1_like_
PLILIPGAGGNQLEAYDKDSDDYYMAGLVNSLYVRDKTLFGSNSNGGKSV
ILVTHSLGGLSWRRHFIKHLVTPWGGSVEGMR-TFPLRVRTE-----VY
>449455413_eudicots_Cucumis_sativus_PREDICTED__lecithine_cholesterol_acyltransferase_like_4_like_
PVLLVPGVGGSILNAYDPTSGESYYHDMIVEMFQEGKTLFGYNASGGKKI
NLISHSMGGLKIFEKYVQNWIAPFQGAPGYVTSTFSKWSMHQPFNLDIL
>449472754_birds_Taeniopygia_guttata_PREDICTED__group_XV_phospholipase_A2_
PAAGVPGDLGNQLEAYNRTSKITYFYMLVQSLYKRDEDVRGYEQYGSP-V
VLIAHSMGNMDWKDKYIKDYVAPWGGVAKTLR-VLSLKIRDQ-----WL
>470115120_eudicots_Fragaria_vesca_PREDICTED__lecithine_cholesterol_acyltransferase_like_4_like_
PVLLVPGIAGSVLNAFDPATGKTYFHDMIVEFYQEGKTLFGYNASGGKKI
NIISHSMGGLDVFEKYVKNWIAPFQGAPGYVTSTFSKWSFHQPYNMEIL
>470138157_eudicots_Fragaria_vesca_PREDICTED__phospholipase_A_1__LCAT3_like_
PVLLISGIGGSILTCYNPLTGYTYFHDMINMLYKKGTTLFGYKASGGRKV
NIISHSMGGLEVFSKYVSKWICPFQGAPGCINDSLSRWTMHQPFNFAIS

>470511210_eukaryotes_Acanthamoeba_castellanii_phospholipase_A1__putative_
PLLLLLSVGATGAAVYNETTMLTYFGAMIQALHVPGRTLFGFNASGGQKV
HLVAHSMGNLQHASRYVASWTAPFLGAGAVGLETVSKELDHAPFGWTQL
>475579927_monocots_Aegilops_tauschii_Group_XV_phospholipase_A2_
-FCMLERFACVMIRSYCDGAGKTYYHDMIVQMYQEGKTLFGYTASGGKKI
NLITHSMGGLDVFEKYVKSWVAPFQGAPGYINSGLSKWTMQQPLNEDIL
>492394624_firmicutes_Brevibacillus_agri_esterase_
PEEIELRSREVIFELEQNELGRSEAHELPADFLHANTDVDYEKPKNANEQ
IIMNVNPGSYNSYKLNVAAKTILIPGIGGSRLVAENDPKHRR-YNFSIP
>502132747_eudicots_Cicer_arietinum_PREDICTED__lecithin_cholesterol_acyltransferase_like_1_like_
PLILIPGNGGNQLEAYDQELDDYYMASLVDSLYIDGKNLFGSNSNGGKPV
ILVSHSLGGLSWRKKFIKHFITPWGGTIDEMF-TFPLLVRNE-----VY
>507623397_rodents_Octodon_degus_PREDICTED__group_XV_phospholipase_A2_
----VPGDLGNQLEAYNRTSGTTYFHTMVDSLYTRGEDVRGYHLYGGP-V
VLVAHSMGNMDWKNKYIRAFVAPWGGVAKTLA-LLSLKIREQ-----WF
>512834221_frogs__toads_Xenopus_(Silurana)_PREDICTED__group_XV_phospholipase_A2_isoform_X3_
PVVLVPGDLGNQLEAYNKTSKTTYFYTLVQSLYTRDENVRGFESYQSP-V
VLVAHSMGNLDWKDKYIHSFVAPWGGVSKTLH-VLSLRIRDQ-----WE
>527203320_eudicots_Genlisea_aurea_hypothetical_protein_M569_04944__partial_
PVLLVSGVCGSILHSYNPQTGYTHFHDMITMLYKKGTTLFGYEASGGRKV
NIISHSMGGIEVFSKYVNKWITPFQGAPGYVNDSISRWSMHQPFNHDIL
>527206771_eudicots_Genlisea_aurea_lecithine_cholesterol_acyltransferase_like_protein__partial_
PVLLVPGIAGSILNAFDPATGKTYFHDMIVEFYQEGTTLFGHAASGGKKI
NIISHSMGGVDTFQKYVVKNWIAPFQGAPGYITSTLKKWSMHQPFNLDIL
>530592773_turtles_Chrysemys_picta_PREDICTED__group_XV_phospholipase_A2_
PVVLIPGDLGNQLEAYNRTSKITYFYTLVQSLYQRDKDVRGYEQNGGP-V

VLIAHSMGNMDWKDKYIKDYVAPWGGVAKTLR-VLSLKIRDQ-----WF
>551633925_cryptomonads_Guillardia_theta_hypothetical_protein_GUITHDRAFT_120982_
------------MSDFNASSMKLYFDAMIQELWIHGISLFGRERNNGRKV
ALVSHSMGALEFFQEAVETWIAPHQGASAKIFMEFGAEAAKVPFNEDCW
>552847477_green_algae_Chlorella_variabilis_hypothetical_protein_CHLNCDRAFT_133423_
PLLLVPGVCGTQLAVYSKDSGLVYFLPLINFLYSPGIDLFGSRRCGGRRV
DLVTHSMGGLAEFEALVGRWVCPFGGAPGYAVDGL-------PFDPQLW
>557025633_coelacanths_Latimeria_chalumnae_PREDICTED__group_XV_phospholipase_A2_like_
PVVLVPGDLGNQLEVYNQTSKSTYFATLVSSMYTRDKDVRGAEQYGEP-V
VLLAHSMGNVQWKDKYIKSYLPPWGGVAKTLR-VLSLKIRTQ-----WL
>557267539_vertebrates_Alligator_sinensis_PREDICTED__group_XV_phospholipase_A2_
DGEIVPGDLGNQLEAYNRTNKITYFYTLVQSLYQRDEDVRGYEQYGSP-V
VLIAHSMGNMEWKDKHIKDYVAPWGGVAKTLR-VLPLKIRDQ-----WL
>558594671_diplomonads_Spironucleus_salmonicida_hypothetical_protein_SS50377_18736_
PILLIPGVCGSKLVANNENFEYVYFGPMIQYLYQLGVNLFAKKLTSSK-I
QIIAHSMGGVKTWYEDISKFIVPFDGCGGLSLEGISAACLHQINVTKTA
>558596676_diplomonads_Spironucleus_salmonicida_Lecithin_cholesterol_acyltransferase_family_protein_
PIVLIHGIFGTVLKSLAGRTNQDCFDLLIDKLYVKNSDLQAK-----TPP
IIIAHSMGALDQINSLISGFIAPFDGATGTTLLLPGNSGMSLPKLTNFK
>558597565_diplomonads_Spironucleus_salmonicida_Lecithin_cholesterol_acyltransferase_
PIIIIPGIFGSRLVNWGRTTADGCFYDISTSLYRENIDLFASLP------
ILITHSIGSLANWSKIFASQIGTFDGANGGGLMAVGFWCNSGECYESLS
>558600899_diplomonads_Spironucleus_salmonicida_Lecithin_cholesterol_acyltransferase_family_protein_
PLIIIPGIFGNILRNLGGYIDKEYFDILIKKLYVEGKSLFIP-----KSP
IILAHSMGGLNWYKQFQK-VIVPFDGINGLGLLVPAQSGMTAPYILPCR
>558602410_diplomonads_Spironucleus_salmonicida_Lecithin_cholesterol_acyltransferase_

PVILVPGVAGSKLWSTENGVFKSYFDYYVDYFYTPGVNLFAMSQNNNQKV
SLIAHSMGGLDWW-KFIRRFCVPFDGSSGGVTACFSKCPGIAILTKNLV
>565357416_eudicots_Solanum_tuberosum_PREDICTED__lecithin_cholesterol_acyltransferase_like_1_like_
PLILIPGAGGNQLEAYQTQLDDYYMEPLVKALYRDGENLFGSSSNGGKPV
ILVSHSLGGLSWSQKYIKHFITPWGGTVMQIL-TFPLLVREE-----VL
>565393861_eudicots_Solanum_tuberosum_PREDICTED__lecithin_cholesterol_acyltransferase_like_4_like_
PVLLVPGVAGSILNAFDPSTGKTYYHDMIVEMYQEGKTLFGHTASGGKKI
NIISHSMGGLDIFEKYVKNWIAPFQGAPGYITSSLSKWSMHQPFNLELL
>566198123_eudicots_Populus_trichocarpa_hypothetical_protein_POPTR_0012s12220g_
PVLLVPGIAGSILKAFDPQTGRSYFHDMIVEMFQEGKTLFGYKASGGKKI
NIISHSMGGLDIFEKYVKNWIAPFRGAPGFVTSTFSKWSMHQPFNFDIL
>567203687_eudicots_Eutrema_salsugineum_hypothetical_protein_EUTSA_v10020730mg_
PVLLVSGVGGSILHSYNPKTGYTHFHDMIEMLYKKGTTLFGYKRSGERKV
TIISHSMGGIEVFSKYVNKWITPFQGAPGCISDSLSRWTMHQPFNFSIL
>568836597_eudicots_Citrus_sinensis_PREDICTED__lecithin_cholesterol_acyltransferase_like_1_like_
PLILVPGNGGNQLEAYDPDLDDFYMASLVESLYVTGETLFGSNSNGGKPV
ILVSHSLGGLPWRKKFIKHFVAPWGGTVEEML-TFPLDVRQE-----VH
>568853727_eudicots_Citrus_sinensis_PREDICTED__phospholipase_A_1__LCAT3_like_isoform_X1_
PVLLVSGMGGSVLHAYNPKTGYTHFHDMIEMLYKKGTTLFGYKASGNRKV
TLITHSMGGLDVFSKFVNKWISPFQGAPGCINDSLSRWTMHQPFNFAIL
>573911430_bony_fishes_Lepisosteus_oculatus_PREDICTED__group_XV_phospholipase_A2_like_
-MEKVPGDLGNQLEAYNRTSNRTYFYTIVQSLYTRDYDVRGAEHFGGP-V
VLIAHSMGNMAWKDRYIKAYVPPWGGVAKTLR-VVPLKIRAQ-----WR
>586756854_flowering_plants_Amborella_trichopoda_hypothetical_protein_AMTR_s00032p00177900_
PVLLVPGIAGSILTAFDPSSGKTYFHDMIEEMFQEGETLFGYKCSEGKKV
TIISHSMGGLDVFEKYVKNWIAPFQGAPGYITDTLSKWSMHQPFNREIF

```
>586779857_flowering_plants_Amborella_trichopoda_hypothetical_protein_AMTR_s00066p00036210_
PVLLVSGIGGSILNAFNPKTGYTHFHDMIDMLYEKGTTLFGYKASGGKKV
NIISHSMGGLDVFEMYVNSWICPFQGAPGCINDSLSRWTMHQPFNFSIL
>587937975_eudicots_Morus_notabilis_Lecithine_cholesterol_acyltransferase_like_4_
PVLLVPGIAGSILKAFDPASGRTYFHDMIMEMFQEGKTLFGYKASGGKKI
NIITHSMGGLDVFEKYVKNWIAPFQGAPGYITSTLSKWSMHQPFNLEIL
>590596874_eudicots_Theobroma_cacao_Alpha_beta_Hydrolases_superfamily_protein_
PLILVPGSGGNQLEAYDRDLDDYYMAQLIESLYVSGETLFGSASNGGKPV
ILLSHSLGGLSWRQRFIKHFVAPWGGTVQEML-TFPLLVRGE-----VI
>602665629_snakes_Python_bivittatus_PREDICTED__group_XV_phospholipase_A2_like_
CIITVPGDLGNQLEAYNRTSGSTYFYTLVQNLYQRDENIRGYEEYGEP-V
VLIAHSMGNMEWKDKYIRDFVAPWGGVAKAFR-VLSFKIRDQ-----WL
>604321025_eudicots_Erythranthe_guttata_hypothetical_protein_MIMGU_mgv1a004228mg_
PVLFVPGIAGSILNAFDPATGRSYFHDMIVEFYQEGTTLFGYTASGGKKI
NIISHSMGGLDIFEKYVKNWIAPFRGAPGYVTSAFSKWSMQQPFNIEIL
>604335585_eudicots_Erythranthe_guttata_hypothetical_protein_MIMGU_mgv1a007059mg_
PVVLISGIAGSILNSYNPDTGYT-------MLYKKGTTLFGYKASGGRKV
NIISHSMGGLEIFSRYVNKWITPFQGAPGCINDSISRWSMHQPFNVAIL
>604335846_eudicots_Erythranthe_guttata_hypothetical_protein_MIMGU_mgv1a0085831mg__partial_
-----------------------HFHDMIDMLYEKGTTLFGYKASGGRKV
NIISHSMGGLDIFTKYVNKWITPFQGAPGCINDSISRWSMHQPFNFEIL
>604345043_eudicots_Erythranthe_guttata_hypothetical_protein_MIMGU_mgv1a004105mg_
PVLLVPGIAGSILNSFDPATGSTYFHDMIVEFYKEGTTLFGYTASGGRKI
NIISHSMGGLEIFEKYVKSWIAPFRGAPGYITSAFSKWSMQQPFNTDIL
>617421572_bony_fishes_Poecilia_formosa_PREDICTED__group_XV_phospholipase_A2_like_
PVVLIPGDLGNQLEAYNNITHTSYFFTIVQAMYTRDDDVRGAEKAGRR-V
```

VLIAHSMGNLAWKDKYIQTFIPPWAGVAKTLR-VVPLKIRSQ-----WL
>629108256_eudicots_Eucalyptus_grandis_hypothetical_protein_EUGRSUZ_E01878_
PVLLVPGVAGSILHAFDPAVGKTYFHDMIVEMFQEGKTLFGYNAAGGKKM
TIISHSMGGLDIFAKYVKNWIAPFQGAPGYVTSTFSKWSMHQPFNQEIL
>629120114_eudicots_Eucalyptus_grandis_hypothetical_protein_EUGRSUZ_B01436_
PVLLVSGMGGSILHSFNPSTGLTHFHDMIDMLYKKGTTLFGYKASGGRKV
DIISHSMGGLDVFSKYVRKWICPFQGAPGCVNDSLSRWTMHQPHR-GLV
>632978216_chimaeras_Callorhinchus_milii_PREDICTED__group_XV_phospholipase_A2_
PVVLVPGDLGNQLEAYNATTKTTYFFTLVESMYTRDEDVRGYGQYGGP-V
VLIAHSMGNLAWKDKYIKTFVAPWGGVSKTLR-VLTLKIRTQ-----WK
>637317326_lizards_Anolis_carolinensis_PREDICTED__group_XV_phospholipase_A2_
-VVLVPGDLGNQLEAYNKTSGATYFYTMVQHLYQRDEDIRGYEEYGEP-V
VLIAHSMGNMDWKDKYIRDFVAPWGGVAKTLR-VLSLKIRDQ-----WR
>657960606_eudicots_Malus_domestica_PREDICTED__LOW_QUALITY_PROTEIN__lecithin_cholesterol_acyltransferase_like_4_
PVLLVPGIAGSVLNAFDPSTGKTYFHDMIVELFQEGTTLFGYNASGGKKI
TIITHSMGGLDVFEKYVNKWIAPFQGAPGYVTSTFSKWSMHQPFNMEIL
>661891941_eudicots_Coffea_canephora_unnamed_protein_product_
PVLLVSGIAGSILHSYNPETGYVHFHDMIDMLYKKGTTLFGYEASGGRKV
NLISHSMGGLDVFTKYVNKWITPFQGAPGCVNDSLSRWSMHQPFNFSIL
>661895457_eudicots_Coffea_canephora_unnamed_protein_product_
PLILVPGTGGNQLEAYDPYLDDYYMEPLARSLYVDGENLFGSSSNGGKPV
ILLSHSLGGLSWRQKYVKHFIAPWGGTVQEMI-TFPLIVRNE-----VK
>664718858_odd-toed_ungulates_Equus_przewalskii_PREDICTED__group_XV_phospholipase_A2_isoform_X1_
PHLKVPGDLGNQLEAYNRTSRATYFHTMVESLYTRDKDIRGHQQYGGP-V
VLVAHSMGNMAWKDKYIHAFVAPWGGVAKTLR-VLTLKIREQ-----WL
>669311063_nematodes_Trichuris_suis_hypothetical_protein_M513_04683_

PVVIVPGDGGNQLEAYDEATGTTYFADMVNAMYERGKTVRGYYSNGNKKV
VTIGHSLGNLSWKEKFIKSHIAPYGGSMKIVK-AFPLTLRAE-----WA
>670368187_monocots_Zea_mays_PREDICTED__LOW_QUALITY_PROTEIN__lecithin_cholesterol_acyltransferase_like_1_
PVVLIPGAGGNQLEAYDADADDYYMNTLASTLYEEGRDLFGCAASGGRPA
ILVAHSLGGLQWRAAHVRRLVTPWGGSVQEML-TFPSLIRDE-----VE
>672133274_monocots_Phoenix_dactylifera_PREDICTED__lecithin_cholesterol_acyltransferase_like_1_
PLVLIPGSGGNQLEAYNRDIDDYYMATLVDALYEDGYNLFGSAANGGQPV
ILLSHSLGSLTWRQKYIKHLVAPWAGTVQEML-TFPLLVRDE-----II
>672160230_monocots_Phoenix_dactylifera_PREDICTED__lecithin_cholesterol_acyltransferase_like_4_
PVLLVPGIGGSILNAFDPSTGKTYYHDMIEEMFQEGETLFGYTSTGGKKL
IIISHSMGGLDIFEKYVKSWIAPFQGAPGYIATTLSKWSMQQPLNLEIL
>672163444_monocots_Phoenix_dactylifera_PREDICTED__lecithin_cholesterol_acyltransferase_like_4_
PVLLVPGIAGSILNAFDPSTGKTYYHDMIEEMYQEGKTLFGYTSSGEKKL
NIITHSMGGLDIFEKYVNSWIAPFQGAPGYITSSLSKWSMQQSFNLEIL
>675355554_green_algae_Auxenochlorella_protothecoides_Lecithin_cholesterol_acyltransferase_like_4_
PLLLIPGVCGTILNTYDRSSGRVYFDTFVEYLYTCGTDLFGHALTG-RRA
DILSHSMGALAEFEKLVETWIAPFGGAPGFGSDALDRGTFRQPFNPDVW
>647565225_firmicutes_Brevibacillus_agri_esterase_
PEEIELRNREVIFELEQNELGRSEAHELPADFLHANTDVDYEKPRNANEQ
IIMNVNPGSYNSYKLNVAAKTILIPGIGGSRLVAENDPKHRR-YNFSIP
>651511060_firmicutes_Bacillus_sp._esterase_
PEEIELRSREVIFELEQNELGRSEAHELPADFLHANTDVDYEKPKNANEQ
IIMNVNPGSYNSYKLNVAAKTILIPGIGGSRLVAENDPKHRR-YNFSIP

Supplementary Data S3. The edited multiple sequence alignment for the phylogenetic tree reconstruction of PSS

>126542379_euryarchaeotes_Methanohalophilus_portucalensis-phosphatidylserine_synthase_

LIMTLNAVGLLIFFIGFFVLVVADGMDGYLARGMGLDSLADAVSFGVAPACLIIAAFFMYLVILRLARFNTFGLPTASAVVLYALLGLVVLLCYLMISDHPKL-MGAVSVLFFSTILSYFIFSTILFLLEM

>159115027_diplomonads_Giardia_lamblia-Hypothetical_protein_GL50803_17427_

YCVSINLLGLLIHVSVLCIMMLFDLFDGRVAETNGFDDVADATSFGIAVGLLISWSTLVYIFFYRLVRFILFGLPPATATMALCVLTSVLVLCLLTISSVPHLVFRISVEALFVVSAIYGLILYCLGSLFY

>406887084_bacteria_uncultured_bacterium-hypothetical_protein_ACD_75C02524G0004_

YILSTNFFGVALIHFAIAIVVLFDLFDGRMAITFGLDDIADFVSFGLSPAYVIDFAFFVFFLAYRLVRFVVFGLPPAGALIVAALCIVAALSVGLMVSHVRHFVLFFLASAVIIVSIAFIMFGYLILAMIK

>406895406_bacteria_uncultured_bacterium-hypothetical_protein_ACD_75C00220G0002_

YILSSNFCGVALIHFAIAIIVLFDLFDGRMAITYGLDDIADFVSFGLSPAYVIGFALFIFFLAYRLVRFVTFGLPPAGALIVAALCSFAALSVGLMISHIRHFVLFFLTSAFIIIFIAFIMFGYLILLMIW

>489238919_firmicutes_Gemella_haemolysans-CDP_diacylglycerol__serine_O_phosphatidyltransferase_

IYLTLNIFGFMILSFGTFIILLADRYDGIVARSIGLDSLCDVVSFGVAPAMLVFSAIIVYVCAYRLARFNVYGVPTTCGTIL--------------IAPIKSIL---------------------ILCLML

>491574368_b-proteobacteria_Sutterella_wadsworthensis-CDP_diacylglycerol__serine_O_phosphatidyltransferase_

LAFTAALFAYYIIMFAAAVFFLFDGMDGRVARSFGMDSLADAVSFGIAPALIVAIGAVVYALLLRLARFNCFGLPPAAAALVFVWLVVTLYAGLTMVSNAPSGYFWVIIVCAVLFIIISSLSIFILFCALV

>493469949_d-proteobacteria_delta_proteobacterium-CDP_alcohol_phosphatidyltransferase_

YILSLNLFGITIVLYATAILAIFDLFDGRMAETVGLDDIADLVSFGVCPGLLIGSFILLYTLGFRLWRYLVFGLPPAGAMGAACLFLVVCLTALLLVSHVRHFVLIVMFGFVVVFIIAYLMLGALLMILIH

>493706386_e-proteobacteria_Helicobacter_canadensis-CDP_diacylglycerol__serine_O_phosphatidyltransferase_

LFFTGSIYGILISTFACLVLLIFDGLDGRVARSFGFDSLADIVAFGVAPAMILVLGIVLYVVAIRLARFNVFGLPPAAAVFVWLLLIGSLIVALLMVSNIRSFLSKIIVLLMIILAILFALSITILITVLS

>494660454_verrucomicrobia_Pedosphaera_parvula-CDP_diacylglycerol__serine_O_phosphatidyltransferase_

LLLTANLTGFVLTVIALYILLIFDLFDGRVARSFGFDSLADLISFGAAPAFLVVIGFIIYLIAFRLARFNCFGFPPSAAGMVLTYFVLMIFLSCMMVSEVKTFLFTKTLVTIIFIVAVLIWALPILFTLII

>495375454_verrucomicrobia_Verrucomicrobiae_bacterium-CDP_diacylglycerol__serine_O_phosphatidyltransferase_

LLFTANLFGFLIKIFAVFILGACDALDGRVARSFGFDSIADIVTFGLAPAMMVIAGFLIYLLAVRLSRFNVFGLPPAAAGMIIVLVFLMLLISYLMISNVRSFIFTTFVAVLVVGGLIYFYTLVLGFIIFV

>495610399_e-proteobacteria_Sulfurimonas_gotlandica-CDP_diacylglycerol__serine_O_phosphatidyltransferase_

LLFTASIFGVFIIVFAALIMLIFDGLDGRVARSFGFDSLADMVAFGVAPALLMVFGVAIFVIAIRLARFNVFGVPPTAAVFVMVLLLLSILVSILMVSNIRSFIMKFFVIIISALFVVFMEGFTLIFVILV

>495612079_e-proteobacteria_Sulfurimonas_gotlandica-phosphatidylserine_synthase_

LLFTFNITGLLTYTFAILAWIAFDIFDGKIARNFGLDSFADFLSFVLVPVFLIVSGVVYYVILRRLIHFNIFGVPPLGAILL----WLVYLT------------ILPAIAVIVLMAII----------SLL

>495859098_CFB_group_bacteria_Niabella_soli-CDP_alcohol_phosphatidyltransferase_

ILFTLNLVGCVIKLMGGLLLAVVDFLDGFVARSMGLDSLADAISFGVAPGMLLLSELPIFPCVWRLAKFNLFGIPPAAGLTVLPAIAVILIISGLMVSNLPSLRVDKLLLVLISVIAAIIAAVPLVFVVII
>495986473_bacteria_Synergistaceae-CDP-diacylglycerol-serine-O-phosphatidyltransferase
IMITSSVFGVSLIAFAALICFFFDVMDGRVARSFGLDSLADAISFGVAPAFLIYWGLTFFALVLRLARFNVFGLPPAGGLALIVIASAMCFVGALMVSSVPNALAKLYGLMTFIALCFFIKAFLAMAVIIV
>496352778_verrucomicrobia_Methylacidiphilum_fumariolicum-CDP_diacylglycerol__serine_O_phosphatidyltransferase_
LLLTANLIGFLILLYSLFILAVFDVLDGRLARSFGFDSLADLISFGVAPALLVVIGIVIYLVALRLARFNVFGFPPAAAGLVITLLVLLLFLSIMMFSKNLSFIVPKFLFVVCILGLTIVWMLAVDFLLLF
>497988185_firmicutes_Kurthia_sp_-CDP_diacylglycerol__serine_O_phosphatidyltransferase_
VFLTINFMGMLVIIMAVFIFLLFDLFDGRVARSFGLDSLADVVTFGVAPAMIASFGVAVYAVLVRLARFNVFGMPPAAAIILFTFTLATFLLAALMVSQVRNFL-------------------------LSN
>499451090_e-proteobacteria_Wolinella_succinogenes-CDP_diacylglycerol__serine_O_phosphatidyltransferase_
LLFTGSVYGILMAFFACLIMLIFDGLDGRVARSFGFDSLADVVAFGVAPAMVLLFGMVLYVVAIRLARFNVFGLPPSAAVFVWILMAMTLGVAILMVSNVRSFMKKAIVLLMMLLALVYLEGITILITILG
>499661848_d-proteobacteria_Pelobacter_carbinolicus-CDP_diacylglycerol__glycerol_3_phosphate_3_phosphatidyltransferase_
YILSINLVGLACWLPALLVFLFLDLFDGRAAETQGFDDVADGTSFGLTVGLIVFIGLIAYLAIYRLIRFVVFGLPPAGALMASCVLIIVIITALLMISRVSHFAVKVLVLGGFLFMLALGAPLLISFVLIF
>500473686_d-proteobacteria_Geobacter_uraniireducens-CDP_diacylglycerol__serine_O_phosphatidyltransferase_
LLFTASLFGFYMVLYAAWILAIFDGLDGKVARSFGFDSLADLVSFGAAPGLLMALGLALFVAALRLARFNVFGLPPAAASMVTVLLLLIYFLALLMVSNVRSFPFAFLVLAVILLIVIAAIMLFVIFTIFI
>501031641_e-proteobacteria_Sulfurovum_sp_-CDP_diacylglycerol__serine_O_phosphatidyltransferase_
LLFTASIFGVLIVSFASLILLVFDGLDGRVARSFGFDSLADVISFGIAPAMLLTFGLVLYVIAIRLARFNIFGLPPTAAIFIWILLFLALGVAILMVSNFRSFIFKTMIALVLIISLLYLEGFALIILVLI
>501126602_d-proteobacteria_Desulfococcus_oleovorans-CDP_alcohol_phosphatidyltransferase_
VVLSGNLLGLFMYYISLFLLMLFDGFDGAAARTWGSDDIADGVNYGIAPGVALIEGVTIYGFISRLVYFTLFGVPPIGGLIVSLILLLGLSVGIACVQMVSYYVILGLPLYAMALLGAFVALLLVIALGFY
>502808847_verrucomicrobia_Coraliomargarita_akajimensis-CDP_diacylglycerol__serine_O_phosphatidyltransferase_
LSLTANLFGFLILIYAVFILFICDALDGRVARSFGFDSIADIISFGVAPTLMMVLSLIVYLLGVRLARFNVFGLPPAAAGMILVLVPFMLAIAFLMISNIQSFIFRTFILLILGLGTAFILFFAILFFIAI
>503257869_high_GC_Gram+_Intrasporangium_calvum-CDP_diacylglycerol__serine_O_phosphatidyltransferase_
LLFTLNMVGFSVLFFAALIAAVLDISDGAVARTFGFDSLADLVSFGIAPAVLVAWATGFWRAAYRLARFNVFGLPPGAAGVVTVFAAAGVLPAALMATSYRSFLLQTFGLLALVLVGLVLVIAYGYVLPLT
>503709921_chlamydias_Simkania_negevensis-CDP_diacylglycerol__serine_O_phosphatidyltransferase_
IIITAGLSGLFVFNLSALLLLFADLLDGAVARSFGFDSLADTISFGVAPSVLFLFAVGLYTIVLRLVRFNVFGLPPAAAAGAVNLFCIMIVIGYLMICKWKSLLHLILFTVILAIFILYGYLPIVLVIIVV
>503716922_a-proteobacteria_Candidatus_Midichloria-CDP_diacylglycerol__serine_O_phosphatidyltransferase_
IIITMALCGTTIRLFAALVIVIMDGLDGRVARSFGLDSLADLVSFGVAPGMVLSVGAVFYITAFRLARFNVFGVPPAAAGLVPLVIVYVALIGMMMISQLPSGVVPLLVLTGVMMAGILIFILPLMGVILI
>504008964_d-proteobacteria_Halobacteriovorax_marinus-putative_CDP_diacylglycerol__serine_O_phosphatidyltransferase_protein_

LTFTANMAGFAIIWFASILLLIFDSVDGRVARSFGFDSISDVVSFGMAPAFLVFLGVVLYLLALRLARFNAFGLPPTAALGLYVLLFYVVLYSLLMISNIPSFAKRVLAIIFLLLALIFTLMIGVITGVLV
>505467392_e-proteobacteria_uncultured_Sulfuricurvum-CDP_diacylglycerol__serine_O_phosphatidyltransferase_
LLFTASIFGFYIALFAAFIFLIFDGLDGRVARSFGFDSLADIVAFGVAPALLLVTGVVLFIIAVRLARFNVFGLPPTAAIMIAVILPLAVVLSILMVSNIRSFMMRFLVGLIVVALGVFIEALAILAALML
>505824619_spirochetes_Treponema_azotonutricium-phosphatidylserine_synthase_
LLFTCNMTGLLIMIFAAALVLFCDILDGRIARSFGLDSLSDLVSFGIAPAMLIVIGAIFFVLAMRLARFNVFGLPPASAGVIFVLSIVMVVLSFLMVSNIPSFMVQLLVFVIVLAILIIVNIIFIIFSALA
>506386592_d-proteobacteria_Desulfobacterium_autotrophicum-phosphatidylserine_synthase_
YILSGNFCGVTLVHFAIIIIILFDLFDGRMALTYGLDDIADFVSFGMAPAYVIG-ALILFILGFRLVRFILFGLPPAGALIVASLISMTLVSVGLMVSHIRHFVLFFMISASIIVTLAFIMFGYLILGMAF
>517800680_verrucomicrobia_Rubritalea_marina-hypothetical_protein_
LLMTANLCGFFVLFYAILIFGIFDLLDGRLARSFGFDSIADVVSFGMAPALLVVLGAIIYLLAMRLARFNCFGIPPMAAGFIITFLATMLGLSALMLSNVRSFVLWGVVIGAIFFMLLLNYTPTLIFTLIA
>522073996_d-proteobacteria_Desulfobacter_curvatus-phosphatidylserine_synthase_
YILSMNFMGVTLIYYAIVIIMLFDLFDGRMALTYGLDDIADFVSFGLAPAYMVGFAFFIYVVAYRLVRFVTFGFPPAGALIVASLIFFTAVSTALMVSNIRHLVIFFLISAGIIVVLSYIMFGYLILALTI
>522167099_d-proteobacteria_Geopsychrobacter_electrodiphilus-hypothetical_protein_
YILSINLVGLACWLPALLVFLFLDLFDGRAAETKGFDDVADGTSFGLTAGLIVFVGLLLYAGVYRLIRFVVFGMPPAGALLVTCVLIIVVTAALLMISRIPHFAVRVLALGAFLFLLALGAPLLIAFILVL
>524687065_verrucomicrobia_Akkermansia_sp_-cDP_diacylglycerol_serine_O_phosphatidyltransferase_
LLFTANLVGFFILFYATLIFALFDLFDGRIARGFGFDSLADIVSFGIAPALLVVVGGILYLLALRLARFNCFGLPPMAAGAVTMYLLAMAGVSVLMMSRVVSFIMYAIVLIVLTVICIFKVMPAVIFSLLV
>544667896_a-proteobacteria_Litoreibacter_arenae-CDP_diacylglycerol__serine_O_phosphatidyltransferase_
VLVTVALCGLTLRIIALLIMLFLDAADGKIARSFGLDTLADFFNFGIAPGLLIFLGLSVLAIALRLARFNVFGVPPALASLAPVFLIYEIGIALLAISTIPSIAPMIAVGCAILVICLMVHTFIFANLLVL
>548224029_b-proteobacteria_Sutterellaceae_bacterium-CDP_diacylglycerol__serine_O_phosphatidyltransferase_
LLFTTSLFAFYIVMFASAIFFIFDGCDGRVARSFGYDSIADMTAFGVAPALVIAIGAAIYCLGLRLARFNTFGLPPAAAALIFVWLVLAVFAGLTMVCNAPSGLFWFLFVAAILFIIISSLSIFIIFCAVV
>550913684_d-proteobacteria_Desulfospira_joergensenii-phosphatidylserine_synthase_
YILSSNFCGVILVQFAIIIIMLFDLFDGRMALTYGMDDIADFVSFGLAPAYVVGFSLVVFILAFRLARFVRFGLPPAGALLVAALVIHTLVSVGLMVSHVRHFVVFFLISASIIITLAYLMFGYLILGMFA
>551333204_g-proteobacteria_Leucothrix_mucor-CDP_diacylglycerol__serine_O_phosphatidyltransferase_
LLITTALFGFYIVMFAAAVFVVLDGADGRVARSFGYDSLSDLVSFGVAPAVVIALGMAIYVAALRLARFNVFGLPPASAAIMMVWFILTISAGLLMVSNVLSFFYLAILAVVLAFALAAIKVLFAAFLALV
>558601611_diplomonads_Spironucleus_salmonicida-Transmembrane_domain_containing_protein_
YIISLNMLGIIIYIQVLYVLAFFDVFDGRAADTYGFDDAADFTSFGVATGVLIFVSISFYISAFRLVRFVIFGLPPATSAFVLTVGYLGLIICILTVSKIKHFVAMGMCGFALISAVSQCGIVGFGIVIFL
>583000071_high_GC_Gram+_Actinokineospora_spheciospongiae-CDP_diacylglycerol__serine_O_phosphatidyltransferase_

LAITVAMCGLSVQLLAIAIAVVLDSLDGRIARSMGLDSLADAISFGVAPALVLRVGVVIFAVVLRLARFNTFGVPPAGGLLAPMILVWTILMAALLISRIPSVVAPLLVGVGLLAAVVITVALAVLLGLAY

>589394119_bacteria_Cloacibacillus_evryensis-CDP_diacylglycerol__serine_O_phosphatidyltransferase_

IMITSSVFGVSLIAFAALICFFFDVMDGRVARSFGLDSLADAISFGVAPAFLIYWGLTFFALVLRLARFNVFGLPPAGGLALIVIASAMCFVGALMVSSVPNALAKLYGLMTFIALCFFIKAFLAMAVIIV

>636809040_d-proteobacteria_Desulfotignum_balticum-phosphatidylserine_synthase_

YILSLNFFGLALVYFAVIIIMLFDLFDGRMAETYGLDDIADFVSFGIAPAYMLGWAVFIYITAFRLIRFLFFGLPPAGALLLAILLPMVLLTTGLMVSTLRHLLLFYTISAGIIVIIAFVLFGGVILGMVV

>640150783_high_GC_Gram+_Nocardia_sp_-CDP_diacylglycerol__serine_O_phosphatidyltransferase_

LIVTIALCGLSVKLLAVMIGAVLDTLDGRLARTIGLDSLADAISFGVAPALVLFAGIILYAVVLRLARFNTFGVPPAAALIAPLTLAWTIFAALLAVSTIPAMVALLLVLTALAAAALVTILLMVLIALGF

>640167859_verrucomicrobia_Verrucomicrobia_bacterium-CDP_diacylglycerol__serine_O_phosphatidyltransferase_

LLMTANLLGFLILLFSLFILAVFDLLDGRVARSFGFDSLADLISFGVAPALLVVLGIVIYLVALRLARFNVFGFPPAAAGLVLTLFVLLLLLSFMMFSKLRSFVLPKFLLVLATVALTVVWMLAVDFLFLV

>644476068_d-proteobacteria_Deferrisoma_camini-CDP_diacylglycerol__glycerol_3_phosphate_3_phosphatidyltransferase_

YILSLNLVGLGIWVPAFLVFLFLDLFDGRAAETRGFDDVADGTSFGVTVGLIVFLGGLVHLAVYRLIRFVVFGLPPGGALLVGCLLAVVLASAALMVSRVSHFALRVGFLALFLLLLAQGAPLAISFAVAF

>653365730_high-GC-Gram-Rhodococcus-CDP-diacylglycerol-serine-O-phosphatidyltransferase

LAVTVAMCGLSVKLLSLMIGAVFDALDGRIARSMGLDSLADAISFGVAPALVLLGGVIIYAVVLRLARFNTFGVPPAGALVTPLAATWVVLSALLIVSRIPAFVTLLLILVGLAGAALITVLLMVLIVLLF

>654643182_b-proteobacteria_Pseudoduganella_violaceinigra-CDP_diacylglycerol__serine_O_phosphatidyltransferase_

LAFTTALFGFFIVMFAAAIFVLLDGLDGRVARSFGYDSLSDMVSFGAAPALVIALGIAVYCAALRLARFNTFGLPPAAASLIFVMLVLAAFAGLSMVSNVPSFIFLAVFLIALFLALISLKVLFGLFVGVV

>655137435_d-proteobacteria_Desulfobulbus_mediterraneus-phosphatidylserine_synthase_

YILSTNFFGLTLYHFAIVIIVLFDLFDGRMAETYGLDDIADFVSFGLAPAYVIGMAFFIFFVAYRLVRFVVFGLPPAGALLVASLTVITSLSVGLMVSHLRHFVLFFLASATLVVTIAYVMFGVLILCMVK

>670501746_d-proteobacteria_Desulfobacula_sp_-phosphatidylserine_synthase_

YILSSNFCGVTLVQFAIIIIVLFDLFDGRMALTYGMDDIADFVSFGLAPAFVVGLAFVLFIIAFRLIRFVRFGLPPAGALMVASLVGMTCVSVGLMVSHIRHFVVFFMISASIIVTLSFIMFGYLILGMVK

>737871262_a-proteobacteria_Defluviimonas_sp_-CDP_diacylglycerol__serine_O_phosphatidyltransferase_

LLITVAICGLSIRLYAVLIIVVFDGLDGRLARSLGLDSLADFLNFGVAPGILIAQGVAIYVVVLRLARFNVFGVPPAGAFLAPIFAIYIVAVGMLMISRIPSLLRLFIVGFVAVLAALLSISLTVLDFL-L

>740752397_a-proteobacteria_endosymbiont_of-hypothetical_protein_

FMITIGLCGISVRIWAALIIVFMDGLDGKIARSFGLDSLADIVSFGVAPSIVISVGAVFYITALRLARFNVFGIPPAAAALAPMISAYMFVIGILMVSSVPSFITLMLVSVGLLIASMVLITLPIVGLLLL

>746642567_e-proteobacteria_Campylobacter_sp_-phosphatidylserine_synthase_

LLFTASIFGIIVIIFALYIILICDGLDGRVARSFGFDSLADLIAFGVAPAILFIFGLILFVVAIRLARFNVFGLPPTAAVVSWVSACIQILLAFLMVSNIRSFILKVLILLVILFSMLYLESALIVASVCI

>754860402_firmicutes_Oceanobacillus_manasiensis-hypothetical_protein_

ISVTLNMYGFLIGVYATLIIIILDALDGRLARSLGLDSLADIVTFGIAPALLFNLDSLLFVLGYRLARFNSFGVPTAAGGILTTLFLIIALLSYAMVSTIRSLIMATILIGIVLL-----VALY-LTAFMY
>754998192_firmicutes_Bacillus_massiliogorillae-CDP_diacylglycerol__serine_O_phosphatidyltransferase_
ILFTFNLFGFLLHVIATLIFIMLDAVDGRIARNLGLDSLADVVSFGVAPAYMVYWGALLFPLAFRLARFNLFGIPTMAGGVVFILLILFYVLAYLMVSKIKSLVITSIFMIYMFI-LLWRILFYVSLALAL
>755782576-high-GC-Gram_Rhodococcus-CDP-diacylglycerol-serine-O-phosphatidyltransferase
LAVTVAMCGLSVKLLTLMIGAVFDALDGRIARSMGLDSLADAISFGVAPALVLLGGVVIYAVVLRLARFNTFGVPPAGALVVPLAVTWTVFSALLAVSRIPAMVAILLVLVGLAGAALITVLLIVLIALAF
>759898726_verrucomicrobia_Diplosphaera_colitermitum-CDP_diacylglycerol__serine_O_phosphatidyltransferase_
LLMTANIFGFGIIVYAVLIFGAFDMLDGRLARSFGFDSLADVVSFGLAPALMMIIGLIIYLLAIRLARFNVFGLPPAAAGTVLVLFFLLLLIAILMVSTVRSGILITFLLFGMVTAAVIMIGMLVCCLIFG
>783330736_bacteria_Candidatus_Magnetobacterium-CDP_diacylglycerol__serine_O_phosphatidyltransferase_
LALTLGMCGVYILMYAGAILIIFDGLDGWVARSFGLDSLSDLVAFGVAPSVLVAIGAVLYVCALRLARYNVFGMPPAAATILFVIFIMTVVLSIFMISTLRGLLFPFLVLLIIIITIITMVMLFVFGFMFV
>806772592_firmicutes_Streptococcus_pneumoniae-phosphatidylserine_synthase_
IMITINFVGLLIHFMAVFIITVFDLFDGMVARSIGLDSFADLVTFGVAPSILAAIGICAYSIMLRLARFNTFGMPPFAAMCLLCRLFGTSVLAYLMVSKIKHFD------------------------LDL
>814316014_firmicutes_Streptococcus_pneumoniae-phosphatidylserine_synthase_
IMITINFVGLLIHFMAVFIITVFDLFDGMVARSIGLDSFADLVTFGVAPSILAAIGICAYSIMLRLARFNTFGMPPFAAMCLLCFLFGTCVLAYLMVSKIKHFD---------------------------
>851232262_euryarchaeotes_Methanosarcina_barkeri-archaetidylserine_synthase_
LLVSLNLIGISIAAFALLLLIIADGADGYIARGLGLDSLADAVSFGVAPALLIFVGVFFYAIVLRLARFNSFGLPTAGCVMLYMLLALTLCLSILMVSSVNKIVIASIFGITMLLYLYNIILPFILMLIFF
>851383053_euryarchaeotes_Methanococcoides_methylutens-archaetidylserine_synthase_
IFMTLNALGMMIFCDAFLILFIADGMDGFLARSVGLDSLADAISFGVAPACALYLMTVFYLFILRLARFNTFGLPTAAAVVMYLLIGIMVLLSLLMVSEYPKLPVALVFALPIISYFVFPTVMFLLLFLEM
>871952701_chlamydias_Estrella_lausannensis-putative_CDP_diacylglycerol__serine_O_phosphatidyltransferase_PssA_
LIITAGLAGLFIFSLIAILILFADLLDGAIARSFGFDSLADAISFGVAPAVVVLLIFAVFTVVLRLIRFNIFGLPPAACGACANLYFVLTFLGYMMISRFRSLLKIVFLSILLTLLLFFGTFFAVSWLVVL
>881117640_firmicutes_Staphylococcus_pasteuri-CDP_diacylglycerol__serine_O_phosphatidyltransferase_
IYLTLNIFGFMILSFGTFIILLADRYDGIVARSIGLDSLCDVVSFGVAPAMLVFSAIIVYVCAYRLARFNVYGVPTTCGTIL--------------IAPIKSIL---------------------ILCLML
>884874903_firmicutes_Streptococcus_pneumoniae-phosphatidylserine_synthase_
IMITINFVGLLIHFMAVFIITVFDLFDGMVARSIGLDSFADLVTFGVAPSILAAIGICAYSIMLRLARFNTFGMPPFAAMCLLCLLFGTCVLAYLMVSKIKHFD--------------------------S
>895741407_firmicutes_Streptococcus_pneumoniae-phosphatidylserine_synthase_
IMITINFVGLLIHFMAAFIITVFDLFDGMVARSIGLDSFADLVTFGVAPSILAAIGICAYSVMLRLARFNTFGMPPFAAICLLCLLFGTCVLAYLMVSKIKHFD--------------------------S
>910222105_verrucomicrobia_Verrucomicrobium_sp_-CDP_diacylglycerol__serine_O_phosphatidyltransferase_

LLMTANLLGFLSLVYAILILLFFDLLDGRLARSFGFDSLADVVSFGVAPALLMVLGLIIYLVAMRLARFNCFGCPPVAAGVILTLFVLMLILSYLMVSNVESFVFHWVLVSILVLFFTVRWMPMVLFVLAV
>910223894_verrucomicrobia_Verrucomicrobium_sp_-CDP_diacylglycerol__serine_O_phosphatidyltransferase_
--MTANLLGFMILVYAIFILVLFDLLDGRLARSFGFDSLADIVSFGMAPALLVVLGLIIYLVAMRLARFNCFGCPPAAAGVILTMTALMLLLSYLMVSSLESFIFHWVLISIIVLAFTVKWMPTVLFVLLV
>918413202_d-proteobacteria_Geoalkalibacter_subterraneus-CDP_diacylglycerol__glycerol_3_phosphate_3_phosphatidyltransferase_
YILSINLIGLACWLPALLVFLFLDLFDGRAAETRGFDDVADGTSFGFTVGLIVFLGLIIYLAVYRLVRFVRFGLPPAAALLASCILATVIIASALMVSRVPHFTVRVLVLATFLVLLAQGAPLVMAYALAF
>918701063_bacteria_Endomicrobium_proavitum-hypothetical_protein_
LLFTCNMTGFLMLVFAALLICACDMLDGRVARSFGLDSLSDLISFGLAPAIMIGIGAILFVLALRLAKFNVFGLPPASAGLLFVLSVVMVVLSLLMVSNIPSFMLQVLVIIIVLLVIVIVNTFFILFSVLV
>924871252_d-proteobacteria_Desulfuromonas_sp_-CDP_diacylglycerol__glycerol_3_phosphate_3_phosphatidyltransferase_
YILSLNLVGLACWLPALLVFLFLDLFDGRAAETQGFDDVADGTSFGLTAGLIVFVGLLLYVGVYRLIRFVVFGLPPAGALAVTCVLIIVVATALLMISRIPHFTVRVLALGAFLFMLALGAPLLISFVLIF
>930065542_a-proteobacteria_alpha_proteobacterium-CDP_diacylglycerol_O_phosphatidyltransferase_
LMLTMNLAGMTIRMWAVAIAVLFDMLDGRIARSFGLDSLSDVVSFGVAPAMILSVGMALMGVALRLARFNTFGVPPAGAFLAPLIAPWTVAVAGLMISAVPSMLVFVLLGVGATAAALVSMTLSVVGLIIF

Supplementary Data S4. The edited multiple sequence alignment for the phylogenetic tree reconstruction of Psd
>470511034_Acanthamoeba_castellanii_str__Neff-phosphatidylserine_decarboxylase_
SKIMTNGKAIFIRDEMVFNFNEFFYRKLKARPLIAVSPADSMHFIQIWIKGFTLTSLLQSLVIARLAPDYHRFHMPVLTPIGTYFTVNPVAVNTVYNKRVVCPGVTFVAIGATMVGSIFPQTIKGDEHGYFF
GSTVLVLFELVKVGIG
>1133456972_Amphimedon_queenslandica-PREDICTED__phosphatidylserine_decarboxylase_proenzyme__mitochondrial_like_isoform_X1_
LLPLSRGTFWFVLHEALYNFRSLFKRRLKLRPIGLVSPVDGVMVLIEQVKGYSLKAFLGHTAVLYLSPDYHHFHSPALRHFGELVTVSPWAVKHLLNERVLLSGFSLTAVGALNVGSIILTNLRGDHIGGFI
GSTVVLIFEIVKYGIG
>340373369_Amphimedon_queenslandica-PREDICTED__phosphatidylserine_decarboxylase_proenzyme__mitochondrial_like_
GLPLSRGLLLYSLEESFYSFSDLFTRNLKSRSFELVCPCDGVLFVIEQVKGYSLSAFLGYHVVIYLGPDNHHFYSPTLRHIGELLTVAPWAAKTLFNERVILAGFSFAAVGAYNVGSILLTNAKGYHLGHFM
GSSIVLVFEVVKIGLG
>501045297_Anaeromyxobacter_sp__Fw109_5-phosphatidylserine_decarboxylase_
ALPVSRGLAMFALSECLYTFGEFFARPLRLRPIVIVSPVDGVSTALVQAKGYPTSALVAAYATIYLSPDYHRIHFPLIRYVGQLWPVNPASVRTLFNERLVTVGCAVVAVGATVVGRVAVVTIKGGELGAFM
GSTVILLFELLRVGIG
>42566885_Arabidopsis_thaliana-phosphatidylserine_decarboxylase_1_
FLPISRGFIYWALEEALYSLQDFFVRSLKCRPICLVSPVDGVLFKIEQVKGYSVPALLGYYCVIYLKPDYHRIHSPAARHFGRLFPVNERATRTLYNERVVLEGMALAAVGATNIGSILLTNLKGKEVAVFM
GSTVVLIFQVVRVGLG
>186513660_Arabidopsis_thaliana-phosphatidylserine_decarboxylase_3_
ALQLSEGKEIFLMAEVLFTFNEFFVRELKARPIVAVSAADCLMFVRFWIKGFSIKGLLGSLVIFRLAPDYHRFHSPVIVNVGSLYTVNPIAVNSVFNKRTIVIGVAFVAIGATMVGSIFEDHVKGDELGYFF
GSTVICVFELVTVGLG
>240256448_Arabidopsis_thaliana-phosphatidylserine_decarboxylase_2_
ALQLSEGKEIFLMAEVLFTFNEFFIRELKARPIVAVCAADCLMFVRFWIKGFSIRGLLGSLVIFRLAPDYHRFHVPVIVDVGSLYTVNPIAVNSVFNKRTVAIGVAFVAIGATMVGSIFEEHVKGDELGYFF
GSTVICVFELVSVGLG
>598008341_Auricularia_subglabra_TFB_10046_SS5-phosphatidylserine_decarboxylase_
---------------MLYSLGEFFYRSLRVRPIALVSPADGVLFEVEQVKGYSLDALLGFFCVIYLAPDYHRFHSPTVRHFGELFSVSPYVAARLFNERVALLGFSMIPVGATNVGSIILTNLPGEEVGGFL
GSTIVLVFEIVQYGLG
>598033293_Auricularia_subglabra_TFB_10046_SS5-hypothetical_protein_AURDEDRAFT_113883_

ALKLSIGKEIFIRDEILFTFNEFFYRKLMARPVTLVSGADCMMFVKFWIKGFTVARLLGALAIFRLAPDYHRFHSPVITYIGEYYTVNPQAIRTVYNARKIVPGVMAVCIGAMMVGSITEQSIRGEEFGYFF
GSTIVLLFELVRVGVG
>598045457_Auricularia_subglabra_TFB_10046_SS5-hypothetical_protein_AURDEDRAFT_187226_
ILVQSIGI-IFILEQLIYTINEFFSRRLKLRPIVIVSAADCLTFVQFWVKGFTIPNLLASLAIFRLAPDYHRFHSPVFVHTTDLYTVNPQAVNEVFNKRDVVVGVAFVAIGALLVGAIWQDEVKGDNIGWFY
GSTCICVFPLVKVGIG
>409927057_Bacillus_azotoformans_LMG_9581-phosphatidylserine_decarboxylase_
LTNISSRYIIFIESEIIYSLHDFFIRTLKARQITVISPVDGVELINILVKGFSIRKMLGVFMIIYLSPDYHRIHSPVVWDLGKSYPVNKWGLKYPLNYRKISEGVVLAKVGALFINSVLF--LKGEEIGYFF
GSTVILLFEGIKMGIG
>855304293_Blastocystis_hominis-Phosphatidylserine_decarboxylase_
ALLGSRGLVYVSVTEFFYTPNSFFTRSLKARPILLLSPVDGVSLHLRQVKGYSLRSFLSYSAVLYLSPDYHHIHAPAIVHVGRLLPVLEAYAAHLFNERVVLAGFALGMVGAYNVGSIIVTNAQGERVGTFL
GSTVVLAFEVVRVGIG
>855308734_Blastocystis_hominis-uncharacterized_protein_
AFMTSRGLIYRPQSKLHRCISLSFIHTIT-RLVVILSPATAFLFFVHQVKGYSMSTFLGFTVTLYLSPDYHRVHSPAIAHFGKLLPVKNFFAQNLFNERVVLTGFSLGLVGAYNVGSILLTNSPGDPVGMFL
GSTVVLAFEVVKMGLG
>955169409_Bodo_saltans-phosphatidylserine_decarboxylase__putative__partial_
TVPMSRGFRILIISEVLYTLQEFFSRRLRARPPPLVSPCDAVLVTILQVKGYAMSTLMTIYFLFHLRPDYHRFHSPAVAHIGTLHPVTYASSKWLFNERVSLMGLAFVPVGATCVGSILITN---------K
TSSVQNIF--------
>955163648_Bodo_saltans-phosphatidylserine_decarboxylase__putative_
LVPLSTAIVIYSLTEIVYTLDEFFVRTLRVRPIVLVSPVDGVLHAVVQVKGYTLKNLFIKHVAFILRADYHHVHTPCVAYVGAMLPLSLRGFRWIFNERVCLPGVWMALVGGTLRGKIALTNFRGDDIARFW
GSAVVLVADVVKVGIM
>955161887_Bodo_saltans-phosphatidylserine_decarboxylase__putative_
MFPFTTGMVYWIMSQFYYTVDQWFTRAINLRPLAVISPADCLLFSSFFLKGVTISTLLGAVAVSRLAPDYHRFHAPVVTRIGTYWSVNADATQSVFARTVVVIGVAVVGIGATCVGSVLISEVRGEPIGSMF
GSTVLVLFSLVNVNIG
>499401904_Bradyrhizobium-MULTISPECIES__phosphatidylserine_decarboxylase_
LIPLTRGFNIWKLSEA-FSLHDCFTRELKLRPFIVASPSDGVGHILFQVKGYSLLDLLGSFVTLRLTSMYHRFHAPYITLIGDVWNVNPIALKRLFNERAVIREVTLVPVAAILVASILLAQVKGEELGWFH
GSTIIILAPIIRAGLL

>515112144_Bacillales-MULTISPECIES__phosphatidylserine_decarboxylase_
LLPMSRGIFIYITSIIAYTLKEFFSRRLKARPITIVSPVDGVSLILIQAKGFSVSELLGKFITIYLSPDYHRIHMPVLCYLGRLYPVNKLGIENLFNERLVTHGMALVKVGALFVGSVVATNYKGSELGWFF
GSTVILLLEVLLMGLA
>71980843_Caenorhabditis_elegans-Phosphatidylserine_decarboxylase_beta_chain_
VLPASRGLILFAMDDCFYSFAAFFNRKLKTRPIPLVSPADGVLFVIEYVKGYDVDKFLGYQVVIYLAPDYHAFHSPAARHVGLLLSVRPTLLSHLFNERVVLNGFSMSAVAATNVGDIVLTNYSGERVGEFL
GSTIVLVFQILRYGLV
>71980840_Caenorhabditis_elegans-Phosphatidylserine_decarboxylase_beta_chain_
VLPASRGLILFAMDDCFYSFAAFFNRKLKTRPIPLVSPADGVLFVIEYVKGYDVDKFLGYQVVIYLAPDYHAFHSPAARHVGLLLSVRPTLLSHLFNERVVLNGFSMSAVAATNVGDIVLTNYSGERVGEFL
GSTIVLVFQILRYGLV
>50291089_Candida_glabrata_CBS_138-hypothetical_protein_
CLPISRGVLFYSLDEMLYNLSDFFYRNIRTRPVVIVCPSDGVLINVEQVKGYSIKEFLGYFAVIYLAPDYHHFHSPICRHFGDLFSVAPYFQRNLFNERVALLGFSMTPVGATNVGSILLTNLKGEEMGGFL
GSTVVLCFEIVKMGLG
>50290391_Candida_glabrata_CBS_138-hypothetical_protein_
FLKLSVGKLIFIMSQC-YTFNEFFYRKLKSRPPVMVSAADSCTYIEIWIKGFSLNRLTGSIAIFRLAPDYHRIHCPVVIFIGEYYTVNPMAVRSVFNVRVIVPGLLYIAVGAMMVGSILEDFKRGDEMGYFF
GSTVILVMQLVKVGIG
>470293729_Capsaspora_owczarzaki_ATCC_30864-phosphatidylserine_decarboxylase_proenzyme_
LLPLSRGFLYYALDEMLYNLSAFFMRHLVARPILMVSPADGVLFVIEQVKGYSLQHFLGFHIIIYLAPDYHRFHSPAAKHFGELLSVAPAVAEILFNERVVLLGFSFSAVGAYNVGSIILTNVKGEEIGTFL
GSTLILIFEVLQVGLG
>754343957_Capsaspora_owczarzaki_ATCC_30864-C2_domain_containing_protein_
SKMLTVGKLIFIMSEFAYTFNEFFYRQLVARPLIALSPADASNFIDLWIKGFTLPTLLDSLAIFRLAPDYHRFHIPVIYDIGEYMTVNPMAIRQVYNRRQVTLGVVYVSIGATLVGSIILETVRGEEFGYFF
GSTLVLLFELVRVGFG
>497559121_Chlamydia_trachomatis-phosphatidylserine_decarboxylase_proenzyme_
VNSFSRGCVIFVIEESLYSFNDFFVRKLKARPIICVTPADGYLFMLFTIKNFSLESFLGSMAIARLAPDYHRFHFPIARRIGHLFSIHPLMLKRVFNKREITIGVAYVEVGALNVGSIQPSYVKGAEKGFFF
GSTVVLLFQLCRMGLG
>599568345_Chondromyces_apiculatus_DSM_436-Phosphatidylserine_decarboxylase_
---------MYTLDAAPYSFDAFFTRALRVRPPSFISPADGLHAVLL-VKGYLVSELVGQFAVIYLSPDYHRVHSPVIRSMGDLYPVNSIGERHLFNRRVAIVGVTVVMVGAMIVGRIVLARLRGDEIGRFL

GSTAVMLVELVLFGLH

>499265967_Clostridium_acetobutylicum-phosphatidylserine_decarboxylase_

LIKFSSGFIVFIILESPFSFNDFFTRKLTARPFILISPGDGLLYILVEIKGYSLKELIKICMILRLCPDYHRFHFVDCSKIGSYYSVNPIALNKLFNKREWSIKILYIEVGATCVGSIQT--VKGDEKGYFF

GSTVVLFFEIVFMGIG

>169843118_Coprinopsis_cinerea_okayama7_130-phosphatidylserine_decarboxylase_1_

VLPMSRGVLYYALDEILYSLGAFFYRKLKARPVILVSPADGILFIVEQVKGYHLDALLGFFSVIYLAPDYHRFHSPTVRHFGDLFSVSPWMAKRLFNERVALLGFSMVPVGATNVGSIVLTNLYAEEMGGFL

GSTIVLVFEIVKVGLG

>169863595_Coprinopsis_cinerea_okayama7_130-phosphatidylserine_decarboxylase_proenzyme_2_

ALKLSIGKAIFIVDEVLFTFNEFFYRKLKARPVRLVSAADCFMFVKIWIKGFSVSKLLGALAIFRLAPDYHRFHSPVVTYIGEYYTVNPQAIRTVYNARKIVPGVMAVCVGAMMVGSITEQTVRGDEFGYFF

GSTIVILFELVRVGIG

>504209987_Corallococcus_coralloides-phosphatidylserine_decarboxylase_

LLPVSSGAAMFAMEEAFYTFAQFFTRGLKLRPVVVVSPVDGVSVSCLQAKGYTVDELLGAWTTVYLSPDYHRIHSPLIAYIGEFWPVNPASVKNLFNERLVTYGVAVVKVGATCVSRIAITHVKGGELGRFM

GSTVILCFEMVRMGIG

>799336207_Cryptococcus_neoformans_var__grubii_H99-phosphatidylserine_decarboxylase_

VLPLSQGLLFYALDEVLYSLGDFFYRELKARPIPMVSPADGVLFAVEQVKGYSLEALLGFFLVIYLAPDYHRFHSPTVRHFGDLFSVSPYIANRLFNERVALLGYSMIPVGATNVGSIVLTNLAGDEMGGFL

GSTIVLVFELVKVGLG

>799326038_Cryptococcus_neoformans_var__grubii_H99-phosphatidylserine_decarboxylase_

VLRKSIGTGIFILDELLYTFNSFFSRRLIARPIVVISAADCLTYVKFWIKGFTLPNLLGALSIHRLAPDYHRFHSPVIKDIGELYTVNPQAINEVFNKRSIMLGIAFVAIGAMLVGSIWPDKVKGEELGWFY

GSTTITVFSLVRVGIG

>799339861_Cryptococcus_neoformans_var__grubii_H99-phosphatidylserine_decarboxylase_

ALKLSIGKAIFIINEILFTFNEFFYRKLKARPIRLVSCADCLMFVQLWIKGFTIGRLLGALAIFRLAPDYHRFHSPVITMIGEYYTVNPQAIRSVYNVRKVVPGVMTVWVGAMMVGSITEQEVRGDELGYFF

GSTIVCIFELVRMGLG

>126644769_Cryptosporidium_parvum_Iowa_II-phosphatidylserine_decarboxylase__partial_

LLPRRRGLLFYSKFEELYSIGELFTRSIREIVFSISSPCEGTIFICIQVKSFKVSELLEYYMIIYLSPDYHRFHSPTIRHIGECFPVFKGIASKLFNERVVIKGMYIVAVAAHGVSDILLTNYKGDELGLFL

GSTIVLIFQVLKLGIG

>544212964_Cyanidioschyzon_merolae_strain_10D-phosphatidylserine_decarboxylase_

ALPLSRGVLLYCLDDILFTLAAFQQRQLKARPISLVAPCDGLLCELLQIKGLSMRALLSYYAVFHLPAGYHRFHSPAIRHVGQLLPGTPRILRRLFNERVCLVGFGMVAVAAFGNGDILLTNLPGDEMGGFQ
GSCIVLLFEVVRTGLF
>488696966_Cystobacter_fuscus-phosphatidylserine_decarboxylase_
LLPLSTGAAIFAVAEALYTFAEFFTRGLKARPIVVVSPVDGVSVSVLQAKGYTVGELLGAWTTIYLSPDYHRIHAPLVAYLGEFWPVNPASVKNLFNERLVTYGCAVVKVGATCVSRIAITHVKGGELGRFM
GSTVILLFELVRMGIG
>321466714_Daphnia_pulex-hypothetical_protein_DAPPUDRAFT_305406_
YIPISRGLLLYALEEALYCLAEFFRRKLKVRPISVVAPSDGVLFTMEQVKGYSLGEFLGFHCVIYLAPDYHCFHSPVVRHFGALLSVNPSIAKWLFNERAVYVGFSMTPVGATNVGSIVLTNLKGEIFGEFL
GSTIVLIFEIIRMGLH
>66801107_Dictyostelium_discoideum_AX4-hypothetical_protein_DDB_G0292748_
RLGLSYGILYWIQDEILYSLADFFSREIIARPIGTVSPVDGVLCVVEQVKGYSISHFLGFHCILYLSPDYHRIHSSEIHHFGTLFPVNKAFLKLLFNERIVLTGYSMTAVGAYNVGSILTTNQRGQEIGQFL
GSTVVLIFEVCKMGIG
>268638101_Dictyostelium_discoideum_AX4-phosphatidylserine_decarboxylase_
LIPTSNGLIYYAIDEAIYTMGDFFARRLKARPIDMVSPVDGVIHDLEQVKGYTLDQFLGYHIGLYLSPDYHGIHSPIIYHFGYLFPVAKVAVDNLFNERVVLTGYSLTPVGASNVGTIMLTNSKGSELAFFM
GSTVIMIFEIVKLGMG
>66812174_Dictyostelium_discoideum_AX4-hypothetical_protein_DDB_G0282337_
VMRLTNGKEIFIVDEILFNFNQFFYRKLKARPIIAVSPADCLNFIELWIKGFTLTTLIQSLVIARLAPDYHRFHVPVITPIGELYTVNPIAIREVYNKRIVTEGVLFISVGATLVGSILQQHVKGDEQGYFF
GSTILLLFELIKVNLG
>330791763_Dictyostelium_purpureum-hypothetical_protein_DICPUDRAFT_147678_
RLGLSYGILYWIQSEILYSLADFFSRPIIARPIGTVSPVDGVLCKVEQVKGYSISHFLGYHCILYLSPDYHRIHSSEIQHFGTLFPVNKPFLKLLFNERIVLTGYSLTAVGAYNVGSILTTNQRGQEIGQFL
GSTVVLIFEICKMGIG
>330801960_Dictyostelium_purpureum-hypothetical_protein_DICPUDRAFT_55778_
FIPTSNGMIFYSIDEAIYTMGDFFARHLKARPIDMVSPVDGVIHVVEQVKGYTLEQFLGYHIGLYLSPDYHGIHSPVIYHFGYLFPVAKVAVDNLFNERVVLTGYSLTPVGASNVGTILLSNSKGDEVAFFM
GSTVILIFEIVKFGMG
>330797644_Dictyostelium_purpureum-hypothetical_protein_DICPUDRAFT_77743_
VMRLTNGKEIFITDEIIFNFNQFFYRKLKARPIIAVCPADCLNFIELWIKGFSLQSLVQSLVIARLAPDYHRFHVPVIIPIGELYTVNPIAIREVYNKRVVTEEVLFVSVGATLVGSILPQKVKGDELGYFF
GSTILLLFKLVKVNLG

>24649524_Drosophila_melanogaster-CG5991__isoform_A_
ILPISRGLLYYSLSEAYYSLAEFFTRPLKVRVIPLVSPADGVLFSIEQVKGYSIEDFLGYQCVIYLAPDYHRFHSPTPRHFGELLSVSPKVAGWLFNERVLYMGFSYTAVGATNVGSVILTNFKGDLVGQFM
GSTIVLLFEIIRVGLG
>255982648_Drosophila_melanogaster-RE68005p_
ILPISRGLLYYSLSEAYYSLAEFFTRPLKVRVIPLVSPADGVLFSIEQVKGYSIEDFLGYQCVIYLAPDYHRFHSPTPRHFGELLSVSPKVAGWLFNERVLYMGFSYTAVGATNVGSVILTNFKGDLVGQFM
GSTIVLLFEIIRVGLG
>1013930299_Drosophila_simulans-uncharacterized_protein_Dsimw501_GD21059__isoform_A_
ILPISRGLLYYSLSEAYYSLAEFFTRPLKVRVIPLVSPADGVLFSIEQVKGYSIEDFLGYQCVIYLAPDYHRFHSPTPRHFGELLSVSPKVAGWLFNERVLYMGFSYTAVGATNVGSVILTNFKGDLVGQFM
GSTIVLLFEIIRVGLG
>446857677_Escherichia_coli-phosphatidylserine_decarboxylase_proenzyme_
LLPLTRGGAIFVMKEATYTFNEFFVRPLRVRPIVLVMPADGISLIILQAKGYSLEALLGTFVTTYLSPDYHRVHMPCLIYVGDLFSVNHLTAQNLFNERVICLGMAQILVGATIVGSITIPPLKGQEMGRFL
GSTVINLFALTKIGLA
>971421155_Gallus_gallus-PREDICTED__phosphatidylserine_decarboxylase_proenzyme_isoform_X1_
LVPLSRGLLYYIMKEALYNLSEFFRRKLKARPVSVISPSDGILFKVEQVKGYSLESFLGYHCVIYLAPDYHCFHSPTVRHFGSLMSVNPGVARWLFNERVVLTGFSLTAVGATNVGSIILTNMKGEHLGEFL
GSTIVLIFELIRFGLG
>159115374_Giardia_lamblia_ATCC_50803-Phosphatidylserine_decarboxylase_proenzyme_
ASPISTGLIYWCTSEILYSLDAFFTRKVNTKNFVLFSPADSLTYFTLTCKGYSASDLLPHWAVFYLSPDYHRFHAPCVKHAGDLLPVNSLFLPRLLNERVILSLFAYVAVGALNVGSII---YAGEEVGQFF
GSTIVLVYEAIRVGFV
>491962598_Haemophilus_influenzae-phosphatidylserine_decarboxylase_proenzyme_
FMPLTQGFWIFAMSIAFYSFNEFFIRPLKARPIALCLPADGISCDLLQAKGFSLEDLLEEFVTTYLSPDYHRVHMPCLIYVGDLFSVNPFLAQHLFNERVICVGMVQILVGATITASITIPPLKGQEMGWFL
GSTVINLFQLVRMGLA
>13489112_Homo_sapiens-phosphatidylserine_decarboxylase_proenzyme__mitochondrial_isoform_d_
LVPLSRGLLYYIMKEALYNLSEFFRRKLKARPVSVISPSDGILFKVEQVKGYSLESFLGYHCVIYLAPDYHCFHSPTVRHFGSLMSVNPGMARWLFNERVVLTGFSLTAVGATNVGSIILTNMKGEHLGEFL
GSTIVLIFELIRFGLG
>170099073_Laccaria_bicolor_S238N_H82-predicted_protein__partial_
VLPMSRGVLLYALDEILYSLGAFFYRKLKVRPVALVSPADGMLFVVEQVKGYSLDALLGFFAVIYLAPDYHRFHSPTVRHFGELFSVSPFMAKRLFNERVALLGFGMVPVGATNVGSIVLTNLPAEEMGGFL

GSTIVLVFEVVKVGLG

>170087950_Laccaria_bicolor_S238N_H82-predicted_protein_

VLRQTEGTKIFVTDELLYNFNDFFARKLLARPVRICSAADSLTYVQFWIKGFNIPNLLVSLAIFRLAPDYHRFHSPIVDHVGQFYTVNPQAVNEVFNSRSVLYGVAFVAIGALLVGSIWKSTVRGEELGYFY

GSTVVTVYPLVKAGLG

>389595267_Leishmania_major_strain_Friedlin-putative_phosphatidylserine_decarboxylase_

FMPFSNGLSAVVMSESKFTFQQFYVRDWTARPVSVVAPCDGVLVVLVQVKGYGMRSLLQVAVVLHMRNDFHHVIAPLCIYVGSLLPTTSAGYHWVLNERLILQAVYMALVGSTLTGRILVTNVRGERLATFW

GSSVVLVMDKVKAGLF

>501357930_Leptospira_biflexa-phosphatidylserine_decarboxylase_

FLPISKGFLLFALSEAIYSLNQFFTRALRARIIAVVSPTDSITFIIIQAKGYSVKELLGKYITFYLSPDYHRIHSPFIYYEGKLFPVNDLAVLNLFNERLITFGIAVIKVGASNVGKIVITNIKGSEMGRFM

GSTVILVFEIIQYGVG

>499873006_Myxococcus_xanthus-phosphatidylserine_decarboxylase_proenzyme_

LLPLSTGAVMFAMEEAFYTFAQFFTRGLKLRPVVVVSPVDGVSVSCLQAKGYTVDELLGAWTTIYLSPDYHRIHAPLIAYIGEFWPVNPASVKNLFNERLVTYGCAVVKVGATCVSRIATHTVKGGELGRFM

GSTVILLFELVRLGIG

>290988313_Naegleria_gruberi_strain_NEG_M-phosphatidylserine_decarboxylase_

LIPISRGILYYGMEEILYSLQDFFSRKLKTRPSLMVSPVDGVAFDLPQVKGYSMQQLLKHYCVLYLAPDYHRFHSPCVKHIGKLYPVMPLYLNKLYNERVVLNGMHYVIVGALNVGSCVLTNIKGEELGYFL

GSTIILIFELVRLGIL

>85103222_Neurospora_crassa_OR74A-phosphatidylserine_decarboxylase_proenzyme_1_

VLPMSRGFIFYALDEVLFNLAAFFYRTLKVRPVALVSPSDGILFEIEQVKGYTIDALLGYYAVIYLAPDYHRFHSPTVRHFGELFSVSPYLQRTLFNERVVLLGFGYVPVGATNVGSIILTNLRGEEMGGFL

GSTIVLVFEVIKMGLG

>85103909_Neurospora_crassa_OR74A-phosphatidylserine_decarboxylase_proenzyme_

ILKLSVGKNIFILSEVLFNFNEFFYRALKARPCIVVSPADCCVFINVWIKGFTVKRLLGALGIFRLAPDYHRFHIPVMKTIGEYYTVNPMAIRSVYNVRVLVPGVMVVCVGAMMVGSTIEDEVRGEELGYFF

GSTIVVLFELIRVGVG

>336478784_Parachlamydia_acanthamoebae_UV_7-phosphatidylserine_decarboxylase_proenzyme_

FFPFSSGWWIFIPSEFVFSFNDFFIRKLKARPLVAIMPADGYLYIGFVVKGFSLDELLKTMVMARLCPDYHRYHFPCPNMIGDLYSVNPIAIRQIFNKRTLCEGVLFIEIGATFVGSIQPSPIKGDEKGYFF

GSSLILLFELCLMGLG

>145536201_Paramecium_tetraurelia_strain_d4_2-hypothetical_protein__macronuclear__

ASVVSYGMIFFGYDDMLYNFQQFFTRKIK-REFKLIVPADSVLFVPILVKNYKLGYFLGYSVIFYLAPDYHRYHLPSLSHIGHLAPVKISYISSVYNERVALFGMSIVLVGATNVGSMLFTNLKGDELGMFL
GSTVVMMFEICKWGFA
>145541768_Paramecium_tetraurelia_strain_d4_2-hypothetical_protein__macronuclear__
ASVVSYGMIFFGYEDMLYNFQQFFTRKIK-REFKLIVPADSVLFVPILVKNYKLGYFLGYSVIFYLAPDYHRYHLPSLSHIGHLAPVKISYISSVYNERVALFGMSIVLVGATNVGSMLFTNLKGDELGMFL
GSTVVMMFEVCKWGFA
>145548758_Paramecium_tetraurelia_strain_d4_2-hypothetical_protein__macronuclear__
KLPVSQGVIYFSRQDMLYTFNKFFTRQIK-RKIGMVSPADSILITCLLVKRYQIGQFLGWSCIFYLAPDYHRYHCPVALHIGKLAPVKESSLRQLYNERVVLEGMYIIFIGATNVGSMVLTNIKGQEIGRFM
GSTVVIIFEAVYFGVA
>145485470_Paramecium_tetraurelia_strain_d4_2-hypothetical_protein__macronuclear__
--------IYFSRQDMLYTFNKFFTRQIK-RKIGMISPADSILIVCLLVKRYQIGQFLGWSCIFYLAPDYHRYHCPVALHIGKLAPVKESSLRQLYNERVVLEGMYIIFIGATNVGSMVLTNIKGQEIGRFM
GSTVVVIFEAVYFGVA
>294953035_Perkinsus_marinus_ATCC_50983-phosphatidylserine_decarboxylase__putative_
YLCRSRGMVFYALTEILYSFQEFFTRALKARPITMVCPCDGVVLIVSQVKGYMLSGFLGMYAVIYLSPDYHRFHSPTLRHFGDVLPVMKPFASALFNERVVLSGCHYVPVAAYNVGGILLTNFHGDPIGTFL
GSTIVMMFEVVKMGLT
>168030155_Physcomitrella_patens-predicted_protein_
FLPISRGFLYWALEEAVYSLRAFFIRKLKARPLNLISPVDGVVYKIEQVKGYSLPALLAFYCVLYLGPDYHRIHSPSIRHFGKLFPVNDRAVRTLYNERVVLEGMAMAAVGATNVGSIILTNVAGDEVAVFL
GSTVVLVFELVKMGIG
>168045623_Physcomitrella_patens-predicted_protein_
TLQISEGREIFIVDEIMFTFNQFFIRELKVRSIVAVSAADSLMFPRFWIKGFSVKGLLEPMVIFRLAPDYHRFHSPVIDDVGMLYTVNPIAVTSVFNKRAICLGVAFVAIGATMVGTIWEDHVKGEEMGYFF
GSTVICVFQLVFMGIG
>568053331_Phytophthora_parasitica-phosphatidylserine_decarboxylase_
SFPASRGVLYWTLDEMLYNLGEFFSRPLKVRPFHLASPVDGVAIVLEQIKGYRLDEFLGFHCVLYLAPDYHRIHAPVVRHFGNLFPVNQTAARLLFNERVALLGFSLTAVGATNVGSILFSNTRGEEMAQFL
GSTVVLVFEIVSYGVG
>906524155_Plasmodium_falciparum_RAJ116-phosphatidylserine_decarboxylase_
WLFRSRGIIYFIKEEIIYSLGDFFSRYIRTRPISIVSPCDSIVFLLDNVKGFNIKTFLGYYAIFYLSPKYHHFHAPFYRHIGEVFPVFQGMFKILFNERVILSGVYYAAISAYNVGNIILTNLIGDEVGEFV
GSSIIVIFEVISVGIG

>1160569205_Polysphondylium_pallidum_PN500-phosphatidylserine_decarboxylase_
-------MVYYAVDEALYSLAAFFCRRLKARKIPLVSPVDGVIYTLEQVKGYSLTHFLGYHVGIYLSPDYHGIHSPCINHFGYLFPVAKVAVDNLFNERVVLAGFSLTPVGASNVGTIMLTNQKGEELAFFM
GSTVILIFELVILGIG
>1160567525_Polysphondylium_pallidum_PN500-hypothetical_protein_PPL_11383_
RLGMSYGILYWAMDEILYSLADFFSRPIKARPIGMVSPVDGVLCVVEQVKGYSINQFLGYQCILYLSPDYHRIHSSEISHFGTLFPVNKPFLKLLFNERIVLMGYSMTAVGAYNVGSILVSNQKGQEVGQFL
GSTVVLIFEVCKMGIG
>1160552353_Polysphondylium_pallidum_PN500-Phosphatidylserine_decarboxylase_proenzyme_2_precursor_
VLKLTTGKEIFITDEILFNFNEFFYRKLKARPISAVSPADCLHFIELWIKGFNLSSLLDSLVIARLAPDYHRFHIPVIKPIGDYYTVNPIAIKEVYNKRAVTIDVLFVSVGATMVGSILEQTVKGDEQGYFF
GSTILLLFKIVKVGIG
>242211984_Postia_placenta_Mad_698_R-predicted_protein_
ADPAQTNVTHMHLVHKILHVGNFVIDRTN-RFFPIYTRLGMLLY-KVLSKSAVLRDLSVAHDSVKLAPDYHRFHSPIVRDFGEYYTVNPQAVNEVFNKRAVLSQVAFVAIGAMLVGSIWPAQVRGDELGYFY
GSTVVVLFPLVKVGIG
>489534254_Pseudomonas-MULTISPECIES__phosphatidylserine_decarboxylase_
SLPLSRGIVTFAMSQAVYHFNAFFTRALKARPLAVLSPADGVSLIVFQAKGYSVLELVGEFATIYLSPDYHRVHMPLLIYVGRLFSVNQTTAENLFNERAVCIGMAVVLVGAMIVASITVPPLKGAELGRFL
GSTAIVLFGLVMMGIG
>6324160_Saccharomyces_cerevisiae_S288C-phosphatidylserine_decarboxylase_1_
CLPMSRGVLYYSLDEMLYNLSEFFYRNIKTRPVVIASPSDGILVNIEQVKGYSIKEFLGFFAVIYLAPDYHHFHSPVCRHFGDLFSVAPYFQRNLFNERVALLGFSMTPVGATNVGSILFTNLKGEEMGGFL
GSTVVLCFEVVKMGLG
>6321609_Saccharomyces_cerevisiae_S288C-phosphatidylserine_decarboxylase_2_
FLKLSIGKAIFILSQC-FTFNEFFYRKLKSLPEILFSPADSCTFIEIWVKGFSIKKLANSIGIFRLAPDYHRFHSPCIVYVGEYYTVNPMAVRSVFNIRVIIPGLLYIPIGAMMVGSILEDVVSGQELGYFF
GSTIIIIIPLVKVGIG
>813126063_Saprolegnia_parasitica_CBS_223_65-hypothetical_protein_SPRG_02934_
GIPISRGVLYWTLHEMLYNLGEFFSRPLKARVIVMSSPVDGMAIVLEQIKGYRMDEFLGYHCVIYLAPDYHRIHSPIVRHFGNLFPVNKFAVNLLFNERVALLGYSLTAVGATNVGSIILTNVRGDQVAQFL
GSTVVLVFEVIRFGMG
>558600419_Spironucleus_salmonicida-Phosphatidylserine_decarboxylase_proenzyme_
-MV------IWITNDILFSLSGFFNRRINNRYIVIYSPSDSLTVFKAKIKGFTLKQILKAYCVFYLSPDFHDFVSFNSTHIGDLMPVNNIFTHKLLQERVVVEGAFYVPVGALNVGNIMITNYTGETIGQFL

GSTVVIIFEIVMIGCI
>503141464_Stigmatella_aurantiaca-phosphatidylserine_decarboxylase_
LLPLSSGAAMFAMAEAFYTFAEFFTRGLKLRPVVVVSPVDGVSVSCLQAKGYTVDELLGAWTTLYLSPDYHRIHAPLIAYIGEFWPVNPASVKNLFNERLVTYGCAVVKVGATCVSRIALHTVKAGELGRFM
GSTVILLFELVRLGIG
>146181023_Tetrahymena_thermophila_SB210-phosphatidylserine_decarboxylase_
SSVVSNGVIYFSEEDILFTFNEFFTRQIKQRNIIIVSPADSCLIQLL-VKGYKLGEFLGYQAIFYLNPDYHRYHSCAFNHIGYLAPVKVSYISKVYNERVALFGFSMVFVGATNVGSMVVTNVKGEEIGQFM
GSTVVIFFEIVRMGIV
>829185877_Tetrahymena_thermophila_SB210-phosphatidylserine_decarboxylase_
KARVSRGVIFFAENDMFYTFNQFFTRRVK-RQIVILSPADSVLIVNILVKGYKMGEFLGYQAIFYLNPDYHRYHSPTANHIGYLAPVKESYISKVYNERVALFGFIQVYVGATNVGSMLLTNIKGAEIGRFL
GSTVVVFFEVVRYGVC
>499689602_Thiomicrospira_crunogena-phosphatidylserine_decarboxylase_
PIPLSKHFQILTIQEAIYHFNAFFTRALKARPIAWCSPADGISSILIQAKCYSLDALLGDSAVIYLSPDYHRIHMPVLTYVGDLFAVNPTTVRNLFNERLIIRGFCLIMVGAIFVGSMTIPDYKGDEIGRFM
GSTVVLLSPIIQMGLA
>1085103788_Toxoplasma_gondii_ME49-phosphatidylserine_decarboxylase_
YLFRSRGVIYLATEEILYCIGHLFARTLKEREISLASPADGVTLVVEQVKGYSLRAFLGKFVVLHLKPNYHHFHAPAVRHMGETLPVFSSFLKRIFNERVVMSGMHMVAVAAYNVGNIILTNYVGQHVGEFL
GSTIVLIFEMVRVGLG
>1085045473_Toxoplasma_gondii_ME49-phosphatidylserine_decarboxylase_
LFPNTRGMAILILEEAAFSVQEFFTRPINVRDMSIMAPADSIQIIIPQVKSFNLREFLGFVSILYLAPDYHRVHSPAVTYIGCTPSVSRRNLEALLYERTALIEFSVTMVAAMFVGGLLPGALASQEIGAFF
GSTVVMIFEVVAAGAG
>71745350_Trypanosoma_brucei_brucei_TREU927-phosphatidylserine_decarboxylase_
MFPISHGICIIIAPRG-YTLQEFFVRRWEERRVPVVMPSDGVLVVLLQVKGYSVRRLFSIAVALHLRTDYHHVVTPCCVYIGALLPHTPAGYHWVLNERVVLLGMGLALVGGTLTGRILITNSKGDLLSTFW
GSSVVLVLDVVKAGLV
>758977635_Ustilago_maydis_521-hypothetical_protein_UMAG_11468_
VLPISRGALFYALDEMLYSLGEFFYRELKARPVILVSPADGVLFAVEQVKGYSLDALLGFFCVIYLAPDYHRFHSPTARHFGELYSVSPYMARRLFNERVALLGFGMVPVGATNVGSIILTNLAGDEMGGFL
GSTIVLVFELVKVGLG
>758978194_Ustilago_maydis_521-hypothetical_protein_UMAG_02799_

VLKMSIGKAIFITDEILFTFNEFFYRKLKARPNRLVSGADCMMFIRIWIKGFSVSRLLGALAIFRLAPDYHRFHCPAVTWIGQYYTVNPMAIRSVYNIRVVVPGFYAVCIGAMMVGSTLEQHVRGDEFGYFF
GSTIVLVFELVRVGIG
>758977525_Ustilago_maydis_521-hypothetical_protein_UMAG_10130_
VLKVSVGVSIFVLDELPYSFNSFFFRKLKARPIIVSSCADCLTFVRYWIKGFTLNRLIGSIAIFRLAPDYHRFHYPVCRHIGEYFTVNPQAVNAVFNRREVLVGVAFVAIGAMLVGSIWQSSVRGDECGYYY
GSTNIVIFPLVRVGIG
>446280066_Vibrio_cholerae-phosphatidylserine_decarboxylase_proenzyme_
LIPLTRGLAIFIMDEAPFTFNEFFVRELKVRPIVITHPADAVSFILIQAKGYSAQELLGDFATLYLSPDYHRVHMPCLIYVGDLFSVNPLTAENLFNERVVCIGMAQVLVGATIVGSILVPPLKGEEMGRFL
GSTVINLFAMTRMGYA
>297744390_Vitis_vinifera-unnamed_protein_product__partial_
FLPISRGLIYWALEEALYTLRDFFVRSLKSRPICLVSPVDGILFLIEQVKGYSVSSLLGFYCVIYLKPDYHRIHSPIVRHFGRLFPVNERATRTLYNERVVLEGMXIAAIGATNICTLLLTNLKGDEMAAFM
GSTVVLVFQTIRVGLG
>225447822_Vitis_vinifera-PREDICTED__phosphatidylserine_decarboxylase_proenzyme_2_
ALQISEGQEIFLLDEVLFTFNEFFIRELKARPIVAVCAADSLTFVRFWIKGFSIQGLLGSLVIFRLAPDYHRFHFPVIVDIGCLYTVNPIAVNSVFNKRVVSVGVAFVAIGATMVGSIFKDYVKGEEFGYFF
GSTVICVFELVAVGLG
>667640782_Vittaforma_corneae_ATCC_50505-phosphatidylserine_decarboxylase_
AMRLTVGRTIFIINEC-FSFNDFFSRKLKARFVKVSSPADCLLN-LLGIKGFKVEELLRNICICRLAPDYHRFHAPLVYHIGSYHSVNP--INKVLNARSVIVGIYYVAVGATLVGSILADHLQMDEIGYFF
GSCVVLITDIVRVGLE
>1078657311_Yarrowia_lipolytica-hypothetical_protein_YALI1_D27274g_
VLPLSRGFLYYSLDEVLYNLGEFFYRELKARPIDIVCPADGVLLNVEQVKGYSLEALLGFFAVIYLAPDYHRFHSPVARHFGELYSVAPYFQKKLFNERVALLGFSMTPVGATNVGSIILTNLKGDQMGGFL
GSTVVLVFEIVRVGIG
>50549787_Yarrowia_lipolytica_CLIB122-YALI0D03480p_
VLRLTNGKLIFILSDVLFTFNQFFYRKLKARPLAVCCAADSATYVQIWIKGFTIKRLFGSIAIFRLAPDYHRFHSPVVKTIGEYYTVNPMAIRSVFNVRVLTPGVMFIAVGAMMVGSTMEEHVRGQELGYFF
GSTCLVLFQLVRVGLG
>488137933_Yersinia-MULTISPECIES__phosphatidylserine_decarboxylase_proenzyme_
LLPLTQGGGIFAMKEAFYTFNEFFVRPLRVRPVLLAQPADGISLRILQAKGYSLEALLGQFVTTYLAPDYHRVHMPCLIYVGDLFSVNPLTAANLFNERVICIGMAQILVGATIVGSITIPPLKGQEMGRFL
GSTVINLFALTRMGLA

Supplementary Data S5. The edited multiple sequence alignment for the phylogenetic tree reconstruction of LPCATs

>76154337_flatworms_Schistosoma_japonicum__SJCHGC09609_protein__partial_
----------SSFLDVGKTESAES-FVGIVIFPEGTCTNRSCIATFKSGA
FSAGVPVQPVVVRWPNKVDCVTLKLLWLAMTQLYNKLENEDEQLDAELYA
NNIRRLMAEHLNIPLCDLSYDD
>147897915_other_sequences_synthetic_construct__Lysophosphatidylcholine_acyltransferase_1__partial_
EAAILTLAPHSSYFDVMKAESRDIPIWGIMIFPEGTCTNRTCLITFKPGA
FIPGAPVQPVVLRYPNKLDTITLEILWLTLCQFHNQVESEEEKRNPALYA
SNVRRVMAEALGVSVTDYTFED
>156555662_wasps_c._Nasonia_vitripennis__PREDICTED__1_acylglycerophosphocholine_O_acyltransferase_1_isoform_X1_
EAPILALAPHSSFFDVAKAETGRIPFFGVMIFPEGTCTNRSCLITFKSGA
FYPGVPVQPVCIRYPNKLDTVTLKLLWLTLTQLNSSCESEAEKLDPKLYA
NNVRRLMAEALQIPVSDYTYDD
>156717626_frogs__toads_Xenopus_(Silurana)__lysophosphatidylcholine_acyltransferase_2_
DAPILLVAPHSSFFDVSRAENISVPIFGVLIFPEGTCTNRSCLISFKPGA
FHPGVPVQPILLRYPNIQDTVTEKLLLLTLCQICTNVTSEEEKKDPFLYA
HNVRHTMAQALGLPVTDHTYED
>159108651_diplomonads_Giardia_lamblia__Hypothetical_protein_GL50803_2692_
--CHAIVANHTDFMDVSKEAATRFWAIRLLIFPEGTTTTGKSLLRFHTSI
FRLGVSVQPISIKY-YSWIH--FRVLIRKLFNPFCVTV-NGE-LSPRTLA
DVAGKRIADSLHIPYLPYTSED
>159108775_diplomonads_Giardia_lamblia__Hypothetical_protein_GL50803_7126_
---IICVSNHVGLLDVSKIGVSSIYLLRIHIFPEGTTTTERGLLRFRTSV
FRLDTDIQPICLIY-RSYNN--LYTLYSYLTNPFCFVKPTGERKDPRAFA
DEVGLAMARYMGSEYLPYTNED
>159114202_diplomonads_Giardia_lamblia__Hypothetical_protein_GL50803_12109_

KDAPVVVANHSSIMDLNNWAIRQIFLAAIVLFPEGTVTPASCFTRFKTGA
FRLNVPVQPVTVRYRSILSTC-LFNLYKILANPVTLVEASEET--PRAFA
DRVGKYMADALGAVYTNYTNDD
>167999847_mosses_Physcomitrella_patens__predicted_protein__partial_
-VAPIIVSNHIGFVDVSAKENVEMPIIGVMLFPEGTTTNGKALISFKTGA
FSPGLPVQPMVIKYPHKYVNPCLVILFQLMTQFVNYMENVHEIKNPHEFA
NRVRTEMAKALGVVCTEHNFLD
>182888253_other_sequences_synthetic_construct__Lysophosphatidylcholine_acyltransferase_2_
EAPIFVVAPHSTFFDVSRNENAQTPLVGILVFPEGTCTNRSCLITFKPGA
FIPGVPVQPVLLRYPNKLDTVTLQLCVLTFCQLFTKVESEEEKNDPVLFA
SRIRNLMAEALEIPVTDHTYED
>190689341_other_sequences_synthetic_construct__lysophosphatidylcholine_acyltransferase_2_protein__partial_
EAPVFVAAPHSTFFDVSRNENAQVPLIGILVFPEGTCTNRSCLITFKPGA
FIPGVPVQPVLLRYPNKLDTVTIQLCMLTFCQLFTKVENDEEKNDPVLFA
NKVRNLMAEALGIPVTDHTYED
>215254108_even-toed_ungulates_Sus_scrofa__1_acylglycerol_3_phosphate_O_acyltransferase_9_protein__partial_
EATILTLAPHSSYFDVMKAESRDIPIWGIMIFPEGTCTNRTCLITFKPGA
FIPGVPVQPVVLRYPNSLDTITLKILWLTLCQFHNQGESEEEKKDPALYA
SNVRRVMAQALGISVTDYTFED
>224994338_even-toed_ungulates_Sus_scrofa__lysophospholipid_acyltransferase_LPCAT4_
QAPVLVAAPHSTFFDVSRAENLSVPVIGVLFFPEGTCSNKKALLKFKPGA
FIAGVPVQPVLIRYPNSLDTTSLKVLWLTASQPCSIVDSPEESRDPTLYA
NNVQRVMAQALGIPATECEF--
>242012711_lice_Pediculus_humanus__conserved_hypothetical_protein_
EAPVLAVAPHSSYFDVAKGETGILPLFGVLIFPEGTCTNRSCLITFKSGA
FYPGVPVQPVCIRYPNKLDTVTLKLLWLTLTQIHSCCENKEEKNDPRLFA

NNVRKVMARALGIPVSNYTYDD
>291390137_rabbits__hares_Oryctolagus_cuniculus__PREDICTED__lysophosphatidylcholine_acyltransferase_2_
EAPIFVVAPHSTFFDVSRNENAQTPLVGILVFPEGTCTNRSCLITFKPGA
FIPGVPVQPVLLRYPNKLDTVTLQLCVLTFCQPFTKVESDEEKSDPVLFA
SRVRNLMAEALGIPVTDHTYED
>301607133_frogs__toads_Xenopus_(Silurana)__PREDICTED__lysophosphatidylcholine_acyltransferase_1_isoform_X1_
EAPILTLAPHSSYFDVMKAESKDIPVWGVMIFPEGTCTNRSCLITFKPGA
FIPGVPVQPVVLRYPNKADTITMKILWLSLCQFHNYVESEEEKKNPALYA
NNVRRVMAKALGVSVTDYTFED
>302772579_vascular_plants_Selaginella_moellendorffii__hypothetical_protein_SELMODRAFT_3969__partial_
-DAPVLVCNHVTFVDVTAEENLNYPFMGLMIFPEGTTTNGKAMVSFKSGA
FSSSSPVQPMVVRYPHVHLDPSYALLFRLMTQFHNYMESKQE--NPRSFA
ERVRAEMARALNVVVTEHTFDD
>302774320_vascular_plants_Selaginella_moellendorffii__hypothetical_protein_SELMODRAFT_93203_
-VAPIVVSNHVSFLDLSAKENAKLPIVGVLIFPEGTTTNGKALISFKTGA
FAQGLPIQPMCIRYPHKCISPAYV-MFRLMTQLVNFMEGLRDLKDPRHFT
ETVRHMMAASLGVPCTEHTFLD
>302799108_vascular_plants_Selaginella_moellendorffii__hypothetical_protein_SELMODRAFT_420881_
-DAPVLVCNHVTFVDVTAEENLNYPFMGLMIFPEGTTTNGKAMVSFKSGA
FSSSSPVQPMVVRYPHVHLDPSYALLFRLMTQFHNYMESKQE--NPRSFA
ERVRAEMARALNVVVTEHTFDD
>311257245_even-toed_ungulates_Sus_scrofa__PREDICTED__lysophosphatidylcholine_acyltransferase_2_isoform_X1_
EAPIFVVAPHSTFFDVSRNENVQVPLIGILVFPEGTCTNRSCLITFKPGA
FIPGVPVQPVLLRYPNKLDTVTIQLCVLTWCQPFTKVENDEERSDPILFA
NRVRNLMAEALGIPVTDHTYED
>311274157_even-toed_ungulates_Sus_scrofa__PREDICTED__lysophosphatidylcholine_acyltransferase_1_isoform_X1_

EATILTLAPHSSYFDVMKAESRDIPIWGIMIFPEGTCTNRTCLITFKPGA
FIPGVPVQPVVLRYPNSLDTITLKILWLTLCQFHNQVESEEEKKDPALYA
SNVRRVMAQALGISVTDYTFED
>322799785_ants_Solenopsis_invicta__hypothetical_protein_SINV_04909__partial_
EAPILVLAPHSTFMDIVRRESGLNPFIGVMIFPEGTCTNRSCLITFKSGA
FYPGVPVQPVCIRYPNKLDTVTLKLLWLTLTQLNSSCESEAEKLDPKLYA
NNVRRLMAEALKIPVSDYTYDD
>327270203_lizards_Anolis_carolinensis__PREDICTED__lysophosphatidylcholine_acyltransferase_1_
EAAILTLAPHSSYFDVMKAESKDIPVWGIMIFPEGTCTNRSCLITFKPGA
FIPGVPVQPVILRYPNKLDTITLKILWLTLCQFHNFVESEEERKNPSLYA
NNVRRIMAKALGVSVTDYTFED
>327276427_lizards_Anolis_carolinensis__PREDICTED__lysophosphatidylcholine_acyltransferase_2_
EAPIFAVAPHSSFFDVSRQENLMAPFVGVLVFPEGTCTNRSCLITFKQGA
FIPGVPVQPVLIRYPNKLDTVTNEALVLTLCQLFTKMETDEEKHDPALFA
NRVRSKMSSALGVPVTDHTYED
>327278304_lizards_Anolis_carolinensis__PREDICTED__lysophospholipid_acyltransferase_LPCAT4_
EAPILVAAPHSTFFDVSRTENLHVPVIGVLFFPEGTCSNKKALLKFKPGA
FISGVPIQPILIRYPNSLDSTTLKVIWLTASQPCTTVESAEEAVNPTLYA
NNVQKVMAKALGIPATECEF--
>347964127_mosquitos_Anopheles_gambiae__AGAP000596_PB_
EAPVLVVSPHSSFLDLVRNAD-RN--LGILIFPEGTCTNRTSLIKFKPGA
FYPGVPIQPVLMRYPNKVDTVTIQLLWRTLTQFHTFCESEAEKANPKLYA
DNVRMLMAKALDIPISDYTFDD
>357611371_butterflies_Danaus_plexippus__hypothetical_protein_KGM_16166_
EAPILVVAPHSSFLDIVRKES-MDNYVGVLIFPEGTCTNRSCLITFKPGG
FYPGVPVQPVTIRYPNAKDTVTLKLLWLTLTQVHSSCESEEEKKDPKLYA

RNVRDVMAKALGVPVLDYTYDD
>390338389_sea_urchins_Strongylocentrotus_purpuratus__PREDICTED__lysophosphatidylcholine_acyltransferase_2_like_
EASILALAPHVSFFDVSRQENVEPPILGIVIFPEGTTTNGQCFITFKGGA
FFPGVPVQPVLLRYNNALNTFPFSVFWLTLCNLSNNLESEEEKHDPKLYA
RNVRAVMAKAYKVPVTDHTYED
>391339752_mites__ticks_Metaseiulus_occidentalis__PREDICTED__lysophosphatidylcholine_acyltransferase_1_like_
QAPLLVIGPHSSFMDCTKNDSKHIPLLGIVIFPEGTCSNGSVLIKFKQGA
FSAGVPIQPVLLRYPNRLNTLTLKTMWLTTCQWTTKMVSEAERQNPTLYA
ENVRQLMAAALGIGTTEFSYDD
>395503331_marsupials_Sarcophilus_harrisii__PREDICTED__lysophospholipid_acyltransferase_LPCAT4_
EAPVLVAAPHSTFFDVSRAENLSVPVIGVLFFPEGTCSNKKALLKFKPGA
FIAGVPVQPVLIRYPNSLDTTSLKVLWLTASQPCSLVDSPEESNNPTLYA
NNVQRVMAQALGIPATECEF--
>395505964_marsupials_Sarcophilus_harrisii__PREDICTED__lysophosphatidylcholine_acyltransferase_2_
EAPIFIVAPHSTFFDVSRTENIYVPLIGLLVFPEGTCTNRTCLITFKPGA
FIPGVPVQPILLRYPNKLDTVTIRLCMLTFSQLFTKVENDEEKSDPVLFA
NHIRNIMANALGVPVTDHTYED
>395510777_marsupials_Sarcophilus_harrisii__PREDICTED__lysophosphatidylcholine_acyltransferase_1_
EAAILTLAPHSSYFDVMKAESRDIPIWGIMIFPEGTCTNRTCLITFKPGA
FIPGVPVQPVVLRYPNKLDTITLKILWLTLCQFHNFVESEEEKKNPALYA
NNVRRIMAKALGVSVTDYTFED
>395530387_marsupials_Sarcophilus_harrisii__PREDICTED__lysophosphatidylcholine_acyltransferase_2_like_
EAPLLVAAPHSSFFDVSREENASYPFVGILIFPEATCTNRTCLITFKPGA
FVPGVPVQPLLLRYPNKLDTVTFQLLLLTLTQPYTNMEDEEERSDPALYA
RRVRNVMANALQLPVTDHTYED
>395837609_primates_Otolemur_garnettii__PREDICTED__lysophospholipid_acyltransferase_LPCAT4_

QAPVLVAAPHSTFFDVSRAENLSVPVIGVLFFPEGTCSNKKALLKFKPGA
FIAGVPVQPVLIRYPNSLDTTSLKVLWLTASQPCSVVDSTEESRDPTLYA
NNVQRVMAQALGIPATECEF--
>395839560_primates_Otolemur_garnettii__PREDICTED__lysophosphatidylcholine_acyltransferase_2_
EAPVFVVAPHSTFFDVSRSENAQAPLVGILVFPEGTCTNRSCLITFKPGA
FIPGVPVQPILLRYPNKLVSI-IQLCVLTFCQPFTKVESDEEKDDPVLFA
NRVRKLMAEALGIPVTDHTYED
>395859477_primates_Otolemur_garnettii__PREDICTED__lysophosphatidylcholine_acyltransferase_1_isoform_2_
EAAILTLAPHSSYFDVMKAESRDIPIWGIMIFPEGTCTNRTCLITFKPGA
FIPGVPVQPVVLRYPNKLDTITLKILWLTLCQFHNQVESEEEKRSPALYA
SNVRRVMAEALGISVTDYTFED
>405964295_bivalves_Crassostrea_gigas__Lysophosphatidylcholine_acyltransferase_2_
EAPIIALAPHSSFLDVAKQETSRAPVLGIIIFPEGTCTNRSCLINFKSGA
FYPGTPVQPVLIRYPNQLDTVTFALLWYTLCQFRTNVESEEEKKNAALFA
SNVRAKMAEALGVPVTDHSYDD
>410949809_carnivores_Felis_catus__PREDICTED__lysophosphatidylcholine_acyltransferase_1_
EAAILTLAPHSSYFDVMKAESRNIPIWGIMIFPEGTCTNRTCLITFKPGA
FIPGVPVQPVVLRYPNKLDTITLKILWLTLCQFHNRVESDEEKKDPALYA
SNVRRVMAEALGVSVTDYTYDD
>410983531_carnivores_Felis_catus__PREDICTED__lysophosphatidylcholine_acyltransferase_2_isoform_X1_
EAPIFVAAPHSTFFDVSRNENAQVPLIGILVFPEGTCTNRSCLITFKPGA
FIPGVPVQPILLRYPNKLDTVTIQLCMLTFCQLFTKVENDEEKSDPILFA
CRVRSLMAEALGIPVTDHTYED
>470304255_eukaryotes_Capsaspora_owczarzaki__lysophosphatidylcholine_acyltransferase_2_B_
EAPILCLASHSTFYDVTRKENVVAPVVGLFIFPEGTCTNRKALISFKSGA
FIPGVPVQPIALRYTNKHYDPCLRGLLFLLAQPVNYLETEEEQSSPALFA

NNVRNSLAAALNVGVTEHSYED
>488508270_placentals_Dasypus_novemcinctus__PREDICTED__lysophospholipid_acyltransferase_LPCAT4_
QAPVLVAAPHSTFFDVSRAENLSVPVIGVLFFPEGTCSNKKALLKFKPGA
FIAGVPVQPVLIRYPNSLDTTSLKVLWLTASQPCSIVDSPEESRDPTLYA
NNVQRVMAQALGIPATECEF--
>488526558_placentals_Dasypus_novemcinctus__PREDICTED__lysophosphatidylcholine_acyltransferase_2_
EAPIFVVAPHSTFFDVSRNENAQVPLIGILVFPEGTCTNRSCLITFKPGA
FIPGVPVQPILLRYPNKLDTVTIQLCVLTFCQPFTKVENDEEKNDPVLFA
SRVRSFMADALGIPVTDHTYED
>507621115_rodents_Octodon_degus__PREDICTED__lysophosphatidylcholine_acyltransferase_1_
EASILTLAPHSSYFDVMKAESRDIPIWGIMIFPEGTCTNRTCLITFKPGA
FIPGVPVQPVVLRYPNKLDTITLEILWLTLCQWHSQVESEEERRNPALYA
SNVRRVMAEALGVSVTDYTFED
>507662570_rodents_Octodon_degus__PREDICTED__LOW_QUALITY_PROTEIN__lysophosphatidylcholine_acyltransferase_2B_
EACIFVTAPHSTFFDVSASQNVQVPIGGILIFPEGVCTNRSCLITFKLGA
FSPGVPVQPVLLRYPNTLDTVTFQACMLTLSQTFTRVENDEEKKDPLLFA
NAVRINMANALGVPVTDHTYED
>507667803_rodents_Octodon_degus__PREDICTED__lysophosphatidylcholine_acyltransferase_2_
EAPIFVVAPHSTFFDLSRSENAQVPLIGILVFPEGTCTNRSCLITFKPGA
FIPGVPVQPILLRYPNELVSIPIQLCILTFCQPFTKVESDEEIKDPILFA
SRVRNLMAEALGIPVTDHTYED
>507695554_rodents_Octodon_degus__PREDICTED__lysophospholipid_acyltransferase_LPCAT4_
QAPVLVAAPHSTFFDVSRAENLSVPVIGVLFFPEGTCSNKKALLKFKPGA
FIAGVPVQPVLIRYPNSLDTTSLKVLWLTASQPCSIVDSPEESRDPTLYA
NNVQQVMAQALGIPATECEF--
>507962346_insectivores_Condylura_cristata__PREDICTED__lysophospholipid_acyltransferase_LPCAT4_

QAPVLVAAPHSTFFDVSRAENLSVPVIGVLFFPEGTCSNKKALLKFKPGA
FIAGVPVQPVLIRYPNSLDTTSLKVLWLTASQPCSIVDSPEESKDPTLYA
NNVQRVMAQALGIPATECEF--
>507968115_insectivores_Condylura_cristata__PREDICTED__lysophosphatidylcholine_acyltransferase_2B_like_
EAPIFVTAPHSTFFDVSASKNAKIPLVGLLIFPEGVCTNRSCLVTFKLGA
FTPGVPVQPVLLKYPNSMDTVTFQVLILTLSQPFTKIESEQEKRDPVLFA
NTVRIKMANALGLPVTDHTYED
>507979621_insectivores_Condylura_cristata__PREDICTED__lysophosphatidylcholine_acyltransferase_2_
EAPIFVVAPHSSFFDVSRNENVQVPLIGILVFPEGTCTNRSCLITFKPGA
FIPGVPVQPVLLRYPNKLDTVTIQLCMLTFCQPFTRVESDEEKSDPVLFA
SRVRNYMAEALGIPVTDHTYED
>512897441_moths_Bombyx_mori__PREDICTED__1_acylglycerophosphocholine_O_acyltransferase_1_like_
EAPVVVMAPHSSFFDVAKADTARIPFIGVLIFPEGTCTNRSCLITFKPGG
FYPGVPVQPVTIRYPNARDTVTLKLLWLTLTQVHSSCESEEEKRDPKLYA
RNVRDVMAKALGVPVLDYTYDD
>514485283_eukaryotes_Capsaspora_owczarzaki__lysophosphatidylcholine_acyltransferase_2_B_
EAPILCLASHSTFYDVTRKENVVAPVVGLFIFPEGTCTNRKALISFKSGA
FIPGVPVQPIALRYTNKHYDPCLRGLLFLLAQPVNYLETEEEQSSPALFA
NNVRNSLAAALNVGVTEHSYED
>524894594_gastropods_Aplysia_californica__PREDICTED__lysophosphatidylcholine_acyltransferase_2_like_isoform_X1_
EAPLLVVAHHTSFFDVSRLENAGVPLIGIIIFPEGTCTNRTCLISFKQGA
FYPGVPVQPVCVKYPNRLDTITFTQLWLTLCQFYTRIESEEEKADPKLFA
NNVRTLMAESLKCPVTDHTYDD
>524894596_gastropods_Aplysia_californica__PREDICTED__lysophosphatidylcholine_acyltransferase_2_like_isoform_X2_
EAPLLLVAPHSSFIDVSKASLQKVPIFGIIIFPEGTCTNRTCLISFKQGA
FYPGVPVQPVCVKYPNRLDTITFTQLWLTLCQFYTRIESEEEKADPKLFA

NNVRTLMAESLKCPVTDHTYDD
>527268415_birds_Melopsittacus_undulatus__PREDICTED__lysophosphatidylcholine_acyltransferase_2_
EAPIFVAAPHSSFFDVSRTENLSTPVFGVLIFPEGTCTNRSCLITFKQGA
FVPRVPVQPVLLRYPNRLDTVTKELCVMTLCQLFTKVETEEEKNDPIFYA
NRVRQIMATALNVPVTDHTFED
>527271138_birds_Melopsittacus_undulatus__PREDICTED__lysophosphatidylcholine_acyltransferase_1_
EAAILTVAPHSSYFDVMKAESKDIPVWGIMIFPEGTCTNRSCLITFKPGA
FIPGVPVQPVVLRYPNKLDTITLEILWLTLCQLHNSVESEEERKNPALYA
NNVRRVMAEALGVSVTDYTFED
>530592402_turtles_Chrysemys_picta__PREDICTED__lysophosphatidylcholine_acyltransferase_2_isoform_X1_
EAPILVVAPHSSFFDVSRLENLSAPVFGVLIFPEGTCTNRSCLITFKQGA
FIPGVPVQPVLLRYPNKLDTVTKELCVMTFCQPLTRVETEEEKKDPNLFA
NRVRNTMATALNVPVTDHTFED
>530593015_turtles_Chrysemys_picta__PREDICTED__lysophosphatidylcholine_acyltransferase_1_isoform_X1_
EAAILTLAPHSSYFDVMKAESKDIPVWGIMIFPEGTCTNRSCLITFKPGA
FIPGVPVQPVVLRYPNKLDTITLEILWLTLCQFHNFVESEEEKKNPSLYA
NNVRRVMAEALGVSVTDYTFED
>530593021_turtles_Chrysemys_picta__PREDICTED__lysophosphatidylcholine_acyltransferase_1_isoform_X5_
EAAILTLAPHSSYFDVMKAESKDIPVWGIMIFPEGTCTNRSCLITFKPGA
FIPGVPVQPVVLRYPNKLDTITLEILWLTLCQFHNFVESEEEKKNPSLYA
NNVRRVMAEALGVSVTDYTFED
>530593023_turtles_Chrysemys_picta__PREDICTED__lysophosphatidylcholine_acyltransferase_1_isoform_X6_
---------------------------QIMIFPEGTCTNRSCLITFKPGA
FIPGVPVQPVVLRYPNKLDTITLEILWLTLCQFHNFVESEEEKKNPSLYA
NNVRRVMAEALGVSVTDYTFED
>544217106_red_algae_Cyanidioschyzon_merolae__similar_to_1_acyl_sn_glycerol_3_phosphate_acyltransferase_

SSGTLIVSNHVSFFDVAKKEVLRLPFVGLVLFPEGTTSNGDALLRFHSGA
FLSGVPVRPLALRYGFWDFDPA-WRLACILAQPWMSLTSPTEMERPRLYA
ENIRALIARALQVECVDMGYRD
>557019001_coelacanths_Latimeria_chalumnae__PREDICTED__lysophospholipid_acyltransferase_LPCAT4__partial_
EAPILVGAPHSSFFDMSRAEDLKNPLMRILVFPEGTCTNGRALIKFKPGA
FIAGVPVQPLLIRYPNRLNTTSLGVFWMTLSQIYTNVEKPEEEKDPSLFA
DNVQKVMADALGIPATAYEFE-
>564268330_vertebrates_Alligator_mississippiensis__PREDICTED__lysophosphatidylcholine_acyltransferase_1_
EAAILTVAPHSSYFDVMKAESKDIPVWGIIIFPEGTCTNRSCLITFKPGA
FIPGVPVQPVVLRYPNKLDTITMEILWLTLCQFHNSVESEEERKNPSLYA
NNVRRVMAEALRVPVTDYTFED
>564268579_vertebrates_Alligator_mississippiensis__PREDICTED__lysophosphatidylcholine_acyltransferase_2_
EAPIFAVAPHSSFFDVSRPENLAAPIFGILIFPEGTCTNRSCLITFKQGA
FIPGVPVQPVLLRYPNRIDTVTKQLCAMTMCQPFTRVETEEEKKDPVLFA
NRVRNNMATALNVPTTDHTFED
>572300575_bees_Apis_dorsata__PREDICTED__1_acylglycerophosphocholine_O_acyltransferase_1_like_isoform_X1_
DAPVLALAPHSSFFDVAKAEIGRIPFFGVMIFPEGTCTNRSCLITFKSGA
FYPGVPVQPVCIRYPNKLDTVTLKLLWLTLTQLNSSCESEAEKTDPKLYA
NNVRRLMAEALQIPVSDYTYDD
>572300579_bees_Apis_dorsata__PREDICTED__1_acylglycerophosphocholine_O_acyltransferase_1_like_isoform_X3_
EAPILVVAPHSTFMDIVRRESGLNPFIGVMIFPEGTCTNRSCLITFKSGA
FYPGVPVQPVCIRYPNKLDTVTLKLLWLTLTQLNSSCESEAEKTDPKLYA
NNVRRLMAEALQIPVSDYTYDD
>586989424_carnivores_Felis_catus__PREDICTED__lysophospholipid_acyltransferase_LPCAT4_
QAPVLVAAPHSTFFDVSRAENLSVPVIGVLFFPEGTCSNKKALLKFKPGA
FIAGVPVQPVLIRYPNSLDTTSLKVLWLTASQPCSIVDSPEESRNPTLYA

NNVQRVMAQALGIPATECEF--
>587014795_carnivores_Felis_catus__PREDICTED__lysophosphatidylcholine_acyltransferase_2_isoform_X3_
EAPIFVAAPHSTFFDVSRNENAQVPLIGILVFPEGTCTNRSCLITFKPGA
FIPGVPVQPILLRYPNKLDTVTIQLCMLTFCQLFTKVENDEEKSDPILFA
CRVRSLMAEALGIPVTDHTYED
>593718724_whales__dolphins_Physeter_catodon__PREDICTED__lysophosphatidylcholine_acyltransferase_1_
EAAILTLAPHSSYFDVMKAESRDIPIWGIMIFPEGTCTNRTCLITFKPGA
FIPGVPVQPVVLRYPNKLDTITLKILWLTLCQFHNQVESEEEKEDPTLYA
SNVRHVMAEALGVSVTDYTFED
>593729048_whales__dolphins_Physeter_catodon__PREDICTED__lysophosphatidylcholine_acyltransferase_2_isoform_X1_
EAPIFVVAPHSTFFDVSRHENVQVPLIGILVFPEGTCTNRSCLITFKPGA
FIPGVPVQPVLLRYPNKLDTVTVQLCVLTCCQPFTRVENDEEKSDPVLFA
SRVRNLMAEALRIPVTDHTYED
>593744866_whales__dolphins_Physeter_catodon__PREDICTED__lysophospholipid_acyltransferase_LPCAT4_isoform_X1_
QAPVLVAAPHSTFFDVSRAENLSVPVIGVLFFPEGTCSNKKALLKFKPGA
FIAGVPVQPVLIRYPNSLDTTSLKVLWLTASQPCSIVDSPEESRDPTLYA
NNVQRVMAQALGIPATECEF--
>593744868_whales__dolphins_Physeter_catodon__PREDICTED__lysophospholipid_acyltransferase_LPCAT4_isoform_X2_
QAPVLVAAPHSTFFDVSRAENLSVPVIGVLFFPEGTCSNKKALLKFKPGA
FIAGVPVQPVLIRYPNSLDTTSLKVLWLTASQPCSIVDSPEESRDPTLYA
NNVQRVMAQALGIPATECEF--
>602657954_snakes_Python_bivittatus__PREDICTED__lysophosphatidylcholine_acyltransferase_1__partial_
EAAILTLAPHSSYFDVMKAESKDIPVWGIMIFPEGTCTNRSCLITFKPGA
FIPGVPVQPVILRYPNKLDTITLTILWLTLCQFHNFVESEEEKKNPSLYA
NNVRRVMAEALGVSVTDYTFED
>617299356_bony_fishes_Poecilia_formosa__PREDICTED__lysophosphatidylcholine_acyltransferase_1_

EAPIITLAPHSSYFDVMKAESKDIPVWGVMIFPEGTCTNRSCLITFKPGA
FIPGVPVQPVVIRYPNKLKLL-FKILWLTLCQLHNEFVSEEEKRNPALFA
VNVRRVMAKALGVPITDYSFED
>617400599_bony_fishes_Poecilia_formosa__PREDICTED__lysophosphatidylcholine_acyltransferase_2_
EAPILAVAPHSAFFDVSRVENLATPVFGVLIFPEGTCTNRSCLITFKQGA
FIPGVPVQPVLLRYPNKMDTVTIRVLLLTLCQLYTTVEKEDEKKCPSLFA
SRVRDTMAKALGVPVTDHTYED
>617437061_bony_fishes_Poecilia_formosa__PREDICTED__lysophosphatidylcholine_acyltransferase_1_like_
EVPILTVAPHSSYFDVAKLESQSVPVWGIMIFPEGTCTNRSGLILFKAGA
FIPGLPVQPVVLRYPNKLDTFSFKLLWLTLCQPHNSIESAKERENPALFA
SNVRKLMAEALEVPLTDLSFDD
>620975450_monotremes_Ornithorhynchus_anatinus__PREDICTED__lysophosphatidylcholine_acyltransferase_1_
EAAIFTLAPHSSYFDVMKAESRDIPVWGIMIFPEGTCTNRTCLITFKPGA
FIPGVPVQPVILRYPNKLDTITLKILWLTLCQFHNFVESEEEKRNPALYA
KNVRRVMAEALGVSITDYTYED
>620982124_monotremes_Ornithorhynchus_anatinus__PREDICTED__LOW_QUALITY_PROTEIN__lysophospholipid_acyltransferase_LPCAT4__partial_
---------------QLQRE----PVTGVLFFPEGTCSNKKALLKFKPGA
FIAGVPVQPVLIRYPNSVDTTSLKVLWLTASQPCSIVESPEESANPTLYA
SNVQRVMAQALGIPATECEF--
>632937816_chimaeras_Callorhinchus_milii__PREDICTED__lysophosphatidylcholine_acyltransferase_1_
EAPIMTLAPHSSYFDVMKTESKDIPVWGVMIFPEGTCTNRTCLITFKPGA
FIPGVPVQPVVLRYPNKMDTITLKILWLTLCQLHNKVESEEEKKNPILYA
NNVRKIMAKALGVPVTDYTFED
>641666842_aphids_Acyrthosiphon_pisum__PREDICTED__LOW_QUALITY_PROTEIN__1_acylglycerophosphocholine_O_acyltransferase_1_
EAPLLVIAPHSTFLDIARIEDGLSPWVGILIFPEGTCTNRSCLITFKPGA
FYPAVPVQPVLLRYPNKLDTVTLKLLWLTMTQPTTTVESEYEKQNPKAFA

EGVRNVMAKALAVPTADYTYDD
>641695175_bats_Eptesicus_fuscus__PREDICTED__lysophosphatidylcholine_acyltransferase_1_
EAAIFTLAPHSSYFDVMKAESRDIPIWGIMIFPEGTCTNRTCLITFKPGA
FIPGVPVQPVVLRYPNKLDTITLEILWLTLCQFHNQIESEEEKKDPALYA
SNVRRVMAEALGVSVTDYTFED
>641704728_bats_Eptesicus_fuscus__PREDICTED__lysophospholipid_acyltransferase_LPCAT4_
QAPVLVAAPHSTFFDVSRAENLSVPVIGVLFFPEGTCSNKKALLKFKPGA
FIAGVPVQPILIRYPNSLDTTSLKVLWLTASQPCSIVDSPEESRDPTRYA
NNVQRVMAQALGIPATECEF--
>641721722_bats_Eptesicus_fuscus__PREDICTED__lysophosphatidylcholine_acyltransferase_2_
EAPIFVVAPHSTFFDVSRNENVQVPLIGILVFPEGTCTNRSCLITFKPGA
FIPGVPVQPVLLRYPNQLDTVTFQLCVLTFCQPFTRVENDEEKRDPVLYA
GRVRNLMAEALGIPVTDHTYED
>641725954_bats_Eptesicus_fuscus__PREDICTED__lysophosphatidylcholine_acyltransferase_2B_like_
EAPIFVTAPHSTFFDVSASQNARIPVAGIMIFPEGVCTNRTCLITFKLGA
FSPGVPVQPVLLRYPNRLDTVTFQLCMLTLSQPFTRVESDQEKKDPILFA
NAVRVNMANALGVPVTDHTYED
>642915531_beetles_Tribolium_castaneum__PREDICTED__1_acylglycerophosphocholine_O_acyltransferase_1_isoform_X2_
EAPVVALAPHSSFMDVAKGETASIPFFGILIFPEGTCTNRSCLITFKPGA
FYPGVPIQPVCIRYPNKLDTVTLKLLWLTLTQPYSYCESEEEKKDPKLFA
NNVRAVMAKALGVPVSDYTYGD
>645028269_wasps_c._Nasonia_vitripennis__PREDICTED__1_acylglycerophosphocholine_O_acyltransferase_1_isoform_X2_
EAPILALAPHSSFFDVAKAETGRIPFFGVMIFPEGTCTNRSCLITFKSGA
FYPGVPVQPVCIRYPNKLDTVTLKLLWLTLTQLNSSCESEAEKLDPKLYA
NNVRRLMAEALQIPVSDYTYDD
>646699140_termites_Zootermopsis_nevadensis__hypothetical_protein_L798_00358_

EAPILVVAPHSTFLDIVRRESGLNPFIGVLIFPEGTCTNRSCLITFKPGA
FYPGVPIQPVCIRYPNKLDTVTLKLLWLTLTQVHSSCENEDEKQDPKLYA
NNVRRLMAKHLGIPVSDYTYDD
>649115777_other_sequences_synthetic_construct__LPCAT4__partial_
QAPVLVAAPHSTFFDVSRAENLSVPVIGVLFFPEGTCSNKKALLKFKPGA
FIAGVPVQPVLIRYPNSLDTTSLKVLWLTASQPCSIVDSPEESRDPTLYA
NNVQRVMAQALGIPATECEF--
>655899270_rabbits__hares_Oryctolagus_cuniculus__PREDICTED__lysophosphatidylcholine_acyltransferase_1_
EAAVLTLAPHSSYFDVMKAESRDIPIWGIMIFPEGTCTNRSCLITFKPGA
FIPGVPVQPVVLRYPNKLDTITLKILWLTLCQFHNQVESEEEKRDPALFA
SNVRRVMAEALGVSVTDYTFED
>662188606_psyllids_Diaphorina_citri__PREDICTED__lysophosphatidylcholine_acyltransferase_1_like_
---------------ELRKIP-------VLIFPEGTCTNRSCLITFKPGA
FYPGVPVQPVLLRYPNKLDTVTWKLLWLTLTQPHSNCESEEEKNDPKLYA
SNVRAVMARALGVPTSDYSYED
>664729378_odd-toed_ungulates_Equus_przewalskii__PREDICTED__lysophosphatidylcholine_acyltransferase_2_
EAPIFVVAPHSTFFDVSRNENAQAPLVGMLVFPEGTCTNRSCLITFKPGA
FIPGVPVQPILLRYPNKLDTVTIQLCMLTFCQPFTRVENEEERNDPVLFA
DRVRNLMAQALGIPVTDHTYED
>664730876_odd-toed_ungulates_Equus_przewalskii__PREDICTED__lysophosphatidylcholine_acyltransferase_2B_like_
EAPIFVTAPHSTFFDVSASQNALIPVAGILIFPEGVCTNRTCLVTFKLGA
FSPGVPVQPVLLRYPNTLDTVTFQACMLTLSQLFTRVENDQEKKDPVLFA
NTVRINMANALGVPVTDHTYED
>664769693_odd-toed_ungulates_Equus_przewalskii__PREDICTED__lysophosphatidylcholine_acyltransferase_1_isoform_X1_
EAAILTLAPHSSYFDVMKAESRDIPIWGIMIFPEGTCTNRTCLITFKPGA
FIPGVPVQPVVLRYPNKLDTITLKILWLTLCQFHNQVESEAEKKDPVLYA

SNVRRVMAAALGVPVTDYTYED
>664769695_odd-toed_ungulates_Equus_przewalskii__PREDICTED__lysophosphatidylcholine_acyltransferase_1_isoform_X2_
EAAILTLAPHSSYFDVMKAESRDIPIWGIMIFPEGTCTNRTCLITFKPGA
FIPGVPVQPVVLRYPNKLDTITLKILWLTLCQFHNQVESEAEKKDPVLYA
SNVRRVMAAALGVPVTDYTYED
>672141675_monocots_Phoenix_dactylifera__PREDICTED__lysophospholipid_acyltransferase_LPEAT2_isoform_X1_
-VAPIVVANHISYIEVASESHDALPFVGVLLFPEGTTTNGRSLISFQLGA
FIHGFPLQPVVVRFPHVHFDQSMKLFFRMFTQFHNFMELEKQ-KNAVHFA
ERTGYAMASALNVVQTSHSYGD
>672141677_monocots_Phoenix_dactylifera__PREDICTED__lysophospholipid_acyltransferase_LPEAT2_isoform_X2_
-VAPIVVANHISYIEVASESHDALPFVGVLLFPEGTTTNGRSLISFQLGA
FIHGFPLQPVVVRFPHVHFDQSMKLFFRMFTQFHNFMELEKQ-KNAVHFA
ERTGYAMASALNVVQTSHSYGD
>673014826_oomycetes_Aphanomyces_invadans__hypothetical_protein_H310_00380_
VLTQLIVANHIGFIDAVRGDMGNAPIIGLLIFPEGTTSSQDYLTKFKKGA
FAAGLPVQPVLLKYPFRYFDISGYLLFRMLCQVYMPMESPTEQSSPDLFA
ENVRQYMAKPMNAKCTNHTFED
>152070938_g-proteobacteria_Beggiatoa_sp.__Phospholipid_glycerol_acyltransferase_
----CFIR-HFS---LSKQEIQNWPILGVLFFPEGTSTDGTNVRRFHARL
FQAAIDVQPIALRY-RQGENISLGNLWRVLGETGLRVE--HE-KKRRDLS
DYAQ------------------
>489546793_g-proteobacteria_Pseudomonas_pseudoalcaligenes__1_acyl_sn_glycerol_3_phosphate_acyltransferase_
---MLWVSNHVSWTDLSKAEVRAWPVAGLLIFPEGTTTDGSLVKTFHSRL
LTSAVDVQPVAIRYLRDGRR-DLSHLLRVLRSEVAEVE--GE-LGRTLLA
RKA-------------------
>610424756_CFB_group_bacteria_Draconibacterium_orientale__hypothetical_protein_FH5T_11970_

SGNYILMPNHRSYIDVGKAEIAKWPFVAVILFPEGTTYKGPLTKKFKNGS
FKIAADVIPMAIDFKDVNDA--VGHFFRQLGKPFTHVT-----NDHKQLQ
EKTK------------------

Supplementary Data S6. The edited multiple sequence alignment for the phylogenetic tree reconstruction of Ek
>815807516_ants_Linepithema_humile-PREDICTED__ethanolamine_kinase_1_
--ITIAIIIPWLFFTDGRTNLVILIRVYGNSDLLIDREIILHIYFNGFAFLGTLVVIAMKMHL-LWTFMIMPQLEYMTLPVVFHNDLLGNVLVFIDYEYLNFDIVNHFTEAGEPDYLYPEQWLKYLSEVNLV
TKFTPMPHFFWACWALIQSSIDFDFLEYAAIRFYK-
>646691503_termites_Zootermopsis_nevadensis-Ethanolamine_kinase_
VILTISVLIPWF-FTDGITNLLVLVRVYGKTDLLIDRETLLPLYFNGLAYVGILCVVAMLMHVNVWIFLIMPQLEYLELPIVFHNDLLANIIVFIDYEYYNFDIGNHFAEAGDVDYAYPQQWLRYLTSISLV
NKFSLASHFLWAIWALIQASIDFDFLGYADIRLYK-
>924564623_flies_Drosophila_busckii-eas_
RVIFVAILIPWVFFTDGITNLVVLVRVYGKTDLLIDRETLLPLYFNGLVYVGTLVIVAMEMHVKIWTFLLVPHLEFLYLPIVFHNDLLGNVIVFIDYEYYNFDIGNHFAECGEVDYRYPRQWLRYLQEVELV
NQFALAAHIFWTVWSLLQASIDFDYVGYAFLRYYK-
>919033837_brachiopods_Lingula_anatina-PREDICTED__ethanolamine_kinase_1_like_
LVMKIALLVPWI-FTDGITNLVVLVRIYGNTELFIDRELLLPVYFNGLCFVGVGVVIAMRIHINVFIWLLVPDLELLPLPVVFHNDILANLVVFIDYEYYNFDIGDHFCEAGDVDYLYPKQWLRYLTDVELV
NKFALLAHFFWGVWAILQASIEFDFVQYAAQRFYKF
>291234579_hemichordates_Saccoglossus_kowalevskii-PREDICTED__ethanolamine_kinase_1_like_
-LVTVALMIPWILFTDGITNIFVLLRVFGKTELIIDREIILALYFNGLCFIGILVVIAMKMHIPLFLWYNCPNLEVLALPIVFHNDLLANIIVFVDYEYFNFDIADHFAEAGEVDYRYPKQWLHYLTDTELV
NKFVLACHLFWGLWALIQSSIDFDFLGYGIERLYKF
>551511971_bony_fishes_Xiphophorus_maculatus-PREDICTED__ethanolamine_kinase_1_like_
-LVAVILLLPWIMFTEGITNLIVLVRLYGMTELYVNREVVFPIYFNGICFVGVLLIIAMKIHIPLWMFLLVKILEMLHLPTVLHNDLLKNIIVFIDYEYYNFDIGNHFNEAGDVDYLYPQQWLTYLTEVTLV
CKFSLASNFFWGLWAILQSSIDFDFQRYAIARLYKY
>927120595_snakes_Thamnophis_sirtalis-PREDICTED__ethanolamine_kinase_2_
-VIAVALILPWVTFTEGITNLMVLVRVYGKTELIVDRELVLPLFFNGYCFMGALIMVAMKMHITLWLYLIVKNLEIMHLPIVFHNDLLKNIIVFIDYEYYNYDIGNHFNEAGEVNYLYPKQWLRYLSELELV
NQFALASHFLWACWGLIQASIDFDFARYADIRFYKV
>543741010_birds_Columba_livia-PREDICTED__ethanolamine_kinase_1_
-VITVALLLPWVLFTDGITNLIVLVRIYGKTELLVDREVVLPLYFNGLCFMGALVIIALKIHIALWMYFLIPNLEMMRLPVVLHNDLLKNIIVFIDYEYYNYDIGNHFNEAGEVDYLYPRQWLRYLSEVELV
NQFALASHFFWGLWALIQASIDFDFLGYAIVRFYKV
>507978828_insectivores_Condylura_cristata-PREDICTED__ethanolamine_kinase_1_isoform_X2_

-VVTVALLLPWVLFTDGITNLIVLVRIYGKTELLVDREVVLPLYFNGLCFIGALVIIALKIHIALWMYFLIPNLEMMSLPVVLHNDLLKNIIVFIDYEYYNYDIGNHFNEAGDVDYLYPRQWLRYLSEVELV
NQFALASHFFWGLWALIQASIDFDFLGYAIVRFYKV
>548467300_even-toed_ungulates_Capra_hircus-PREDICTED__ethanolamine_kinase_1_
-VVTVALLLPWVLFTDGITNLIVLVRIYGKTELLVDREVVLPLYFNGLCFIGALVIIALKIHIALWMYFLIPNLEMMILPVVLHNDLLKNIIVFIDYEYYNYDIGNHFNEAGDVDYLYPRQWLRYLTEVELV
NQFALASHFFWGLWALIQASIDFDFLGYAVVRFYKV
>852774793_rodents_Dipodomys_ordii-PREDICTED__ethanolamine_kinase_1_
-VVTVALLLPWVLFTDGITNLIVLVRIYGKTELLVDREVVLPLYFNGLCFIGALVIIALKIHIALWMYFLIPNLEMLILPVVLHNDLLKNIIVFIDYEYYNYDIGNHFNEAGDVDYLYPEQWLRYLTDVELV
NQFALASHFFWGLWALIQASIEFDFLGYAVVRFYKV
>504165381_rabbits___hares_Ochotona_princeps-PREDICTED__ethanolamine_kinase_1_
-VVTVALLLPWVLFTDGITNLIVLVRIYGKTELLVDREVVLPLYFNGLCFIGALVIIALKIHIALWMYFLIPNLEMMILPVVLHNDLLKNIIVFIDYEYYNYDIGNHFNEAGDVDYLYPRQWLRYLTEVELV
NQFALASHFFWGLWALIQASIEFDFLGYAIVRFYKV
>488513335_placentals_Dasypus_novemcinctus-PREDICTED__ethanolamine_kinase_1_
-VVTVALLLPWVLFTDGITNLIVLVRIYGKTELLVDREVVLPLYFNGLCFIGALVIIALKIHIALWMYFLIPNLEMMILPVVLHNDLLKNIIVFIDYEYYNYDIGNHFNEAGDVDYLYPRQWLRYLTEVELV
NRFALASHFFWGLWALIQASIEFDFLGYAIVRFYKV
>634864823_placentals_Orycteropus_afer-PREDICTED__ethanolamine_kinase_1_
-VVTVALLLPWVLFTDGITNLIVLVRIYGKTELLVDREVVLPLYFNGLCFIGALVIIALKIHIALWMYFLIPNLEMMILPVVLHNDLLKNIIVFIDYEYYNYDIGNHFNEAGDVDYLYPRQWLRYLTEVELV
NQFALASHFFWGLWALIQASIEFDFLGYAIVRFYKV
>10092615_primates_Homo_sapiens-ethanolamine_kinase_1_isoform_A_
VVVTVALLLPWVLFTDGITNLIVLVRIYGKTELLVDREVVLPLYFNGLCFIGALVIIALKIHIALWMYFLIPNLEMMILPVVLHNDLLKNIIVFIDYEYYNYDIGNHFNEAGDVDYLYPRQWLRYLTEVELV
NQFALASHFFWGLWALIQASIEFDFLGYAIVRFYKV
>918816358_ascomycetes_Aureobasidium_namibiae-choline_ethanolamine_kinase_
L--CYALILPWVLFTEGITNLMILLRAYGGTDVLIDRETLLPLFFNGLLYIGVCLVVALQWHTPLWMWIALP-LELLELDYIFHCDLLGNVIVFIDYEYPAFDIANHFAEGGDCDYALPRRFIAYLTEQQLI
DNFRGLPGFYWGIWSLIQASIDFDYASYADIRLYKQ
>666865203_ascomycetes_Scedosporium_apiospermum-Choline_ethanolamine_
ILLAYALVVPWVFCTDGITNLLILLRAYGGTHVIIDREALLPLLFNGMLYIGVALIVALEWHTPLWMWIALPKLELLDL-LVFHCDLLGNVIVFIDYEYPSFDIANHFAEAGECKYRLPKRFIRYVVEAQLV
DLYRGIPGFYWGIWALIQASIDFDYATYAELRLYKA

>512188910_ascomycetes_Ophiostoma_piceae-ethanolamine_kinase_
-LLQYALVLPWVFFTDGITNLLILLRAYGGTDVIIDREALLPLLFNGMLFIGPTLIIALQWHIPIWMWIALPELELLNL-LVFHNDLLGNVIVFIDYEYPSFDIANHFAEGGDCDMVIPVRFIGYIIEIALV
DVFRGLPGFYWGIWASIQASIDFDYASYAETRLYKT
>780591877_ascomycetes_Sporothrix_schenckii-ethanolamine_kinase_
-VLRYALVLPWVFFTDGITNLLLLLRAYGGTDLIIDREALLPLLFNGMLFIGVTIIIALQWHTPIWMWIALPCLELLSL-LVFHNDLLGNIIVFIDYEYPAFDIANHFAEGGDCNMVLPVRFIDYILEKAMV
DVFRGVPGFYWGIWALIQASIDFDYATYAEARLYKV
>923150023_ascomycetes_Madurella_mycetomatis-Ethanolamine_kinase_1_
YILSYALVIPWVFFTDGITNLLILLRAYGGTDVIIDRETLLPLLFNGMMFIGVTLIVALQWHVPVWMWIALPQLELLEL-LVFHCDLLGNVIVFIDYEYPSFDLANHFAEGGECDFVLPCRFIAYVKEVKLV
DHFRGVPGFYWGIWALIQASIDFDYASYAETRLYRV
>636749939_ascomycetes_Glarea_lozoyensis-Protein_kinase_like__PK_like__
FILSYALILPWIFFTDGITNLLVLLRAYGGTDLIIDRETLLPLLFNGMLFIGVTLIVALEWHVPVWMWIALPELELIEF-LVFHCDLLGNVIVFIDYEYPSFDIANHFAEGGDCEHLLPKRFIRYIEEIRLV
DVWRGIPGFYWGIWALIQASIDFDYASYAEVRLYRG
>661189246_fungi_Lichtheimia_corymbifera-ethanolamine_kinase_2_
-TYEVAIVIPWL-VQDGITNLVVLVRAFGDSEILIDREITLPLYFNGLMFIGVSLEIALKWHVPLWLWIEVPQMEMLLLPVVFHNDLLGNIIAFIDYEYYAFDIGNHFNEASDCDYKYPKQWFEYLSELEVV
EGFSLASHYYWGLWALVQASIDFDYLNYAILRFYKI
>672821385_fungi_Mortierella_verticillata-hypothetical_protein_MVEG_08380_
-IMVVAVMLPWIYFKDGITNLIVLVRAYGKSEVIIDREIILPLFFNGLACVGVFMKVAAIWHTILFIWIEVPQLELLHLPVVFHNDLLGNLIAFIDYEYYSFDLGNHFNEAGECEYNYPKQWLRYLRDLDLV
NKFALASHFYWGVWALVQASIDFDYMPYAVLRFYRF
>470243980_cellular_slime_molds_Dictyostelium_fasciculatum-ethanolamine_kinase_B_
VIY--LVMLDFF-LVGGVTNLFIIIRLYGASENFIDRESLLPFYFNGCIFVGQLLIIATKWHMLTFLWISTKSEYLFSRHIVFHNDLIRNMIVYIDFEYYNFDLGNFFCESGDLDYRYPVQFIRYLDEVHLA
NHFTLASHLMWGFWGII-SGIDFDYIDYANKRFYKV
>281206759_cellular_slime_molds_Polysphondylium_pallidum-ethanolamine_kinase_B_
-----LVILDFIFMTGGVTNLFIIIRLYGGSENFIDRESLLPFYFNGCIFVGQLLIIATKWHLLLLLWINVKTYETFFTPINFHNDLIRNMIVYIDFEYYNFDIGNFFCESGDLDYRYPMQFINYLTEIHLA
NHFTLGSHLMWGFWGIVQNSIEFDYIGYALKRFYKV
>831780967_cellular_slime_molds_Acytostelium_subglobosum-hypothetical_protein_SAMD00019534_042480_
---IALVILD-DLMTGGVTNLFIIIRLYGGTENFIDRESLLPFYFNGCIFVGQLLIIASTWH-DLTLWVNVQTYETFFKPVVFHNDLIRNMIVYIDFEYYNFDIGNFFCESGDLDYKYPVQFLRYLTEVHLA

NHFTLGSHLMWGFWGIVQNSIDFDYIGYALKRFYKV

>123474064_trichomonads_Trichomonas_vaginalis-Choline_ethanolamine_kinase_family_protein_

-INGMILILK-ILMAGGITNVY-IVRIYGNTEQIIDREQ--IIYFNGMVFQGTIMILALLLHSYVFIFIKTNKLEILN-PLALHNDLLGNILVFVDYEYYTYDIANHFLECGELDLRFPYQFIKYLPEVEWV

DKLVHLSHLFWGSWAFFQASVNFPYFEYGLWRLFLL

>857974265_cercozoans_Plasmodiophora_brassicae-hypothetical_protein_PBRA_007433_

-----T---P---VV-----VV------GPVPDVADRDALLKVYFNGRVWLGALLVVAMALHVIIWVYMEADTWAGLLLPVVLHNDLLGNIIIFIDFEYYNFDIANHWCEAGDCDWKFPPQFARYLTDVRLV

RPWPLVSHLVWAFWGIVQASIDFDFIGYARQRFFLA

>669199557_apicomplexans_Plasmodium_vinckei-ethanolamine_kinase_

SV-EKDILEEELFINGGITNLVYLIRLYGKTSEIINREKILKIYFNGRIFMGALIFIALVLHILLWIYFLLYPLTIVLCPIVLHCDLLSNFIILIDFEYPMYDIANHFNEAGNCEWLIPKEFIKYL-IINLV

QPFYLISHIYWALWSLLQGSIDFDFINYGMTKLSIF

>697888986_apicomplexans_Theileria_orientalis-choline_ethanolamine_kinase_

-ILSDCYCLAYLVVFGGITNLYVLVRIFGHTSRIIDRERLLKVYFEGQIWIGNLLYIALKLHIILWLYYLCQKLKILVCPLVLHCDLLGNIIVFIDFEYCMYDIANHFNEMGTGDFLIPLQFIRYLSAVDLI

QPFFMASHIVWGIWSLLQSSVEFDFLRYAKKRIFSF

>156086690_apicomplexans_Babesia_bovis-choline_ethanolamine_kinase_

LI-AHGLS--LV-IVGGWTNLYVAVRIFGQTERFIDRERHLKVYFGGQIWLGVVFYIALKLHTGLWVFYLCLVIVILVCPVVLHCDLLGNVLVFLDYEYFMFDIANHFIECGECNWRIPEQFIKYI-AIELI

QPFFMAANIFWGLWGLLQCSHDIDFKRYANFRIAHR

>237842237_apicomplexans_Toxoplasma_gondii-choline_ethanolamine_kinase_domain_containing_protein_

AVLSEAIVLAGLAVEVGSTNMVCAVKFFGHTGKYICRELLLKIFFGGLIWLGSLL-IAMRMHIALWLFLLCKRVRLLLAPVVLHGDLLGNIIVFIDFDYFMFDIANHFAESGECDFRCPERFLRYLLEVALI

NVFFPLSNILWGLWALIQAVREMNYWRFAFDRLAPS

>325188595_oomycetes_Albugo_laibachii-ethanolamine_kinase_putative_

FYFAVIVVLPWIIVSGGITNLYLLVRIYGNTEILIDRENKLPYYFNGRIWLAPLMLIAVKMHMLLWLFVLAMQWKALSSAVVFHNDLLGNILVIIDYEYYNFDIANHFCECGEMDLQYPMQFFDYFRFYKFV

NKFAMASHLFWAFWAIVQASIDFDFLDYAHKRFFRF

>301109172_oomycetes_Phytophthora_infestans-ethanolamine_kinase__putative_

VLYAVAVVIPWIVIVGGITNLYVLVRLYGHTEEFIDRENLLPYYFNGRVWLAPLMYIGLIMHMILWIFELAMNLKLLVPVIVFHNDLLGNILVIIDYEYYNFDFANHFCECGELDLLYPIQFFKYMSFFHLV

NRYALASHLFWGYWALVQASIDFDFFEYAAKRFFRF

>698777663_oomycetes_Aphanomyces_astaci-hypothetical_protein__variant_

VLYSVCVAIPFIIICGGITNLYVLLRIYGHTEVFIDRDNELPYHFNGRIWVGPLMLIAVKMHMMLWIFELASQIRVAIPTSVFHNDMLGNVLVVIDYEYYNYDFANHFCECGDMNLAYPQQFYKYLLFVALG
NLYALASHLFWGLWAVVQASIEFDFLEYARVRLFAF
>669156808_oomycetes_Saprolegnia_diclina-hypothetical_protein_SDRG_09792_
-LTVYCVVIPWIVICGGITNLYVLVRLYGHTEVFIDRDNELPYFFNGRVWVGALMLIPLKMHMILWIFELAARIRVLVLYSVFHNDLLGNILVIIDYEYYNFDFGNHFCECGDMNLDYPKQFYKYLLFLGLG
NVYALAAHMFWGLWAIVQASIDFDFLDYARMRFFLF
>937933352_monocots_Oryza_sativa-Os09g0438400__partial_
GRSAAHICLRWFIVSGGITNLLVTVRLYGNTDLVIDRELHLALLFNGMVFIATLMIIAIRFHVILWIFMKASQIEVLLSPVVFHNDLLGNLMLFIDFEYYSYDIANHFNEAGDCDYLYPKQFFRYLQDLDLT
NTYRLASHIYWALWALIQASIDFDYLGYFFLRYYRC
>195620822_monocots_Zea_mays-choline_ethanolamine_kinase_
VVISLILCLEWFIVSGGITNLLVTVRLYGNTDLVIDRELYLALLFNGVVFIATLMIIALKFHVILWIFLKAAQIEVLLLPVVYHNDLLGNLMLFIDFEYYSYDIANHFNEAGDCDYLYPKQFFRYLDDMELT
NTFRLASHIYWALWALIQASIDFDYLGYFFLRYYRC
>743774145_monocots_Elaeis_guineensis-PREDICTED__probable_ethanolamine_kinase_isoform_X2_
IIISLILCFRWFIVSGGITNLLLTVRLYGNTDLVIDRELYLALLFNGMVFIATLMIIALKFHVILWMFLKAAQIEILLTPVVFHNDLLGNLMLFIDFEYYSYDIANHFNEAGDCDYLYPKQFFRYLHDLELT
NTFRLASHIYWALWALIQASIDFDYLSYFFLRCYKC
>255566975_eudicots_Ricinus_communis-choline_ethanolamine_kinase__putative_
--------LKWFVVSG----VLITVRLYGNTDYVINRELYLALLFNGMVFIATLMLIALKFHVILWIFYNASQVEVILTPVVFHNDLLGNLMLFIDFEYYSFDIGNHFNEAGDCDYLYPKQFFRYLSDLELT
NTFMLASHLFWALWALIQASIEFDYLGYFFLRYYKS
>747074221_eudicots_Sesamum_indicum-PREDICTED__probable_ethanolamine_kinase_
IVHALILFLKWFLVSGGITNLLMTVRLFGNTEYVINRELHLALLFNGMVFIATLMLIALRFHVILWIFLKASQIELLITPVVYHNDLLGNLMLFIDFEYYNFDIGNHFNEAGDCDYLYPKQFFRYLTELELT
NTYMLASHLYWALWALIQASIDFDYLGYFFLRYYKC
>21593269_eudicots_Arabidopsis_thaliana-putative_choline_kinase_
IVTSLILCLKWFVVSGGITNLLVTVRLYGNTEYVINREIYLALLFNGMVFIATLMIIALKFHVILWIFYKASQLEILFTPVVFHNDLLGNFMLLIDFEYYNFDIGNHFNEAGDCDYLYPKQFIKYLSEVEVT
DAYKLASHLYWAIWAIIQASIEFEYLGYFFLRYYKT
>731407198_eudicots_Vitis_vinifera-PREDICTED__probable_ethanolamine_kinase_isoform_X2_
------FMNGW----DGWQG-LAFLIIH----LLV--ELYLALLFNGMVFIATLMLIALKFHVILWIFFKASQVEVLLTPVVFHNDLLGNLMLFIDFEYYSFDIGNHFNEAGDCDYLYPKQFFRYLSDLELA
NTFMLASHLYWALWALIQASIDFDYLGYYFLRYYKC

>168005868_mosses_Physcomitrella_patens-predicted_protein_
VIPRLVVCLRWIVISGGITNLLVTVRVFGNTDAVIDRELYLALLFNGMIFIGTLMLIAVRLHLILWIFIKASQIEILISPVVFHNDLLGNFMLIIDYEYHSYDIANYFNEAGDCDYLYPKQFFRYLTELEFC
NFYSLVSHMYWATWAIVQASIKFDYLGYFFLRFYKL
>168063767_mosses_Physcomitrella_patens-predicted_protein_
VVTRLAVCLRWIVITGGITNLLLTVRVFGNTDAVIDRELFLALLFNGMIYLGTLIFIAVRLHLILWIFIKGSQIEVIISPVVFHNDLLGNFMLIIDYEYHNYDIANYLNEAGDCDYLYPKQFYRYLTELELC
SFYSLASHLYWATWAIVQASIEFDYLGYFFQRFYNI
>302760153_vascular_plants_Selaginella_moellendorffii-hypothetical_protein_SELMODRAFT_438648_
LVPSLVVCLSWLIISGGITNLLVTVRIFGNTDAVIDRELHLALVFNGMIFIATLLVIALRLHLILWIFYKARQLDILISPIVFHNDLLGNIMLLIDFEYYSYDIGNHFNEAGECDYLYPKQFFRYLSELELT
NFYALVSHLYWAIWAIVQASINFDYLGYHCLRYYKV
>159109095_diplomonads_Giardia_lamblia-Ethanolamine_kinase__putative_
-VI--AIL-E-YYTLEGCSNVHYIVRF-TQSRFQ-NYERHVTQAFDGIVCIGIELYIAMRLHIVLLSYMKGRELEDLLVLLWTHNDLHGNIIVFIDWEYYSFDIACFFLETGDCEIAFPARFYRYF--SNLL
CLFFVLACLFWAAWSSGVDDKNRTRLGHAVLRAICK
>558596733_diplomonads_Spironucleus_salmonicida-Ethanolamine_kinase__putative_
-MN--IIEI--ILVS-GCTNNFYLLRRYLREEIFMDQEIYLVELFAFFIYIGLLLNIAFKLHHILFIFY---QIQIYAY-VCIHNDAHGNIIAFIDFEYVSYDIACFFAETGDAIYDYALRFYKYF-ENVIE
EILAIFQPFVWGLWSLWGSGL------YAKNRLF-Y
>475549664_monocots_Aegilops_tauschii-hypothetical_protein_F775_09005_
-R----LCLKWFIVSGGITNLLVTVRLYGNTDLVIDRELYLSMLYLQVYFCHCAMIIALKFHVILWVFLKASQIEVLLSPVVFHNDLLGNLMLFIDFEYYSFDIANHFNEAGECDYLYPKQFFRYLQDLDLT
NTFRLASHLYWALWALIQASIDFDYLGYFFLRLFKY
>695027911_monocots_Musa_acuminata-PREDICTED__probable_ethanolamine_kinase_
IVISLILCLKWFIISGGITNLLLTVRLYGNTDLVIDRELHLALLFNGMVFIATLMIIALKFHVILWIFLEAMQIEILLTPVVFHNDLLGNLMLFIDFEYYSYDIANHFNEAGDCDYLYPKQFFKYLPDLEIT
NTYRLASHIYWALWALIQASIDFDYLWYFFLRYYKC
>242044794_monocots_Sorghum_bicolor-hypothetical_protein_SORBIDRAFT_02g025680_
VVISLILCLIWFIVSGGITNLLVTVRLYGNTDLVIDRELYLALLFNGVVFIATLMIIALKFHVILWIFLKAAQIEVLLLPVVYHNDLLGNLMLFIDFEYYSYDIANHFNEAGDCDYLYPKQFFRYLQDMELT
NTFRLASHIYWALWALIQASIDFDYLGYFFLRYYRC
>902226724_eudicots_Spinacia_oleracea-hypothetical_protein_SOVF_052210_
VVTSLVLCIEWFVVAGGITNLLITVRLYGNTDYVVNRELYLALLFNGMVFIATLMLIALKFHVILWLFFKASQLEVLLTPVVFHNDLLGNLMLIIDFEYYNFDIGNHFNEAGDCDYLYPKQFFRYLPDLELT

NSFMLASHLLWALWALIQESIDFDYLSYFFMRFYKV
>731353359_eudicots_Beta_vulgaris-PREDICTED__probable_ethanolamine_kinase_
IVTSLILCIEWFVVSGGITNLLITVRLYGNTDYVINRELYLALLFNGMVFIATLMLIALRFHVILWLFFKASQIEVLLTPVVFHNDLLGNLMLIIDFEYYNFDIGNHFNEAGDCDYLYPKQFFRYLLDLELT
NSFMLVSHVYWALWALIQASIDFDYLGYFFMRYYKV
>661885770_eudicots_Coffea_canephora-unnamed_protein_product_
IIHSLILCLKWFVVSGGITNLLITVRLYGNTEYVINRELYLALLFNGMVFIATLMLIALKFHVILWIFYKASQIEILLTPVVYHNDLLGNLMLIIDFEYYSFDIGNHFNEAGDCDYLCPKQFFRYLSDLELT
NMYMLASHLYWALWALIQASIDFDYLGYFFLRYFKC
>697114263_eudicots_Nicotiana_tomentosiformis-PREDICTED__probable_ethanolamine_kinase_
IVHSLILCLNWFVVSGGITNLLMTIRLYGNTEYVINRELYLALLFNGMVFIATLMLIALKFHVILWVFFKASKVEILLTPVVFHNDLLGNLMLFIDFEYYNFDLGNHFNEAGDCDYLYPKQFFRYLSDLELT
SSYMLASHLYWALWALIQASIDFDYISYFFLRYYKC
>565404151_eudicots_Solanum_tuberosum-PREDICTED__probable_ethanolamine_kinase_like_
IVHSLILCLNWFVVSGGITNLLMTVRLYGNTEYVINREMHLALLFNGMVFIATLMLIALNFHVIVWVFFKASKVEILLTPVVFHNDLLGNLMLFIDFEYYNFDIGNHFNEAGDCDYLYPKQFFRYLSDLELT
SSYMLASHLYWALWALIQASIDFDYISYFFLRYYKV
>674239694_eudicots_Arabis_alpina-hypothetical_protein_AALP_AA6G244500_
IVTSLLLCLKWFVVSGGITNLLVTVRLYGNTEYVINRELYLALLFNGMVFIATLMIIALKFHVILWIFFRASHLEILFTPVVFHNDLLGNLMLLIDFEYYNFDIGNHFNEAGDCDYFYPKQFIKYLSEVEVT
DAYKLASHLYWAIWAIIQASIEFDYMGYFFLRYYKT
>590578052_eudicots_Theobroma_cacao-Kinase_superfamily_protein_isoform_1_
IVTALVLCLGWFVVSGGITNLLVTVRLYGNTEYVINRELYLALLFNGMVFIATLMLIALRFHVILWIFFKASQVEVLLTPVVFHNDLLGNLMLFIDFEYYNFDIGNHFNEAGDCDYLYPKQFFRYLSDLELT
NTFMLASHLYWALWAIIQASIDFDYLGYFFLRYYKC
>645275312_eudicots_Prunus_mume-PREDICTED__probable_ethanolamine_kinase_
IVPSLILCLKWFVVSGGITNLLVTVRLYGNTEYVINRELYLALLFNGMVFIATLMLIALRFHVILWLFFKASQVEILLTPVVFHNDLLGNIMLLIDFEYYNFDIGNHFNEAGECDFLCPKQFLRYLSDLELE
NTYTLASHLYWALWGLIQASINFDYLGYFFLRYFKC
>694386047_eudicots_Pyrus_x-PREDICTED__probable_ethanolamine_kinase_
IVPSLILCLKWFVISGGITNLLVTVRLYGNTDYVINRELYLALLFNGMVFIATLMLIALRFHVILWLFFKASQVEMLLTPVVFHNDLLGNIMLFIDFEYYNFDIGNHFNEAGECDYSCPKQFLRYLSELELT
NTYMLASHLYWAVWGIIQASINFDYLGYFFLRYYKC
>922390973_eudicots_Medicago_truncatula-choline_ethanolamine_kinase_

IIPSLVLCMKWFVISGGITNLLITVRLYGNTEHIIDRELYIAWLFNGIVFIATLMLIALKFHVILWIFFKASQVEILLSPVIFHNDLLGNIMLFIDYEYYNYDIGNHFAEAGECDYLYPMQFFRYLSDLKLV
NTYALASHLFWSLWGLIQASIDFDYLGYFFLRYYKL
>734411332_eudicots_Glycine_soja-Putative_ethanolamine_kinase_A_
IIPSLVLCMKWFVISGGITNLLITVRLYGNTEYIIDRELYIAWLFNGMVFIATLMLIALRFHVILWVFFKASQVEILLCPVIFHNDLLGNIMLFIDYEYYNYDIGDHFAEAGECDYLYPMQFLRYLSDLELA
NTFSLASHIFWALWGLIQASIEFDYLGYFFLRYYKY
>575480684_chytrids_Batrachochytrium_dendrobatidis-hypothetical_protein_BATDEDRAFT_89060_
LVIEIVVIVPWVLCTNGITNLVVLVRTYGGSSVLIDRELVLPLYFNGIVFTGVFMKVALIWHVLLFLWIAIPALDLLHLPVVFHCDLLGNIIVFIDYEYYSFDIANHFCEAGDCDWLYPEQWLSYWTQLNLT
LKFSLAAHFFWAVWALIQASLDFDYLDYAMLRLYKW
>907091063_chytrids_Spizellomyces_punctatus-hypothetical_protein_SPPG_07302_
---SFVVIFPWVFCTAGITNLVALVRCYGGSDILIDREMALPLYFNGLVYVGPFMKVALRWHVLLFLWLAVPDLDLLVLPVTFHNDLLGNIVVFIDYEYYSFDIGNHFCEAGDCDWLYPKQWLRYLVEVELV
AKFSLASHFYWAVWALVQASLDFDYLEYALMRLYR-
>511006955_fungi_Mucor_circinelloides-hypothetical_protein_HMPREF1544_04966_
-TIEVAVVVPWI-VQDGITNLVVLVRAYGGSELIIDREVTLPLYFNGLVFIGVSLTIALKWHVLLWMWLQVPQIELLELPIVFHNDLLGNIIAFIDYEYYAFDIGNHFNEAGECEYRYPKQWYDYLTEKELV
DGFSLASHYYWGLWAMIQASIDFNYMDYAVLRFYKV
>748504014_ascomycetes_Arthrobotrys_oligospora-hypothetical_protein_AOL_s00078g295_
-IHVFVLVLPYIVVTEGTTNLFALVKIYGGTETLIDREIILPLLFNGHAFLGPCIIVALQWHIPIWAWLAIPELDFLRLAFVLHGDLLGNIIVFIDYEHYCFDLANHFSETGDCDYLLPARFIRYLSEVSLV
DSYRGFPGFYWGLCAVIQTSIDFDYAGYAELRFYRE
>302887954_ascomycetes_Nectria_haematococca-hypothetical_protein_NECHADRAFT_37289_
-VQVFSLSLPALVLTQGTTNLYVLVKVYGGTDITIDRELLLSPLFNGHAFIGTCMIVALRWHTPVWAWLAIPSLQFVKLPLVLHGDLLGNIIVFIDYEHYCFELANHFAETGECDYLLPRRFIHYL--IALG
DHADI-----PGLCALIQASIDFDYAGYAEKRLYRV
>644995447_wasps__c._Nasonia_vitripennis-PREDICTED__LOW_QUALITY_PROTEIN__ethanolamine_kinase_1_
TFIFIAIVLPWH-FTNGISNLVVLIRVYGKTDLLIDREIILHLYFNGLAFLGTLIVVAMEMHLAIWTFMIMPQLEYLNLPVVFHNDLLGNILVFIDFEYFNFDIANHFAEAGDPDYLYPEQWLRYLSDIILV
NQFVLMTHFFWGCWALIQSSIDFDFLEYAALRFYKV
>817208021_hymenopterans_Orussus_abietinus-PREDICTED__ethanolamine_kinase_isoform_X1_
--ITIAVVIPWLFFTNGITNLVVLVRVYGKTDLLIDRETILHIYFNGLAFIGTLIVVAMQMHL-IWTFMIMPQLEYLKLPVVYHNDLLGNVLVFIDFEYYNYDIANHFAEAGGPDYLYPEQWLRYLSEVTLV
NKFVLLTHFFWGCWSLIQSSIDFDFLTYAFRRFYRC

>340729228_bees_Bombus_terrestris-PREDICTED__ethanolamine_kinase_1_
--ITIAIIIPWLFFTDGITNLIVLVRVYGKTDLLIDRETVLHIYFNGLAFIGILVIVAMQMHLEIWLFMIMPQLEYLELEVVFHNDLLGNVLVFIDFEYYNYDIANHFAEAGDPDYLYPEQWLNYLPEINLV
NKFVLLSHFFWGCWGLIQSSIDFDFLEYAAIRFYKC
>572318560_bees_Apis_dorsata-PREDICTED__ethanolamine_kinase_like_isoform_X1_
--IVVAIIIPWLFFTDGITNLIVLIRVYGKTDLLINRETILHIYFNGLAFIGILVIIAMQMHLEIWLFMIMPQLNYLELDIVFHNDLLGNILVFIDFEYYNFDIANHFAEAGNPDYLYPEQWLNYLLEINLV
NKFVLLSHFFWGCWALIQSSIDFDFLQ---------
>383852473_bees_Megachile_rotundata-PREDICTED__ethanolamine_kinase_1_
--ITIAVIIPWLFFTDGITNLIVLVRIYGKTDLLINRETILHLYFNGLAFIGTLIIIAMQMHL-IWMFMIMPQLNYLELEVVFHNDLLGNVLVFIDFEYYNYDIANHFAEAGNPDFLYPEQWLNYLPELNLV
NKFVLLSHFFWGCWGLIQSSIDFDFLEYAAIRFYK-
>768409835_moths_Plutella_xylostella-PREDICTED__ethanolamine_kinase_
VVVKIILLIPWF-FTDGITNLVVLVRIYGKTDLLIDRELTLPVYFNGLAYYGTLVIVAMKMHVLVWIFLLLPQLEFLYLPIVFHNDLLGNVIIFIDYEYYNFDIANHFNEVGDIDYRYPKRWIKYLPTVEVV
EQMSLASHFLWGVWSLVQYSIDFDFGRYAEIRLYKI
>357607247_butterflies_Danaus_plexippus-hypothetical_protein_KGM_05435_
VINQIIVLIPWY-FTDGITNLVLLVRIYGKTDLLIDREILLPIYFNGLVYYGTLVIVAMKMHVLIWIFLLIPQLEYLRLPLVFHNDLLGNVVVFIDYEYYNFDIANHFNEVGDIDYRYPEQWVHYLGQVSVV
RRLAPLSHFLWAVWALVQYSIHFDFLRYAEIRLYK-
>780008149_firmicutes_bacterium_UASB270-hypothetical_protein_
LLV---LFLPWIILSGGITNLYVALRIYGKTELFINREAKMPLILHVTIYIGTLFLIVIRIHSVVEMILDLN-ITILLAPYTAHNDLLENFIMIIDWEYMAYDLADMFQE------LVPREFVEY--EFDNI
DMFKPFPDIYWFLWSLIQQSIAFDFYNYGRVKYALE
>918378711_firmicutes_Robinsoniella_peoriensis-hypothetical_protein_
SILGTALFLDWMKVSGGMTNNFYILRIPGCTEVFIDREQIAPILFGVKVYIGTLAYTTLKLHSVVWYYKEILGLFFLDLDRRPHNDLVENFVMLIDWEYYNWDVAAHLLECE----F--KEYLQYL-PGREL
LMFQICQDVLWSAWTIAKEG-E-DFGAYGEGRFAKY
>501110649_firmicutes_Alkaliphilus_oremlandii-choline_kinase_
---MMIKLVQ-IFFAGGLTNNYYVIRQPGMTNYMIDREKIA-SQYTGIKYINNIPNVSMKTHSVFQLYEIVENLKLYTLVRIPHNDTVENFIMLIDWEYLNWDIAAYILESR-----LTDEFLEY---KEEI
KCFMLAQDLLWTVWALI-RN-GDDFLDYCHIRYFM-
>551177443_fusobacteria_Fusobacterium_russii-choline_kinase_
-IT--IILA--LIIQGGMTNNFYVLRVSGGTQGMINREKKISLIYGNKIYIGTLMNVSLRLHSVVFIYELANGVKTLKLRLVSHNDTVENFILLIDWEYMNWDLAAYSLENS----L--KEFLEYF--PKEV

QIFKILQDFLWSIWTLVKEGI--KFGDYGIRRFCLI
>924349927_firmicutes_Lysinibacillus_sp_-hypothetical_protein_
LVANYLSCVKWIKFSGGMTNNYLQIRIPGGTNSLVNREIAIPFYFGVKIYVDTLANIALKLHI-FIIYEVIITKKILKISLCPHNDLVENFIILIDWEYYNWDLAAFILESE----L--SEFLKYF-PLEEL
NFYKSVQDILWSLWTIIKEG-SFGNYG-----YYGL
>491799065_firmicutes_Granulicatella_adiacens-choline_kinase_
L-LPYQIAKEWVILKKGMTNSFYIMRIPGGTDHLINREAVLDVVNGYKIYLGSCK-CMLEFHLALWQYY---HTEILFVP-VLHIDSVDNFLILIDWEYMQIDIA-MFCIAG----Y--RALMDYF--EKTI
YAYMAVSGMLWSNWC--EYTLGVDFGEYSLMQYY-V
>739479301_g-proteobacteria_Salinisphaera_hydrothermalis-choline_kinase_
-----TIP-RLMALG-GLTNVYLVLRIPGGTEAYIDREEGAAVLFGLMLELDTMFAAGLRMHSVLFIYRVLSDARAIALELAPHNDPLENFLMIVDWEYMNWDVGDFSVEAG----L--PDLLRYL-PTAEM
ILYKAACDLLWTLWGLIQHG-AEDFWAYATERFCMD
>740059221_a-proteobacteria_Sulfitobacter_mediterraneus-choline_kinase_
-----VQLLPFE-ITGGLTNVHVIVRIPGGTEEYIDREIAAPVLEGVMIAVGTMFAAGLQLHSVLFIYLILSDVAAVALPLAPHCDPLENFLMIVDWEYMNWDVGDLSVEAE----M--AELLQYF-PTAEV
VIYKAMCDLLWTLWGLIQHG-AEDFWAYATGRFCMD
>751299615_a-proteobacteria_Mesorhizobium_plurifarium-choline_kinase_
--T--LIP-AYL---GGLTNVYVCLRIPGGTEEYINREAEAPVLFGLMAFIGTMFSAGFKLHSALLIYLVLSDVEAVALPLAAHCDPLENFLMIVDWEYMNWDLGDLSVEGR----F--TDLMRYF-ARPEV
VIYKAMCDLLWTLWGLIQLD-ADDFRAYADGRFCME
>495070065_a-proteobacteria_Pelagibaca_bermudensis-choline_kinase_
-----A--MPWIYMVGGLNNNWYFLKIPGGTEIFINRSYHAPVIDGTEVFLNTCFVVIFTLHGVIFIHLQARGLHYIAFDLVTYNDPMGNFLLLIDYEFANYDIGVFACE------MFLETLIEYF-VTPMV
SLCRVLADMKWGSWAVVNRTWDFDFQKYGLWKYGMR
>587639549_a-proteobacteria_Skermanella_stibiiresistens-choline_kinase_
-MA--IVA--WVYVLGGISNNWYFLKVPGGTEMFIDRAYQAPTFLDIEIFIGVSFVAVYRFHAPVFIHKQVAGLQYAALDLVPFNDPMGNFLILIDFEYNNYDLAIWSGE------MFFEDILEYF-FDQLL
MVHKVLADIKWGTWAMVQNSLDFDFYKYGIWKLAIR
>739511382_a-proteobacteria_Salipiger_mucosus-choline_kinase_
-----IMILS-LYVSGGISNNWWFVKVPGGTEMFIDRALKAPVYLGVEIFLDASFPAIYTMHLPVFIHAQVAGMNAAALDLVPFNDPMGNFMILIDYEYMNYDLGIWFGE------MFFPELIEYF-VRPIV
IVHKALADVKWALWSMVQLSLDFDFHKYGMWK-RED
>759735584_g-proteobacteria_Halotalea_alkalilenta-choline_kinase_

```
---MMEIY-PFIYVSGGISNNWYFVKLPGGTEMFIDRALKAPVHLGIEIFMTASFAAIYRFHL-VFIHEQVR-INYAALDLVPFNDPMGNFMILIDYEYMNYDLGIWFGE------MFFPELIEYF--TDLV
TIHKALADIKWAAWSMVQMSLEFDYFKYGAWKLFVA
>550981772_a-proteobacteria_Fodinicurvata_sediminis-choline_kinase_
--R--LAVIPWVYVNGGISNNWYFVKVPGGTEMFIDRAHKAAVYIGIEIFVGTSFVALLAFNQPVFIHLQVEGLQYAALDLVPMNDTLGNFMILVDFEYNNYELGLWFGE------MFFPELLEYF-D-DIL
NVGKALADLKWSTWAMVQSSLDFDFHKYGIWKHAMR
>640449480_b-proteobacteria_Burkholderia_andropogonis-choline_kinase_
--KRFIID--WIYVGGGISNNWYFFKVPGGTEMFINRAHKAAVLLGVEIFMGASFVALLAFNQLVLIHQQVEGLQFAALDLRPMNDTLGNFMILVDFEYNNYELALWFGE------MFFPELVEYF-VTSTI
AVNKALADLKWATWAMVQRSIDFDFYKYGAWKHAHR
>503276538_enterobacteria_Pantoea_sp_-choline_kinase_
--S--RQAASWIYVSGGISNNWYFVKIPGGTERFINRAHKAVVVLGVEVFIGASFIALLSFNQLVFLHIQVRQLQAAALDRVPMNDTLGNFMILVDFEYNNYELALWFGE------MFFPELLEYF-VDAIV
QIHKYLADMKWSTWAIIQHAIDFDFSKYGRWK-LSD
>736893068_enterobacteria_Erwinia_typographi-choline_kinase_
-MG--RKAAPWVYVSGGISNNWYFVKIPGGTERFINRAHKAAVVLGVEIFMGTSFVALLSFNQLVFLHIQVNGLQSAAIDKVPMNDTLGNFMILVDFEYNNYELALWFGE------MFFPELLEYF-D-NCV
QMHKFLADMKWSTWAIIQHAIDFDFSKYGAWKTALD
>488809703_a-proteobacteria_Rhodobacter_sphaeroides-choline_kinase_
---MTIVILR-MYVSGGISNNWWFVKIPGGTEMFIDRAFRAPVYLGVEIFMDPAFKAMYTLHLPVFIHAQAAGINEAALDLVPFNDPMGNFMILIDYEYMNYDFGLFFGE------MFFPELIEYF-VRPIV
IVHKALADVKWALWSMVQLSLAFDFHKYGMWKLF-D
>654517060_d-proteobacteria_delta_proteobacterium-hypothetical_protein__partial_
------VIIDWIFVTGGITNNWYFVKIPGGTEKFIDRCHIAPVFFGVEVWLGVLIKMILRFHVLIITMLMALNERMTQIDYAPHNDVYNNFVMLIDFEYMNYDMACWSCG------NYFEDIIRYC-YDEIF
KLYKILSEIKWSMWSCVQASVEADYIEWLNGKMLSY
>750144977_a-proteobacteria_Hoeflea_phototrophica-choline_kinase_
--K--IVIAPFVYVSGGISNNWWFVKVPGGTEMFIDRAIKAPVYLGVEIFLGPSFTAIYILHLLVFIHVQVRGINTAAMDLVTFNDPMGNFMILIDYEYMNYDFGIWFGE------MFFPELIEYF-VRSIV
IVHKALADVKWCLWSMVQLSLRFDFHKYGMWKKAH-
>565847640_e-proteobacteria_Helicobacter_canis-hypothetical_protein_
LTHYFAVLLEYTIFSDGITNNY-VIRIPGHTEQVIDREAIIPSRGGIKMYLDPLIIIALALHIHLLIYELA-IERLIKLELVLHRDLQPNILILIDFEYFAWELGNLAAELE----L--KILLHY--PRLTV
LCGALLANYIWALWGWV--H-RIDLGRMYLARFN-E
```

>739707252_spirochetes_Spirochaeta_sp_-hypothetical_protein_
-----IVVIDWIIMTGGMTNNFFFVRVPGSTELLIDREHAAPVILHVMVFIGTMLMIALKLHGKMFVYLIVEQYRLLIEQGVPNNDLLENYILLIDYEYNNFELGNTCQEQQ----Y--EYLCAYF--QRLM
YLFSIMSDFGWTLWGSIQNSLDFDFWEYTMERWALE
>518986967_high_GC_Gram_Terracoccus_sp_-hypothetical_protein_
-MR--LALLA-SVLPGGLTNNYVVVRVSSTSALLVDHEWAAAVVVGILVFLGTYVNIALRLHGPMFQYAIVRGFAAVALPLVPHNDLLANFLVIIDYEYTNFELGNLVNESR----L--HLLVDYH-VSDVA
ELWGLAGQYAWTLWGAIQHSLDADFWGFATERFASL
>497463945_high_GC_Gram_Janibacter_sp_-hypothetical_protein_
-----AQL--WRVLPGGLTNNYVVVRISSDSGLLVDREWTAAVVLGVLVFLGTYVNIVVKIHSPMFQYAIVSGHRMVALPLVPHNDLLANVLLIIDYEYMNFELGNLINESQ----L--HLLVGYY-VSHLA
ELWGLTGRYAWTLWGAIQYSVDHDFWDFTLER-LTR
>665500652_high_GC_Gram_Tetrasphaera_jenkinsii-conserved_hypothetical_protein_
-----LLLLV-IALPGGLTNNVVVVRISPAAGLLIDREFHAPVILGVLAFVSTLV-IALTLHTSMFQYLLLTGLAWLALPRVPHNDLLANFLLIIDFEYNNFELGNLAQENH----L--DLLATYY-DDELC
RLWQIASAYAWTLWGLISANLDFDFEGWGLEKFAMG
>736805739_high_GC_Gram_Intrasporangium_oryzae-hypothetical_protein_
-----ASL--WV-LPGGLTNNFVVVRLSSDSELLVDREWAAAVLLGVLVFLGTYVNIALRLHG-MFIYRIVA-LRVVALALVPHNDLLANFLVIIDYEYMNFELGNLVNESR----L--HLLVEYY-VTARA
ELWGLAGQYAWTLWGAIQHSVGADFWSFALERFASV
>738270011_high_GC_Gram_Marmoricola_sp_-choline_kinase_
----MAAVLG--VLSGGLTNNVVVVRIAQGSELLIDRERAAAVIVRLLVFIGTFLHVACQLHG-MFQYLIAHGFQAIALATVPNNDLLGNFIILIDYEYNNFELGNLWSEAN----L--DLLMEY---LRRA
RLWGLMSKYGWTLWGSIQSSIDFDFWEWAMEKYAFG
>916541658_high_GC_Gram_Frankia_sp_-choline_kinase_
-----TSA-S--ILPGGLTNNYYVARTSSHSGLLIDREYAAPVVRGVMVFVGTWVNIACQLHGRMFQYLIVQGFTLIALLTVPHNDLLENFILLIDYEYNNFELGNIASESN----L--DLLVSYF--SPKA
QLWALMSQYGWTLWASIQDSLDFDFWNWGMEKYAFD
>829459231_high_GC_Gram_Mycobacterium_sp_-choline_kinase_
----MLLA-P--LLSGGLTNNIYVARCSIATSLLIDREYAAAVIRGILLFLGTLLVVACTLHG-MFQYLVVQGFEFVILVTVPNNDLLGNFVVLIDYEYNNFELGNIWAECG----L--TLLVSYY--LRKA
RLQGIIGKYGWTLWGCIQNSLDFDFWEWAMERYARD
>654609830_actinobacteria_Solirubrobacterales_bacterium-hypothetical_protein_
-----VAVQG-GPLGGGITNNFVVLRIAGDTELLIDREHAAPVLLSCLVFVAPILAVALAFHRPVPAYLLARAALAIAAP-VSHDDLLANILTLVDWEYMGFDLGNLSINNG----F--DDLLEYW--TPRL

RLMRSMSDVREALWGVAQASLDFDFSAYADKHLLSR
>652639947_actinobacteria_Conexibacter_woesei-hypothetical_protein_
-----VAVRG-GPLGGGITNNFAVLRVAGDTELLIDREHAAPVLLSCLVFVAPILAVALAFHHPVPAYLLAG-LRIAGS-LVPHDDLLANVLVLVDWEYMGFDLGNLSINNG----F--EDLLSYW-CTPRL
RLMRVMSDVREALWGVAQTS-DFDFPGYAERHFLTR
>505063039_planctomycetes_Singulisphaera_acidiphila-choline_kinase_
-MS--NRIITWVILPGGITNNYYVARLCVKTLLGIDREVAAPLVEGVLVHVGTLVFLVLTLHSAPFAYATAANLDALQLVPVLHNDLLGNIIVLVDWEYIGFDLAGVCANNA----F--EELLGY---NEDL
RILKTMSSLREALWAVIQTSIDFDYVRYANENFYRR
>916635378_a-proteobacteria_Stappia_stellulata-choline_kinase_
---MTTRVLPWPMLSGGLSNSVHVVRL-GDFPFHVERETAAPLVEGVMVFIGTYVDIAIRFHE-FWVYATLKHIAM--HPIVFHHDLLANFILLIDFEYFGFDLAGVTSNAG----L--ASLMNYL-PDAMY
AAMQVASLLRETMWSLVELT-GVDYAAYTAETLYLR
>640280018_a-proteobacteria_Aminobacter_sp_-choline_kinase_
-----EIK-PWIILLGGISNSWHVVRL-GDYPFHVLRELAAPVEEGIMVYLATYVNIAVAFHEPFWVYATLRGLYLALAPIVFHNDLLANFLLLIDFEYFSFDLAGLSSNAG----F--ASLLEYF-PDELL
AAMQCASLLREAMWSMVELN-GTDYVAYTEMNLLLR
>874081251_a-proteobacteria_Nitratireductor_sp_-choline_kinase_
-----SIA-PWIILIGGISNSWHVVRF-GDYPFHVLREVAAPVEEGVMVHLATYVNVTVTFHRPFWVYATLSGLELA--PIVFHNDLLANLLLLIDFEYFSFDLAGLSSNAS----F--DSLLGYF-PDRLL
AAMQCASLLREAMWSMVEID-GTDYIAYTQQNLLLR
>590076013_a-proteobacteria_Aquamicrobium_defluvii-choline_kinase_
---MVQ--GPWIILMGGISNSWHVVRF-GDYPFHVLRELAAPVEGGVMIHLATFVNVAVGFHKPFWVYATLIGMEL--APIVFHNDLLANLLLLIDFEYFSFDLAGLSSNSG----F--RSLLGYF-PDELL
AAMQCASLLREAMWSMVELD-GTDYVAYTAENLLLQ
>917008000_a-proteobacteria_Phyllobacterium_sp_-choline_kinase_
---MAEVAIAWIILKGGISNSYYVVRF-GDYPFHVFREVAAPVFDGVMVFLGTYVNIAVQFHRPFWVYATLEKIEMA--PIIFHNDLLANILLLIDFEYFSFDLAGLASNAG----F--ASLLDYF-PDTLH
AAMQCASLLREAMWSMVELK-GVDYVAYTTENLLLL
>918755647_high_GC_Gram_Actinobacteria_bacterium-hypothetical_protein_
--V--INLLPWILLEGGITNNYFVLRIPGRTDLLIDREARAPVVLGTQIFVGHLFRVVISLHSDIHLHADAEGLESIVFPLVLHNDLLGNLLVLLDYEYMNFDLANLSINFG----L--AELLQYF-VASEL
SLMKVMSEFREGMWGVVQQSLETDFVEYTRNRLADE

Supplementary Data S7. The edited multiple sequence alignment for the phylogenetic tree reconstruction of Pis

>71743968_kinetoplastids_Trypanosoma_brucei_phosphatidyltransferase_

LYPNLITRVILSISFCLLAFLFCYIIVDAVDGVARTFGAILDLTDRAAGLIVVVGAACLVFLDIPFMVLFYLNLFFMALTAALSAFKQVNV

>118389454_ciliates_Tetrahymena_thermophila_CDP_diacylglycerol_inositol_3_phosphatidyltransferase_

LIPNLIGRVGCIYVCFYYMTAFMYALADAFDGAARNFGAVLDVCDRVAIMLSILVFLLACGLDIGFLLMFLVNMSLLLFSVPVFILKSFSV

>120593503_b_proteobacteria_Polaromonas_naphthalenivorans_CDP_diacylglycerol__glycerol_3_phosphate_3_phosphatidyltransferase_

LIPTLMTRIVAILIVGFYIATVMFVVADWLDGLARSFGAFLDVADKFCASLLVLVALIIIGREIVKTIFL-LYDL-IWLSAILTVWSMVYL

>124001348_trichomonads_Trichomonas_vaginalis_CDP_alcohol_phosphatidyltransferase_family_protein_

FIPNLIARIILMVAYLFAIFFLSYFSFDMFDGAARTFGALLDVTDRCVGLLLVLTLHFFIWLDIWFMFGWPCTMLLKYVLLPLFMLKHINV

>156102783_apicomplexans_Plasmodium_vivax_phosphatidylinositol_synthase_

LIPNIIIRVILAWGFVVCLFAVLYGTIDAFDGTARSFGQILDITDRLTLLYLLNAIGLIMIADIVVMISFWFAAYALLCSSPLAAFKMFNI

>157435272_diplomonads_Giardia_lamblia_CDP_diacylglycerol_inositol_3_phosphatidyltransferase_

FLPNIVSRIVVLVAVAVFMAVFCFVYMDCFDGLARSLGATLDVIDRCALLCMILGFMFLTFLDIPFLVAMLISLLIACFSSPLTILKIYNG

>183230968_eukaryotes_Entamoeba_histolytica_CDP_diacylglycerol__inositol3_phosphatidyltransferase_

LYANICVRIGVLIAVFFVTFIICYIILDAVDGLARSLGAVLDITDRATILITALICCLITFIDIVL-LATFVMWTLLIISTPLCILKNLNI

>255074271_green_algae_Micromonas_sp__predicted_protein_

AVPNIIVRVLLAAALRLATSLALYALVDELDGFARSFGKLLDVTDRLTGLLMVLFFVSLVALDVIFMVVLYLAAQLALVALPFWAVKQANV

>281204118_cellular_slime_molds_Polysphondylium_pallidum_hypothetical_protein_PPL_08965_

AIPEIVARVIFVLAFFYSSFFVYYALLDMADGAARSFGALLDVTDRCAALIVVLIF--LIVLDIYFLFGFFLFSIQWYIWFPICFVKQSNV

>290979137_eukaryotes_Naegleria_gruberi_predicted_protein_

FIPNLILRVFLSLSFFIYTVIVIYTTVDGLDGAARSFGAVLDVTDRFAALVTILYFVLLNILDFTFMVLFYVFLLIIALALLVWAGKQLNF

>294874500_eukaryotes_Perkinsus_marinus_phosphatidylinositol_synthase__putative_

LVPNLITRVALMISFYVAAFVFCYFWMDAFDGAARSFGAVLDVTDRINILALILPLVMFAVVDYVMLFAVLVLMYAAVAFFPIALLKQVNV

>299115878_brown_algae_Ectocarpus_siliculosus_CDP_diacylglycerol___inositol_3_phosphatidyltransferase_

FFPNLIARIIAVASYYLAASFVCYVLVDAVDGVARSFGAVLDVTDRCAGLLMVLLFLLLMFIDLPFFVLFYVALVCYYVFLPACVCKQANV

>401396403_apicomplexans_Neospora_caninum_hypothetical_protein_NCLIV_002660_

LVPNIIVRIALLAAAIWQLFFFCYLTCDAVDGAARSVGACLDVVDRLCLLYILNAFFLALFLDVPVMLGFFMCLVIVYATSPLMLFKTLNL
>403345639_ciliates_Oxytricha_trifallax_hypothetical_protein_OXYTRI_06808_
MVPNLIARFLFLGSYFFSLYPIFYGILDMADGAARSYGAALDICDRAGTMFLLLFFYMCFILDFIFFITCIIVLILAGVLTAIMSTKMLNV
>413955065_monocots_Zea_mays_hypothetical_protein_ZEAMMB73_557571__partial_
CLPATPFRIIINIAFAVCLFAILYFIVDGVDGFARSFGAVLDVTDRVACLLALLVFLILLGLDI---VILF-----SFCKFPVCFLVST--
>488763027_planctomycetes_Gimesia_maris_CDP_diacylglycerol__glycerol_3_phosphate_3_phosphatidyltransferase_
WLPNLISRLVLAVLFVIYTSAAIFILADFLDGFARTLGRILDFVDKICGAFIFLFVLIIIGREMIKMVLCLLSLISIWSAALITLYSGVYV
>493938115_euryarchaeotes_Halosimplex_carlsbadense_hypothetical_protein_
WVPNLISRLPLVGI--VLVRYALFALLDGVDGVARTLGALLDALDKLLVLVVALAYALFFARDAVAMGALLWALVHDWRGIAVAAAAVAAF
>495187196_GNS_bacteria_Ktedonobacter_racemifer_CDP_diacylglycerol__glycerol_3_phosphate_3_phosphatidyltransferase_
RVPNILSRLISTIVFIVLVATVLFALVDYFDGLARSLGVFLDTADKVVSILIAMIVFIIVTREFIAIKAHLLSLILLIVATIWTIFSGIYT
>499776298_a_proteobacteria_Jannaschia_sp__CDP_diacylglycerol__glycerol_3_phosphate_3_phosphatidyltransferase_
WVPNILVRLIAAMVFVLLVALFLFMAMDYLDGLARSFGAMLDIADKAIALAVLLILIVILFREIMVAWTHYFWAVLLWVAALLTAITGFYY
>502756950_aquificales_Thermocrinis_albus_CDP_alcohol_phosphatidyltransferase_
F-PNLILSLITGLSAYFWTAASFLILLDLADGVARSFGAVLDMVDKWGVVLGTVLTAVVVVTASPETLLERLSFVGIKIIGLLTLLSLLRL
>503404040_bacteria_Desulfurobacterium_thermolithotrophum_CDP_alcohol_phosphatidyltransferase_
VIPNVIIRGFLVLFIMVFLALIIFLVIDALDGLARTLGVILDIADKANSGFILLLTILVLSRDVLTATTLLNLNVIYGVTALLTVLSAIYG
>505045546_crenarchaeotes_Caldisphaera_lagunensis_phosphatidylglycerophosphate_synthase_
L-PNAAIGFLFALAPTAYAIPIMIVLLDVVDGIARTFGAYLDLTDRIMLFFLALLSIIALGFSELILVLALLKYIIIVIIAILGAITVYRS
>510903358_apicomplexans_Babesia_equi_CDP_diacylglycerol_inositol_3_phosphatidyltransferase__putative_
LKPNLVTRFLLLTSFYFARFLFLYVVTDIFDGIARTVGGLFDSLDRVLYIYLLVHMYLITVLDVNFMITFWLSLVLLYISFPLMFYKTLNL
>517492295_firmicutes_Thermobrachium_celere_CDP_diacylglycerol__glycerol_3_phosphate_3_phosphatidyltransferase_
IIANKVLALVVGLPLFLIIPILFLWLFDAVDGIARTFGTVMDVFDRIVALIISLHLSFVILSCSLMEFTLIIIFLIAFVFAGTIIFTAIRF
>518338952_cyanobacteria_Pleurocapsa_sp__hypothetical_protein_
LVPNIIVRFIFYISFIHSLCISFYAIIDEFDGAARSFGAALDVADRSAGLCLILFFIVAIALDISFMLLFYMLLLLIIICLPIYLLKQANI
>524118579_firmicutes_Firmicutes_bacterium_putative_uncharacterized_protein_

MIPNLLIRLLSPFIIPFLLAFILVIVIDIFDGIARSFGRLLDVTDKVLSLLIPLLYIVLIILEIIKTILCYLSFVVFVINIILELITLSYV
>524248679_firmicutes_Anaerotruncus_sp__cDP_alcohol_phosphatidyltransferase_
FIPNWMVRIALIVFAVFIWAVITMIVLDVFDGIARSLGKILDIADKLMAIVVILIFFLFIIKELTIIGSPLVGVVMVIISALLAFVSLFYA
>544213758_red_algae_Cyanidioschyzon_merolae_CDP_diacylglycerol__Inositol_3_phosphatidyltransferase_
LVPNLIARIGLAVAFWVPTFLTFYTTLDAADGAARSFGAVLDLTDRFAGLLLLIVCVALLILDASVLLVFLLLHAAYYGCAIVYVLKQFSL
>545353830_green_algae_Coccomyxa_subellipsoidea_CDP_diacylglycerol__inositol_3_phosphatidyltransferase_
LVPNLIARILSACAFAIALCVVFYFFVDELDGFARTLGQVLDALHRLAGLLAILYFLALLMLDILFMVILYLSLVVAVLAVPGFVIKQFNC
>551065165_firmicutes_Bavariicoccus_seileri_phosphatidylglycerophosphate_synthase_
IIPNILFRLLLIVIIYYVVAGVMLIIVDLVDGVARSVGKVLDVADKLLAILFCLLFGLLIVKEALSTLMIIHVLALVIATIVVTTFSLIYL
>558596188_diplomonads_Spironucleus_salmonicida_CDP_diacylglycerol_inositol_3_phosphatidyltransferase_
LVPNLIIRLLLLIVLY--KQAVLLILVDLLDGIARTLGQILDVVDRITIISILIPFTLLIVIDIILLFGFLISFLFCSISFIPLILKVWSI
>565490487_eudicots_Capsella_rubella_hypothetical_protein_CARUB_v10021013mg_
LIPNIVATFLIVFPLNLSLNSLLFFFCDAVDGVARSFGAVLDVTD-------------------IFMVVLYIILLSFLLALTLWSMKQTNV
>654595314_actinobacteria_Solirubrobacter_soli_CDP_diacylglycerol__glycerol_3_phosphate_3_phosphatidyltransferase_
L-PNVLLRILLVVLVALVLAAAIFVVADALDGIARSFGKLMDLADKLAALVSLVVAMVIIAREFLKTVILVEDSALVWATVIITVISGAYF
>655079852_firmicutes_Ruminococcus_gauvreauii_hypothetical_protein_
LIPNLLFRILLLVYLYYVLAVFIIFLLDFLDGIARTFGKILDIADKIGALALSFLLAVFLIKEAICTMLLFHDILLILACILAMIISFIYL
>655940994_g_proteobacteria_Thermithiobacillus_tepidarius_CDP_diacylglycerol__glycerol_3_phosphate_3_phosphatidyltransferase_
MLPNIIFRILLVAMIYLLWAVAIFLVIDALDGIARTLGAILDLADKLAAVVILLLTAAILLRDLFILLARWLALAGFVLVFASTVLSGLYV
>697891333_apicomplexans_Theileria_orientalis_CDP_diacylglycerol_inositol_3_phosphatidyltransferase_
IKPNSIFRVILLTSFLFYHFLTLYVAVDMCDGIARTFGAMFDLFDRMTFLYMMLFFYFILFWDVPLMVAFFLSLFLVFGSMPLAFFKAFNT
>736186722_firmicutes_Alkalibacterium_sp__hypothetical_protein_
FIPNILVRLLLIVFVHYLQAGGILFFLDALDGIARSLGQLIDIADKLLAVVGVLLFLFLLFLFKIATFMLLAVLALIWLIAGLASFFQLAF
>736816486_firmicutes_Exiguobacterium_alkaliphilum_CDP_alcohol_phosphatidyltransferase_
KTPNEVLGIVGVVGFFIYTAVILLWLLDVLDGMARTFGTVLDVVDRLIAVVIALMTVFLLLLASGILFSLMILFVIATIFLILEVVTISRM
>749162981_apicomplexans_Gregarina_niphandrodes_putative_CDP_diacylglycerol_inositol_3_phosphatidyltransferase_

LWPNIIVRILFLAAVSTFTAAGYYTIIDALDGVARSLGACLDVTDRLLAIYMLLLIFALAAADILFMLVLCCSFWLGVISAPLGVVKAVTQ
>753852537_firmicutes_Halobacteroides_halobius_CDP_diacylglycerol__glycerol_3_phosphate_3_phosphatidyltransferase_
MVANCLIRILLLFFLFFFIAAIIFTLLDLFDGIARTLGKILDLADKLIIVFTALLIVFILVRELGATYVSYIWDPAAFIAIGLALLSGVYC
>753990668_e_proteobacteria_Sulfurovum_sp__CDP_diacylglycerol__glycerol_3_phosphate_3_phosphatidyltransferase_
FIPNILIRLLLALMFLLVFAAFIFVVADFFDGIARTLGAILDLADKMLAGFLGLAIFLILTRELVKTI-FLLMQVLLWIAVVLTLYSGYYV
>771605825_d_proteobacteria_Desulfonatronovibrio_magnus_CDP_alcohol_phosphatidyltransferase_
WIPNVLLRIFITAFVVFFLALVLFITIDGLDGLARSLGAMIDLADKFLTAFVCLLIVVVFTRDLLNTLAMVAHTFLVYIVGFLTIFSGIYV
>828175862_bacteria_Marinitoga_sp__CDP_diacylglycerol__glycerol_3_phosphate_3_phosphatidyltransferase_
WIPNILIRLATILFIIYNIAFFIYILLDLLDGIARSFGKFMDIADKINALFIVFFVAIIITRDIFKTLLYLTAIILIILTVFFSLFSGYYL
>836601192_bacteria_Kosmotoga_pacifica_CDP_diacylglycerol__glycerol_3_phosphate_3_phosphatidyltransferase_
MIPNILSRILTIIVAFYFVSFAFFIILDYFDGLARSFGKFLDISDKISLLLIFMLVFVVLFRDTAKTFLYFQQITFQWLVALITVLSGVYI
>857975063_cercozoans_Plasmodiophora_brassicae_hypothetical_protein_PBRA_007070_
LWPNLIARIVSLVALFYAQAGLCYAVLDAVDGVARSFGALLDLTDRMTCLLVVITF--IIVLDLFLLVAFIIGTAIKYVCLPVFLGKNFNV
>873225939_eukaryotes_Vitrella_brassicaformis_unnamed_protein_product_
LVPNIIVRVVLAVAFALAGFVVCYSTVDAFDGAARTFGAVLDVTDRFAVVLMIDLFLCLVILDIGVLAMCWLTLGLAVICAPLSLCKQVNL
>873235542_eukaryotes_Vitrella_brassicaformis_unnamed_protein_product_
FVPNLIVRLVLLAMFGFMAFSLLYALTDFFDGTARSFGAIIDVIDNIQLLWSRV---SVAPLIAFRTVLMFLPLLARWLAHPAAVLAAGLL
>910760488_kinetoplastids_Perkinsela_sp__phosphatidylinositol_synthase_
FWPNIIIRVFLLFAFSKAAFAISYILADGLDGLARSFGAVLDMCDRTCGLIIVLTGIFLIALDFWIMALWYLSMTIMANIFGLFLIKQWNL
>916698507_d_proteobacteria_Desulfobulbus_mediterraneus_hypothetical_protein_
RIPNTLLRLLLAIFFS--LWLWLIIGCDVLDGIARSLGAILDVADKLLAALLTLIP-LLLARDLLATLTAVIFPPILWLATTLSVLAASYG
>917308060_GNS_bacteria_Thermorudis_peleae_CDP_alcohol_phosphatidyltransferase_
-MANLIARVALLVGIALYVVLFAVGLVDAVDGVARTFGAILDAGDRVNALWIVFAPLLVVTRGFALYLAFLSGLVTVYVTLALVVVRGLVL
>922862606_haptophytes_Chrysochromulina_sp__phosphatidylinositol_isoform_a_
LVPNLILRLALALGYAYAVLLGAYSLLDAADGAARSFGAVLDTTDRATCLCIVLLFAALVMVDMVL-ALWYLSLLTLMFCTPFFVFKQVNL
>933880933_a_proteobacteria_Prosthecomicrobium_hirschii_CDP_alcohol_phosphatidyltransferase_

L-PNLIARLIAVMVVVISLAFWLFVVADGVDGIARTLGAYLDIADKSVAIYVTLLVILVVSRDLILLLAFAADLVGLWIVAGLTVGSGAYL
>939328615_firmicutes_Oxobacter_pfennigii_CDP_alcohol_phosphatidyltransferase_
L-PNAIMRIVCSILLFKPLFFILYILIDFLDGIARSFGAVIDTADLIVLLMIFIVLGAITIIRIKASFYNFLGLILCSMASI-SALEELTS
>939329865_firmicutes_Oxobacter_pfennigii_CDP_diacylglycerol__glycerol_3_phosphate_3_phosphatidyltransferase_
MIPNLLLRFILVVFLFFFISSMIFIFFDILDGIARTWGTLLDLADKLLTVLISLVPFIIGIKEGMATITLEFINIFMYITVFAALFALYYL
>941500400_bacteria_Candidatus_Cloacimonas_hypothetical_protein_APR54_11285__partial_
L-PNLLTRFLIVIFYFIFYASLLFIVLDYFDGLARSFGKIMDLADKVTALIALSLVYLILIREIIKTAVALIYEIIDNLFLIFFLIVAVTW
>949039628_actinobacteria_Acidimicrobium_sp__hypothetical_protein_ABR67_00975_
SVPNAIVRLLTLWFYMFSGAALLLGVMDWVDGIARSFGSVFDAVDRVVGAVSVMLCIAILTREIRYTTLLLLAGMLAIPGLFLSYTTAFYI
>949080227_CFB_group_bacteria_Cryomorphaceae_bacterium_hypothetical_protein_ABR83_00670_
RVPNALYRIIMFVLLYVYVFSWLICILDIADGIARTLGAQLDIADYGAAAFYGLFVLVWVFVAFKVGLLFSFGFGVVSFTEGMLILALLEM
>950900471_eukaryotes_Blastocystis_sp__hypothetical_protein_JH06_0781_
LIPNIIIRAVTGLSFYFVAFCWLYFIIDALDGCARSFGAVLDICDRFASLYLSLYYTLLLILEIYSLLTYIVSSLINKYFFPVFVFKQISL
>50556792_YALI0F23837p_Opisthokonta_Yarrowia_lipolytica_CLIB122_YALI0F23837p
YLPNILTRLLSALIGYVILATGLFVYVDMLDGIARSVGSVIDLADKFITLTVCLLTTLILGRDFYNTIMSKPIIFLSGMSYVFTAVKILYP
>72044581_PREDICTED__uncharacterized_protein_LOC583015_Opisthokonta_Strongylocentrotus_purpuratus_PREDICTED__uncharacterized_protein_LOC583015
LVPNIIVRLALVSSCFFYVFISLFLAIDGFDGLARSFGAWFDVIDNLGMLWCRLGY-FIAAIE-QGTFLWLYAHVQYAGIAFLSAGRALFV
>358342389_CDP_diacylglycerol__inositol_3_phosphatidyltransferase_Opisthokonta_Clonorchis_sinensis_CDP_diacylglycerol__inositol_3_phosphatidyltransferase
LVPSVSGRIILLYSCWHMLTVFAYVVLDAVDGAARSFGAMLDLVDRCMCLLACLLFQISMLVDISPE-------CIVWLHITI------ST
>401825193_CDP_diacylglycerol_inositol_3_phosphatidyltransferase_like_protein_Opisthokonta_Encephalitozoon_hellem_ATCC_50504_CDP_diacylglycerol_inositol_3_phosphatidyltransferase_like_protein
FVPNTIFRIGLLASVF--SFVALYGLLDFFDGFARSLGSSLDITDRVVVISLRIFLSLYMVFDLVL-LVFF--MALLYTLGGITTLKALHL
>484854657_CDP_diacylglycerol__inositol_3_phosphatidyltransferase_Opisthokonta_Nosema_bombycis_CQ1_CDP_diacylglycerol__inositol_3_phosphatidyltransferase

FVPNIILRIILLFAIT--IFSVSYIISDFFDGAARSLGGCLDIIDRIVIIGLRMFVSFYILIDLFVLLAFFISARIVNLLQIVCWLKTFHI
>514686679_CDP_diacylglycerol_inositol_3_phosphatidyltransferase_Opisthokonta_Salpingoeca_rosetta_CDP_diacylglycerol_inositol_3_phosphatidyltransferase
LIPNLIARVALLASLFFMVAMSLYWLFDAFDGAARTFGSVLDVTDRVLCLMMTCAFQLLAVLDIVL-SLFFMALWIGWITFPLFALKHLSL
>528890808_CDP_alcohol_phosphatidyltransferase_domain_containing_protein_Opisthokonta_Rozella_allomycis_CSF55_CDP_alcohol_phosphatidyltransferase_domain_containing_protein
Y-----AI-------------VVYLLIDAFDGAARSLGAVLDVTDRSAGLICYLIFQFLLGLDLTALALFYVFLNALYVVFPVWLLKQVNV
>530540390_cdp_diacylglycerol_inositol_3_phosphatidyltransferase_pis_Opisthokonta_Nosema_apis_BRL_01_cdp_diacylglycerol_inositol_3_phosphatidyltransferase_pis
LIPNLITRLILLLSIY--YFILFYTTLDILDGVARTLGSCLDITDRLPIICYRIFISLYTLIDLTVLFLFFICLMFYKFFYGFVIVKMFNF
>597875750_hypothetical_protein_Y032_0012g1711_Opisthokonta_Ancylostoma_ceylanicum_hypothetical_protein_Y032_0012g1711
LYPNLIGRIVLAISFYTMIALLCYALIDAFDGAARSFGAMLDLTDRCMALCMALMFQMSTVVDITFLAAFYQILYFAALAFPIALVKSLSL
>598028451_hypothetical_protein_AURDEDRAFT_183519_Opisthokonta_Auricularia_subglabra_TFB_10046_SS5_hypothetical_protein_AURDEDRAFT_183519
YLPNLLSRIVACVLGWVLLATGILVYADWIDGIARSLGTILDAADKATTLTVTLLAVIIIGRDVVNTMTVNPVLYLQWTVGATTIWSGLYV
>667641650_hypothetical_protein_VICG_01696_Opisthokonta_Vittaforma_corneae_ATCC_50505_hypothetical_protein_VICG_01696
FIPNMIFRIILLVSVF--SFALLYITSDYFDGAARSLGGALDIIDRVMVILSKICI-LYSIIDFGFLLLCFILTSILSFLQLVAATKTFHI
>682265091_hypothetical_protein_V490_06054_Opisthokonta_Pseudogymnoascus_pannorum_VKM_F_3557_hypothetical_protein_V490_06054
LWPNIIMRIVLAGSLYYMTCTLLYSILDALDGAARTFGAVLDVTDRCACLLVFLSIQGLISLDLTVLAIFFIALVIARVSFPVMAGKQINV
>749714097_CDP_diacylglycerol__inositol_3_phosphatidyltransferase_Opisthokonta_Thelohanellus_kitauei_CDP_diacylglycerol__inositol_3_phosphatidyltransferase
LIPNIVVRLALLASCYYMAALILYGLIDALD----SVGVVLDLIDRVLLI----AFQVLAALDIVVLVMFLLLIPMVIFCLPIFAFKQLNV
>871250927_PREDICTED__CDP_diacylglycerol__inositol_3_phosphatidyltransferase_like_Opisthokonta_Aplysia_californica_PREDICTED__CDP_diacylglycerol__inositol_3_phosphatidyltransferase_like
FVPNIIFRVVFAVSFYYMRASLFYLLFDAFDGFARSLGAMLDLIDRIMCLCATLFFQFAMALDIVVLALFFCMLLVAVLTGPLACLKGVSV
>884947681_PREDICTED__uncharacterized_protein_LOC105012953_isoform_X1_Opisthokonta_Esox_lucius_PREDICTED__uncharacterized_protein_LO

C105012953_isoform_X1
VWPNILIRLVLITA-WNAVFFTSYSIIDGLDGTARSFGAWLDVVDNLSMVWGQLRFGTYNKQTVLDVGMRYVGLFTRPTTLAVWVPAGGHP
>906371880_hypothetical_protein_NEPG_00667_Opisthokonta_Nematocida_parisii_ERTm1_hypothetical_protein_NEPG_00667
CIPNIISRVVLLIALI--VFVILYSVLDALDGAARSLGYILDATDRAAILIIKTPLCGFLIVDIPVLLMFLLNSGIYLICGSIFAFKHLNM
>908414209_PREDICTED__probable_CDP_diacylglycerol__inositol_3_phosphatidyltransferase_2_Opisthokonta_Biomphalaria_glabrata_PREDICTED__probable_CDP_diacylglycerol__inositol_3_phosphatidyltransferase_2
FIPNVIVRILLAGAF-LFWFTILYTASDGIDGLARSFGAWFDVIDLFGYLWCAL---LIACLE-THLGLLPLWLLILVVLLTGRLLCSMEF
>919064282_PREDICTED__CDP_diacylglycerol__inositol_3_phosphatidyltransferase_like_Opisthokonta_Lingula_anatina_PREDICTED__CDP_diacylglycerol__inositol_3_phosphatidyltransferase_like
FVPNIILRIGIAVAFCLMAAFWLYIVSDMVDGFARSFGSLLDLIDRCLCMLTRLVFSALTCLDILFLYTFFGLLLICVACAPVEIVQIVNL
>919100578_PREDICTED__CDP_diacylglycerol__inositol_3_phosphatidyltransferase_like_Opisthokonta_Lingula_anatina_PREDICTED__CDP_diacylglycerol__inositol_3_phosphatidyltransferase_like
IVPHIILRILMGGLFHCLKAFWLYTFADMVDGIAKSFGTLLDMSDRGLCMLARLLFIGIACTDVVIFYYFTTFTVICIGCSPFAALQVGTL
>926635337_PREDICTED__CDP_diacylglycerol__inositol_3_phosphatidyltransferase_1_like_Opisthokonta_Limulus_polyphemus_PREDICTED__CDP_diacylglycerol__inositol_3_phosphatidyltransferase_1_like
VIPNIIIRIILLIG-WIIWFLVVYVTMDGIDGAARSFGAWLDIIDNIGMLWCLIVV--------TPPAFLPVWLQYIVLGFLLRALAMSEV
>918755913_high_GC_Gram_Actinobacteria_bacterium_hypothetical_protein_
WIPNLILRLLCVIFVWLLSAAILLGVGDWVDGIARSIGQVLDVADRIITAAVTLIAALVLFREAAGTFGFLLLTSATIGGLLLSYWAAAYV
>880971721_high_GC_Gram_Tetrasphaera_australiensis_CDP_diacylglycerol__glycerol_3_phosphate_3_phosphatidyltransferase_
WIPNLLLRLVGVIFLWILLALVVLMLIDYLDGIARSLGALLDIADRLLSTLLGLLVGVLIAREVAATYCLLLADGFAWWGIVLYWVAGLYA
>915462665_high_GC_Gram_Pseudonocardia_sp__CDP_diacylglycerol__glycerol_3_phosphate_3_phosphatidyltransferase_
WVPNALLRLLGVLFLWMLWAVTVLAIFDWLDGLARSLGAMLDAVDRLLAALVGLVVAALVARDVAATYLLLLGDAFAIWGVALYLWTGVYL
>658453440_high_GC_Gram_Bifidobacterium_sp__CDP_diacylglycerol__glycerol_3_phosphate_3_phosphatidyltransferase_
LVPNVILRILSILIAYVAISLVVMLILDGVDGIARSLGQILDVADRLLCSVLALLLALVGARDVTGTALIFADVAAVIWGVALYWLAGLYA
>939720301_high_GC_Gram_Flaviflexus_sp__hypothetical_protein_
LIPNIIVRLLLIICYLAIVTAILLVTADWIDGVARSVGEILDIADRLAVIALFLIAYVVIGTDLVRTALTVFGRVAAAVGAILHAITGIYA

```
>498209542_high_GC_Gram_Nesterenkonia_sp__phosphatidylglycerophosphate_synthase_
WVPNIVLRFLLVVFIQALAAFVVLVVGDWIDGIARSVGRWLDLADRLAIVAVTLFVLSILVPDAVRTVLVLLARSVLAAGCLMHILAAVYL
>655295142_high_GC_Gram_Propionibacteriaceae_bacterium_CDP_diacylglycerol__glycerol_3_phosphate_3_phosphatidyltransferase_
WVPNVLIRIILVLMVLFLWPTVVFAVLDFLDGIARTFGKLWDIADKAGAAFISLFTVIILVREWLKTQLLLFLLVLMWAALILTVVTGIYL
```

Supplementary Data S8. The edited multiple sequence alignment for the phylogenetic tree reconstruction of Pgps

>154420388_trichomonads_Trichomonas_vaginalis_CDP-diacylglycerol_glycerol-3-phosphate_3-phosphatidyltransferase
VNIANAITLRIPMLFMIVAFLKIATIIFFFGWTDLDGLARKICSLGAFVDAVTDKIFMLFIMLLVTLFFILREFLITALRAVAASIVIAAGKIKTVQMISITLLILALFIFMTAFSGVYIVL
>159113558_diplomonads_Giardia_lamblia-CDP_diacylglycerol_glycerol_3_phosphate_3_phosphatidyltransferase_
VNLPNILSLRIPLLFSITACLFVSFTLCVVAITDLDGTARKATSFGKLFDALCDKILTIFIFITYFVFPVLREFLVTGLRMIAAGVVMAAGKVKTAQMFICAILFIVMFFVLALMSGYYLLI
>168991685_firmicutes_Lysinibacillus_sphaericus-CDP_diacylglycerol__glycerol_3_phosphate_3_phosphatidyltransferase_
MNIPNKITIRILLIPFFVIVMMIGALIFILATTDVDGYARKLVTFGKFLDPLADKLLVAFIMVLAVIIIIIREFAVTGLRLILAGEVVAAGKIKTWQIVIAAALLFIMLVFFTLWSGWYFVL
>251770945_bacteria_Leptospirillum_ferrodiazotrophum-CDP_diacylglycerol__glycerol_3_phosphate_3_phosphatidyltransferase_
LNLANVITLRIFLIPAVIVFDKIAATLFVIALTDVDGVARRQVTMGKLLDPIADKILVVLILVHLLAIVIVREFAVSGLRQVAAGIVIPAGKVKMAQTIIALLLDFLAFVILAIVSGIYLIW
>291542168_firmicutes_Ruminococcus_bromii-CDP_diacylglycerol__glycerol_3_phosphate_3_phosphatidyltransferase_
MNLPNKLTVRIILVPFFVAALFVALIIFVMALTDFDGIARKLVTFGKFADPLADKILVAFLFVLCAVIIVLREFSVTSIRLIAAGKVVAAGKAKTVQMIIIAVIAIILIIVFTIISGVYIFI
>441493095_d-proteobacteria_Lawsonia_intracellularis-CDP_diacylglycerol_glycerol_3_phosphate_3_phosphatidyltransferase_
LNLANSITLRVFVIPIIVLLYGLATLLFCIAVTDLDGIARKMVTFGKFLDPLADKLLIILIFVLVVTIIIIRELAVTGLRAIAAGIIIAAGKIKTIQMVIVPLLLFFLLIILTVLSGVYFNW
>491173748_bacteria_Holophaga_foetida-CDP_diacylglycerol__glycerol_3_phosphate_3_phosphatidyltransferase_
MNLPNYLTFRILMVPILVVVLVIGILVFWAAITDLDGLARKQVTLGKLLDPLADKLLIALILVLAMVFIILREMAITGLRAIASGITIAAGKWKLGQIAISCLILYLLLVILAIWSGVYILA
>491761022_firmicutes_Thermicanus_aegyptius-CDP_diacylglycerol__glycerol_3_phosphate_3_phosphatidyltransferase_
MNLPNRLTLRVLIIPFFMVAILVALFLFLIALTDLDGLARKLITFGKLMDPLADKMLVALVLVLIMVILILREFAVSGLRMIAAGMVMAAGKLKTVQMIISLLIVLIMLAMVTLISGFYFIW
>493620763_firmicutes_Pseudoflavonifractor_capillosus-CDP_diacylglycerol__glycerol_3_phosphate_3_phosphatidyltransferase_
MNTANKLTLRVIMIPLFLVVLFVALAIFILAITDVDGIARHQVTFGKFMDPLADKLLVAMLFVTFALLIVIREFAVTALRLIAVGRVIAAGKIKTATMVICVMFVNVVMVLTTLYSGVYFVM
>493646570_firmicutes_Pseudoramibacter_alactolyticus-CDP_diacylglycerol__glycerol_3_phosphate_3_phosphatidyltransferase_
MNLPNKITLRMICIPFFVAAMFVALVIFAIAYSDVDGIARSLITFGKFMDPLADKLLTAFILVTIVVIVIIREFAITGLRTLAAGVVIAAGKAKTMQMIIIALILFIMIVALTLISGIYFVI
>493929206_firmicutes_Anaerotruncus_colihominis-CDP_diacylglycerol__glycerol_3_phosphate_3_phosphatidyltransferase_
MNLPNKLTVRMALIPVFLVFMIIAAVIFAAAFTDLDGIARRLVTFGKLMDPLADKLLVALVFILSIVFIILREFLVTSVRLIAAGTVIAAGKMKTVQIIVLVALIASFVIILTVFSGFYILL
>494110011_firmicutes_Anaerofustis_stercorihominis-CDP_diacylglycerol__glycerol_3_phosphate_3_phosphatidyltransferase_
MNLANKITLRVIMIPVYVVLMIIAGIVFIIAISDVDGIARKMVTFGKFVDPLADKLLVAMLFVIIVVITILREFIVTGLRTLAAGVVIAAGKLKTTQMILVLMHFIVLMAILTIVSGVYLLF
>495380235_verrucomicrobia_Verrucomicrobiae_bacterium-CDP_diacylglycerol__glycerol_3_phosphate_3_phosphatidyltransferase_

MNLPNLLTLRIPMMFVIVWLMFLAFALFVIAITDLDGFARKQVSFGILMDALTDKILMLMVLVRERIFLILREFMITGLRLVAAGVVMAAGKQKTVQIIVGGLLLVVFFLVMTLYSGAYFLA

>495988033_bacteria_CDP-diacylglycerol_glycerol-3-phosphate_3-phosphatidyltransferase_Synergistaceae

WNLPNMLSLRVFLVPVILVFLVIAGLVFIIAITDADGIARKLVTMGKFIDPLADKILVVLTLVLFMVVVIVREFIVSGLRMVAAGVVIAAGKLKTVQIIIVMLIFYAVMVILTVWSGVYVLL

>496551675_firmicutes_CDP-diacylglycerol_glycerol-3-phosphate_3-phosphatidyltransferase_Clostridiales

MNTANKLTMRVALIPVFLILLFFALTVFILAVTDVDGIARHQVTFGKFMDPLADKVLVALLFVALVLLVVIREFAVTALRLIAVGRVIAAGKVKTATMVICLMLLAVCTVVTTVHSGAYFVV

>497195025_verrucomicrobia_Opitutaceae_bacterium-CDP_diacylglycerol__glycerol_3_phosphate_3_phosphatidyltransferase_

LNLPNLLTLRVPLTFLVVALMWLAFILFVGAVSDLDGIARKIVSFGKFMDALTDKIFVLMVFVI-PIWITLREFMVSGMRMLASGVVVAAGKVKTLQLIIGFLLAVAGFAVLTVWSGYYFLM

>501344812_verrucomicrobia_Opitutus_terrae-CDP_diacylglycerol__glycerol_3_phosphate_3_phosphatidyltransferase_

MNLPNLLTFRLPAMFAIVALMFLAFWLFIAALTDFDGLARRQVSFGRFMDAVIDKVMVVMILVGFTIPAILREFMISGLRMVAAGVVVEAGKLKTFQLNIGWLLGVGLFLLLTITSGVYFVV

>501441106_verrucomicrobia_Methylacidiphilum_infernorum-phosphatidylglycerophosphate_synthase_

LNLPNQLSLRIGMCGLFVADLWMALFIFLLALTDLDGIARNLVSLGKLLDPLADKILIALLLLFAAVITMIREFLITGLRTLLAGKVMAAGKQKTLQMIIIGCFVLPMMLAITVVSGTYFLI

>501542775_bacteria_Dictyoglomus_thermophilum-CDP_diacylglycerol__glycerol_3_phosphate_3_phosphatidyltransferase_

MNLPNILTIRIFMLFPIVILI-LSGILFILAFTDLDGIARRKISLGKFLDPLADKILVILVFVLFIVILILREFMVTGLRLSLVGIVLPAGKLKTFQDFILLYLWFILLIVLTLYSGFYFEI

>502758672_firmicutes_Mageeibacillus_indolicus-CDP_diacylglycerol__glycerol_3_phosphate_3_phosphatidyltransferase_

MNLPNKLTIRCILTPFVVLFMNIALILFAIAITDLDGIARKLITLGKFLDPIADKILVVLIFVLVAPIIILREFAITGVRLLAIGKVIAAGKLKTVQIILIILMLLTALIVLTLLSGYYILF

>502807957_verrucomicrobia_Coraliomargarita_akajimensis-CDP_diacylglycerol__glycerol_3_phosphate_3_phosphatidyltransferase_

MNLPNILTLRIPILFLIVGFLFIAFILFVIGLTDADGYARKLVSFGKLMDALTDKVFMLFILLMLALPLILREFLITGLRLVAAGIVLAAGKHKTVQIVAILLLLLGFFILLTVSSGTYMVF

>518916663_Clostridium__Clostridium__sporosphaeroides-CDP_diacylglycerol__glycerol_3_phosphate_3_phosphatidyltransferase_

MNLPNKLTMRLILVPFFVAVLLIAAILFAVAYTDLDGLARKQITFGKFMDPLADKVLVALVFVLAMVLLIIREFMVTSIRLVAAGQVIAAGKAKTVQIIILVVLLALLIIVLAVVSGAYLIV

>524043416_firmicutes_Firmicutes_bacterium-cDP_diacylglycerol__glycerol_3_phosphate_3_phosphatidyltransferase_

MNTANKLTLRVVMIPIFIVLLFVALAVFILAITDVDGVARHQVTFGKFMDPLADKLLVAMVFVVMACFIVIRELAVTGLRLVAVGLVIAAGKVKTATMVICLLLANVCVVVTTVYSGVYFVI

>524302024_firmicutes_Clostridium_sp_-cDP_diacylglycerol__glycerol_3_phosphate_3_phosphatidyltransferase_

MNLPNKLTMRILLIPVFMVFILWAFVIFVAALTDVDGIARKMVTFGKFMDPLADKILVALVFILAVVILILREFLVTSLRLIAAGIVIAAGKYKTATMVICYALLVVLMLALTLISGAYLLI

>524459540_firmicutes_Eubacterium_sp_-cDP_diacylglycerol__glycerol_3_phosphate_3_phosphatidyltransferase_

MNLPNKLTLRIILVPFFVASLFVALVIFVAAFTDFDGIARSLVTFGKFADPLADKILVALLFVTCAVIIVLREFSVTSIRLVAAGKVVAAGKIKTVQLVIIAILLFCLIIIMVLVSGVYIFI

>529162804_diplomonads_Spironucleus_salmonicida-CDP_diacylglycerol_glycerol_3_phosphate_3_phosphatidyltransferase_

NHVPNLLSLRVPFTFIIAGLLYISFVLFVIALTDFDGIARKVVTFGQLFDALSDKVLTIQALILYFIFGVIREFFISGLRMAAAGKVLAAGKIKTAQMVICISFAFLLYILLAIQSGYYFFL
>545628039_firmicutes_MULTISPECIES_CDP-diacylglycerol_glycerol-3-phosphate_3-phosphatidyltransferase_Oscillibacter
MNLPNKLTLRIILIPVFMVVLFVALAIFIIALTDLDGIARKLVTFGKFADPLADKMLVAMLFVNMMLLIVIREFAVSGLRMIASGRVIAAGKVKTATMVVVLMFLILCVVLTTLYSGVYFVI
>651373490_firmicutes_Caldanaerobius_polysaccharolyticus-CDP_diacylglycerol__glycerol_3_phosphate_3_phosphatidyltransferase_
MNIANKLTLRIIILPFFMVFAFIAVLIFLVALTDFDGIARKQTTWGKLMDPLADKLLIALILVAVAAIIIIREFAVTGLRSLAANIVISAGKIKMVQIVITALLLFIALIIITVLSGYYFVI
>651943977_firmicutes_Fictibacillus_gelatini-CDP_diacylglycerol__glycerol_3_phosphate_3_phosphatidyltransferase_
MNLPNKITIRVMLIPIFLIVLLVAALIFIVACTDIDGYARKLVTLGKFLDPLADKLLVAFILVLTMVIIIIREFAVTGIRTIAAGVVIAAGKVKTWQIIISVTLLFISLIAITLYSGWYFVM
>651954940_firmicutes_Bacillus_megaterium-CDP_diacylglycerol__glycerol_3_phosphate_3_phosphatidyltransferase_
MNVPNKITLRILLIPVFIILMLVAGLLFIVAATDIDGYARKLVTLGKFLDPLADKLLVALILVMAVVIIIIREFAVTGLRLVAAGIVLAAGKLKTAQMIIAVLLLFWMLIFFTILSGVYFVM
>652506256_firmicutes_Peptostreptococcaceae_bacterium-CDP_diacylglycerol__glycerol_3_phosphate_3_phosphatidyltransferase_
MNLPNKLTLRIFLIPVLVVVMIISCIIFIVAITDMDGIARKLVTFGKFMDPLADKLLVVLTMIVVMVIIIVRELTVSILRAIAAGKVIAAGKLKTIQMGIIIILLLIAMILLTLYSGWYLLF
>653149359_firmicutes_Anaerovorax_odorimutans-CDP_diacylglycerol__glycerol_3_phosphate_3_phosphatidyltransferase_
MNLPNKLTLRIILIPIFIVLL-ISAVIFIVAATDLDGIARKLITFGKLMDPLADKLLVALVLVLVMVIIILREFTITALRSVAAGIVIAAGKLKTVQMVITALLLFIMLAIMTIYSGIYMVF
>696592221_firmicutes_Intestinimonas_butyriciproducens-CDP_diacylglycerol__glycerol_3_phosphate_3_phosphatidyltransferase_
MNTANKLTLRVVMIPIFLVVLFIALAIFILAITDIDGVARHQITFGKFMDPLADKLLVAMLFVAMTVLIVVREFAVTGLRLVAVGRVIAAGKVKTATMVICLMLLIVCVLATTVYSGAYFVL
>736809908_Eubacterium__Eubacterium__sulci-CDP_diacylglycerol__glycerol_3_phosphate_3_phosphatidyltransferase_
MNLPNKLTVRVIAVPFFILAF-IAFVIFILALTDLDGIARKLVTFGKIMDPLADKILVAFCLITVMLIIILREFTISGMRMVAAGIVIAAGKIKTVQMIVPLLLLFIFLAIMTVWSGIYVVF
>738733852_firmicutes_Oscillospiraceae_bacterium-CDP_diacylglycerol__glycerol_3_phosphate_3_phosphatidyltransferase_
MNLPNKMTLRIILIPVFMVVLFVALAIFIIALTDLDGIARKLVTFGKFADPLADKMLVAMLFVIMMLLIVIREFAVSGLRMIASGRVIAAGKVKTATMVVVLMFLILCVVLTTLYSGVYFVI
>738874569_firmicutes_Peptostreptococcus_sp_-CDP_diacylglycerol__glycerol_3_phosphate_3_phosphatidyltransferase_
MNLPNKLTVRICMVPLFMLFIIISLIIFAIAITDLDGIARKLVTFGKFMDPLADKLLVALTMIPVIVVVIVRELAVSIMRAIAAGKVIAAGKLKTVQMFIVFLLLFVLILILTIYSGYYFLF
>740129893_bacteria_Synergistes_jonesii-CDP_diacylglycerol__glycerol_3_phosphate_3_phosphatidyltransferase_
WNLPNILSLRVFLAPVILVFLAIAGIVFIIALTDADGIARKLVTMGKFIDPIADKILVVLTLVLFMVVVIVREFIVSGLRMVAAGVVIAAGKLKTVQIIIIMLLFFAVMVLLTILSGVYVLL
>754502410_e-proteobacteria_Helicobacter_mustelae-CDP_diacylglycerol__glycerol_3_phosphate_3_phosphatidyltransferase_
KNLPNVLTTRIVISILIIFVLFVAFWFFLIAITDFDGLARRLVSFGEVFDPLADKMLITFILLIIAVFIILREFFITGLRVVVAQHSVRAGKCKTVQMGVCLLLL-VVLVFVTLYSGLYASM
>759902839_verrucomicrobia_Diplosphaera_colitermitum-CDP_diacylglycerol__glycerol_3_phosphate_3_phosphatidyltransferase_

LNLPNLLTLRVPLTFIVVALMWLAFVLFLGAISDLDGIARKIVSFGKFMDALTDKIFVLMVFVI-PIWITLREFMVSGMRMLASGVVVAAGKAKTVQLIIGFLLAVAGFIILTVWSGYYFLI
>809019226_verrucomicrobia_Verrucomicrobia_bacterium-CDP_diacylglycerol__glycerol_3_phosphate_3_phosphatidyltransferase_
WTLPNILTLRIPLMFLIVWLTWLAFSIFILALSDLDGIARKVVSFGKFMDALTDKILVLMVFVR-PLPFTLREFIVSGMRMVVAGIVVAAGKIKTVQMIIGCLLSIVAFLYLAVWTGYYLVF
>827088859_firmicutes_Bacilli_bacterium-CDP_diacylglycerol__glycerol_3_phosphate_3_phosphatidyltransferase_
MNLPNKLTLRILMIPIFILLLLIAALIFIIATTDVDGIARKLVTLGKFLDPLADKLLVALILIMAVVIIIIREFAVTGLRLVAAGIVLAAGKIKTAQMIIAILLLIIMLIFFTVLSGIYFVM
>836601192_bacteria_Kosmotoga_pacifica-CDP_diacylglycerol__glycerol_3_phosphate_3_phosphatidyltransferase_
MNIPNILTLRILTIPIVAFLYEVSFAFFIIALTDFDGLARKQVSFGKFLDQISDKIMILLLFMIVLVFVVLRDTLVSGIRMLAASVVIAAGKAKTVQMALLFLYFITFQLLITVLSGVYINI
>913007707_firmicutes_Virgibacillus_pantothenticus-CDP_diacylglycerol__glycerol_3_phosphate_3_phosphatidyltransferase_
MNLPNKLTLRICLIPVFIILMLVSAVLFIVAATDVDGYARKLVTLGKFLDPLADKLLVALILVMAVVILIIREFAVTGLRLVAAGIVLAAGKLKTAQMVIAVLLLFIMLIFFTVLSGAYFVM
>914736555_firmicutes_Ruminococcaceae_bacterium-CDP_diacylglycerol__glycerol_3_phosphate_3_phosphatidyltransferase_
MNLPNKLTVRMALVPFFVAAVLVALLLFIVAYTDLDGIARELITFGKFMDPLADKILVALVFVLIWVVIILREFLVTSLRLVAAGIVIAAGKVKTVQIVIVAILAFLLVILFTALSGVYVVV
>930210967_firmicutes_Oceanobacillus_caeni-CDP_diacylglycerol__glycerol_3_phosphate_3_phosphatidyltransferase_
MNLPNKLTLRILMIPIFILLLLIAALIFIIATTDVDGIARKLVTLGKFLDPLADKLLVALILIMAVVIIIIREFAVTGLRLVAAGIVLAAGKIKTAQMIIAILLLIIMLIFFTVLSGIYFVM
>933238553_firmicutes_Flavonifractor_plautii-CDP_diacylglycerol__glycerol_3_phosphate_3_phosphatidyltransferase_
MNTANKLTMRVALIPVFLILLFFALTVFILAVTDVDGIARHQVTFGKFMDPLADKVLVALLFVALVLLVVIREFAVTALRLIAVGRVIAAGKVKTATMVICLMLLAVCTVVTTVHSGAYFVV
>939198283_verrucomicrobia_Candidatus_Xiphinematobacter-CDP_diacylglycerol__glycerol_3_phosphate_3_phosphatidyltransferase_
MTLPNRLTLRFSLTALFVAATFLALIFFALALTDLDGLARRLMTFGILMDPLADKVLNAFILVYLVVIVIVREFLITGLRLVAGGVILPAGKHKTIQVMIFFFLIIMFVVTLTLYSGLYLLF

Supplementary Data S9. The edited multiple sequence alignment for the phylogenetic tree reconstruction of Slc1

>27370046_rodents_Mus_musculus
VANHTSPIDVGQVHGGLMGWFERSEIKDRHLVPILIFPEGTCINNFKKGS
FEGGTIYPVAIKFGDAFWNSSKYNLVSYLLRIMTSWAIVCDVWYMPPMTR
EEGEFANRVKSAIAVQGGLWDGGLKR
>27370522_rodents_Mus_musculus__lysophosphatidylcholine_acyltransferase_2_
VAPHSTFFDVSRNENAQTPLVSRVDPDSRKNTQILVFPEGTCTNRFKPGA
FIGVPVQPVLLRLDTVTWTWQGYTFLQLCVLTFCQLFTKVEIEFMPVQAP
SEEEFASRIRNLMAEALEIHTYEDCR
>58037223_rodents_Mus_musculus__lysophosphatidylcholine_acyltransferase_2B_
SAPHSTFFDVSDSQLARVPLVKREDPNSRKTTQILIFPEGLCTNRFKLGA
FSGVPVQPVLLRLDTVTWTWNGFSGFQVCMLTLSQLFTRVEVEFMPVYIP
SEEEFANTVRIKMANALKLHSLEDCK
>73950376_carnivores_Canis_lupus__PREDICTED__lysophosphatidylcholine_acyltransferase_2_isoform_X2_
AAPHSTFFDVSRKENVQVPLVSRIDPDSRKNTQILVFPEGTCTNRFKPGA
FIGVPVQPVLLRLDTVTWTWQGYTFIQLCMLTFCQPFTKVEVEFMPVQVP
NDEEFAGRVRNLMAETLGIHTYEDCR
>115472509_monocots_Oryza_sativa__Os07g0531600_
VANHTSMIDIMQKHPGWVGWFNRNDLKDREVVPLLIFPEGTCVNNFKKGA
FEGCAVCPIAIKFVDAFWNSKKQSFTMHLVRLMTSWAVVCDVWYLEPQYL
RDGEFAERVRDMIAARAGLWDGYLK-
>116786817_seed_plants_Picea_sitchensis__unknown_
VANHTSMIDIMQKHPGWVGWFNRTEAKDRHIVPLLIFPEGTCVNNFKKGA
FEGCTVCPIAIKFVDAFWNSKKLSFTMHLVRLMTSWAVVCEVWYLEPQTL
QPGEFAERVRDIISMRAGIWDGYLKY
>123434066_trichomonads_Trichomonas_vaginalis__Acyltransferase_family_protein_

ISNHSAYHDVCKWEIGQ-SYVRRDQSGGQSKLPVLIFPEGTTHKGFHRSA
FIQHKVQPVLIRFVPRGWNWTQTNTLEYFFMCLAMPLNFVDVTFLPAMTL
AENEFAENAELLVANFFGIRSNDEIF
>148228837_frogs__toads_Xenopus_laevis
VANHTSPIDVGQVHGGLMGWFERSEMRDRHLVPILIFPEGTCINNFKKGS
FEGGTIYPVAIKFGDAFWNSSKNSMVSYLLRMMTSWALKCNVWYLPPVNR
QDGEFANRVKSAIAKQGGLWDGGLKR
>149636999_monotremes_Ornithorhynchus_anatinus__PREDICTED__lysophosphatidylcholine_acyltransferase_2_isoform_X1_
VAPHSSFFDVSRVENANALLVSRVDPDSRKTTQILIFPEGTCTNRFKPGA
FIGVPVQPVLLRLDTVTWTWQGYTFIQLCTMTFCQVFTKVEIEFLPVYVP
NDEEFANGVRNTMATILNVHTYEDCR
>156372700_sea_anemones_Nematostella_vectensis__predicted_protein_
VANHTSPIDVGQRQPGLFGWFERSEMKDRIIVPILIFPEGTCINNFKKGS
FEGGVIYPVAIKFGDAFWNSSSESFGQYLFSLMTSWALVCDVWYLKPMYK
REDEFANRVKAEIAAQGGLWDGQLKR
>156385520_sea_anemones_Nematostella_vectensis__predicted_protein__partial_
VAPHSTFIDVSRKENDKIPYVARTDPNSRQNTHLCIFPEGTCTNRFKPGA
FYGSPVQPIILKLDTVTWTWSGPGALKLLWLTMCQFHNFLEIEILPVYYP
CTEEFARNVRMQMSSALGVHTFEDTR
>156545299_wasps_c._Nasonia_vitripennis__PREDICTED
VANHTSPIDIGQRHGGFLGWFERSEVKDREAVPILIFPEGTCINNFKKGS
FEGGVIYPVAIKFGDAFWNSSRYSMIQYLYMMMSSWAIVCDVWYLPPMYR
REDEFANRVKSVIARQGGLWDGQLKR
>159113552_diplomonads_Giardia_lamblia__Lysophosphatidic_acid_acyltransferase__putative_
VANHTTIMDVGQKYSGLLGWFNRSDRTERTEAPLLLFPEGVLVNNFKKGA
FEGAEICPIAIKLSSAYWSSRDVSFYRYLFDLMTNWILIVDVWFLPPTSI

QDGEFAERVKLSIARAARLWDGYLKY
>167537509_choanoflagellates_Monosiga_brevicollis__hypothetical_protein_
VANHTTVLDTGQGHGGVIGWFDRMESRDRTTVPLLVFPEGTCVNNFKRGA
FDGRVIVPVAIKITDAFWNSKKTSFPMHLFHFMTSWALIADVYYLDPQTR
REGEFAARVKEMMANVAGLWDGYYKY
>167999847_mosses_Physcomitrella_patens__predicted_protein__partial_
VSNHIGFVDVSAKENVEMPPVDRINPASRHHAHVMLFPEGTTTNGFKTGA
FSGLPVQPMVIKYVNPCWC-DQGGPLVILFQLMTQFVNYMEVEYLPVMTP
NVHEFANRVRTEMAKALGVHNFLDIK
>168029775_mosses_Physcomitrella_patens__predicted_protein_
VSNHVTFVDVTAYENLNLPAVDRISRESRQSAHVMIFPEATTTNGFKAGA
FTGFPVQPILIRHMDPCWVAEGPVIYWLLFRLMTQFHNFMSVEYLPVIHP
TLEEFAERVRLTMARAMNTHTYEDAY
>168038034_mosses_Physcomitrella_patens__predicted_protein_
VANHTSMIDIMQKHPGWVGWFNRTEANDRHAVPLLIFPEGTCVNNFKKGA
FEDCVVCPIAIKFVDAFWNSKKQSFTMHLVRLMTSWAVVCDVWYLEPQTI
KKGEFSERVRDLICTRAGIWDGYLKY
>168039994_mosses_Physcomitrella_patens__predicted_protein_
--------------------------------PLLIFPEGTGINNFKKGA
FEDCMVCPIAIKFVDAFWNSKKHRMFTVSATKDVHWCAQTVIYNAAPDDI
LGGCIAPWVREFIL--ALLWDGYLKY
>168057779_mosses_Physcomitrella_patens__predicted_protein__partial_
VSNHIGFLDVSAKENVEMPPVDRTDAQSRHHAHVMLFPEGTTTNGFKTGA
FSGLPVQPMVIRYVNPSWC-DQGGPLVVVLQLMTQFINHMEVEYLPVMKP
TVREFASRVRSEMAKALGIHSFLDIK
>196013697_placozoans_Trichoplax_adhaerens__hypothetical_protein_TRIADDRAFT_31283__partial_

VTNHASPLDVGQKQGGLLGLFERSEANDRKALPLLIYPEGTCVNSFKKGA
FEGGTIYPVALEFGDIYWNSLAKGWLKYLIGIFTCWGLVCNVHYLPPAKI
KPNEFANRVKTEIAKHGQIWDGQLKR
>196013699_placozoans_Trichoplax_adhaerens__hypothetical_protein_TRIADDRAFT_50933_
VANHTSPIDVGQIHGGFLGWFERSEMRDRMTVPMLIFPEGTCINNFKKGS
FEGGTIHPAAIKFGDAFWNSSRESWVQYLVMMLTSWAIVCDVWYLPPRKM
EENEFANRVKAEIAEKGGLWDGQLKR
>242017203_lice_Pediculus_humanus__conserved_hypothetical_protein_
VANHTSPIDIGQRHGGFLGWFERSEVKDRHAVPILIFPEGTCINNFKKGS
FEGGTVYPVAIKFGDAFWNSSKYSMLQYLYCMMTSWAIVCDVWYLPPMQQ
KPGEFANRVKRAISERGGLWDGQLKR
>270002450_beetles_Tribolium_castaneum__hypothetical_protein_TcasGA2_TC004512_
VANHTSPIDIGQSHGGFLGWFERSEVRDRHAVPILIFPEGTCINNFKKGS
FEGSVIYPVAIKFGDAFWNSSKYSMMQYLYMMMTSWAIVCDVWYLPPMQQ
EEGEFANRVKSVIAKQGGLWDGQLKR
>296489258_even-toed_ungulates_Bos_taurus__TPA__acyltransferase_like_1B_like_
AAPHSSFFDVSASQNANIPLVTRDDPNSRKTTQILIFPEGVCTNRFKLGA
FSGVPVQPVLLRLDTVTWTWQGFTGFQACMLTLSQPFTRVEVEFMPVYIP
NVQEFANTVRIIMANALGVHTYEDCR
>302769996_vascular_plants_Selaginella_moellendorffii__hypothetical_protein_SELMODRAFT_89351_
VSNHVSFLDLSAKENAKLPPVDRAIRRSRRDAHVLIFPEGTTTNGFKTGA
FAGLPIQPMCIRCISPAWVNRSMPYVMFRLMTQLVNFMEVRVQYLPVVEP
GLRDFTETVRHMMAASLGVHTFLDMK
>302799108_vascular_plants_Selaginella_moellendorffii__hypothetical_protein_SELMODRAFT_420881_
VCNHVTFVDVTAEENLNYPTIRRESQESRNKASLMIFPEGTTTNGFKSGA
FSSSPVQPMVVRHLDPSWVADGPSAYALLFRLMTQFHNYMEIEYLPVMRP

SKQEFAERVRAEMARALNVHTFDDV-
>302819641_vascular_plants_Selaginella_moellendorffii__hypothetical_protein_SELMODRAFT_133604_
VANHTSMIDIMQKHPGWVGWFNRTESKDRHVVLLLIFPEGTCVNNFKKGA
FEDCTVCPVAIKFVDAFWNSRKQSFTMHLLRLMTSWAVVCDVWYLEPQTI
RPNEFAERVRDMIAKRAGIWDGYLKY
>302829298_green_algae_Volvox_carteri__hypothetical_protein_VOLCADRAFT_78866_
VSNHTSMIDIMQLHHGWIAWFNRTEVNDRAVVPLLIFPEGTCVNNFKRGA
FDGATVCPIAIKFVDAFWNSRREAFGKHLFRLMTSWALVCDVYFLEPQSI
QPDEFAGRVQAMIAKYANLWDGYLKY
>313233336_tunicates_Oikopleura_dioica__unnamed_protein_product_
VANHTTILDIGQSHGGALGWFERTELRDRQIVPLLIFPEGTCVNNFKKGS
FECDRVYPVAIKFGDAYWNSSQYGMMHYLMRVFTSWAIVADVYYLPVMHR
RFSEFANRCKSEIAQAGGLWDGQLKR
>313236318_tunicates_Oikopleura_dioica__unnamed_protein_product_
LGPHSTVYDVGSAYGNDFCFVDRTDRSSTSNAQLMIWPEGTTHNRFKNGA
FNGAVVQPLTLKWDTFTWCFMGPSFVQMIYLTLCQFTINVEINFLDPVAP
TEEEFAERVRKIMADSLEILTRDD--
>321476486_crustaceans_Daphnia_pulex__hypothetical_protein_DAPPUDRAFT_221699_
VANHTTPVDIGQRHGGFLGWFERSEVKDRETVPILIFPEGTCINNFKKGS
FEGSIVYPVAIKFTDAFWNSSRHSMIQYLYMMMSSWALVCDVWYLPPMHR
RPEEFANRVKAVIAKQGGLWDGALKR
>327276427_lizards_Anolis_carolinensis__PREDICTED__lysophosphatidylcholine_acyltransferase_2_
VAPHSSFFDVSRQENLMAPTVSRVDPDSRKNTQVLVFPEGTCTNRFKQGA
FIGVPVQPVLIRLDTVTWTWQGYSFNEALVLTLCQLFTKMEVEFLPVHIP
TDEEFANRVRSKMSSALGVHTYEDCR
>329663129_even-toed_ungulates_Bos_taurus__lysophosphatidylcholine_acyltransferase_2_

VAPHSTFFDVSRNENVQVPLVSRVDPDSRKNTQILVFPEGTCTNRFKPGA
FIGVPVQPILLRLDTVTWTWQGYTFIQLCVLTWCQPFTRVEVEFMPVQVP
SDEEFASTIRNVMAEALKIHTYEDCR
>330799834_cellular_slime_molds_Dictyostelium_purpureum__hypothetical_protein_DICPUDRAFT_97873_
VANHTTVMDVGQKHKGLLGWFDRAESKDRLLVPLLIFPEGVCVNNFKKGA
FENVTIHPVAIKYVDAFWNSKKQSFIRHMFNLMTSWALVCDVWYLEPQTI
RDGEFSNRVKAMIAKKAGIWDGYLKY
>330846675_cellular_slime_molds_Dictyostelium_purpureum__hypothetical_protein_DICPUDRAFT_160307_
VCNHLTDFDLVASHIKNVPYVDQTNKA-----PLLLYPEGLTNGKFQKFV
FGGHSVLPIAMKVDYINSSW----FKNFFWWMLIPYHTFS-LEFLPPVSI
NSNEFASRVQNIIANHLNIYFYSQKK
>340370774_sponges_Amphimedon_queenslandica__PREDICTED
VANHTTPVDVGQRHGGIMGWFERKIASDRRMVPILIFPEGTCINNFKKGC
FEGATIFPVVIKFADPYWNSQEQSMVTYLAMLMTSWAIVCDIDYLNPTTL
KEGEFANRVKADICRRGGLWDGMIKR
>340725736_bees_Bombus_terrestris
VANHTSTIDIGQRHGGFLGWFERCEVKDREAVPILIFPEGTCINNFKKGS
FEGGVIYPVAIKFGDAFWNSSRYSMIQYLYMTMSSWAIVCDVWYLPPMYR
NEGEFANRVKSVIARQGGLWDGQLKR
>355756782_primates_Macaca_fascicularis__Lysophosphatidylcholine_acyltransferase_2__partial_
AAPHSTFFDVSRNENAQVPLVSRVDPDSRKNTQILVFPEGTCTNRFKPGA
FIGVPVQPVLLRLDTVTWTWQGYTFIQLCMLTFCQLFTKVEVEFMPVQVP
NDEEFANKVRNVMAEALGIHTYEDCR
>357603976_butterflies_Danaus_plexippus__hypothetical_protein_KGM_07300_
VANHTSPIDIGQRHDGFLGWFERSEVKDRHAVPILIFPEGTCINNFKKGS
FEGGTIYPVAIKFGDAFWNSSRYGMLHYLLNMMSSWAIVCDVWYLPPMTR

AENEFANRVKGAIARRGGLWDGQLKR
>387593773_microsporidians_Nematocida_parisii__1_acyl_sn_glycerol_3_phosphate_acyltransferase_zeta_
IANHTTYMDIAQRQDGFMSQFERKIKANRNEVSIIVFPEGTCVNNFQKGA
FEGVPVCPVAIKLGDPYWNTKKQSFTKYFIYLITRWRTEVSVWWLPPMKA
EENEFATRVKKLISEKAGLWNGYLK-
>390353548_sea_urchins_Strongylocentrotus_purpuratus
---------IGQKHTKFLGWFERSEMRDRKNVPMLIFPEGTCINNFKKGS
FEGGRIYPAAIKFGDAFWNSSRYSMVRYLLMMMTSWALVVDVWYLPPMDR
LVDEFANRVKAAIAKQGGLWDGQLKR
>391328463_mites__ticks_Metaseiulus_occidentalis
VANHTSPIDVGQNQGGFLGWFERGEDKDRLETPILIFPEGTCVNNFKKGS
FEDAPIHPCAIRFGDPFWDSAKHGYIMYLLRMMTSWAIVADVWFMEPIRK
DKNEYANRVRSMIARRGGMWDGMLKR
>391339752_mites__ticks_Metaseiulus_occidentalis__PREDICTED__lysophosphatidylcholine_acyltransferase_1_like_
IGPHSSFMDCTKNDSKHIPFVKRSSKESRLTTQIVIFPEGTCSNGFKQGA
FSGVPIQPVLLRLNTLTWTWDGPSALKTMWLTTCQWTTKMVIEYLPVYCP
SEAEYAENVRQLMAAALGIFSYDDLR
>395505964_marsupials_Sarcophilus_harrisii__PREDICTED__lysophosphatidylcholine_acyltransferase_2_
VAPHSTFFDVSRTENIYVPLVSRVDPDSRKTTQLLVFPEGTCTNRFKPGA
FIGVPVQPILLRLDTVTWTWQGYTFIRLCMLTFSQLFTKVEVEFMPVHVP
NDEEFANHIRNIMANALGVHTYEDCR
>443684445_segmented_worms_Capitella_teleta__hypothetical_protein_CAPTEDRAFT_172192_
VANHTSPIDVGQAQGGFMGWFQRSESKDRLAVPILIFPEGTCINNFKKGS
FEGGVVYPAAIKFADPFWNSSKQSLSKHLLMILSSWALVCDVWYLPPVTQ
QPNEFANRVKAVIAQQGGLWDGGLKR
>449665131_hydrozoans_Hydra_vulgaris

VANHTSPIDIGQMQSGIMGFFERSELKDRLLVPILIFPEGTCINNFKKGS
FEGGVIYPVAIKFGNPFWNSAKESMLLYIVNMVTSWAIVCDVWYLPAAEK
LPGEFANRVKKDIARQGGLWDGGLKR
>471382116_placentals_Trichechus_manatus__PREDICTED__lysophosphatidylcholine_acyltransferase_2B_like_
VAPHTTFFDVSASGNMRIPLVTQDDPNSRKSTQILIFPEGVCTNRFKLGA
FSGVPVQPVLLRVDTVTWTWQGFSALQVCMLTLSQLFTRVTVEFMPVYTP
NDDEFANAVRINMANALGVHSYEDCK
>504142965_rabbits__hares_Ochotona_princeps__PREDICTED__LOW_QUALITY_PROTEIN__lysophosphatidylcholine_acyltransferase_2_
AAPHSTFFDVSRNENAQTPLVSRVDPDSRKNTQILVFPEGTCTNRFKPGE
IN--------ITVDTVTWTWQGYTFLQLCVLTFCQPFTKVEVEFMPVQVP
SDEEFASRVRNLMAEALGVHTYEDCR
>507968115_insectivores_Condylura_cristata__PREDICTED__lysophosphatidylcholine_acyltransferase_2B_like_
TAPHSTFFDVSASKNAKIPLVTREDPNSRKTTQLLIFPEGVCTNRFKLGA
FTGVPVQPVLLKMDTVTWTWQGFTAFQVLILTLSQPFTKIEIEFMPVHVP
SEQEFANTVRIKMANALGLHTYEDCR
>511000003_fungi_Mucor_circinelloides__hypothetical_protein_HMPREF1544_11692_
VANHTSYLDVMARHTGALGCFDRANITERKYLPMIIFPEGTCVNNFQKGA
FEGVKVCPVGIKFGDPYWD-TRKGFMHYAYYRMTRWMTTVDVVYCEPEVP
RRGEYGERIKDKIAISIELFNGMAKR
>512901362_moths_Bombyx_mori
VANHTSPIDIGQRHGGFLGWFERSEVKDRHAVPILIFPEGTCINNFKKGS
FEGGTVYPVAIKFGDAFWNSSRYGMLHYLLNMMSSWAIVCDVWYLPAMSR
RPDEFANRVKSVIARQGGLWDGQLKR
>521033827_bats_Myotis_brandtii__Lysophosphatidylcholine_acyltransferase_2B_
TAPHSTFFDVSASENARIPLVAREDPNSRKTTQIMIFPEGVCTNRFRLGA
FSGVPVQPVLLRLDTVTWTWQGLTAFQVCMLTLSQLFTRVEVEFMPVYVP

SDQEFANAVRVNMANALGVHTYEDCK
>530592402_turtles_Chrysemys_picta__PREDICTED__lysophosphatidylcholine_acyltransferase_2_isoform_X1_
VAPHSSFFDVSRLENLSAPLVSRLDPDSRRNTQVLIFPEGTCTNRFKQGA
FIGVPVQPVLLRLDTVTWTWQGYSFKELCVMTFCQPLTRVEVEFLPVHVP
TEEEFANRVRNTMATALNVHTFEDCR
>530597160_turtles_Chrysemys_picta
VANHTSPIDVGQAHGGLMGWFERSEMKDRHLVPILIFPEGTCINNFKKGS
FEGGTIYPVAIKFGDAFWNSSKYDIVSYLLRIMTSWAIVCNVWYMPPMVR
EEGEFANRVKSAIAHQGGLWDGGLKR
>543732322_birds_Columba_livia__PREDICTED__lysophosphatidylcholine_acyltransferase_2__partial_
AAPHSSFFDVSRAENLSTPSVSRQDPDSRKNTQILIFPEGTCTNRFKQGA
FLGVPVQPVLLRVDTVTWTWQGYSGTELLLMTLCQLYTRVEVEFLPVHVP
TEEEFADRVRQTMATALNVHTFEDCR
>543743246_birds_Columba_livia
VANHTSPLDVSQTHGGLLGLFERSELKDRHLVPVLIFPEGTCINNFKKGS
FEGGTIYPVAIKFGDAFWNSTKHSFVTFVFNVMTSWAIVCNVWYLPPMVK
EEGEFANRVKAVIAARGGMWDGGLKR
>545212083_odd-toed_ungulates_Equus_caballus__PREDICTED__lysophosphatidylcholine_acyltransferase_2__partial_
VAPHSTFFDVSRNENAQAPLVSRVDPDSRKNTQMLVFPEGTCTNRFKPGA
FIGVPVQPILLRLDTVTWTWQGYTFIQLCMLTFCQPFTRVEVEFMPVQVP
NEEEFADRVRNLMAQALGIHTYEDCR
>545701106_red_algae_Galdieria_sulphuraria__phospholipid_glycerol_acyltransferase_family_protein_
VANHTSLIDIGQRHGGFAGWFDREVGRDRHIVPMLVFPEGTCVNNFKKGS
FEGAQVVPIAIKYANPYWDSSQCGFLRHVWDLMTSWAVVVDVYYLEPMKR
EPNEFAKRVKRAIVHRIGLWDGFLKR
>545701739_red_algae_Galdieria_sulphuraria__phospholipid_glycerol_acyltransferase_family_protein_

VANHTTMLDLGQLHNGFVGWFHRDDLRDRDMVPLLIFPEGTCVNNFKKGA
FEDATIYPVAIKFSDAFWDSKSENFLQYLFRLMTSWALVCDVYFLPPETK
EPEEFAARVKRLVCQKAGLWDGYMK-
>556988634_coelacanths_Latimeria_chalumnae
VANHTSPIDVGQVHGGLMGWFERSEMKDRHLVPILIFPEGTCINNFKKGS
FEGGTIYPVSLKFGDAFWNSSKYSMVNYLLRMMTSWAIVCSVWYLPPMTR
KEGEFANRVKAAIARQGGLWDGGLKR
>556991508_coelacanths_Latimeria_chalumnae__PREDICTED__lysophosphatidylcholine_acyltransferase_2_
VAPHSTFFDVSRTENTNVPLVSRLDPDSRKTTQVLIFPEGTCTNRFKQGA
FIGVPVQPVLIRLDTVTWTWQGYTAKGLLFLTLCQFYTNLEIEFLPPHVP
SEEEFANRVRNTMAKALSLHTFEDCR
>557267549_vertebrates_Alligator_sinensis__PREDICTED__LOW_QUALITY_PROTEIN__lysophosphatidylcholine_acyltransferase_2__partial_
VAPHSSFFDVSRPENLAAPLVSRQDPDSRRNTQILIFPEGTCTNRFKQGA
FIGVPVQPVLLRIDTVTWTWQGYSFKQLCAMTMCQPFTRVEVEFLSVHVP
TEEEFANRVRNNMATALNVHTFEDCR
>557766400_flies_Musca_domestica
VANHTSPLDIGQRHGGFLGWFERGEAKDRHAVPILIFPEGTCINNFKKGS
FEGGVIYPVAIKFGDAFWNSSKYSMLQYLYMMMTSWAIVCDVWYLPPMYR
QQGEFANRVKSVIAKQGGLWDGQLKR
>557766402_flies_Musca_domestica
VANHTSPIDIGQRHGGFLGWFERGEAKDRHAVPILIFPEGTCINNFKKGS
FEGGVIYPVAIKFGDAFWNSSKYSMLQYLYMMMTSWAIVCDVWYLPPMYR
QQGEFANRVKSVIAKQGGLWDGQLKR
>568248771_mosquitos_Anopheles_darlingi__hypothetical_protein_AND_009803_
VANHTTPIDIGQRHGGFLGWFERAEAKDRMFVPILIFPEGTCINNFKKGS
FEGGVIYPVAIKFGDAFWNSSRYSMMQYLFLMMTSWAIVCDVWYLPPMYR

EEGEFANRVKSVIAKQGGLWDGQLKR
>568953791_rodents_Mus_musculus
VANHTSPIDVGQVHGGLMGWFERSEVKDRHLVPILIFPEGTCINNFKKGS
FEGATVYPVAIKFGDAFWNSSKYGMVTYLLRMMTSWAIVCSVWYLPPMTR
EKDEFANRVKSAIARQGGLWDGGLKR
>573907636_bony_fishes_Lepisosteus_oculatus__PREDICTED__lysophosphatidylcholine_acyltransferase_2_like_
VAPHSSFFDVSRSENLATPLVSRLDPDSRKNTQVLIFPEGTCTNRFKQGA
FIGVPVQPVLLRLDTVTWTWQGPKARTLLLLTLCQLYTNVEIEFLPPHVP
SEEEFASRVRSTMAKALGVHTYEDCR
>575481988_chytrids_Batrachochytrium_dendrobatidis__hypothetical_protein_BATDEDRAFT_20142_
VSNHTSVIDVAQKHGGLIGMFNRNEKNDRSVLPLLIFPEGTCVNNFHKGA
FENAAIVPVAIKWADAYWHSKTQSFTYHLLYLMTRWALVADVWYLEPRCL
REGQFSNEVKAEISSVAKLWDGYFK-
>585662189_hemichordates_Saccoglossus_kowalevskii
VANHTSPIDIGQEQGGFFGWFDRAEMKDRKAVPILIFPEGTCINNFKKGC
FENATIYPVAIKFGDAFWNSSKFSLLEYLILMFTSWALVCDVWYLPPMTK
KDDEFANRVKSAIAKQGGLWDGQLKR
>586692626_flowering_plants_Amborella_trichopoda__hypothetical_protein_AMTR_s00080p00126880_
VANHTSMIDIMQKHAGWVGWFNRTEAKDREIVPLLVFPEGTCVNNFKKTP
FEDCTVCPIAIKFVDAFWNSKKQSFTNHLLRLMTSWAVVCDVWYLEPQTL
KPGEFAERVRDIIAVRAGIWNGY---
>602663065_snakes_Python_bivittatus
VANHTSPIDVGQSHGGVMGWFERSEIKDRHLVPILIFPEGTCINNFKKGS
FEGATIYPVAIKFGDAFWNSSKHNILSYLLRIMTSWAIVCHVWYLPPVTR
EEGEFANRVKSAIARQGGLWDGGLKR
>620976148_monotremes_Ornithorhynchus_anatinus

VANHTSPIDVGQVHGGLMGWFERSEXXXXSLLPILIFPEGTCINNFKKGS
FEGGTIHPVAMKFGDAFYNSSKYNMVSYLLRMMTSWAIVCDVWYLPPETR
EEGEFANRVKSAIANQGGLWDGGLKR
>627949616_eukaryotes_Fonticula_alba__hypothetical_protein_H696_02426_
VANHTSLIDLGQRHVGFVGWFNRMEAKDRKMVPLLIFPEGTCVNNFKRGA
FEDADVCPIAIKFADAFWNSKKESFLGYLFKLMTSWALVVDVYYLEPQTR
HPEEFADRVKKLIARCAGLWDGYLKY
>632978409_chimaeras_Callorhinchus_milii
VANHTSPIDVGQVHGGLMGWFERNEMKDRHLVPILIFPEGTCINNFKKGS
FEGGTFYPVAIKFGDAFWNSSKYGMVTYLLQMMTSWAIVCNVWYLPPMTK
QEDEFANRVKAAIAQQGGLWDGALKR
>632981106_chimaeras_Callorhinchus_milii
VANHTSPIDVGQIHGGLMGWFERSEVRDRNLVPILIFPEGTCINNFKKGS
FEGATVYPVAIKFGEAFWNSSKYGMVNYLLRMMTSWAIVCSVWYLPPMTR
EEGEFANRVKAAIARQGGLWDGGLKR
>641647519_aphids_Acyrthosiphon_pisum
VANHTSPVDIGQRHGGFLGWFERSEAKDREIVPILVFPEGTCINNFKKGS
FEGSVIYPVAIKFGDAFWNSSKYSMIQHLYLMMTSWAIVCDVWYLPPMYQ
NENEFANRVKRVIADQGGLWDGQLKR
>642912307_beetles_Tribolium_castaneum
VANHTTPCDIGQSHGGFLGWFERSEVRDRHAVPILIFPEGTCINNFKKGS
FEGSVIYPVAIKFGDAFWNSSKYSMMQYLYMMMTSWAIVCDVWYLPPMQQ
EEGEFANRVKSVIAKQGGLWDGQLKR
>642912309_beetles_Tribolium_castaneum
VANHTSPIDIGQSHGGFLGWFERSEVRDRHAVPILIFPEGTCINNFKKGS
FEGSVIYPVAIKFGDAFWNSSKYSMMQYLYMMMTSWAIVCDVWYLPPMQQ

EEGEFANRVKSVIAKQGGLWDGQLKR
>646705360_termites_Zootermopsis_nevadensis__hypothetical_protein_L798_13043__partial_
VANHTSPIDIGQRHGGFLGWFERSEVKDREAVPILIFPEGTCINNFKKGS
FEGSVIYPVAIKFGDAFWNSSRYSMMQYLYLMMTSWAIVCDVWYLPPMYQ
QEGEFANRVKSVIARQGGLWDGQLKR
>673053251_oomycetes_Aphanomyces_invadans__hypothetical_protein_H310_14488_
VANHSTYIDAMMKEVADLPLIERRSEHGRKKAPLVIFPQGLTCNPFKKGA
FTGLPVQPVLLRHLDISW-FPSVDILPLFFRHLCQFRMYLEVTYLEPYVP
TSEEYAENVRQVMASALPAHAFEDVR
>674592879_flatworms_Hymenolepis_microstoma
VANHTTPVDIGQRQGGFFGWFDRGEVFDRSAVPILIFPEGTCINNFKKGC
FEGAVIHPVAIKYADCFWNSSQDGLFQYLVKMMTSWAMVVDVWYLPPETA
LPGEFSSRVKRKIATCGGLWDGELKR
>674878396_eudicots_Brassica_napus__BnaC09g53950D_
VANHTSMIDIMQKHPGWVGWFNRSEAKDREIVPLLIFPEGTCVNNFKKGA
FEGCTVCPIAIKFVDAFWNSRKQSFTMHLLQLMTSWAVVCEVWYLEPQTI
RPGEFAERVRDMISLRAGLWDGYLKY
>675367041_spiders_Stegodyphus_mimosarum
VANHTSPIDVGQKQGGFLGWFERFEMKDRALVPILIFPEGTCINNFKKGS
FEGGTIYPAAIKFGDPFWNSSKQGYVHYLLMMMSSWAIVCDVWYLPPMTR
QEGEFAKRVKSIISRQGGLWDGQLKR
>676439655_gastropods_Lottia_gigantea__hypothetical_protein_LOTGIDRAFT_140795_
VANHTSPIDIGQSQGGLLGWFERSEVKDRILVPILIFPEGTCINNFKKGS
FEGTTIFPVAIKFGDAFWNSSKQGMIQHLLNIMTSWAVVCDVWYLPPMNK
LEGEFANRVKREIAAKGGLWDGQLKR
>685825785_nematodes_Strongyloides_ratti__Phospholipid_glycerol_acyltransferase_domain_containing_protein_2_

VSNHLTANDTGQKHTGLIGWLERSDKNQRIEFTILLFPEGYCTNNFKKSL
FVNVCIYPIAIKLGDAFWY--EDNFASYTLRLLTSWAIVYHVRYLPVQFK
KASEFACRVQKQIANAA---------
>685825787_nematodes_Strongyloides_ratti__Phospholipid_glycerol_acyltransferase_domain_containing_protein_1_
VANHTSPIDIGQSQGGFLGWFERTEAKDRLAVPILIFPEGTCINNFKKGS
FEASTIYPIAMKLGDAFWNSSLQGYGQYLWMVMTSWAIICDVWYLPPMTR
NEDEFSRRVKKEIANKGGLWDGQLKR
>685829356_nematodes_Strongyloides_ratti__Phospholipid_glycerol_acyltransferase_domain_containing_protein_5_
TVNHMSPTDTGQKGKGTIKWVNRHSQSERKTF-IVLFPEGYCSNGFRKSI
FVNVNVYPIAYKYNDTFW-WED-DFPTFFIRQLTSLGIFYDIYYLPKLVK
KSNEFAYRTSETIAKKLETWSGE---
>685832290_nematodes_Strongyloides_ratti__Phospholipid_glycerol_acyltransferase_domain_containing_protein_4_
VANHTSPIDIGQKHYGFLGWFERSESKDRLAVPIIIFPEG---------S
FEASTIYPIAMKLGDAFWNSSNEGWIKYLWMMMTSWTIICDVWYLPPMIK
GENEFAKRVKKIIANKGGLWDGQLKR
>685836583_nematodes_Strongyloides_ratti__Phospholipid_glycerol_acyltransferase_domain_containing_protein_3_
VANHTSPIDIGQKHGGLLGWFERSEAKDRALVPILIFPEGTCINNFKKGS
FEGATIYPIAMKFGDAFWNSSRQSYCEYVFRIMTSWALICHVWYLPPMTR
FENEFANRVKKVIAQAGGLWDGQLKR
>406915842_bacteria_uncultured_bacterium__hypothetical_protein_ACD_60C00037G0006_
VANHASYLDVGKKELFNAPFVDRSDFSKSMED-VIIFPEGTFTYAFKPGA
FMNSGVCPIAIKILR----DEEYLFKPGKIHVVIN----------KPIYA
RGSDLRNKTHQAIAEHC---------
>494028610_d-proteobacteria_Plesiocystis_pacifica__1_acyl_sn_glycerol_3_phosphate_acyltransferase__putative_
MCNHISWQDIAKREVGRWPLVERGCAHSGAKV-VLTFPEGTTSYGFHRGM
FGDIPVTPIALRFHAAGWV-GDANFLPHYLETVGRPRTVAELHFGAPMGA

RAGEFAARVREAM-------------
>500030376_CFB_group_bacteria_Gramella_forsetii__1_acyl_sn_glycerol_3_phosphate_acyltransferase_
VANHTSMLDIGKKELGRIPLVDRKNQKSRLEASICIFPEGGVPNDFKDGA
FRQIPIVPIS---------HDNKKRFSYRFLTGCPGKLRVKIHNF---IC
TEGL--------ATERKNL-------
>501935167_aquificales_Sulfurihydrogenibium_azorense__acyltransferase_domain_protein_
TPNHLSYLDVSTHEVKETFFINRQSKNGIKQE-IVIFLEGTTSNGFKSSF
VEGVKIVPTCIRRDLIFYYGDMNLFKHVFNFLLNVNSVEVEVIFLNPINP
SDFKLSKYLHSEI-------------
>504009532_d-proteobacteria_Bacteriovorax_marinus__putative_acyltransferase_
VANHLSYLDVTSIEMKTTPYVER---RSRAHLDVVVFPEATSTNGFKRPL
FAGATIIPLTLN--------------------------------------

>505236696_bacteria_Candidatus_Cloacimonas__putative_acyltransferase_
VSNHTTYLDITSVEMRKNPYTNRKKYISLPAE-VVLFPEGTSTNGFRRSL
FQKCPILPVCIK--------------------------------------

>517505166_CFB_group_bacteria_Riemerella_columbina__hypothetical_protein_
IANHTSIVDVGKKELVKIPMVDRSDAKSRSEVSIVIFPEGLVPDDFKNGA
FIAEHQLPLCC----TFIGLRK----MFPFNYSKGYPGRIPVYFNGIIEP
KPED----------------------
>521518339_d-proteobacteria_Sorangium_cellulosum__hypothetical_protein_
VANHVSYLDIAKQEIGGWPLVDRQRPASGARVPVLNFPEGSTTRGFRKGI
FGGVPVVPAAITE--LCW-AGDDTFVPHYLRFSSRRESSARITFGPPLGD
HGG-LAERARAV--------------
>648625579_a-proteobacteria_Ahrensia_kielensis__hypothetical_protein_

```
AANHISWLDVAKDEVKEWAFIDRTTRLGAKAQ-IVLFPEGTTSDGFKSSL
FGSSPIQPVSVARPLAAWPGDIEIVPHLIGVISEPHLG-VVVSFGEPVEQ
CEGMQIVQERVA--------------
>654615253_g-proteobacteria_Solimonas_soli__hypothetical_protein_
VCNHASYIDVVQDGAERWPFVNRREARAGART-LTIFAEGTFKPEFKAGA
FLGVPVVPAAIRFGG--------------GRRLPRWSAL-HIEIAPPLKA
DGSE---------EAALAL------R
>EST49318_1_Acyltransferase_Spironucleus_salmonicida
VCLIANFVNIQRYYLQFICFLSKAEMKKVPIIPIIVFPEGTISNQFKTGA
FRKDEVQPLCIYFQ-IEWL--TEHALHHIYTLFTSPFSRTKYYFLEPMKR
FENEFADRVGKKMADEMGIWMGDHAH
>EST48751_1_Lysophosphatidic_acid_acyltransferase_Spironucleus_salmonicida
VSNHASTLDVGQKHGGIQGMFDRTAKKEREKVPLLIFPEGVCVNNFKRGA
FQGAEVMPVSIKSVHAYHNSKEKNFIQYMISIFTQWELDYKIEFLPAMKI
EEGEFAHRVQLAIAKAGGLWDGFLKY
```

Supplementary Data S10. The edited multiple sequence alignment for the phylogenetic tree reconstruction of PLB

>672964254_Bifidobacterium_cuniculi_peptidase_C45_acyl-coenzyme_A_6-aminopenicillanic_acid_acyl-transferase
IGFQHGYLLMQGMADGFCSAFVAHESFDDFWSGQYFN-VCMQTVPGFIDSMTDFYETTIAGFVIPEYVRGGYANIWLIARFEQ--ELLRTKDPRIRNL--G--YNDARRARFEQLILADKFICAHYDVGSCD
GKLYDRPS-QPW
>517252862_Burkholderia_sp__JPY347_phospholipase
RGEQHGQLLIQGIAAG-CSAFIAHNSWDRYAAGDAFNVVFMQGLPGCISSLTDFWETTISNFAAPEFYRGGYANSWLIARYEL--GFESTKDLKIRNQ--GEDYTDARRLRFMQLMIADHHICGHLELGAND
GKMRDRPS-WPW
>146162174_Tetrahymena_thermophila_Laminin_A_family_protein
AGFLEGLASLVGLKNGYCSAFFKHSTWEPFSSMNRIYKFYMSSYPGAITSTDDFIETSISSQNIPDFIRKMYSSEWMFIVLSSSYDKTQELNKIIRDYLGNDLFSLPRNLIFKHFMLHNSYLGARGDIGEVD
VKHQGQPTAWNF
>551613722_Emiliania_huxleyi_CCMP1516_hypothetical_protein_EMIHUDRAFT_63066__partial
G-YLSAERIMDGLRAGVCSMAVKHNTWGGYHMMLRVHKRYMSSYPGALVSGDDFFETTLPSYNVPYWIRGTYNNAWILTVGEQLPDATATLYPETARRVGGERFSYPRAQIFRRDLRYNQFLACRADLGALD
AKHEGHPDRFRF
>459174469_Ciona_intestinalis_PREDICTED__uncharacterized_protein_LOC100170064
TGH-FCSPSAQGLIDGYCSALVKHATWTNYRSMLRMMKRYFPSQPGQLYSKNDFYETSMSNFNVLEWIRGTYNNQWMLYLLEQMPDITEHLFKETNKLSQKS-FSYTRAKIFARNMRYNNYIAARGDLVATD
AKHKGMPDKYDF
>514685344_Salpingoeca_rosetta_hypothetical_protein_PTSG_12806
AGYTEGKLTLLGVCEGYCSALIKQDTWSSFNSMTRIMKRYFSSYPGVLFSGDDFYETTIGNSNVLEWLRGTYNNQNFLYMVEQIPDVTSKLDPEIRKLSGGSWFTYARARIFRRNMRYNNYIACRGDLVATD
SKHHGMPDAWTF
>300175895_Blastocystis_hominis_unnamed_protein_product
AGLIEGYLSVDGVYDGYCSALVRQSTWSALHTMFKLHKIYFSTYPARISSGDDYYETTIGNYNVMQWARGCYNNQWMFFLLEQIPDATDVLDPFIYEMSGGKREKWPRARIFARDLRYNDFIGCRNDLAATD
GKHWRLPEKYEF
>300123434_Blastocystis_hominis_unnamed_protein_product
AGFLEGALTVNGIYDGYCSALIKHVTWSDAQSMYRIYKAYMPAYPGRFTSGDDFYETTIAIWDVLQWARGLYNNQWMFWIFEQIPDMTWWLFPAIYEACGAKAMSWARAQIFRRDMRYNDYISSRSELMGTD
VKHEDYPERYDF
>560138182_Haemonchus_contortus_Phospholipase_B_domain_containing_protein

AGFVEGKATVIGMIDGYCSALIKHVTWESYSTMLRAQKKYFSGYPGVITSNDDFVETTIGNNNIMCWIRGTYNNQWNLHVLEQIPDLTHVLFPKIFKWADGDWYTYPRALIFRRDMRSNDYISCRSDLGSTD
VKHHGQPIRWEF
>193648093_Acyrthosiphon_pisum_PREDICTED__putative_phospholipase_B_like_2
AGYLEGTLTLDGLYDGYCSALIKHATWSGYETMLRIQKRYFSSFPGGIQSGDDFYETTIENYNVLEFVRGTYNNQWMLHVLEQIPDFTGHLFPFIFNVSGGSWFSYPRARIFARDMRSNNYISSRNDLGATD
VKHLGHPDCFNF
>170045958_Culex_quinquefasciatus_conserved_hypothetical_protein
AGMLEGALSLDGLEFGFGMMLLKHTSDGSYASMLRMVKKYFTGYPATLASLDDFYGIKIANDNVALAPRSTGAKQWLVNFEEHASDMTEAIFKELADIGR------QDKILNNITIRHSAYYSGPLY-----
-KHQGQPDVWDF
>195127387_Drosophila_mojavensis_GI11981
AGILEGSLTLEGMETGYASMLTKHSTAGSYSSMLRIQKRYFTGYPGIVGSTDDFYGVGIKNENVPLVARITGAKQWIVNEKDNAADVTEHFFDEILKASG------TAAEDAELTLRNHGFFAGPIFLAAMG
DEHEGHPDEWNF
>646721955_Zootermopsis_nevadensis_Putative_phospholipase_B_like_lamina_ancestor
AGLLEGSLTLDGLETGWSSAFVKHNSGGLYQSMLRVLKRYFSSYPGVIHSQDDFYGTAF-SYNVLVGPRGTGSKQWLPKQQNAVKDQTRMLYQ---DTSH------HDDQLDATT-SSSSFISGPPVT----
NNHIGLPDLWQF
>512930550_Bombyx_mori_PREDICTED__putative_phospholipase_B_like_lamina_ancestor_like_isoform_X1
AGIVEGALTINGILTGWSSAFLRQNAAGRYSSMTRILKRYFSSYPGSITSQDEFYGTALRNYNVPAGPRGSASKQWLNDVRHTIYDISDAFFKNVAEKTHV-----QKGYTNATTFEASGYVSGPPYTTKEF
DNHRGLPDKWAF
>572300312_Apis_dorsata_PREDICTED__putative_phospholipase_B_like_lamina_ancestor_like_isoform_X2
AGLLEGSLTLDGLEAGWSMIYFKHHTSAS-----------MSSYPGALSSRDEFYGTSLLVTNVMLTVRGASALQWIISYIGISNHL-MRLFRNMMRG--------SQ-EGCSA-LQELMFISGP-------
--HVGHPDTFNF
>307214880_Harpegnathos_saltator_Putative_phospholipase_B_like_lamina_ancestor
AGLLEGSLTLDGLEAGWGMICLKHTTAASYAKMLRLLKRYMSSYPGALSSHDEFYGIPLSVPNVMSSAENTSARQWILCRTGKNRELVARLFRRLMRG--------SHKDKTTIAAQILTYTSGPNT-----
--HIGHPDVFEF
>242007586_Pediculus_humanus_corporis_conserved_hypothetical_protein
AGMLEGSLSLEGMQQGFSSSFSAKKLIQGRSDNVESMEKLLNSFQNMRNYFSPIIGTPITVFNLLSGVRGTGNKQWLFWVAEQLPDLTENLYKDMYEMSGGDAFSYPLAKIYRRDMRSNSFLEKKLENGPAH
SLHNGHPNNWDF

>241732653_Ixodes_scapularis_secreted_protein__putative
AGAVEAYLTLAGLSDQFCSALIKHNTWFGYRKMLRIEKKYMSSYPGKLVSLDDFYETTIENSNPLTWVRGTYNNQWMLWILEQMPDVSAYLFPAIFNISGGNYYSYPRAQIFRREMRYNNYIAARYDLAAID
MKHEGHPDKWMF
>123474028_Trichomonas_vaginalis_G3_Laminin_A_family_protein
AGYIEGIFTIEGMVEAYGTVIVGQTAWRTYGESVRIAKRYLSSYPFMLHSDDEFCSTSIITTNYPYWFRGT-GIEYIVLAVDEIPDMTSRLVESIFKTAGPEWYSYARAKILQRTIRLNDKIAGRFELGAFD
AK----------
>471208388_Entamoeba_invadens_IP1_hypothetical_protein_EIN_182450
TGFADGYFSVRGITAGHCSGLVKQAAWFYYGAMTRVMKYFFSSYPGFVFSFDDFYETTFSNFNILDWLRGTYNNQWIFTVSEQLPDFSKSLIQETIDMSNDKFWNSCRANIFRANMKYNKWIGARYDLGQMD
AKTDGVVKVWNF
>558594890_Spironucleus_salmonicida_Phospholipase_B
VGYAEGKLFFDGMVLGYCSGLVRQTAWFFYGSMTRVWKEYFSSYPGFSYSFDDFYETTNNIYNVLTWIRGTYNNQWVMWVLEQVPDGTEQLLKNVRKVSGSDDYDA-RNLIFQRDMRYNDYISSRYDQGGFD
NKRRGLPD----
>159109313_Giardia_lamblia_ATCC_50803_Phospholipase_B
VGFAEGKIFFDGLVEAYCSGLVYQTAWFFYGGMTRVFKGYFSSYPGFSYSFDDWTETTANVLNVLTWIRGTYNNQWIVWVSEQMPDVAHDMFDELWIVAGSYRYDK-RGLMFARDMRYNKWISARYDLGGYD
AKKSTL------
>471208514_Entamoeba_invadens_IP1_hypothetical_protein_EIN_184180
AGMVEGYLTTFGVREGACTGLIKQDTWSSPTTMNRIMKDYFSSYPGVTHSLDDFWETTMHCWNVLTWIRGTYNNQYILYAIEQMPDRTQELFADVYEYAGNDWYFDDRMKLIQRDMRYNNWILSRYDLGGLD
SKYNGLPTDWKF
>123449793_Trichomonas_vaginalis_G3_Laminin_A_family_protein
AGALDGYLGFDGLVEGYCTGLITQAAWSSFLKMHQVLKEYISTRMGALASSDDFWETTNNNFNLLTWIRGTYNNQYVLWVIEQLPDLTDILFEEIFNTAGGDYY-SSRFLIFNRNIRYNHYPLSRYDLGGLD
TKYDGLPKVWNF
>154412091_Trichomonas_vaginalis_G3_Laminin_A_family_protein
AGYLEGVLAGQGIIDGYCTAGIRHNAWSDYRNSIGCVIDYLTTLAGLISSLDDFYETSLMSQDLFCGYRGTYNNDYFVWLVEQLPDVTGNLSEDLYYVLQEYYLPYPRYLISARDSRYNNYISSREDLGAFN
QKHIGIPDTLNF
>123433661_Trichomonas_vaginalis_G3_Laminin_A_family_protein
AGYIEGALSFDGLVAGYCSGLIRHDAWSDYRELHGQLKEYMSTRIGKLASYDDFYETTLNNYNLFTWIRGTYNNQYVIWIIEQFPDVTDYLHEDLYNLAGGDYRSNPRYLIMQRDMRYNQWIASRYDLGDLD

TKYYGLPTRWNF

>123484021_Trichomonas_vaginalis_G3_Laminin_A_family_protein

AGIVEGYLSIKGINEGYCTGLIRHDAWSDYRDLHGQLKEYLSTRPGKISSYDDFYETTMSIFNISTWFRGTYNNQYVIWVVEQVPDVTEYVSKFLFDLMGALFY-TARYRLMEREMRYNNWIMSRYDQGGLD

TKHDGLPQEWNF

>123474499_Trichomonas_vaginalis_G3_Laminin_A_family_protein

AGAVEGYLAAKGVYEGYCTGLVRHDTWSNILALHGTLKEYLSTRPGQIASADDFYETTMGIFNVSTWMRGTYNNQYLLWVIEQSPDITDKLFPELSKLFMASFW-DARNLLIEREMRYNNWIMSRYDLAGLD

TKRIGLPDRWNF

>123418314_Trichomonas_vaginalis_G3_Laminin_A_family_protein

MGYIDTAISFRGMVNGLCTGLLKQDTWSDVRELHAYLKSYISTRTGHLSSVDDFWETTLHCFNVLTWMRGTYNNEYIVWMIEQLPDITDKFFEDIFNLADKDFW-SPRYKVIVKHMRWNKYILARYDLAGLD

SKNYGLVDTWKF

>159118002_Giardia_lamblia_ATCC_50803_Phospholipase_B

AGYLEGYTMLQGLTHGYCSAAVRHDTWTSFGSGNRAMKHYFSSMPGYFSSIDDFYETTYHTFNVLTWMRYTYNNMWVLYVNEVVPDVTEMLDEELFVISGDPYWSFSRYKIFTREMRYNDYIASRYDLGCTD

SKRHGVPDTLPD

>558600934_Spironucleus_salmonicida_Phospholipase_B

GGYAEGYATLEGITKG-CSAAIVHDTWTSFYSGVRIAKTYFSSMPGVFFSIDDFYETTYHTFNALTWLRYTYNNNYLLYTLEVVPDATDTLNEDLLELAGEDYWSYSRYCIAFKQMRYNDYISSRYDLGATD

SK----------

>159114385_Giardia_lamblia_ATCC_50803_Hypothetical_protein_GL50803_115159

MGYAEGYLLISGLHAGYCSGCVRHATWTSWGFGLRVMKIYFSSYPGFVSSVDDFYETSYNIMNVLTWSRYTYNDNWLLTSLEVVPDATSKLDP-YVRKYGDP---YPRYSIFMREMRHNSYIASRYDLGATD

AKHRGVP-----

>167523280_Monosiga_brevicollis_MX1_hypothetical_protein

A-YAAGYAEFDGMFNGYCSAIVKHTTWCSYSQMLRLYKTYFSAYPGLIVGIDDFYETTNVVFNVMYWIRGTYNNQWMFVVSEQLPDQTSVLYPETYNMSGGTKYSYPRAKIFRRDMRENHYIAARRDLGAID

AKHYGHVTTFDF

>514684492_Salpingoeca_rosetta_hypothetical_protein_PTSG_09026

A-FSAGYVELNGLVAGYCSAMVKHTTWCPYSQMLRIFKVYFSGYPALLESLDDYYETTNGIFNLPYWVRGTYNNQWMFWVSEQIPDKTMDLYPWIYNVSNGDDYTYPRAKIFRRDIRMNHFIAARRDLGAID

AKHYGHAQRFGF

>294948268_Perkinsus_marinus_ATCC_50983_conserved_hypothetical_protein

```
MGLAEGYLTLDGMMEGLCSALIKHNTWTTYTEMLRVYKSFMSSYPGYISSTDDWVETTTESMSVTTVIRGTYNNQWMFWLIEQMPDMTAKLFKDIQDAGGGQWYKYPRARLFREWMTSNYWIAGRYDIGCID
CKHWGMPDRWQF
>72389124_Trypanosoma_brucei_brucei_TREU927_lysosomal_endosomal_membrane_protein_p67
VGFGEGYATIDGMVAGYCSAFVKHATWSSFNTMLRQYKTYMSSYPGLAHSVDDWYETTNVIHNVATFLRGAYNNQWMFWVLEQLPDMTSVLFPNVYNLSGGDFYSYSRARIFERDMRYNNYIAARGDLGAID
AKTYGLLKLYNF
>528228776_Angomonas_deanei_lysosomal_endosomal_membrane_protein_p67
VGLAEGYATMAGLVDGYCSAFIKHVTWNSYESMMRQYKTYMSSYPGLVFSLDDWYETTFGLSNVPTFLRGTYSNQWMFWVGEQLPDLTQTLFEQVYQESGGELYSYSRARIFRRDMRKGDYIASRGDLGGTD
VKIWGLPVEYDF
>391336144_Metaseiulus_occidentalis_PREDICTED__putative_phospholipase_B_like_2_like
AGALEGTLSMAGIRDGYCSLLVKHSAWTSYRTMLRVQKKYMSSYPGKLNSMDDFYETTNNVFNP-TGFRGTYNNQWVFWILEQLPDMTEVLFPAIYNESGGDFYGHPRAKIFRRDLRYNDYIASRYDLGAID
AKPLGHPDVFDF
>290991259_Naegleria_gruberi_predicted_protein
AGFIEGFTTVEGLFAGYCSAFVKHATWDTFQNMLRIFKHYFSSSPGWLVSVDDFYETSNNIFNLFYWIRGTYNNQWILYILEQIPSVSHILFPYIYNISGGDFWSYPRANIFRRDIRQNKYVAARCDLGAID
AKHTDQPVRFAF
>669163350_Saprolegnia_diclina_VS20_hypothetical_protein_SDRG_12931
AGYAEGALTIEGLADGYCSALIRHTTWDIFSAMNKMFKHYMSSSPGYISSVDDWYETTNGVYNVLSWMRGTYNNQWMLTILEQMPDMTHVLFPAIYNATGSSDWTYARAEIFRRDMRYNDYIAARYDLGGID
SKHRGQPTAFKF
>569378683_Reticulomyxa_filosa_hypothetical_protein_RFI_23087
----------D--FLTYCSAIVKHSTWSEYFWMNRVFKHYFSSYPASLSSIDDWYETTNDIFNLLSWQRGTYNNQWIVWIAEQIPDVTSEVIEYIYKISGGPSLSYPRARIFRRDMRYNNYIMSRYDLGGVD
TKHIACPIKYDF
>290980526_Naegleria_gruberi_phospholipase
AGYLEGVLTMEGLLDGYCSAIFKHNSWFVFAAMTRIYKTYFSGYPATLASNDDFYETSNNIFNLLCWQRGTYNNQFMFWVVEQIPDQTQILYKEVFTISGSDMLSYVRANIFRRDLRYNNWISSRNDLGGYD
TK----------
>470492121_Acanthamoeba_castellanii_str__Neff_Phospholipase_Blike_protein__putative
AGYLEGAMTFDGLVKGYCSALIKHTAWFSYESMLRIAKSYFSSYPGFLVSLDDFYETSLSVFNLLSWQRGTYNNQWMLTILEQIPDQTSILYPRVYEAAG--MASYCRANIFRRDLRYNDWIAARFDLGGYD
TKHVGQPLRFHF
```

>676384759_Aureococcus_anophagefferens_hypothetical_protein_AURANDRAFT_24092__partial
-----GYLTFEGVVDGVCTVLITHATWFHYAMMNRIYKHYFSSYPGTLSSFDDFYQTTNGIYNLPSWQRGTYNNQYMLYVVEQIPDATAELFEEIYDKSGARGSEYPRANILRRDMRANGYVCARGDLGCQD
AKATGSTQSEAI
>302836638_Volvox_carteri_f__nagariensis_hypothetical_protein_VOLCADRAFT_90360
AGFLEGYLTADGLLAGYCTALVKHVTWWSYLSMLRIYKHYFSSYPGQISSADDWYETSLDTYDLLSWQRGTYNNQYLLTISEIIPDATDDLFREVYEAAGAQGISYPRAKIFRRDLRSNGWLCARGDLGCYD
GKYRGMLDVYD-
>545357273_Coccomyxa_subellipsoidea_C_169_laminin_A
AGFLEGFISFDGL---YCSALITHSTWDSYSQMTKVFKHYFSSYPGELFSDDDLYSTTNKMFNLVSWQRGTYNNQYMLWVAEQIPDLTEQLFPQ--EM--VAWLSYPRASIFRRDMRSNNFVCARGDLGCFD
TKHAGMPRVFDF
>302830318_Volvox_carteri_f__nagariensis_hypothetical_protein_VOLCADRAFT_103148
AGFLEGYLTFEGLVGGYCSALIKHSTHDSFTAMTRIYKHYFSSYPGELFSDDDFYETTNHIYVVLSWQRGTYNNQYMLWVVEQLPDVTAELFPEVYVAAGHQ---G-RLTAM---LKY---IFRR---GVVD
S-HRGMPDTFNF
>545364488_Coccomyxa_subellipsoidea_C_169_laminin_A__partial
AGFLEGYLTHEGLMDGYCSALVKHSSWFTYSGMVRIYKHYFSSYPGELSSDDDFYQTTNDILDVLNWQRGTYNNQYILWVVEQIPDQTEILYPEIYRFEAWF-IIYPRATIFRRDMRSNNYICGRGDLGCYD
SKHQGQPRVFDF
>281210921_Polysphondylium_pallidum_PN500_phospholipase_B_like_protein
-GFIEGVITFDGMVAGYCSALVRHTTWRYYYGMMRIFKTYFSSSPGFLSSKDDFYETTNEIFNFLVWQRGTYNNQWMFWILEQIPDVTPELFPYIYNISGGNSYSYPRAQIFNRNMQFNNYISSRADLGGVD
SKHIGLPDLWDF
>290994795_Naegleria_gruberi_laminin_A_family_protein
FGDNNEWVKFNGLVQGYCSALIKHTTWRGYNIINKMYKVYFASSPSFLSSKDDFYETTNDVFNVLCWVRGTYNNQWMFWILEQIPDVTDVLFEHIYNISGGNDYDYSRALIFKRELQFNEWVSSRYDLGGID
TKHLGQPQTYNF
>290980990_Naegleria_gruberi_phospolipase_B_like_protein
-GMAEGLVSFDGLVDGFCSALIKHTTWRRYNIINKFFKTLMSSSASFFSSKEDYLETSLSTYNLLSWVRGTYNNQWMLWIIEELPDVSSIVFPEIYNMLGGNEMDYPRAKIFARELQFNEWIAARYDLGGAD
TKHLGQPDTYDF
>167533173_Monosiga_brevicollis_MX1_hypothetical_protein
MGLLEGALSARGVQDGYCSGLVKHTTFNAYWCMLRIYKHYFSSRPGDLHSKDDYFETSLTVFNVPCWMRGTHNNQWIVSMLEQMVDLSAVISPVIFEISGAQGYSYTRARIFRRDLNANDYISARCDLGGID

CKHAGMPTQLNF
>146182316_Tetrahymena_thermophila_Laminin_A_family_protein
AGYLEGYLTLNGTYDGYCDAYVKHSTFNIYSLMLRVYKAYFSSRPGDLESKDDFYETSLNNYNVPCWMRGTHNNQWIVWMVEESFDVTYQLNQEIFNITMGYGFNYPRAIEFAQYIRLN--IAPRFDLGAID
AKHQGMPDNYNF
>145534678_Paramecium_tetraurelia_strain_d4_2_hypothetical_protein
AGRYEGEESLENMYEGYCNSFYKHSTFNVYQTMLRIYKSYFTARPGDLESKDDFFETSFNNYNVPCWMRGTHNNQWVIFMGEEAFDLTNTLDQEIFDSLKTTSYN-VRARQFRMYVRYN--LSPRCDLGGID
GKHYGMTDEWRF
>294933021_Perkinsus_marinus_ATCC_50983_conserved_hypothetical_protein
-GYLESYFLTQGYIDGYCTGLIRHNTWRLYEGFIRVFKRIMSSTPGLIHSKDDFYETTNAMHDALSWQQGTYNNQWMAMIAELSVDVSSVLFPDVFEQMGS----YSRSLISDRDSRYNEYIASRYDLGAID
AKHVGMPDRYDF
>167379235_Entamoeba_dispar_SAW760_hypothetical_protein
-GYIESYLTFRGLWNGYCSAYIKHNTWRPYYAMLRIYKHYFASSPGLIHSKDDFYETTNSFFLLLSWQRGTYNNQWMLYIGEQMPDVTSILIKEIFESSGKKEYNYSRALIFTRDMTMNHYIAARYDLGAVD
CKHWGTPRVYNF
>67479823_Entamoeba_histolytica_HM_1_IMSS_hypothetical_protein
-GFLEGYLTFRGLLSGYCTAIVKHNTWRPYYAMMRIYKKYFASSPGFLHSKDDFYETTNSIFNVLSWQRGTYNNQWILMIAEQIPDVTKLLSKKIRDLSGGDRYSYPRRRIFDREMLLNKFIAARYDLGAVD
CKHKNLPDRFDF
>167378009_Entamoeba_dispar_SAW760_hypothetical_protein
-GYLEAYLTFNGLVDGYCSALVRHGTWRGYYAMLRIYKVYFSSSPGLIHSKDDYYETTNSVHDLLSWQRGTYDNQWILFICEQMPDVTEYL---I-----------G-LHII--DMLYNDYIASRYDLGAID
AKHVGVPEKFNF
>145537896_Paramecium_tetraurelia_strain_d4_2_hypothetical_protein
AGYLEGSLTNDGLFQAICSSLIKHTTWTSYQNMLRIYKYYFSSKPGLLYSKDDFYETTNSILDLLTWQRGTYNNQYIIWISETVPDVTAKVTTEVYDRAGPIKYSYPRGQMMNRDIRYNDYISSRLDLGSID
GKHWGQPTTFNF
>300122096_Blastocystis_hominis_unnamed_protein_product
-GYLEGYVEVEGLTNGFCSALVRHATWRNFAAMLRHYKFYFSASPGFVNSKDDFYETTNAIYDVPSFIRGTYNNQWMMWIVEQIPDVTRTLIREIYEKSGENDY-YSRHNIFQRDLRSNDYIASRYDLGAID
SKHIGHPDVFDF
>470301806_Capsaspora_owczarzaki_ATCC_30864_mannose_6_phosphate_protein_p76

-GFIEGALTFDGLGHGYCSVLIKHATWRDFGQLIRLYKFYFSSSPSFLSSKDDFYETTNSILDVLCWQRGTYNNQWQFWIIEQIPDVTGFLSPMIYNLSGGNQFSYPRATIFRRDMRYNEYIASRYDLGAID
GKHLGHPDTFDF
>66817926_Dictyostelium_discoideum_AX4_phospholipase_B_like_protein
AGYLEGYLTQIGLYEGYCTSIIKHTSWADFSVMIRIYKRIFSSYPGLLVSIDDFYETLNTILNFMYWVRGTNNIQFVLWIVEQYPDVTLTLFEEVFDILGGDLFTYPRANIFRRDIDYNQYINARFDIGGID
GKHQYMPQIYNF
>330797815_Dictyostelium_purpureum_hypothetical_protein_DICPUDRAFT_150991
AGYLEGYLTFEGLTQGYCSALIKHVTWGSYFTMLRIFKRVFSSYPGVLTSDDDFFETTNDILNLLYWVRGTYNNQWMLWIVEQLPDVT-DIFPKVQEAMGGDILSYPRAKIFRRDMRYNEYISSRYDLGGID
AKHVGLPNVFNF
>66809709_Dictyostelium_discoideum_AX4_phospholipase_B_like_protein
FGYLEGYLTINGMLDGYCSALIKHTTWSGYYTMLRIFKSYFSSYPGALISVDDFYETTNSLVTVLSWMRGTYNNQWMLYVLEQIPDQTQALYETIYDASGIYYMSYPRAEIFRNFLRYNDFIASRYDLGATD
TKHLGCPEKYNF
>281208626_Polysphondylium_pallidum_PN500_phospholipase_B_like_protein
AGYIEGYLTISGMVEGYCSSFIKHTTWTYYTSLIRTFKYYFSAYPATLSSIDDFYETTNNIYNVLSWIRGTYNNQWILYILEQIPDQTKILYENIYNMSGQMQISY-RAEIFRRDMRYNNYICSRFDLGGTD
SKHIGMPDEYNY
>281206970_Polysphondylium_pallidum_PN500_phospholipase_B_like_protein
AGFIEGYLSMSSMVDGYCSSLIKHTSWSSYYVMVRMFKSYFSGYPATLASCDDFYETTNGLLNVLTWMRGTYNNQWMLYVLEQIPDVTNILFENIFNMSGSDYEAYPRSQIFRRDMRYNNFISSRFDLGGID
SKTIGMPNTWNF
>470451087_Acanthamoeba_castellanii_str__Neff_putative_phospholipase_blike_2__putative
AGYLEGRVTLRGIHQGYCSAYVRHTTWADYYNMVRVFKHYFSSYPAFVSSVDDFYETTNGITNVMSWIRGTYNNQWILHVVEQIPDVTHYLFESIFNESGGDWFNYPRAYIFRRDLRYNDWISSRFDLGGID
SKHIGQPKLWNF
>471411717_Trichechus_manatus_latirostris_PREDICTED__phospholipase_B_like_1
AGYLEGYLTLDGLYMGACSALIKHSSWYTYAAMLRMYKHWFSSYPGFLESLDDFYQTTNSVFNLLAWQRGTYNNQYMLYVVEQIPEQTEILHEKIYNWSGGMDYSYPRAKIFRRDMRYNNYICCRGDLGCYD
TKHQGMPEAYNF
>512903832_Bombyx_mori_PREDICTED__putative_phospholipase_B_like_2_like
AGFLEGFLTLEGLAAGYCSGLVKQVTWNSYQSMLRIQKMYLSSYPAFVQSTDDFYETTIGNSNILEFVRGTYNNQWYLWVVEQLPDITEELFPSVFNLSGGDWFGYPRAKIFKQKMRYNDYVAARNDLGATD
AKHVGHPDVWMF

>25152678_Caenorhabditis_elegans_Protein_F09B12_3
AGYLEGLLSVSGLIDAYCSGLIKQVTMSGFQNMLRVIKLYFSSYPGLLYSSDDFAETTISVFNLPTWIRGTYNNQWAFYVLEQMPDLTWFVFKEITEISGGDWFKWPRAKIFERDMRYNDYISARGDLGALD
YKHVGHPDEWKF
>541048601_Ascaris_suum_phospholipase_b_like_3
AGLAEGILTVTGIWHGYCSGFAKHVAMSGYNTMNRVLKVYFSGYPAGISSADDFVETTFAIYNLHCWIRGTYNNQWVLWVLEQVPDMTWFIFTVISEISG-DWYKWPRAKIFDRDM----------------
----
>156380850_Nematostella_vectensis_predicted_protein
AGMAEGFITVEGIKKGYCSALVKHVTWDTYLGMLRIFKMY------------DLRETTIGNTNLLEWVRGTYNNQWMLSVLEQIPDMSVYLFPTIYNMSGGSWFDYPRAQIFKRDMRWVNC-STKED-HP--
-QPLGHPDKFDF
>669314293_Trichuris_suis_hypothetical_protein_M513_01416
AGVLEGNLTLAGLEDGY-----------------------MSSYPGVLLSIDDYYETTIGNDNVPSWIRGTYTNQWMFYVLEEIPDMTRLLFQAIFNASNGDWFTYPRALIFKREMRYNNFISARNDLGGTD
VKHYGHPDVFSF
>301619729_Xenopus__Silurana__tropicalis_PREDICTED__putative_phospholipase_B_like_2
AGVAEAAVTLKGLEDSYCSALVKHDTWNTYQSMLRIIKKYFSSYPGTIFSGDDFYETTIGNSNVMEWLRGTYNNQWMLTILEQIPDKSDVLFQEVFNASGGDWFTYPRALIFQRDMRYNDFISARSDLGGTD
MKHMGHPDLWKF
